# Methylation-directed regulatory networks determine enhancing and silencing of mutation disease driver genes and explain inter-patient expression variation

**DOI:** 10.1186/s13059-023-03094-6

**Published:** 2023-11-28

**Authors:** Yifat Edrei, Revital Levy, Daniel Kaye, Anat Marom, Bernhard Radlwimmer, Asaf Hellman

**Affiliations:** 1 Department of Developmental Biology and Cancer Research, The Institute for Medical Research Israel-Canada (IMRIC), The Hebrew University-Hadassah Medical School, 9112102 Jerusalem, Israel; 2 Division of Molecular Genetics, German Cancer Research Center (DKFZ), Heidelberg, Germany

**Keywords:** Gene regulation, Gene regulatory-domains, DNA methylations, Enhancers, Silencers, *Cis*-regulatory elements, Cancer driver genes, Glioblastoma, Expression variation

## Abstract

**Background:**

Common diseases manifest differentially between patients, but the genetic origin of this variation remains unclear. To explore possible involvement of gene transcriptional-variation, we produce a DNA methylation-oriented, driver-gene-wide dataset of regulatory elements in human glioblastomas and study their effect on inter-patient gene expression variation.

**Results:**

In 175 of 177 analyzed gene regulatory domains, transcriptional enhancers and silencers are intermixed. Under experimental conditions, DNA methylation induces enhancers to alter their enhancing effects or convert into silencers, while silencers are affected inversely. High-resolution mapping of the association between DNA methylation and gene expression in intact genomes reveals methylation-related regulatory units (average size = 915.1 base-pairs). Upon increased methylation of these units, their target-genes either increased or decreased in expression. Gene-enhancing and silencing units constitute *cis*-regulatory networks of genes. Mathematical modeling of the networks highlights indicative methylation sites, which signified the effect of key regulatory units, and add up to make the overall transcriptional effect of the network. Methylation variation in these sites effectively describe inter-patient expression variation and, compared with DNA sequence-alterations, appears as a major contributor of gene-expression variation among glioblastoma patients.

**Conclusions:**

We describe complex *cis*-regulatory networks, which determine gene expression by summing the effects of positive and negative transcriptional inputs. In these networks, DNA methylation induces both enhancing and silencing effects, depending on the context. The revealed mechanism sheds light on the regulatory role of DNA methylation, explains inter-individual gene-expression variation, and opens the way for monitoring the driving forces behind deferential courses of cancer and other diseases.

**Supplementary Information:**

The online version contains supplementary material available at 10.1186/s13059-023-03094-6.

## Background

Many diseases display large between-patient heterogeneity in their time of onset, course of development, symptoms, severity, and treatment response. Understanding and control the genetic and environmental factors that generate these differences are crucial for the development of personalized medicine and may largely improve diagnosis, prognosis, and treatment protocols. Variation in the regulatory sequences of disease genes were shown to be involved in the generation of inter-patient, gene-expression variation [1–7], but sequence mutations alone were failed to explain the range of observed variations [8, 9]. Likewise, studies of regulatory epigenetic mutations revealed significant contributions to variation in gene expression, but descriptions of the full range of variation were seldom attained [10].

Whereas transcriptional enhancers were intensively studied [11], less is known about the mechanism of transcriptional silencing, its interaction with enhancers, and its possible effect on gene expression-variation. Transcriptional silencers are DNA sequences that upon binding of repressors or co-repressors reduce the transcription potential of linked promoters [12–17]. Interestingly, gene silencing is not the obligatory functioning of these loci. Rather, they may swap between silencing and enhancing effects in alternate cellular contexts [18–23]. Indeed, both enhancer and silencer loci were associated with variable chromatin states and bound activators and repressors, over alternate cellular conditions [24–30]. However, the molecular mechanisms that control the switching between silencer and enhancer functionalities were not defined. Moreover, while silencers and enhancers were showed to cooperate in the regulation of gene transcription [25], their participation in the producing of inter-individual expression variation remained unclear.

Here, we explored methylation-related variation in the mode-of-action and activity level of gene-associated silencers and enhancers, under controlled conditions as well as in intact cancer genomes. Based on this, we described an essential mechanism which drives differences in harmful gene activities among glioblastoma patients.

## Results

### High-resolution mapping of regulatory methylation sites

We applied a methylation-orientated strategy to explored gene-associated, *cis*-regulatory elements. Our design allows for interrogation of methylation sites with the potential to affect the regulation of targeted genes, at single-site resolution and precise methylation-level assessment. While the method is applicable to various genes and diseases, here, we focused on 125 pan-cancer or glioblastoma (GBM) driver genes and 52 reference genes (Additional file 1: Table S1). We performed the analysis in windows of two million base-pairs (bp) centered at the promoters of the genes, thus ensuring equal assessments of the studied genes, whether or not the boundaries of their topologically associated domains are known (Additional file 2: Fig. S1). Within these windows, we located chromatin regions that carry the general marker of distal regulatory elements, histone 3 mono-methylated lysine 4 (H3K4me1). To focus on inter-patient regulatory variation, we have further located, within the H3K4me1-marked chromatin, regions in which the activity marker histone 3 acetylated lysine 27 (H3K27ac) present in some, but not all, of the subjected glioblastoma tumors (Methods). An initial analysis confirmed that H3K4me1 indeed marks sites that display both negative and positive associations of DNA methylation with gene expression, in various cancer types (Additional file 2: Fig. S2). Analysis of DNaseI-hypersensitivity further confirmed the general regulatory potential of the selected chromatin regions (Additional file 1: Table S2).

**Fig. 1:**
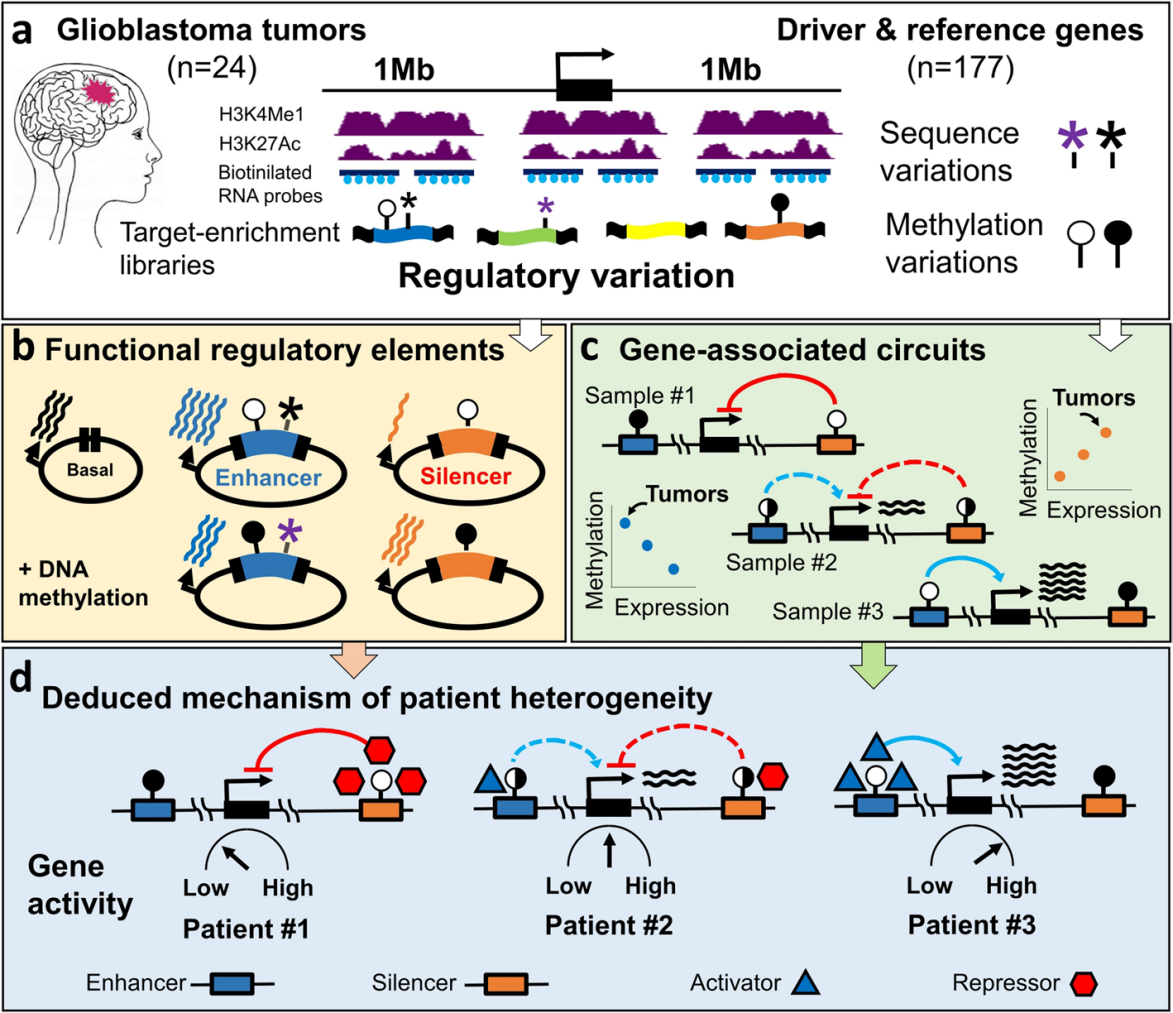
Methylation-oriented interrogation of regulatory gene-domains. **a** Library construction: Regulatory chromatin blocks were identified among glioblastoma (GBM) tumors in two megabases windows surrounding 125 driver and 52 reference cancer genes. The fractions of tumors carrying the general marker of regulatory chromatin H3K4Me1, or the marker of active chromatin H3K27ac, are schematically outlined in purple. Following random segmentation of tumor DNAs, the segments encompassing CpG methylation sites were captured using biotinylated RNA probes (blue dots). The obtained target-enriched libraries, representing the spectrum of methylation and sequence variations of the targeted regions, were used for following stages. **b** Experimental assessments of regulatory functions: a representative tumor library were cloned into gene-reporter vectors, downstream to minimal promoters, and assessed for enhancing or silencing of the vector’s basal transcription level, before or after DNA methylation. **c** Mapping of gene regulatory circuits: Methylation levels of the captured sites were assessed, and associated with expression levels of the studied genes, across the tumors. Schematic positive and negative circuits of a representative gene are shown. **d** Integration of the regulatory principles learned from the experimental assay, with actual gene-regulatory data, allows disclosing of *cis*-regulatory networks which govern inter-patient heterogeneity in the expression level of cancer driver genes

We then targeted the CpG methylation sites within these regions (*n* = 140,494), using a gene-enrichment assay with custom-designed targeting probes (size = 120 bp, *n* = 38,050) (Additional file 1: Tables S3 and S4). By applying the probes to tumors of GBM patients presenting the typical ranges of ages, genders, and subtypes characteristic of the disease (Additional file 1: Table S5), we obtained libraries of captured DNA segments (median size = 224 bp) (Additional file 1: Table S6). These captured segments carried a diversity of sequence and methylation variation presenting in the tumors. We assessed the regulatory potential of the captured segments, and the effects of sequence and methylation alternations, under experimental and native conditions (Fig. 1, Additional file 2: Fig. S3).

### Functional annotation of isolated regulatory segments

**Fig. 2:**
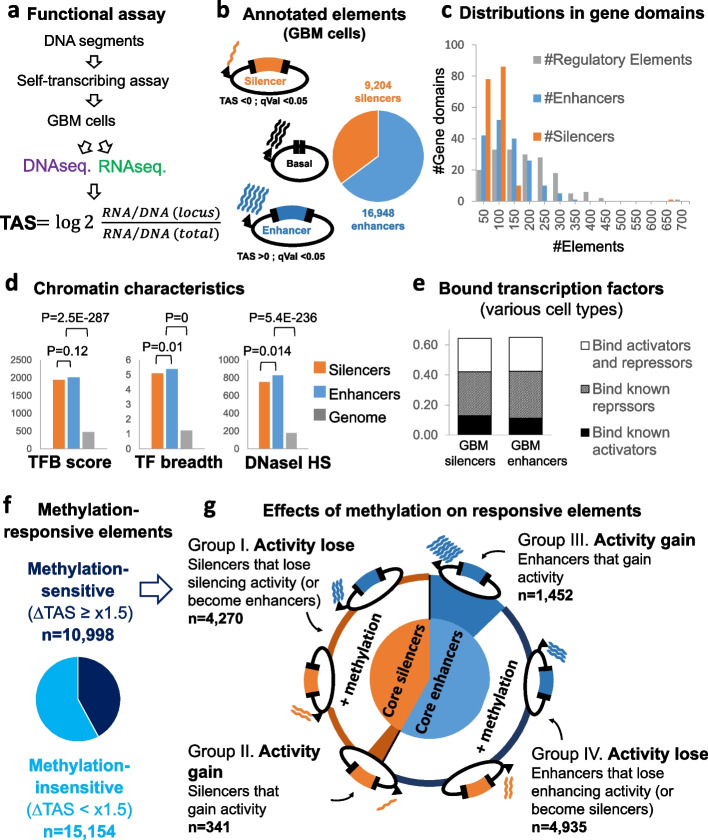
DNA methylation modify the transcriptional effect of enhancers and silencers. **a** Method: Putative regulatory DNA segments were captured from GBM tumors and allowed to drive self-transcription in T98G GBM cells, following complete de-methylation or in vitro re-methylation of the expression vectors. Local DNA to RNA ratios, relative to the total DNA to RNA ratio, denote transcriptional activity score (TAS) of the evaluated DNA segments. **b** Fractions of silencers (TAS < 0) or enhancers (TAS > 0) along the studied gene domains. **c** Distributions of regulatory elements, or of the elements that were annotated as silencers or enhancers, along the studied gene domains. **d** Regulatory chromatin characteristics of enhancer and silencer loci, across a variety of different cell types (ENCOD data). Level of transcription factors binding (TFB), factor variety (breadth), and DNase I hyper-sensitivity are shown. **e** Fractions of regulatory elements that are functioning as silencers or enhancers in GBM cells, which bind transcriptional repressors, activators, or both across a variety of different cell types (ENCOD data). **f** Fractions of regulatory elements which altered their functions upon DNA methylation. **g** Patterns of methylation effects on methylation-sensitive silencers and enhancers

Initially, we sought to understand the core functionalities of the captured DNA segments, without the effects of chromatin factors and interactions with other regulatory elements. For this experiment, we arbitrarily selected a representative library of captured DNA segments (library #100, Additional file 2: Fig. S3). Following stripping of chromatin layers and methylation marks, the entire set of captured DNA segments were cloned into gene-reporter vectors, downstream to minimal promoters (Fig. 1b). The obtained expression vectors were inserted into GBM cells and allowed to produce RNAs. The transcriptional effect of each segment was then examined, using a massively paralleled self-transcribing assay, adapted for detection of silencers and enhancers (Methods). For segments in windows of 500 bp, we calculated a transcriptional activity score (TAS), representing the level of produced RNAs, relative to the copy numbers of vector DNAs, and to the overall RNA to DNA ratio (Fig. 2a and Additional file 2: Fig. S4). Of 42,182 examined DNA segments, 26,152 revealed significant (*q* < 0.05) effect on transcription; of them, 9204 silence the basal expression level of the vectors, and 16,948 enhance (Fig. 2b, Additional file 1: Table S7). Additional 16,030 segments with non-significant effects were excluded from latter analyses. In most (176 of 177) of the analyzed gene domains, we observed multiple (11–693) functional elements (Fig. 2c). Of these, 175 domains contained both enhancers and silencers. Noticeably, annotated silencers and enhancers shared the characteristics of general regulatory chromatin and bound both activators and repressors in various cell types (Fig. 2d, e), suggesting that GBM silencers and enhancers may adapt different functionalities across cell types. We concluded that along gene regulatory domains, regulatory elements with core-GBM enhancer and silencer functionalities are similarly distributed.

### DNA methylation modifies the core functionalities of regulatory elements

We next compared the functionalities of the captured segments under whole-methylation versus de-methylation conditions. Of the 26,152 annotated segments, 10,998 displayed TAS differences of at least one and a half fold between methylated and un-methylated states (Fig. 2f, Additional file 2: Fig. S5a, Additional file 1: Table S7). The remaining 15,154 sites were not classified and may be insensitive to methylation or affected below the detection threshold of the assay. Both methylation-sensitive and methylation-insensitive sites display the characteristics of general regulatory chromatin across cell types (Additional file 2: Fig. S5b), suggesting that a more specific mechanism (e.g., cell-type-specific binding of methylation-sensitive transcription factors) underlays their differential response to methylation.

Of the methylation-sensitive elements, the majority (83.7%) reduced their original activity, or shifted to the opposite functionality (i.e., enhancers became weaker or turned silencers, and vice versa), upon methylation (groups I and IV in Fig. 2g, Additional file 2: Fig. S5c, Additional file 1: Table S7). The rest 16.3% increased their activity upon DNA methylation (groups II and III Fig. 2g). We concluded that under controlled conditions, DNA methylation retunes the activity level and induces functionally switching of many regulatory elements.

### Mapping methylation-related regulatory circuits in intact GBM genomes

**Fig. 3:**
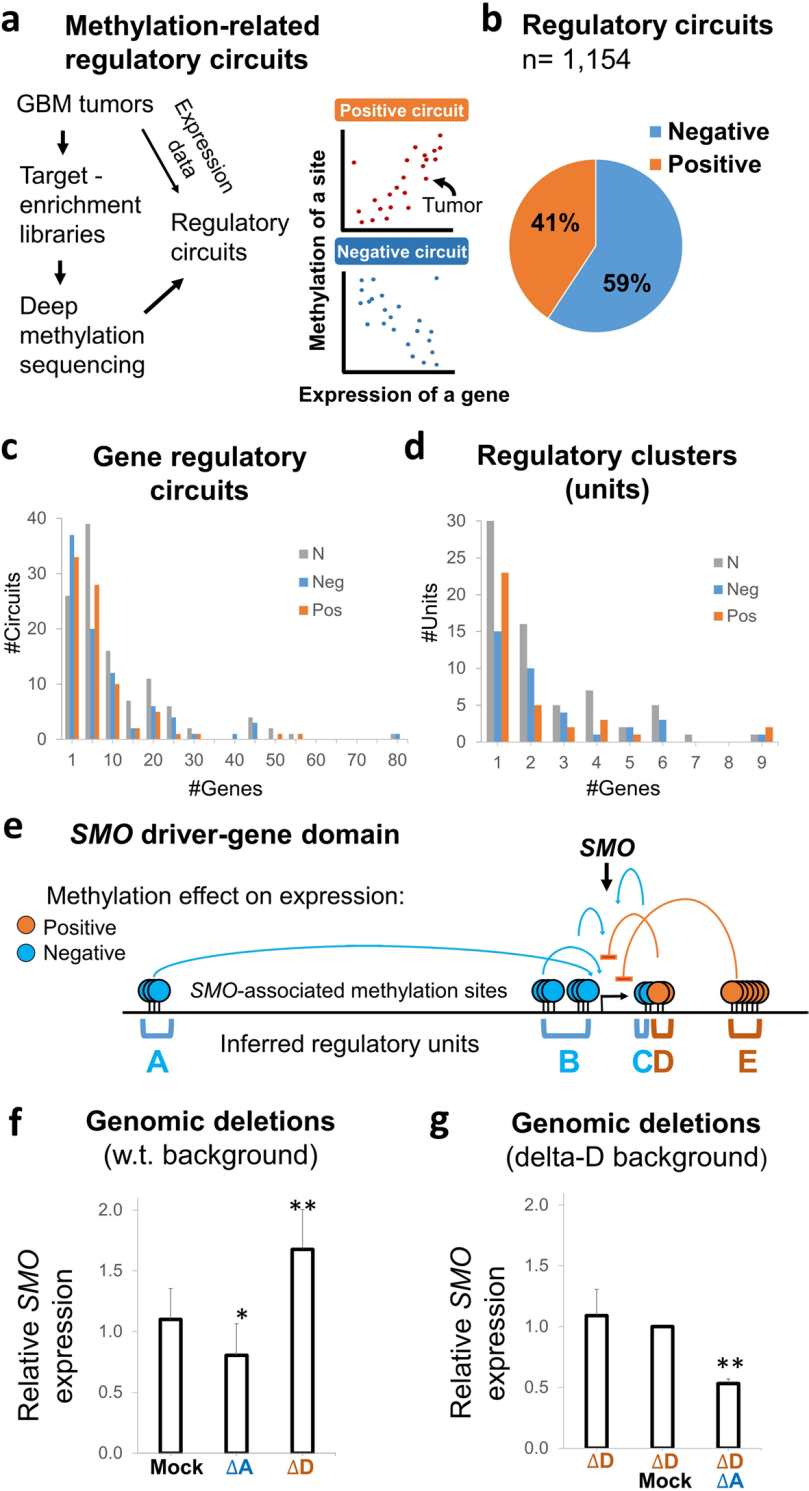
Mapping of *cis*-regulatory circuits, units, and networks in tumor chromatin. **a** Method: Each of the sequenced methylation sites was assessed for association with the expression of the targeted genes across the tumor libraries. Theoretical positive and negative regulatory circuits are shown. **b** Fractions of the mapped positive or negative regulatory circuits. **c** Distributions of all, negative, or positive circuits per genes. **d** Distributions of all, negative, or positive units per gene. **e** A schematic map of regulatory sites and units that were associated with expression of the SMO driver-gene. **f** levels of SMO expression in GBM cells, following genomic deletion of unit “A” (an inferred enhancer) or of unit “D” (an inferred silencer), versus mock genomic targeting by scrambled targeting guides. **g** Levels of SMO expression in GBM cells lacking unit “D,” following genomic deletion of unit “A,” or mock targeting of unit “A,’ versus untreated cells

While the above experiments revealed the principles of methylation effects on enhancers and silencers, actual chromatin and methylation conditions are essentially differing from the experimental assays. We next studied the relationships between DNA methylation and gene expression variation in intact GBM genomes, applying an established method to locate regulatory methylation sites of particular genes [31, 32] (Figs. 1c, and 3a). Utilizing deep methylation-sequencing data of 24 capturing libraries (Additional file 1: Table S1), we analyzed the association between methylation levels of the captured sites and expression levels of the targeted genes, across GBM tumors. These interactions were evaluated for each of the measured methylation sites, versus any one of the genes, within the 2 MB intervals. To avoid possible indirect effects, gene-body and promoter sites, which may display methylation-expression associations due to secondary interactions, were excluded from the analysis (*n* = 232, Additional file 2: Fig. S6). The analysis revealed *cis*-regulatory circuits (*n* = 1154; *q* < 0.05; *R*^2^ > 0.3) between certain methylation sites and controlled genes (Fig. 3b, Additional file 1: Table S8). Most (78%) of the genes had multiple (2–68) circuits, averaging 8.3 circuits per gene; of them, 3.5 circuits in average were positive (expression raised with methylation) and 4.8 negative (Fig. 3c, Additional file 1: Table S9).

### Matching between experimental and genomic analyses

We compared the maps of regulatory sites that have been obtained under experimental or natural conditions. Of the 26,152 functional elements identified by the reporter assay, 15,304 (58.5%) were matched with a gene-associated methylation site, located up to 500 bp of the elements (Additional file 2: Fig. S7a). The non-matched elements may be regulatory elements that are non-functional in GBM chromatin or reflect the limitations of the experimental assay. To discern between the possibilities, we analyzed whether gene-associated sites matched with functional segment. Indeed, 95.7% of the 1154 gene-associated methylation sites matched with a nearby element found by the experimental assay (Additional file 2: Fig. S7b), suggesting that GBM regulatory sites were effectively detected by the experimental assay. TAS analyses of the gene-associated sites revealed patterns of methylation effects similar to the patterns found in the experimentally defined elements (Additional file 2: Fig. S7c). We concluded that in spite of the essential differences between the simplified and actual conditions, the basic roles deduced from the experimental assay are relevant to GBM genomes.

### Clusters of gene-associated sites form *cis*-regulatory units

We explored the organization of gene-associated sites along gene domains. The analysis revealed clusters (average size = 915 bp, average number of methylation sites = 4.9), spreading across the gene domains (Additional file 1: Table S10). In each cluster, all methylation sites display the same (positive or negative) effect on the expression of an associated gene. We termed these clusters methylation-related regulatory units. Most (55%) of the genes were associated with multiple (2–9) units. The average number of units per gene was 2.5, of them 1.1 units in average mediate positive effect on expression and 1.4 negative (Fig. 3d, Additional file 1: Table S10).

### Gene expression level is derived from the sum effects of positive and negative units

We explored the interactions between the regulatory units of a gene. As a case study, we analyzed unit interaction in the smoothened, frizzled class receptor (SMO) driver-gene. Expression variation of this gene was associated with methylation variations in three negative units and two positive units (Fig. 3e). TAS analyses of these units suggested that the negative units served as enhancers in GBM cells, and the positive units served as silencers (Additional file 2: Fig. S8a). Indeed, deletion of unit “A” (an inferred enhancer) from GBM cells reduced the expression level of *SMO* relative to mock-treated cells, whereas deletion of unit “D” (an inferred silencer) gained its expression (Fig. 3f and Additional file 2: Fig. S8b-d). A control deletion of a neutral (non-regulatory) segment located between the units yielded no effect (Additional file 2: Fig. S9). We then analyzed the effect of co-deletions. Markedly, the enhancer unit “A” maintained its effect (30–50% of SMO expression), regardless of the silencer (Fig. 3g, Additional file 2: Fig. S8). Hence, enhancer and silencer units provided independent, additive effects on expression*.*

**Fig. 4:**
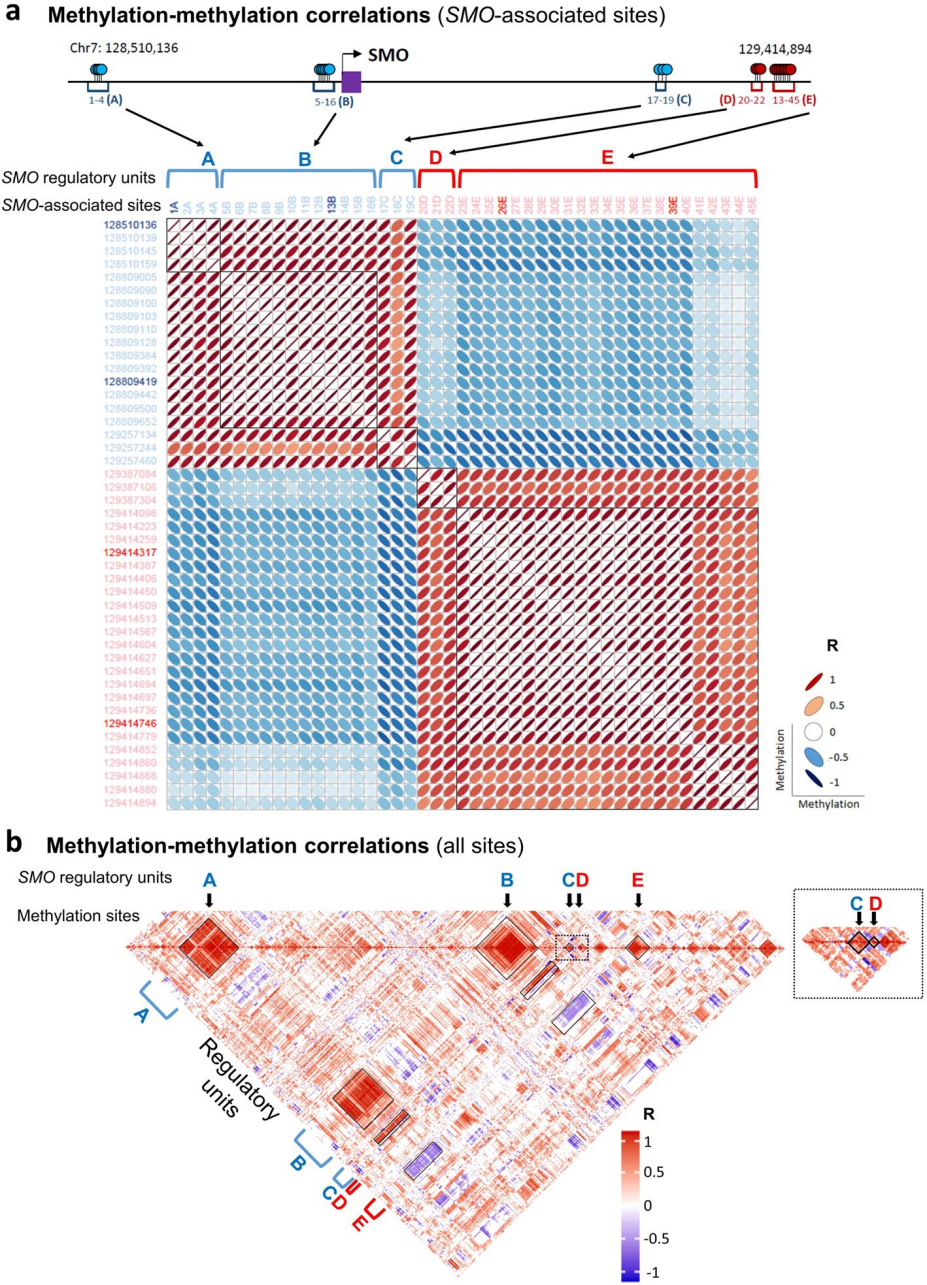
Between-unit interactions. **a** Top: Map of the methylation sites (listed 1–45) and the five regulatory units (listed **A**–**E**) that associated with SMO expression variation. Blue: negative associations, red: positive associations. Bottom: Matrix of the correlations between the methylation levels of SMO-associated sites. Each square in the matrix shows the methylation-versus-methylation correlation (R) between two of the associated sites. Genomic coordinates of the sites are indicated to the left. Bold coordinates indicate the sites that consist the SMO expression model (see Fig. 6). The analyses of the other studied genes are given in Additional file 3. **b** Correlations within the entire list of methylation sites that assessed in the SMO domain. The sites and units that presented in **a** are indicated

**Fig. 5:**
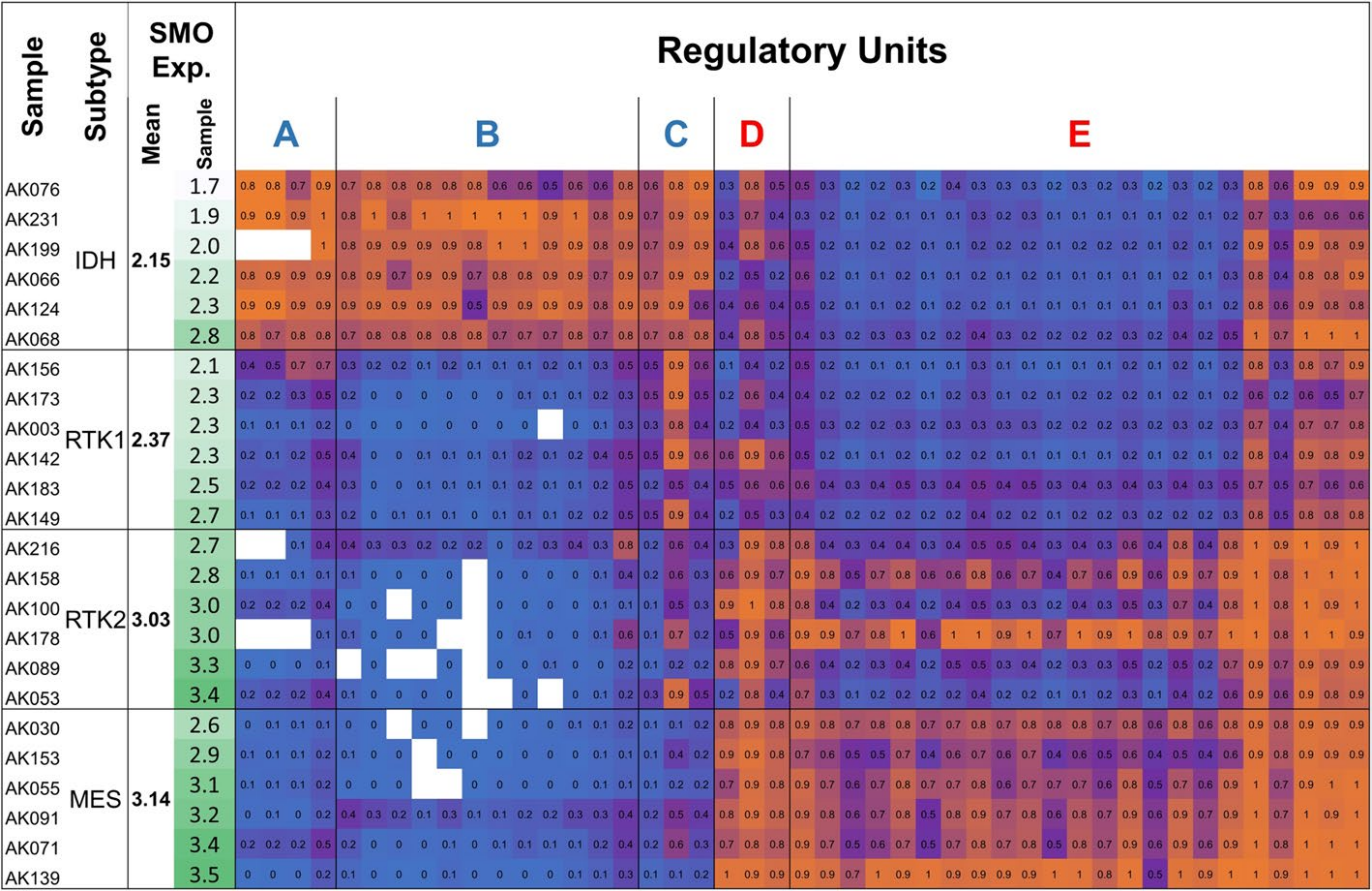
SMO expression level versus the methylation levels of *SMO*-associated sites and units. Tumor samples and subtypes are as described in Additional file 1: Table S5. Associated sites and units are as described in Fig. 4a. White cells signify unavailable data

We further study between-unit interactions on the large scale, by analyzing coordination of methylation levels across gene domains. As expected, direct correlations were observed among the methylation sites of given units, thus revealing their coordinated effects on gene expression (Fig. 4, Additional file 2: Fig. S10a, Additional file 3). We then analyzed the interactions between different units. Units with same (positive or negative) effects, i.e., units A, B, and C or units D and E of the *SMO* gene, were tended to show direct correlations. However, positive and negative units were reversely correlated (Fig. 4, Additional file 2: Fig. S10a, Additional file 3). To better understand the biological meaning of these interactions, we analyzed their effect on gene expression. Clearly, tumors with unmethylated silencers and methylated enhancers display the lowest levels of gene expression, tumors with methylated silencers and unmethylated enhancers displayed the highest, and mid-methylated enhancers and silencers associated with intermediate expression. These trends observed between GBM subtypes, which differentially expressed the genes, and to some extent also within the subtypes (Fig. 5, Additional file 2: Fig. S10b).

Taken together, the genetic-manipulation experiments and the analyses of intact tumors suggest that the mapped regulatory units provide complement, positive, and negative effects on expression. The sum of these effects describes the observed level of gene expression. We termed these cooperating regulatory units the methylation-related, *cis*-regulatory networks of genes.

### Genes in overlapping regulatory domains have independent regulatory networks

We further analyzed the relationships between networks of neighboring genes. Interestingly, units of given genes maintain their inter-network coordination, while showing no interactions with the activity of the other gene’s units within same regulatory domains (Additional file 2: Fig. S11, Additional file 4). Thus, genes with shared regulatory domains may collect their own transcriptional inputs from units that spread over the entire domain. These structures of spatially intermixed, gene-specific networks allow to neighboring gens to maintain independent expression profiles.

### Key methylation sites signify the overall effect of *cis*-regulatory networks

**Fig. 6:**
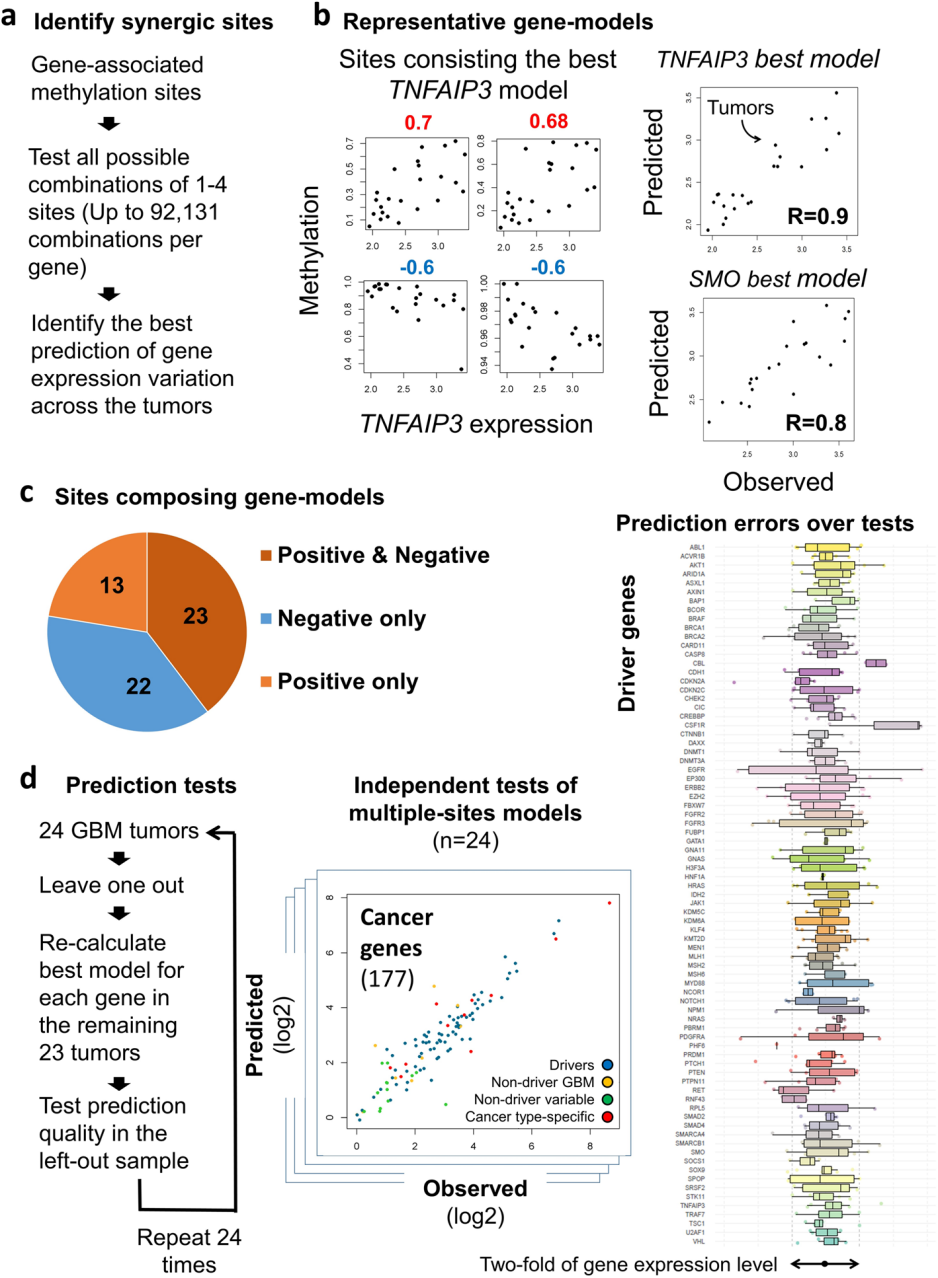
Key methylation sites describe inter-patient expression variation. **a** Development of methylation-based models of gene expression variation. **b** Example models of gene expression variations. Left: Methylation versus expression of the sites consisting the best model of the *TNFAIP3* gene. Right: Predicted versus observed expression levels of the *TNFAIP3* or the *SMO* genes across the tumors. *SMO* model was based on the four sites shown in Fig. S8a. **c** Fractions of gene-expression models which incorporate methylation data from, positive, negative, or both positive and negative units. **d** Assessing the prediction power of gene-expression models. Left: Developing of a prediction model for each of the 24 GBM tumors, based on the other 23 tumors. Middle: Example distribution of predicted versus observed expression levels of cancer genes. The full list of distributions is given in Additional file 5. Right: Distributions of predicted versus observed expression levels of driver genes across the 24 tumors. Log 2 of the differences between predicted and observed gene expression levels for driver genes with developed models are shown. Box plots describe the distributions of prediction errors in 24 independent tests

To gain insight to the mechanism of methylation-related networks, we examined the contribution of particular networked sites to the overall effect of the network: given a certain effect of a regulatory site on expression of an associated gene, we asked which other sites may improve the description of the gene expression variation across the tumors. Hence, redundant regulatory sites should provide no improvement, whereas antagonists or synergistic sites are expected to improve the descriptions provided by each of the sites alone. For this analysis, we focused on the major groups of enhancers and silencers (groups I and IV in Fig. 2g), by omitting 254 (22%) of the circuits, which according to the reporter assays may not belong to these groups. Using stepwise analyses, we identified the best models out of the possible combinations of one to four sites (Fig. 6a). For example, the gene *TNFAIP3* was associated with eighteen methylation sites. When individually assessed the methylation variations of these sites described the expression variation of *TNFAIP3* with *R*-values ranging between − 0.72 and 0.71 (Additional file 1: Table S9). However, a specific combination of four of these sites, out of 4029 possible combinations of one to four sites, provides *R* = 0.9 at *p* = 1.41E − 06 (Fig. 6b). Similarly, the model of the *SMO* expression, out of 17,875 possible combinations, was based on sites with individual *R* values between − 0.64 and 0.75 (Additional file 2: Fig. S8a), which together provide *R* = 0.8 at *p* = 0.00027. Noticeably, these best models rely on a single methylation site per unit to represent the effect of the entire unit. However, both positive and negative units were required in order to described the expression variations of the genes.

Overall, significant models of inter-patient’s expression variation were developed for 81 of the genes (58 drivers and 23 reference genes). Out of these, the expression of 58 genes (39 drivers and 19 reference genes) were best described by synergic combinations of sites that together provide better description than each of the sites alone. Of these synergic models, 23 used sites from both negative and positive sites (Fig. 6c, Additional file 1: Table S9). Note that the models were free to employ any associated site. Therefore, the fact that sites from both positive and negative units were often selected further highlighted the complementary role of positive and negative effects on gene expression.

We further controlled for possible bias due to the limit of up to four associated sites in the models, by re-developing the models using a least absolute shrinkage and selection operator (LASSO) approach [33], in which no limitation on the number of participating sites was applied. This independent analysis yielded very similar results, with an average of 3.8 contributing sites per gene-model across all genes. We concluded that methylation variation at relatively small number of indicative methylation sites may effectively describe gene expression variation.

### Prediction quality

Aside from understanding the mechanism of expression variation, we also sought to evaluate the possible usefulness of methylation-based models in predicting gene expression levels, e.g., when RNA biopsies are not available. Hence, we tested the ability of the developed models to predict gene expression levels in GBM tumors which was not assessed during model development. For each of the genes, we repeated the model-development procedure 24 times, each time using another combination of 23 samples, and analyzed the prediction errors in the left-out samples (Fig. 6d, Additional file 2: Fig. S12-13, Additional file 5). Then, we tested the ability to predict gene-expression alterations by identifying the cases (*n* = 868) in which the left-out tumor displayed irregular expression level (> 1SD) of a targeted gene. In 646 (74.4%) of these abnormalities, the models accurately predicted the expression level of the gene (prediction error within twofold of gene expression-level) (the “Methods” section, Additional file 1: Table S11), suggesting that further development of methylation-based models may be of practical value.

### Relative effects of methylation and sequence alternations

Finally, we compared relative contributions of sequence and methylation alternations to gene variation. In nearly half (45.8%) of the tumors, fewer than five driver genes were affected by nonsynonymous or copy number mutations (Table 1), in line with previous analyses of this cancer [34, 35]. This is in spite to the minimum of five to eight mutated driver genes that was suggested as the smallest number of mutations that enables the development of a tumor, suggesting that coding-mutation alone cannot account to many GBM tumors [36]. Further deep sequencing of silencer and enhancer sites in eight of the tumors (Additional file 1: Table S5) revealed only one regulatory sequence mutation that may possibly affect expression (Additional file 2: Supplemental Note S1). In contrast, all tumors included more than nine cancer-driving genes, which were abnormally expressed in the tumors compared with normal brains, and their inter-patient expression variation explained by methylation variation (Table 1). In many of these transformed driver genes, alternate methylation was the only type of mutation that associated with their malfunction (Table 2). These abnormally expressed, methylation-related genes involved in a range of cancer-initiation and progression processes, which may support the development of the previously unexplained tumors (Additional file 2: Fig. S14).

**Table 1:** Mutated driver genes per tumor

**GBM sample**		66	68	76	124	199	231	30	55	71	91	139	153	3	142	149	156	173	183	53	89	100	158	178	216
**GBM subtype**		**IDH**						**MES**						**RTK1**						**RTK2**					
**Coding mutation**	**SNV**^**a**^	4	na	**5**	4	**8**	**7**	na	na	3	**6**	3	4	na	**5**	2	3	4	2	na	2	4	**6**	2	**6**
	**CNV**^**b**^	-	na	-	-	2	-	na	na	1	1	-	-	3	1	2	-	-	-	1	1	1	1	1	-
**Regulatory mutations**	**SNV**^**c**^	na	na	na	-	-	na	na	na	-	na	na		na	na	-	1	na	na	na	-	-	na	na	na
	**CNV**^**d**^	na	na	na	-	-	na	na	na	-	na	na		na	na	-	-	na	na	na	-	-	na	na	na
	**Meth**^**e**^	**22**	**25**	**19**	**28**	**22**	**17**	**20**	**17**	**21**	**22**	**22**	**24**	**20**	**25**	**19**	**28**	**17**	**21**	**26**	**26**	**27**	**15**	**21**	**23**
	**Meth. only**^**f**^	**20**	**25**	**19**	**27**	**17**	**16**	**20**	**17**	**20**	**19**	**22**	**23**	**19**	**24**	**18**	**27**	**17**	**21**	**25**	**23**	**26**	**13**	**19**	**23**

Tumors with 5 or more mutations are in bold

*na* data not available

*-*, not detected

^a^Coding-sequence alternation

^b^Coding-copy-number alternations

^c^Regulatory-sequence alternation

^d^Regulatory-copy-number alternations

^e^Numbers of abnormally expressed (twofold or more expression differences from normal brain samples) driver genes for which methylation alternations describe their expression-variation

^f^Numbers of abnormally expressed driver genes for which methylation alternations were the only type of mutation

**Table 2:** Mutations of abnormally expressed driver genes

Mutation type	Driver gene	Fraction (%) of tumors with coding mutations	Fraction (%) of tumors with abnormal expression^a^	Expression variation explained^b^	Methylation-expression associations
**Regulatory**	FBXW7	0	100	Yes	Negative
	SMO	0	95.8	Yes	Both
	SOX9	0	79.2	Yes	Positive
	FGFR2	0	79.2	Yes	Negative
	CASP8	0	70.8	Yes	Positive
	TNFAIP3	0	70.8	Yes	Both
	AR	0	70.8	Yes	Negative
	CHEK2	0	66.7	Yes	Negative
	H3F3A	0	54.2	Yes	Both
	ABL1	0	45.8	Yes	Both
	DAXX	0	29.2	Yes	Both
	MSH6	0	29.2	Yes	MSH6
	ZIC2	0	12.5	Yes	Negative
	JAK1	0	8.3	Yes	Both
	U2AF1	0	8.3	Yes	Positive
	CTNNB1	0	8.3	Yes	Negative
	MLH1	0	8.3	Yes	Negative
	SOCS1	0	4.2	Yes	Both
	SRSF2	0	4.2	Yes	Both
	SMAD2	0	4.2	Yes	Negative
	VHL	0	4.2	Yes	Negative
**Regulatory and coding**	CDKN2C	5.3	100	Yes	Negative
	BRCA1	21.1	83.3	Yes	Both
	TRAF7	5.3	41.7	Yes	Positive
	AKT1	5.3	20.8	Yes	Positive
	PBRM1	5.3	12.5	Yes	Both
	MSH2	10.5	8.3	Yes	Both
	FUBP1	5.3	8.3	Yes	Negative
	MEN1	5.3	4.2	Yes	Both
	CREBBP	10.5	4.2	Yes	Positive
	PRDM1	10.5	0.8	Yes	Both
**Coding**	TP53	47.0	100	No	-

^a^Two-fold or more expression differences from normal brain samples

^b^Have a verified methylation-based model of expression variation

## Discussion

Whereas the general structure of gene regulatory domains was comprehensively described [37], their internal organization are less understood. Particularly, the operational sites and units within these domains, the interaction between them, and the genetic and epigenetic mechanisms that organize their effects on genes were not well mapped and explained. Due to this lack of knowledge, the origin of variable expression levels of disease genes remained unclear. Here, we presented a practical way to decipher the internal structure of large regulatory domains, by mapping and annotation of *cis*-regulatory methylation schemes. Utilizing this approach, we revealed a main source of gene variation among glioblastoma patients. Furthermore, our study shed light on the mechanism of gene regulation and explains some long-standing wonderings regarding the effect of epigenetic mutations.

Our results suggest that DNA methylation either directly affects, or is a very close bystander, of the binding profiles of transcriptional activators and repressors to regulatory DNA sites. In turn, the binding of these factors determined the transcriptional activity of genes, which involve in cancer initiation and progression pathways (Additional file 2: Fig. S14). We further showed that the methylation profiles at key regulatory sites efficiently predict the variation in the activity level of these genes among GBM patients (Fig. 6). While the experimental assay supports a direct, causative role for methylation in the setting of these expression profiles (Fig. 2), we cannot exclude a more complex mechanism in actual chromatin.

We initially choose to focus on DNA methylation due to its unique efficacy as a sensitive and quantitative indicator of *cis*-regulatory activity [31, 38]. However, the regulatory effect of DNA methylation was unclear: whereas methylation of gene promoters was almost exclusively associated with transcriptional repression [39], positive and negative effects on gene expression were described in non-promoter sites [40–42]. The reason for this duality was not explained. Moreover, while evidence for direct effects of DNA methylation on transcriptional enhancers has been presented [43, 44], its effect on silencers and on the interaction between silencers and enhancers remains unknown. Due to these inconsistencies, interpretation of methylation data was highly complex. We showed that this complexity may be deciphered by considering specific effects of methylation in particular regulatory contexts.

A key feature of our design is the ability to co-explore enhancers and silencers. This was achieved by adaption of a commonly used reporter assay [45], so that its intrinsic bias toward enhancers [24] was removed. Utilizing this assay, we showed that under experimental conditions, DNA methylation can enforce regulatory elements to switch their mode-of-action between enhancer and silencer functionalities (Fig. 2).

It was previously shown that silencers are relatively enriched by sequence binding motifs of transcriptional repressors [17]. Our analysis showed a weak tendency of the positive sites towered repressor binding motifs, including EZH2 and REST motifs; however, both positive and negative sites encompass binding motifs of repressors and activators, and no significant difference was revealed. Moreover, positive and negative sites bind both activators and repressors, over a range of tissues and cell types (Fig. 2e). These findings are in line with the previously shown ability of regulatory sites to bind activators and repressors over alternate cellular conditions [24–30] and to swap between silencing and enhancing effects in alternate cellular contexts [18–23]. The mechanism of transition between silencing and enhancing effects of regulatory sequences is not well understood. It was recently observed that regulatory elements that switch functionality during T cell development contain binding sites of opposing functions within same region, suggesting the use of different transcription factors for establishing activating and repressive functions, but the mechanism that govern this transition was not defined [23]. We showed that regulatory sites may swap between enhancing and silencing functioning even within a given cell type, when no developmental process occurred. Therefore, alternation in the expression of activators and repressors may not be necessary for functional switching. Rather, we showed that DNA methylation is both required and sufficient to induce functionality switching, at least under experimental conditions. Moreover, methylation not only directs functionality switching but may also tune the degree of regulatory effects, ranging from low to high gene enhancing or silencing (Fig. 2g and Additional file 2: Fig. S5c). Whether these features defined a unique class of methylation-depended regulatory sites, or also applies to other sites, remained to be determined.

Our results also hint at the mechanism of methylation-mediated switching and retuning of regulatory elements. First, the finding that many enhancers and silencers loose or reduce their activity upon methylation (Fig. S5c) suggests a similarity with promoter methylation, in which DNA methylation eliminates the binding of transcriptional activators [39]. However, we also showed that in non-promoter regulatory elements, methylation can decrease or increase regulatory activities. Taken together with the former observations of dual-functional binding sequences in altering regulatory elements, these observations fit with methylation-mediated retuning of the balance between bound activators and repressors. Indeed, a wide repertoire of gene activators and repressors bind the mapped enhancer and silencer elements (Fig. 2e). The identity of the transcription factors that bind to the described sites upon acting as enhancers or silencers, and whether they binding affinities are methylation-dependent, remained to be studied. Considering that many activators and repressors are sensitive to methylation of their binding sites [46], this is a feasible scenario.

Our study applied both experimental and in vivo analyses to explore methylation-related regulatory structures. The experimental gene-reporter assay allowed for the evaluation of methylation effects under controlled conditions, while other influences were neutralized. This assay was apparently effective in the revealing of net methylation effects and was crucial for the detection of methylation roles in enhancers and silencers (Fig. 2). However, its simplified structure is essentially different from the actual tumor conditions and ignored the important effects of chromatin states, long-distance interactions, and partial methylation conditions. The profiles of expressed transcription factors may be also differing between the experimental assay and actual tumors. Therefore, the effect of methylation in particular regulatory sites should be learned in genuine tumors. Nevertheless, the experimental assay revealed general principles of methylation effects and facilitate their following detection in actual tumors. Specifically, the participation of multiple elements in the regulation of particular genes, the cooperative positive and negative effects on gene expressions that provided by these elements, and the involvement of DNA methylation with these opposing effects were first learned in the experimental assay and subsequently described in the tumors.

Based on these understandings, we gained insight to the organization of gene regulatory domains in actual tumors. We showed that regulatory domains comprise gene-specific networks, each of them spreading over large (typically hundreds of kilobases) genomic spans, which may overlap with networks of other genes. These *cis*-regulatory networks are composed of discrete, spatially separated regulatory units. Each of these units mediate a defined, silencing or enhancing effect on the expression of the target genes. The combined effects of the units describe the observed level of gene expression (Figs. 3 and 6). In line with the excess of functional enhancers (Fig. 2b), negative associations, both on the level of single sites and site units, were more abundant than positive, possibly due to an access of bound gene activators over gene repressors (Figs. 4 and 5, Additional file 1: Tables S9 and S10). Nevertheless, the expression models of many gene relay on both positive and negative associations (Fig. 6c), suggesting that gene regulation often required the both. Variation in the activity levels of the networked units, as indicated by their level of DNA methylation, conveys gene expression variation (Figs. 4 and 5 and Additional file 2: Fig. S8-S10). The typical span of the units, as appeared from methylation-based analyses, is several hundred bp (average size = 915.1 bp, Additional file 1: Table S10). Under controlled conditions, DNA methylation determines the mode and the level of the transcriptional effects carried by these units (Fig. 2). In actual tumors, it closely reports these effects, although its causative role in real chromatin conditions remained to be determined.

Our analyses further reveal how differential expression profiles are enabled for genes in shared regulatory domains. Apparently, gene-specific territories [47] are not essentially required. Rather, we showed that gene-specific networks may use overlapping genomic spans (Additional file 2: Fig. S11, Additional file 4). This structure may cause spatial interfering when units of overlapping networks converge with their target promoters, unless if the network convergences occur within short, differential time-windows. A recent analysis of DNA looping dynamics [48] support this view.

Our mapping approach provided unique coverage and resolution of the explored domains, which are not achievable by other methylation-mapping approaches including whole-genome bisulfite sequencing and commercial microarrays. Yet, some functional sites and units might be missed in our maps. Particularly, the filtering of possible gene-body [49, 50] or secondary effects (Additional file 2: Fig. S6) may abolish some actual sites. In addition, units with low number of methylation sites may escape our mapping. Additional limitations of our mapping criteria include the constant 2 Mbp windows that we applied to all genes and the specific chromatin regions that we targeted. Since TADs were not defined for about a third of our genes of interest, we choose to apply equal analysis-windows to all genes. This provided full coverage of > 80% of the mapped TADs while allowing unbiased analyses of the genes. However, we cannot eliminate the possibility that more relevant sites may reside outside of the analyzed domains. Within these domains, we focused on methylation sites within H3K4me1-marked and H3K27ac-variable chromatin blocks. As shown, these targeting criteria are relevant to a large bulk of glioblastoma silencers and enhancers. Nevertheless, additional regulatory elements may reside in areas that has not been covered.

Knowing the level of DNA methylation at key sites within the mapped units, in bona fide cancer tumors, was sufficient to obtain effective description of gene expression variations, across patients (Fig. 6). Moreover, DNA sequencing of these sites revealed no sequence alternations that may account for the observed variations (Additional file 2: Supplemental Note S1). In 20 of the genes, methylation variation alone is accountable for > 80% of the observed expression variations (*R* =  > 0.9, Additional file 1: Table S9), thus leaving little space for other genetic or environmental factors. Hence, methylation variation appeared as a prime indicator of inter-patient expression variation. Whether it may also explain the transformation of normal cells into cancer remains unknown. The observation of driver genes which are abnormally expressed in the cancer, and have models of expression variation but no coding or regulatory sequence mutations (at least in their known *cis*-elements) (Table 2), may support this possibility. However, more research is needed to established a gene-transformation effect.

The general nature of our findings suggests relevance to other cancers and diseases besides glioblastoma; however, further study is needed to establish this possibility. Interestingly, same methylation sites can switch between positive and negative effects on gene expression, between cancers (Additional file 2: Fig. S2b). Hence, while the general structure of *cis*-regulatory networks may be conserved over cancers, their particular sites and units may be disease-specific.

The origin of inter-patient methylation variation remains unknown. Global and local epigenetic schemes, including DNA methylation blueprints, are often disrupted during the development of cancers [51–58]. Several mechanisms may cause methylation alternations and may account for the methylation-alternation that observed in this study. These include loss or gain of methylation or demethylation factors, errors in the replication of methylation signals during cell divisions, and remote effects of sequence alternations [59]. However, elucidation the cause of particular alternations during the development of the tumors required additional research.

The role of methylation alternation in gene regulation and inter-patient variation was not fully explained. Our findings call for a new evaluation of these roles. It was sometimes deduced that the overall effect of methylation is limited, as global de-methylation of the genome yields little effect on the expression of the most of the genes [60, 61]. Re-analyses of global de-methylation experiments, in light of our findings, may reveal that these seemingly insensitive genes are actually protected from the otherwise harsh effect of global methylation failures, by balanced numbers of positive and negative sites in their *cis*-regulatory networks. Yet, they may be strongly affected by site-specific methylation alternations. Therefore, the number of genes that was controlled by DNA methylation may be significantly greater than already known.

## Conclusions

Utilizing high-coverage, high-resolution mapping of regulatory methylation sites, we shed light on the internal organizations of gene regulatory domains as well as on the regulatory role of DNA methylation. We found that *cis*-regulatory domains are composed of spatially overlapped, gene-specific regulatory networks. These networks comprised multiple regulatory units; each of them provides a define, positive, or negative effect on the expression of the targeted genes. Under control conditions, DNA methylation dictates the mode and the level of these effects. The sum effects of methylation variation in a small number of key methylation sites, located in positive and negative units, effectively describe the variation in the expression of cancer genes among glioblastoma patients. The revealed mechanism shed light on a long-standing enigma regarding the regulatory role of DNA methylation, explains inter-individual gene-expression variation, and flags particular methylation sites as candidates for RNA-free monitoring of inter-patient expression variation. Whether genetic or epigenetic editing of these sites will improve GBM phenotypes, and the relevance for other illnesses, remains to be determined.

## Methods

### Input materials

Input materials for target enrichment library were provided by the German Cancer Research Center (DKFZ), Heidelberg, Germany. Four main GBM subgroups, as defined based on sequence and methylation profiling [62], were included. Equivalent subgroup representation was given (Additional file 1: Table S5).

#### The Cancer Genome Atlas (TCGA)

Gene expression (RNAseqV2 normalized RSEM) and DNA methylation data (HumanMethylation450) were downloaded in May 2019 using TCGAbiolinks [63–65] for the following cancer types: BRCA (778 genomes), CESC, (304), COAD (306), ESCA (161), GBM (50), KICH (65), KIRC (320), KIRP (273), LIHC (371), LUAD (463), PAAD (177), SKCM (103), THYM (119).

#### NIH Roadmap Epigenomic Project [66] 

H3K4me1 broad peaks of corresponded TCGA tumor types and DNaseI cell-specific narrow peaks of normal brain (E081 and E082) were obtained.

#### Encyclopedia of DNA Elements (ENCODE) [67]

DNaseI hypersensitivity peak clusters (wgEncodeRegDnaseClusteredV3.bed.gz) and transcription factor ChIP-seq clusters (wgEncodeRegTfbsClusteredWithCellsV3.bed.gz) and DNase brain tumor data (Gliobla and SK-N-SH) were obtained. The ENCODE transcription factor binding (TFB) scores presented in Fig. 2 represent the peaks of transcription factor occupancy from uniform processing of ENCODE ChIP-seq data by the ENCODE Analysis Working Group. Scores were assigned to peaks by multiplying the input signal values by a normalization factor calculated as the ratio of the maximum score value (1000) to the signal value at one standard deviation from the mean, with values exceeding 1000 capped at 1000. Peaks for 161 transcription factors in 91 cell types are combined here into clusters to produce a summary display showing occupancy regions for each factor and motif sites within the regions when identified. One-letter code for the different cell lines is given in https://hgdownload.cse.ucsc.edu/goldenpath/hg19/encodeDCC/wgEncodeRegTfbsClustered/wgEncodeRegTfbsClusteredV3.bed.gz.

#### Additional public data

HiC Data for TADs were downloaded from https://wangftp.wustl.edu/hubs/johnston_gallo/ [68].

### Cell lines

Human GBM T98G cells were purchased from the ATCC collection (ATCC® CRL-1690™) and cultured in minimum essential medium-Eagle (Biological Industries), supplemented with 10% heat-inactivated FBS #04-127-1A (Biological Industries), 1% penicillin/streptomycin P/S # 03-031-1B (Biological Industries), 1% L-glutamine #03-020-1C (Biological Industries;), 1% non-essential amino acids, #01-340-1B (Biological Industries), and 1% sodium pyruvate #03-042-1B (Biological Industries), at 37 °C and 5% CO_2_.

### Genes

Genes analyzed in the study included the pan-cancer driver genes listed by Vogelstein et al. [36] including the GBM driver genes listed by Kandoth et al. [35], but excluding the HIST1, H3B, and CRLF2 genes due to missing expression data, and the AMER1 gene for which probe design failed. Cancer type-specific genes (*n* = 23) were selected from a published list of 840 genes [69]. Non-driver candidate GBM genes (*n* = 14) were suggested by BR. Non-driver variable genes (*n* = 22) were defined as those showing top expression variation among the 70 analyzed GBM samples for which we found at least two correlative sites in the TCGA-GBM dataset. We used the genomic coordinates for gene features from the hg19 refGene table of the UCSC Genome Browser [70]

### Target enrichment assays

Variable regulatory regions were defined as the regions carrying H3K4me1 marks in all tumors, and also H3K27ac in at least 25% of the tumors, but not in at least other 25% of the tumors. RNA probes were designed to target all methylation sites within these regions, utilizing the SureDesign tool (https://earray.chem.agilent.com/suredesign/). Probe duplication was applied in cases (*n* = 8652) of > 5 CpG sites within the 120 bp span of the probes. Repetitive regions were identified by BLAT [71] and excluded from the design. Custom-designed biotinylated RNA probes were ordered from Agilent Technologies (https://www.agilent.com).

Genomic tumor DNAs were arbitrarily sheared using a sonication device into collections of DNA fragments of various sizes. These DNA segments were then allowed to attach the probes which fully or partially overlapped their span. The resulting collection of captured DNA segments (median size = 224 bp) was integrated into gene-reporting vectors or underwent sequencing.

Enrichment libraries of GBM-targeted regulatory DNA segments were constructed using the SureSelect protocol #G9611A (Agilent) for Illumina multiplexed sequencing, which used 200 nanograms genomic DNA per reaction, or the SureSelect Methyl-Seq protocol #G9651A using 1 μg genomic DNA per reaction. Quality and size distribution of the captured genomic segments were verified by TapStation nucleic acids system (Agilent) assessments of regular or bisulfite-converted libraries. Target enrichment efficiency and coverage was evaluated via sequencing.

### Massively paralleled reporter assay

Massively parallel functional assays were performed as described by Arnold et al. [72], with the following modifications.

#### Reporter backbone

The pGL3-promoter vector (Promega, GenBank accession number U47298) was modified as described in Additional file 2: Fig. S15.

#### Genomic inputs

Plasmid libraries were constructed using a target-enriched library as input material: 1 μL of adaptor-ligated DNA fragments from the AK100 target enrichment library was amplified in eight independent PCR reactions, using KAPA Hifi Hot Start Ready Mix #KK2601 (KAPA Biosystems). Reaction conditions included 45 s (s) at 95 °C, 10 cycles of 15 s at 98 °C, 30 s at 65 °C, 30 s at 72 °C, and 2 min final extension at 72 °C, applying the forward Illumina universal primer: 5′-TAGAGCATGCACCGGTAATGATACGGCGACCACCGAGATCT-3′ and reverse Indexed Illumina primer: 5′-GGCCGAATTCGTCGACCAAGCAGAAGACGGCATACGAGAT-3′, containing Illumina adapter sequences. A specific 15-nt extension was added to each adapter as homology arms for directional cloning. PCR reactions were pooled and purified on NucleoSpin Gel and PCR Clean-up #740609 columns (Macherey–Nagel). The screening vector was linearized with AgeI-HF and SalI-HF restriction enzymes (NEB) and purified through electrophoresis and gel extraction. Purified PCR products were cloned into the linearized vector by recombination with the adapter-ligated homology arms in 12 reactions of 10 μL each, applying the In-Fusion HD #639649 kit (Clontech). The reactions were then pooled and purified with 1x Agencourt AMPureXP DNA beads #A63881 (Beckman Coulter) and eluted in 24 μL nuclease-free water.

#### Library propagation

Aliquots (*n* = 12, 20 μL each) of MegaX DH10B T1 Electrocomp Bacteria #C640003 (Invitrogen) were transformed with 2 μL of the plasmid DNA library, according to the manufacturer’s protocol, except for the electroporation step, which was performed using the Nucleofactor 2b platform (Lonza) Bacteria program 2. Every three transformation reactions were pooled (total of 4 reactions) for a 1-h recovery at 37 °C, in SOC medium, while shaking at 225 rpm, after which each reaction was transferred to 500 ml LB AMP (Luria Broth Ampicillin) for overnight 37 °C incubation, while shaking at 225 rpm. Propagated plasmid libraries were extracted using the NucleoBond Xtra Maxi Plus Kit (#740416) (Macherey–Nagel). To verify unbiased amplification of the targeted genomic segments, the size distribution and coverage of the library were analyzed before and after the propagation step (Additional file 2: Fig. S16).

#### In vitro methylation assay

Complete de-methylation stages were achieved by propagation of the libraries in bacteria following PCR amplification stages. In vitro methylation of the de-methylated plasmid DNA was performed using the New England Biolabs CpG Methyltransferase M.SssI #M0226M according to the manufacturer’s instructions. Efficient methylation level was confirmed by using a DNA protection assay against FastDigest HpaII #FD0514 (Thermo Scientific) digestion (Additional file 2: Fig. S17).

#### Transfection to GBM cells

Twenty micrograms of DNA were transfected into 2 × 10^6^ T98G and U87 cells at 70–80% confluence, using the Lipofectamine 3000 transfection kit #L3000-015 (Invitrogen), according to the manufacturer’s protocol. In each experiment, 5 × 10^7^ cells were transfected and incubated at 37 °C, for 24 h.

#### Isolation of plasmid DNA and RNA from GBM cells

Plasmid DNA was extracted from 2.5 × 10^7^ cells, 24 h post-transfection. Cells were rinsed twice with PBS pH 7.4, using the NucleoSpin Plasmid EasyPure kit #740727250 (Macherey–Nagel), according to the manufacturer’s protocol. Total RNA was extracted from 2.5 × 10^7^ cells 24 h post-transfection using GENEZOL reagent # GZR200 (Geneaid), according to the manufacturer’s protocol. The polyA + RNA fraction was isolated using Dynabeads Oligo-(dT)25 #61002 (Thermo scientific), scaling up the manufacturer’s protocol 5-fold per tube, and treated with 10 U turboDNase #AM2238 (Invitrogen) at 20 ng/μL 37 °C, for 1 h. Two reactions of 50 μL each were pooled and subjected to RNeasy MinElute clean up kit #74204 reaction (Qiagen) to inactivate turbo DNase and concentrate the polyA + RNA.

#### Reverse transcription

First-strand cDNA synthesis was performed with 1–1.5 μg polyA + RNA in a total of four reactions, 20 μL each, using the Verso cDNA Synthesis Kit #AB1453B (Thermo Scientific) according to the manufacturer’s protocol, with a reporter-RNA specific primer (5′-CAAACTCATCAATGTATCTTATCATG-3′). cDNA (50 ng) was first amplified by PCR, at 98 °C for 3 min, followed by 15 cycles at 95 °C for 20 s each, 65 °C for 15 s, and 72 °C for 30 s. Final extension was performed at 72 °C for 2 min, using the Hifi Hot Start Ready Mix (KAPA), with reporter-specific primers. Forward primer: 5′-GGGCCAGCTGTTGGGGTG*T*C*C*A*C-3′ which spans the splice junction of the synthetic intron, and reverse primer: 5′-CTTATCATGTCTGCTCGA*A*G*C-3′, where “*” indicates phosphorothioate bonds. In total, 16–20 reactions were performed. The amplified products were purified with 0.8 × Agencourt AMPureXP DNA beads and eluted in 20 μL nuclease-free water. The resultant purified products served as a template for a second PCR performed under the following conditions: 95 °C for 3 min, 12 cycles of 98 °C for 15 s, 65 °C for 30 s, 72 °C for 30 s. Final extension was performed at 72 °C for 2 min, with forward Illumina universal primer: 5′-TAGAGCATGCACCGGTAATGATACGGCGACCACCGAGATCT-3′ and reverse indexed Illumina primer: 5′-GGCCGAATTCGTCGACCAAGCAGAAGACGGCATACGAGAT-3′. PCR products were purified with 0.8 x Agencourt AMPureXP DNA beads, eluted in 10 μL nuclease-free water, and pooled.

### Transcriptional activity analysis

Quality and size distribution of extracted plasmid DNAs and RNAs were verified using TapeStation (Additional file 2: Fig. S18). DNA and cDNA samples were sequenced using the HiSeq2500 device (Illumina), as per the 125-bp paired-end protocol. Alignment with the hg19 reference genome was performed on the first 40 bp from both sides of the DNA segments, using Bowtie2 [73]. Reads with mapping quality value above 40 aligned with the probe targets were considered for further analyses. Each of the captured genomic segments was given a unique ID according to genomic location and indicated the total number of DNA and RNA reads. Only on-target segments with at least one RNA read (*n* = 623,223 pre-methylation; 304,998 post-methylation) were included. > 99% of the targeted regions were presented following the propagation in bacteria and re-extraction from T98 cells (Additional file 1: Table S12). Technical and biological replications performed using Illumina MiSeq sequencing.

Transcriptional activity score (TAS) was calculated as follows:

$$\mathrm{TAS }= {\mathrm{log}}_{2}(({\mathrm{RNA}}_{j}/{\mathrm{DNA}}_{j})/({\mathrm{RNA}}_{\mathrm{total}}/{\mathrm{DNA}}_{\mathrm{total}})),$$

where *j* is a genomic element and RNA_total_ or DNA_total_ is the sum of all segment reads.

For the analyses of isolated regulatory elements, TAS was determined in 500 bp, 50% overlapping windows, across the genome, based on the DNA and RNA reads of segments overlapping with the given window. TAS significance was tested by chi-square against total RNA to DNA. Multiple comparisons were corrected. Functional regulatory elements were defined as elements with FDR *q* value < 0.05 and minimum 100 RNA reads, where positive TASs were defined as enhancers and negative as silencers. The methylation effect was analyzed by calculating TAS difference between treatments, where regulatory elements with a difference of ≥ 1.5-fold activity were counted.

### Inferring *cis*-regulatory circuits

#### Methylation sequencing

Methyl-seq-captured libraries were sequenced using a Hiseq2500 device (Illumina), by applying paired-end 125 bp reads. Sequence alignment and DNA methylation calling were performed using the Bismark V0.15.0 software [74] against the hg19 reference genome. The sequencing yielded 52–149 million reads per sample, at an average mapping efficiency of 78.1%, average bisulfite efficiency of 97.6%, and 99.4% on target average. Overall, a mean coverage of 916 reads per site was obtained, and 86% of the targeted sites were covered by at least 100 reads. Sites that appeared in less than eight of the tumors were excluded from the analyses.

#### Circuit annotation

Correlation between the expression level of each targeted gene and the DNA methylation level of targeted CpG sites in a 2-Mbp region flanking its transcription start site (TSS) was assessed by applying pairwise Spearman’s rank correlation coefficient with Benjamini–Hochberg correction for multiple-hypothesis testing at FDR < 5%. Circuits with *R*^2^ > 0.3 were included. Sites that correlated (*R*^2^ > 0.1) with expression of the PTPRC (CD45) pan-blood cells marker were considered a possible result of blood contamination and were eliminated from later analyses, as described [75]. Potential secondary effects were considered in two cases: (1) the correlated site was included within the prescribed portion (the gene body, excluding the first 5 Kbp) of another gene, and (2) the correlated site was located within the promoter (from TSS-1500 bp to TSS + 2500 bp) of another gene. For these cases, a correlation between the expression level of the genes was tested, and circuits with *R*^2^ > 0.1 that fit one of the scenarios described in Additional file 2: Fig. S7 were excluded. For model developing, we excluded circuits which mismatched the report assay: circuits with methylation sensitive TAS (which were calculated for the DNA segments overlapping the given site and were changed by × 1.5 fold by methylation) which mismatched the canonical mode (i.e., groups I and II in Fig. 2g).

#### Methylation-based prediction of gene expression

For each gene, we performed two methods: (1) multiple linear regression and (2) lasso regression. (1) In multiple linear regression, we should reduce the number of variables since we have only 24 samples. Thus, we tested all the possible combinations of one to four associated sites. For each combination with full data in at least 12 tumors, we generated a predictive model of expression level based on multiple linear regression of the sites’ methylation levels. A significant model (*q* value < 0.05) was evaluated by ANOVA for linear model fit and corrected for the number of possible models per gene by FDR. A gene was considered to have a synergic model if the predictive value of the model was better than each of the involved sites alone.

Validation of methylation-based predictions was performed using the leave-one-out cross validation approach for assessing the generalization to an independent dataset. One round of cross-validation involves 23 datasets (called training set) in which performing all the analysis and one sample for validating the analysis (called testing set). The cross-validation was performed 24 times. For each training dataset, *cis*-regulatory circuits were generated (as described in the “Circuit annotation” section), and possible predictive models were developed for the targeted genes. Prediction quality of each gene was then tested in the 24 rounds, by comparing predicted versus observed expression level. Difference up to 2-fold was considered as a success. The ability to accurately predict the expression level of a gene was considered verified if it has good prediction quality in at least 20 of the 24 rounds.

The ability to predict gene expression alterations was performed by analyzing the genes with irregular expression levels in certain tumors. Out of 4248 analysis sets (177 genes in 24 left-out tumors), 2652 were for genes with prediction models. Of them, 868 were for genes with irregular expression (expression level in the left-out tumor was > 1SD of the gene expression levels across the tumors). Predictions in which the prediction errors were within twofold of the observed expression-level were considered accurate.

### Analysis of coding sequence variations

VCF files describing single nucleotide variations (SNV) were provided by the DKFZ. Synonymous SNV, SNVs overlapping with published SNPs (COMMON), or SNVs with a less than 25-read coverage or bcftools-QUAL score > 20 were excluded. Copy number variations (CNV) were analyzed by whole-genome sequencing (WGS) data provided by the DKFZ. Association between gene expression and copy number was evaluated by Pearson or Spearman’s correlations. *p*-values were adjusted for multiple-hypothesis testing using the Benjamini–Hochberg method, with FDR < 5%.

### Analysis of regulatory sequence variations

#### Pre-alignment processing

GBM tumors (*n* = 8) were sequenced using the paired-end 250- or 300-bp read protocol in Illumina MiSeq V2 or V3 devices. FASTQ files were filtered, and sequence edges of Phred score quality > 20 were trimmed up to 13 bp of Illumina adapter applying Trim Galore (http://www.bioinformatics.babraham.ac.uk/projects/trim_galore/). Reads that were shortened to 20 bp or less were discarded, along with their paired read. Exclusion of both reads was implemented after verifying that retention of unpaired reads did not significantly increase high-quality alignment coverage. Quality control of the original and filtered FASTQ files was performed with FastQC (http://www.bioinformatics.babraham.ac.uk/projects/fastqc), deployed to verify the reduction in adapter content and the increase in base quality following the filtering stage. Duplicates were removed at the pre-alignment stage with FastUniq [76]. Duplicate pair-ends were removed by comparing sequences rather than post-aligned coordinates, allowing preservation of variant information.

#### Sequence alignment

Sequences were aligned to GRCh37/hg19 assembly of the human genome applying paired-reads Bowtie 2 [73]. Discordant pairs or constructed fragments larger than 1000 bp were discarded, thereby improving mapping quality by allowing both reads to support mapping decisions. Default values (Bowtie 2 sensitive mode) were applied to end-to-end algorithm parameters, seed parameters, and bonus and penalty figures. Outputted SAM and BAM alignment files were examined using the Picard CollectInsertSizeMetrics utility to verify correctness of final insert-size distribution (http://broadinstitute.github.io/picard; version 1.119).

#### Variation calling

A BCF pileup file was generated from each BAM file using the samtools [77] mpileup function, set to consider bases of minimal Phred quality of 30 and minimal mapping quality of 30. Variant calling performed using bcftools was initially set to output SNPs only to create SNP VCF files, according to the recommended setting for cancer [78]. The VCF files were filtered by applying depth of coverage (DP) above 40 and statistical quality (QUAL) above 10. DP filtering in this context refers to DP/INFO in the VCF file, which is a raw count of bases.

#### Variant post-processing

Post-processing of VCF SNPs included additional filtering, variant frequency calculation, mapping variants to probes, and mapping variants to public databases, performed with a custom-written Python script. Additional depth coverage filtering of 20 was applied on the high-quality bases, which were selected by bcftools as appropriate for allelic counts. Frequency calculations were based on high-quality allelic depth (ratio of each allelic depth to sum of all allelic depths). SNPs were mapped to the following dbSNP [79] and ClinVar [80] databases: dbSNP/common version 20170710, dbSNP/All version 20170710, and clinvar_20170905.vcf. A match was determined when the position, reference, and variant were all in agreement. In our analysis, we refer to de novo variations (not in COMMON and not in ALL) which were detected in at least one sample (of eight). For each targeted gene, we counted the number of de-novo variations that were at a distance of ± 500 bp from its correlated sites.

#### Regulatory CNVs

Non-coding CNVs were detected from WGS of 5 Kbp sliding blocks in 2-Mbp region flanking gene TSSs, with a 50% overlap. Correlation of the total copy number (TCN) of each block with the gene expression level was assessed (at least six samples with available TCN data, Pearson and Spearman correlation). Correlation *p* values were adjusted for multiple-hypothesis testing using the Benjamini–Hochberg method.

### Genome editing

#### Design and cloning of sgRNA

Guides to perturb *SMO* regulatory units were designed using the ChopChop, E-CRISP, and CRISPOR software; 20-bp sgRNA sequences followed by the PAM “NGG” for each unit were identified and synthesized. For the *SMO* regulatory unit at chr7:128,507,000–128,513,000 designated unit “A,” 4 guides were cloned into a backbone vector bearing puromycin resistance (addgene, 51,133), using the Golden Gate assembly kit (NEB® Golden Gate Assembly Kit #E1601). Each guide sequence was cloned with its own U6 promoter and was followed by a sgRNA scaffold. For the regulatory unit at chr7:129,384,500–129,389,500, designated unit “D,” two guides were cloned into the same backbone plasmid using the same method (Additional file 2: Fig. S7).

#### Transfection/CRISPR–Cas9-mediated deletion

After validating the sgRNA sequences by Sanger sequencing, T98G or T98Gdelta*SMO*-D cells were co-transfected with a Cas9-bearing plasmid (addgene, 48138) and either the plasmid bearing the guides targeting *SMO* A, the plasmid with bearing the guides targeting *SMO* D, or the same plasmid harboring a non-targeting gRNA sequence (scramble) as a negative control. The molar ratio between the transfected guide plasmid and the Cas9 plasmid was 1:3, in favor of the plasmid not carrying the antibiotic resistance. 1.5–3 × 10^5^ cells/ml, > 90% viable, were plated 1 day prior to transfection in a 6-well dish. On the transfection day, each well received 3 μL Lipofectamine® 3000 Reagent, 5 μg total plasmid DNA, and 10 μL of Lipofectamine® 3000 Reagent (2:1 ratio). Puromycin (3 μg/μL) was added to the cells 1 day after transfection. After 72 h, the antibiotic was washed, and the cells were left to expand. The cells were harvested 8–21 days post-transfection, and genomic DNA and RNA were immediately collected (Qiagen; DNeasy #69504 and RNeasy #74106, respectively).

#### Genotyping of mutant populations

Genomic DNA was subjected to genotyping PCR (primers listed in table). Deletion or partial deletion was confirmed by gel electrophoresis or TapeStation, by Sanger sequencing, and by Illumina MiSeq sequencing (150 bp paired-end). Sanger sequencing analyzed using BLAST® and sequence logo was generated using by ggseqlogo R package [81]. RNA extracted from populations of cells bearing such mutations was then checked for an effect on *SMO* transcription level, using qPCR (QuantStudio 3 cycler, Applied Biosystems, Thermo Fisher Scientific, Waltham, MA, USA).

#### Single-cell dilution to obtain CRISPR-targeted cell clones

Puromycin-selected cells were isolated by trypsinization, counted and diluted to a concentration of 20 cells/100 μL. Diluted cells (200 μL) were then serially diluted, to ensure single-cell occupancy of rows 6–8 (eight dilution series). By calibrating the number of cells in the first row, we ensured that single cells could be isolated from the sixth to eighth rows onwards. Cells were incubated until the low-density wells were confluent enough to be transferred to 24-, 12-, and finally to 6-well plates. Selected clones were tested for a stable DNA profile and for *SMO* transcription level by genotyping PCR (primers listed in table), followed by gel electrophoresis or TapeStation and qPCR analysis, respectively.

#### RT-qPCR

Each isolated mRNA (500 ng) was transcribed to cDNA using the Verso cDNA Synthesis Kit (#AB-1453/A, Thermo Fisher Scientific, Waltham, MA, USA) according to provided instructions, using the oligo dT primer. qPCR was performed using the Fast SYBR™ Green Master Mix (#AB-4385612, Thermo Fisher Scientific, Waltham, MA, USA) and qPCR primers for *SMO* and reference genes *HPRT* and *TBP* (see table), on a QuantStudio 3 cycler (Applied Biosystems, Thermo Fisher Scientific, Waltham, MA, USA). The reaction was conducted in triplicates, and 20 ng of template was placed in each well. For each primer set, a no-template control (NTC) was also run, to check for possible contamination. QuantStudio Design & Analysis Software v1.4.3 (Applied Biosystems, Thermo Fisher Scientific, Waltham, MA, USA) was used for analysis. All presented data were based on three or more biological replications of the genome editing experiments, each with three technical repeats of the DNA and RNA.

Guide list

**Table:** 

A1	ACCCTGCGCGCCGAGGTATC
A2	GCGACCTGGGAGCCGCCGCC
A3	ACCGCCGGTGCCGACCTTTG
A4	GCGTGGTAGTCCTTCTCCGG
D1	GTCCTGCTCTATCTTGTCGT
D2	CACATGTAGGTCTTTCTGAC
N1	CCGGCTCTGGGACTTACACCAATG
N2	CCGGACGGTGGATCTTCTTTAGTT
N3	CCGGTCCACCTTTTTGTTTCCTCT
N4	CCGGAAGATGGATGTCCCAGCACC

Primer list

**Table:** 

Genotyping *SMO* A (F)	1066F	GCAGTGCGCTCACTTCAAA
Genotyping *SMO* A (R)	1066R	CTCCTGGGGCGAGATCAAAG
Genotyping *SMO* D (F)	1069F	CATGGTCCCGGTTCCCATTTGG
Genotyping *SMO* D (R)	955R	GCCCTCCACAGACCAAACAGC
Genotyping *SMO* NULL (F)	1120F	GCTCAGTCTCAGTGTGGGAG
Genotyping *SMO* NULL (R)	1120R	GGCGTTTCCACAAGAGATGAGC
qPCR *SMO* F	950F	TGCTCATCGTGGGAGGCTACTT
qPCR *SMO* R	950R	ATCTTGCTGGCAGCCTTCTCAC
qPCR *HPRT* F	442F	TGACACTGGCAAAACAATGCA
qPCR *HPRT* R	442R	GGTCCTTTTCACCAGCAAGCT
qPCR *TBP* F	850F	TGCACAGGAGCCAAGAGTGAA
qPCR *TBP* R	850R	CACATCACAGCTCCCCACCA

### Statistics and data visualization

All analyses were performed using both public and custom scripts written in R (http://www.R-project.org) and MATLAB (The Mathworks, Inc.). Plots were generated using plotting functionalities in base R and using ggplot2 package (https://ggplot2.tidyverse.org) and corrplot package (https://github.com/taiyun/corrplot). Sequence logos were generated using the ggseqlogo package [81]. Heatmaps were produced using the ComplexHeatmap package [82]. Lasso regression was performed using the default parameters of gmlnet package [83].

### Supplementary Information

**Additional file 1: Table S1.** Driver and reference cancer genes. **Table S2.** DNase hypersensitivity signals within the targeted chromatin regions. **Table S3.** Design and sequences of the RNA probes used for the target-enrichment assay. **Table S4.** Distribution of RNA probes per targeted gene domain. Probes were allocated to all methylation sites within the assigned variable-chromatin blocks. **Table S5.** Glioblastoma samples. **Table S6.** Qualities of the libraries of captured DNA segments. **Table S7.** Functional regulatory elements before and after DNA methylation. **Table S8.** Inferred regulatory circuits*. **Table S9.** Methylation-based gene expression models. **Table S10.** Gene-associated regulatory units. **Table S11.** Model predictions. **Table 12.** Reads distribution per probe pre and post metylation.

**Additional file 2: Supplemental Note S1** [87, 88]**.** **Supplemental Figure S1.** Studied genes and domains versus topological associated domains (TAD). **Supplemental Figure S2.** Associations between DNA methylation and gene expression across cancer types. **Supplemental Figure S3.** Overall flow and terminology of the study. **Supplemental Figure S4.** Functional annotation of isolated regulatory elements. **Supplemental Figure S5.** Characteristics of methylation-sensitive and methylation-insensitive elements. **Supplemental Figure S6.** Eliminated associations due to possible secondary effects. **Supplemental Figure S7.** Alignment of positive and negative units with silencers and enhancers. **Supplemental Figure S8.** Genomic deletions. **Supplemental Figure S9.** Compliance between assays. **Supplemental Figure S10.** Methylation-methylation coordination maps of genes with multiple regulatory circuits. **Supplemental Figure S11.** Gene-specific networks. **Supplemental Figure S12.** Models of inter-patient variation in reference genes. **Supplemental Figure S13.** Gene models developed by Lasso-type analysis. **Supplemental Figure S14.** Cellular functions of misregulated driver genes. **Supplemental Figure S15.** Map of the screening vector used for functional analyses of isolated DNA segments. **Supplemental Figure S16.** Properties of a captured library. **Supplemental Figure S17.** Efficiency of the in-vitro methylation assay. **Supplemental Figure S18.** Properties of a plasmid library.

**Additional file 3.** Methylation-methylation correlation for genes with multiple regulatory circuits. Matrixes presenting the methylation versus methylation correlation (R) between two of the associated sites. Genomic locations of the associated sites are given to the left, the sites chosen by best prediction models (Fig. 6) are highlighted. Genomic maps of the associated sites (red bars), units (letters) and genes (purple) are given as well.

**Additional file 4.** Methylation-methylation correlation across overlapping gene domains. Matrixes presetting the correlation map of a gene which share its domain with another gene (the other gene’s map is presented below). Each square in the matrixes show the methylation versus methylation correlation (R) between two of the associated sites. Gene symbols and genomic locations of the associated sites are given to the left. White squares denote no correlation (R2 <0.1).

**Additional file 5.** Evaluation of methylation-based prediction by 23-leaving-1 tests. Scatter plots showing the predicted versus observed gene expression levels in one of the 24 independent tests. Cancer driver genes (red dots), GBM subtype signature (green), non-driver GBM candidate (blue) and non-driver variable (black) genes are shown. Gene expression values are in log2 RSEM. The number of test sets, name and subtype of the leaving-out tumor, and the obtained correlation value are indicated above each plot.

**Additional file 6.** Review history.

## Data Availability

Data generated in this study are available at the NCBI Gene Expression Omnibus (https://www.ncbi.nlm.nih.gov/geo) under accession number GSE163021 [84]. Whole-genome, whole-exome, H3K4me1, and H3K27ac chromatin immunoprecipitation data: GEO accession number GSE121719 [85]. RNA sequencing of the GBM biopsies and the normal brain samples: GSE121720 [86]. All other relevant data supporting the key findings of this study are available within the article and its Information files. Manipulated cells and genome-editing vectors may be obtained from the Hellman lab upon justified request.
