# Supplementary material for: Methylation-directed regulatory networks determine enhancing and silencing of mutation disease driver genes and explain inter-patient expression variation
Source: Genome Biol. 2023 Nov 28;24:264. doi: 10.1186/s13059-023-03094-6 (PMC10683314; doi:10.1186/s13059-023-03094-6)
Supplement: Supplementary file 2 — Additional file 2: Supplemental Note S1 [87, 88]. Supplemental Figure S1. Studied genes and domains versus topological associated domains (TAD). Supplemental Figure S2. Associations between DNA methylation and gene expression across cancer types. Supplemental Figure S3. Overall flow and terminology of the study. Supplemental Figure S4. Functional annotation of isolated regulatory elements. Supplemental Figure S5. Characteristics of methylation-sensitive and methylation-insensitive elements. Supplemental Figure S6. Eliminated associations due to possible secondary effects. Supplemental Figure S7. Alignment of positive and negative units with silencers and enhancers. Supplemental Figure S8. Genomic deletions. Supplemental Figure S9. Compliance between assays. Supplemental Figure S10. Methylation-methylation coordination maps of genes with multiple regulatory circuits. Supplemental Figure S11. Gene-specific networks. Supplemental Figure S12. Models of inter-patient variation in reference genes. Supplemental Figure S13. Gene models developed by Lasso-type analysis. Supplemental Figure S14. Cellular functions of misregulated driver genes. Supplemental Figure S15. Map of the screening vector used for functional analyses of isolated DNA segments. Supplemental Figure S16. Properties of a captured library. Supplemental Figure S17. Efficiency of the in-vitro methylation assay. Supplemental Figure S18. Properties of a plasmid library. [file 13059_2023_3094_MOESM2_ESM.docx]

**Supplemental Notes and Figures**

[**Supplemental Note S1** 3](#_Toc80044706)

[**Supplemental Figure S1**. Methylation-expression associations in various cancer types 4](#_Toc80044707)

[**Supplemental Figure S2**. Associations between DNA methylation and gene expression across cancer types 5](#_Toc80044708)

[**Supplemental Figure S3**. Overall flow and terminology of the study 7](#_Toc80044709)

[**Supplemental Figure S4**. Functional annotation of isolated regulatory elements 8](#_Toc80044710)

[**Supplemental Figure S5**. Characteristics of methylation-sensitive and methylation-insensitive elements 10](#_Toc80044711)

[**Supplemental Figure S6**. Eliminated associations due to possible secondary effects 11](#_Toc80044712)

[**Supplemental Figure S7**. Alignment of positive and negative units with silencers and enhancers 12](#_Toc80044713)

[**Supplemental Figure S8**. Genomic deletions 16](#_Toc80044714)

[**Supplemental Figure S9**. Compliance between assays 17](#_Toc80044715)

[**Supplemental Figure S10**. Methylation-methylation coordination maps of genes with multiple regulatory circuits 19](#_Toc80044716)

[**Supplemental Figure S11**. Gene-specific networks 20](#_Toc80044717)

[**Supplemental Figure S12.** Models of inter-patient variation in reference genes 21](#_Toc80044718)

[**Supplemental Figure S13**. Gene models developed by Lasso-type analysis 22](#_Toc80044719)

[**Supplemental Figure S14**. Cellular functions of misregulated driver genes 23](#_Toc80044720)

[**Supplemental Figure S15**. Map of the screening vector used for functional analyses of isolated DNA segments 24](#_Toc80044721)

[**Supplemental Figure S16.** Properties of a captured library 25](#_Toc80044722)

[**Supplemental Figure S17.** Efficiency of the in-vitro methylation assay 26](#_Toc80044723)

[**Supplemental Figure S18.** Properties of a plasmid library 27](#_Toc80044724)

# Supplemental Note S1

In agreement with former glioblastoma analyses [87, 88], analyses of copy number variations in the studied tumors showed moderate karyotype aberration (average tumor ploidy = 2.5) (Supplemental Table S5). Since copy-number alterations are not necessarily accompanied by abnormal expression of residing genes, we assessed functional events by examining the correlation between gene copy number and mRNA levels. Genes displaying significant correlations (R^2^>0.3; p<0.05) between their copy numbers and expression levels across the tumors, and corresponding two-fold or greater expression deviation from normal brain tissue, were considered misregulated genes attributable to gene copy number variation (CNV). *EGFR* was the only gene that fully met these criteria. Possible subtle effects were observed for *PDGFRA/KIT, MET* and *CHEK2*. Overall, 0-2 genes per tumor were potentially altered by coding CNVs. To evaluate regulatory sequence mutations, single-nucleotide variations (SNV) were analyzed in the regulatory segments captured from eight of the GBM tumors, as listed in supplemental table S5. Only one potentially contributing variation reveled in 100-bp windows around gene-associated sites, and extended analyses using larger window sizes revealed no additional events. Theoretically, amplification or deletion of enhancer sequences may also affect transcription. However, a full screen of CNVs in 5,000 bp windows across the driver gene domains revealed no events apart from gene CNVs.


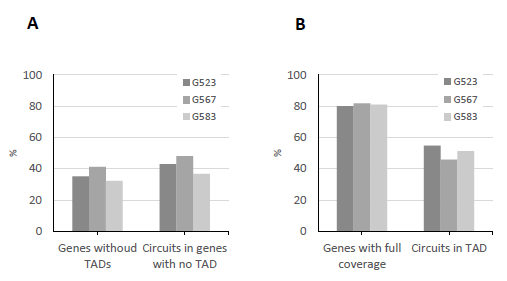


**Targeted domains Vs. TADs**

**Targeted genes Vs. TADs**

Supplemental Fig. S1. Studied g**enes and domains versus topological associated domains (TAD). (A)** **Left**: Fractions of the studied genes for which the boundaries of topological associated domains (TAD) were not well defined, following Hi-C analysis of three GBM samples (25kb resolution) [68]. **Right**: Fractions of methylation-related regulatory circuits of genes without identified TADs, out of all uncovered circuits. **(B)** **Left**: Fractions of genes with defined TADs, for which the applied targeting criteria (windows of two million base-pairs around transcription start sites) provide full coverage of the TAD. **Right**: Fractions of circuits within defined TADs, out of all uncovered circuits.

(
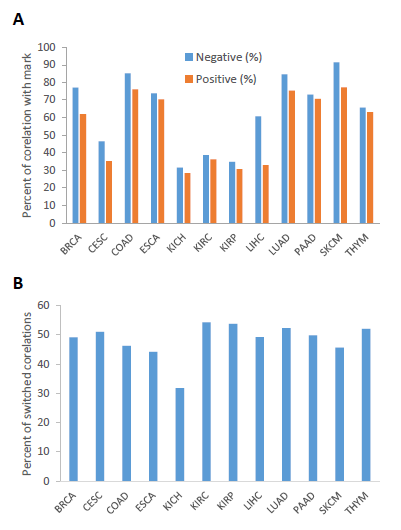


**Switching of methylation sites between positive and negative effects on expression**

**Associations between methylation and expression in H3K4me1-marked regions**

Supplemental Fig. S2. Associations between DNA methylation and gene expression across cancer types**. (A)** Percentages of negatively and positively-associated sites that carry H3K4me1 marks, out of all gene-associated sites across various types of cancer. **(B)** Percentages of gene-associated methylation sites in given types of cancers, which displayed the opposite effects on expression of the associated genes in at least one other cancer type. The analyses performed using public data (TCGA).

Supplemental Fig. S3. Overall design and terminology of the study**. (A)** Domains of the human genomes that have been explored, covering one million base pairs to each side of the transcription start sites (TSSs) of 177 driver and reference cancer genes. **(B)** Within these domains, we located the regions showing variable marking of regulatory chromatin across the analyzed tumors. **(C)** Biotinylated RNA Probes (120 bp each) were designed to target the CpG methylation sites within the identified chromatin regions. **(C)** Biotinylated RNA Probes (120 bp each) were designed to cover all CpG methylation sites within the identified chromatin regions. **(D)** Randomly-sheared DNA segments of tumor genomic DNAs were allowed to attach to (partially or fully) overlapping RNA probes. **(E)** Pulling-out the attached segments yielded a library of captured DNA segments of various sizes (mean = 224 bp). The distribution of the sizes of the captured segments in an example library (sample #100) is shown. **(F)** The captured segments were then integrated into gene-reporting vectors, forming a library of reporter assays. **(G)** Enhancer or silencer functionalities were analyzed in 500 bp (50% overlapping) windows across the studied regions, before or after methylation of the vectors, thus allowing to locate significant (FDR q value < 0.05) methylation-sensitive and insensitive enhancer & silencer elements, and uncovering the general rules of enhancers’ and silencers’ responses to extreme methylation conditions **(H). (I)** In parallel, the libraries of captured DNA segments were sequenced with or without bisulfite treatment. **(J)** The correlation between the methylation levels of each methylation site and the expression of the explored genes over the tumors were analyzed, revealing methylation-related regulatory circuits of genes. **(K)** Clusters of methylation sites that associated with a given gene, and have same effect on its expression, defined as methylation-related regulatory units. **(L)** The units of given genes defined as the methylation-related, cis-regulatory network of the gene. **(M)** Key methylation sites described and predict inter-patient expression variation.

Supplemental Fig. S4. Functional annotation of regulatory elements: Putative regulatory segments (mean size=224bp) were captured from a GBM tumor, ligated into gene-reporter vectors, and allowed to drive own transcription in T98G glioblastoma (GBM) cells. Plasmid DNA and RNA were then extracted from the GBM cells and sequenced. Transcriptional activity scores (TASs) of the targeted elements were calculated by summing the ratio between DNA and RNA copy numbers in 500 bp, 50% overlapping windows, and normalizing the obtained values to the total DNA to RNA ratio of the library.

**Effect of DNA methylation** (example regulatory elements)

**A**

**
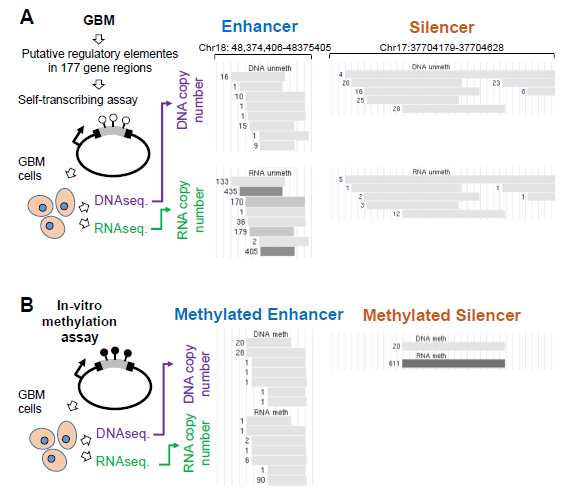
**

**Chromatin characteristics of methylation-sensitive and insensitive elements**

**B**

**Average effect of methylation in even-number groups of regulatory elements**

**C**

Supplemental Fig. S5. Effect of DNA methylation on regulatory elements**. (A)** Representative examples of enhancer and silencer elements before (top) or after (bottom) in-vitro methylation. DNA and RNA copy numbers are indicated to the left of each segment. **(B)** Characteristics of methylation-sensitive and methylation-insensitive elements. The levels of transcription factors binding (TFB), factor variety (breadth), and DNase I hyper-sensitivity are shown across a variety of different cell types (ENCOD Average number of CpG methylation sites per element are shown as well. **(C)** Average effects of DNA methylation on regulatory elements. Methylation-sensitive elements were ranked by pre-methylation TASs, and assigned into even-number (20-quantiles) groups. Average values of the groups are shown before (TAS) or after methylation (Methyl.TAS). The average activity shifts (∆TAS) are shown as well.

Supplemental Fig. S6. Eliminated associations due to possible secondary effects**.** **(A)** Prohibited association between methylation of a promoter site and expression of a possible activator of the indicated gene A. **(B)** Prohibited association between methylation of a promoter site and expression of a possible repressor of the indicated gene A. **(C)** Prohibited association between methylation of a gene-body site and expression of a possible activator of the indicated gene A. **(D)** Prohibited association between methylation of a gene-body site and expression of a possible repressor of the indicated gene A.

Supplemental Fig. S7. Compliance between assays**. (A)** Fractions of regulatory elements located by the gene-reporting assay, for which the analysis of actual tumors revealed an adjacent (≤500bp) gene-associated regulatory site. **(B)** Fractions of gene-associated regulatory sites for which the reporter-gene analysis revealed an adjacent (≤500bp) regulatory element. **(C)** Impact of DNA methylation on regulatory activity of GBM-related sites. The analysis performed as in figure 2g, but for 4,434 negatively-correlating sites with positive TAS (enhancers) and 3,274 positively-correlating sites with negative TAS (silencers). TAS was calculated for the DNA segments overlapping the given sites.

**A**

**B**

**C**

- *SMO* domain (2Mbp): **28K methylation sites**
- In targeted chromatin-regions: **988 methylation sites**
- Successfully captured and sequenced (at least 100x in at least 8 tumors): **837 methylation sites**
- Significant correlations (R_­2_>0.3, q<0.05): **56** **methylation sites**
- SMO circuits: **45 methylation sites (26 negative, 19 positive)**
- Genomic clusters (units): **5** **(3 negative, 2 positive)**

- Excluded due to possible secondary effects: **11**

**Mapping and annotation of *SMO* gene domain**

**A**


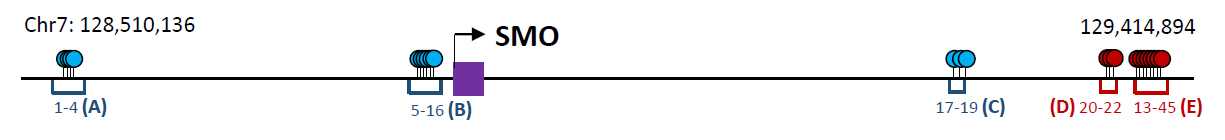

**B**

**Manipulated elements**


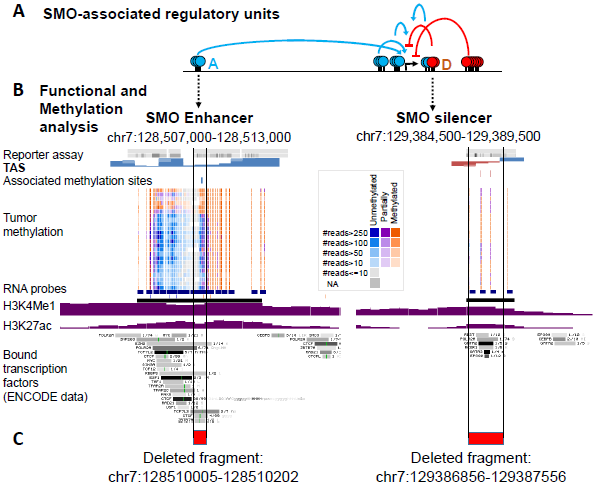


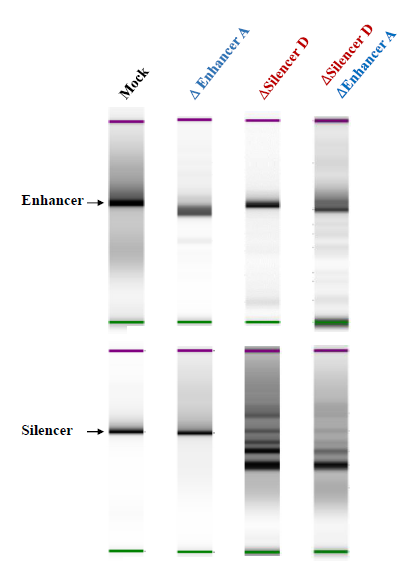


**C**

**D**

Supplemental Fig. S8. Genomic manipulations of regulatory elements**.** **(A)** **Top:** Mapped methylation sites, circuits and units in the *SMO* gene-domain. **Middle:** Correlations between *SMO* expression and methylation of indicative sites from units A, B, D and E, across GBM tumors. **Bottom:** TAS analyses in 5Kb windows centered at the indicated sites. **(B)** Genetic maps of enhancer (left) and silencer (right) regions that were subjected to genomic editing in GBM cells. The next data items are listed from top to bottom: Genomic coordinates (hg19), DNA copy number of captured segments, RNA copy number produced by the captured segments, TAS analyses in 500bp (50% overlapping) windows (blue: transcriptional enhancing, orange: transcriptional silencing), Methylation sites that associated with *SMO* expression in GBM tumors (blue: negative associations, orange: positive associations), sequencing read-depth and methylation levels of the analyzed methylation sites in 24 GBM tumors, targeting RNA probes (blue line), methylation sites that may be assessed using the Illumina 450K commercial microarray, targeted chromatin regions (black lines), regulatory chromatin-marker H3K4Me1 (fraction of tumors from 0-100% carrying this mark), active chromatin-marker H3K27ac (fraction of tumors from 0-100% carrying this mark), lists of transcription factors that bind these regions in various cell types (ENCODE data), DNA fragments that were deleted from GBM genomes (red lines). **(C)** Electrophoreses of the targeted enhancer 'A' and silencer 'D' units. Arrows indicate the unit sizes prior to genomic editing. **(D)** Designs and sequencing of the deletions of silencer unit 'D', enhancer unit 'A', or the co-deletion of the both. Gray: PCR primers used for the sequencing reactions, yellow: CRISPR targeting guides, triangles: DNA breaks at the borders of the deleted fragments, NGS: next-generation sequencing, Sanger: Sanger sequencing (the deleted fragment shown in gray).

Supplemental Fig. S9. Effect of a DNA segment located between the *SMO* units. **Up:** Map and design of the targeted element. Gray: PCR primers used for the sequencing reaction, yellow: CRISPR targeting guides, triangles: DNA breaks at the borders of the deleted fragments. **Middle left:** Gel electrophoresis of untreated or manipulated cells. **Middle right:** Effect of the genomic deletion on *SMO* expression. Averages of four biological repeats, each includes three technical repeats, are shown, as compared with *SMO* expression on following mock treatments with scrambled Cas9 guides. **Bottom:** DNA sequencing of the manipulated cells (the deleted fragment shown in gray).


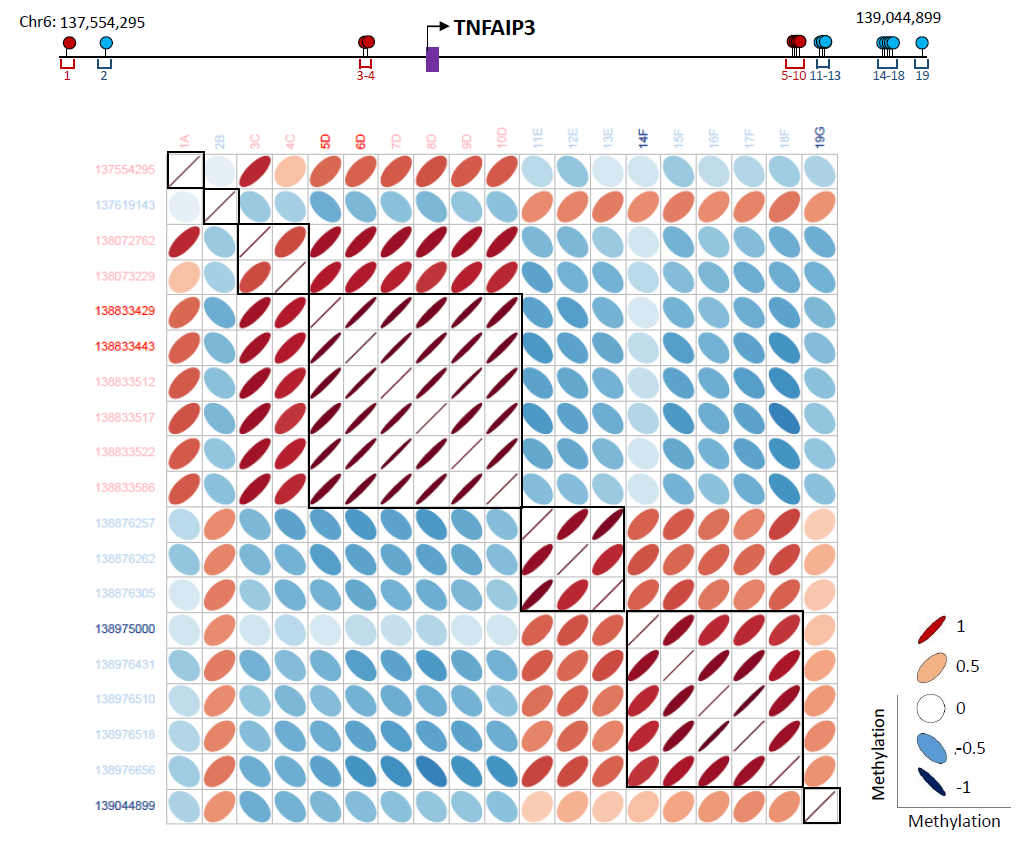


**A**

**B**


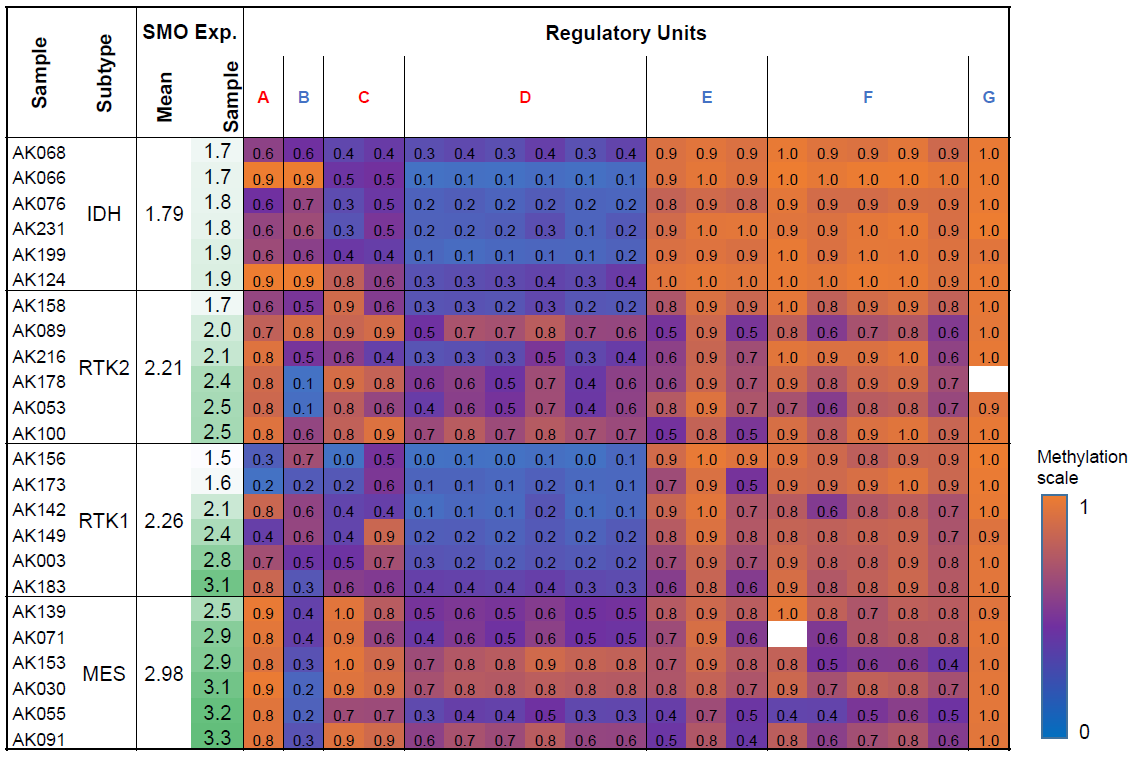


Supplemental Fig. S10. Interaction between the regulatory units of ***TNFAIP3***. (A) Top: Genomic maps showing the locations of the *TNFAIP3*-associated sites and units. Bottom: Coordination between the methylation levels of *TNFAIP3*-associated sites and units. Each square in the matrix show the methylation versus methylation correlation (R) between two of the associated sites. Genomic locations of the associated sites are given to the left, locations of the sites that consist the *TNFAIP3* expression models (see Figure 6) are highlighted. The coordination maps of the other analyzed genes are given in Additional file 3. (B) *TNFAIP3* expression level versus the methylation levels of *TNFAIP3*-associated sites and units. Tumor samples and subtypes are as described in Table S5. Associated sites and units are as described in panel **A**.


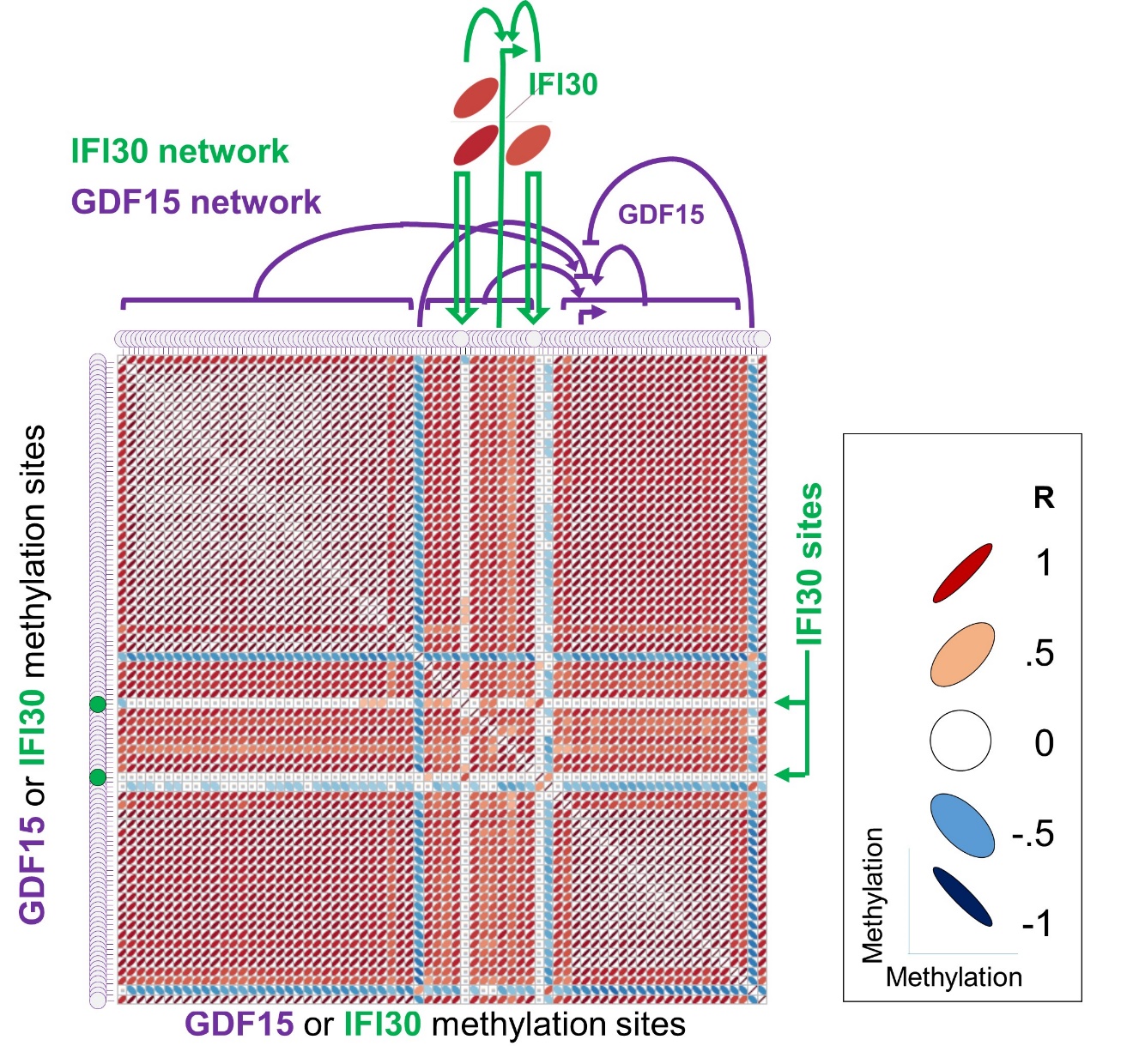


Supplemental Fig. S11. Matrix showing the coordination between the methylation levels of sites associated with the *GDF15* (purple) or with the *IFI30* (green) genes are shown. Each square in the matrixes show the methylation versus methylation correlation (R) between two of the associated sites. White squares denote no correlation (R^2^ <0.1). Maps of the other analyzed overlapping gene domains are given in Additional file 4.


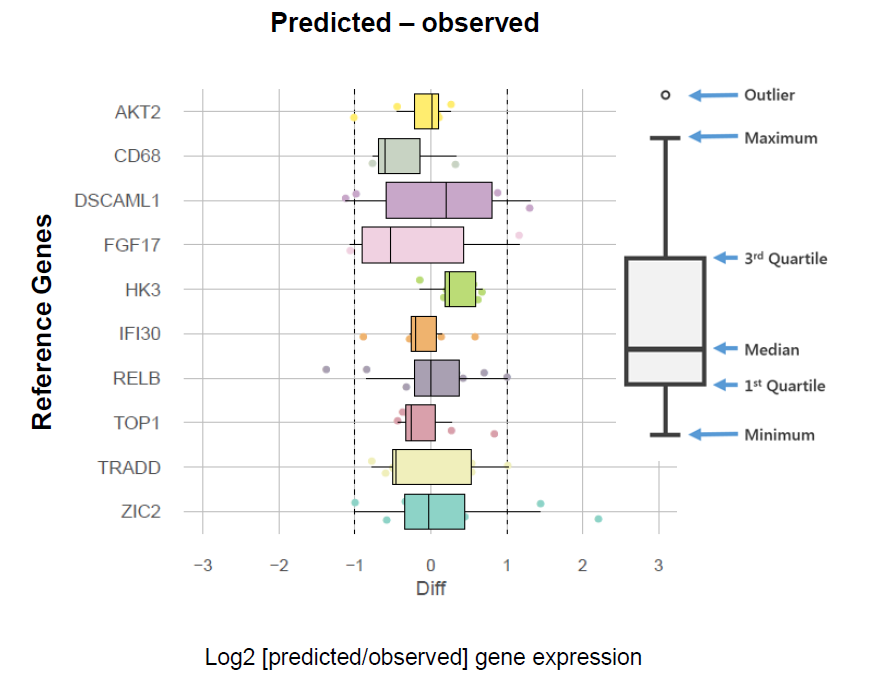


Supplemental Fig. S12**.** Log 2 of the differences between predicted and observed gene expression levels for reference (non-driver) genes with developed models. Box plots describe the distributions of prediction errors in 24 independent tests.


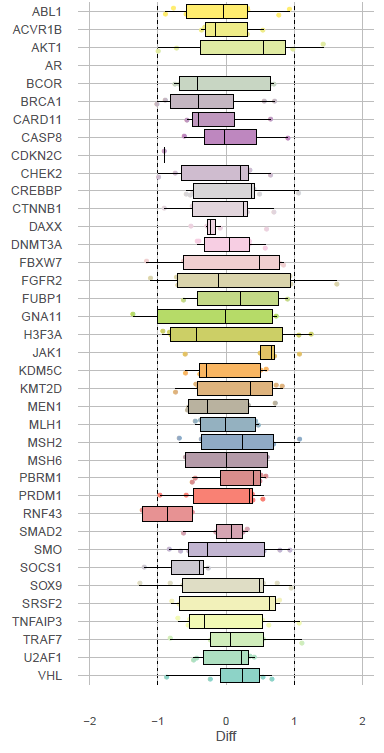


Supplemental Fig. S13. Prediction qualities of gene-expression models developed by LASSO**.** Gene-expression models were developed and validated as described in Figure 6d, but using a least absolute shrinkage and selection operator (LASSO) approach. Box plots describe the distributions of prediction errors in 24 independent tests.


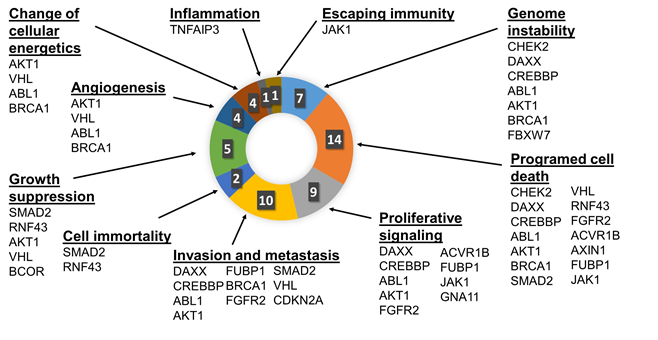


Supplemental Fig. S14. Cellular functions of misregulated driver genes for which a methylation-based model of expression variation was developed.


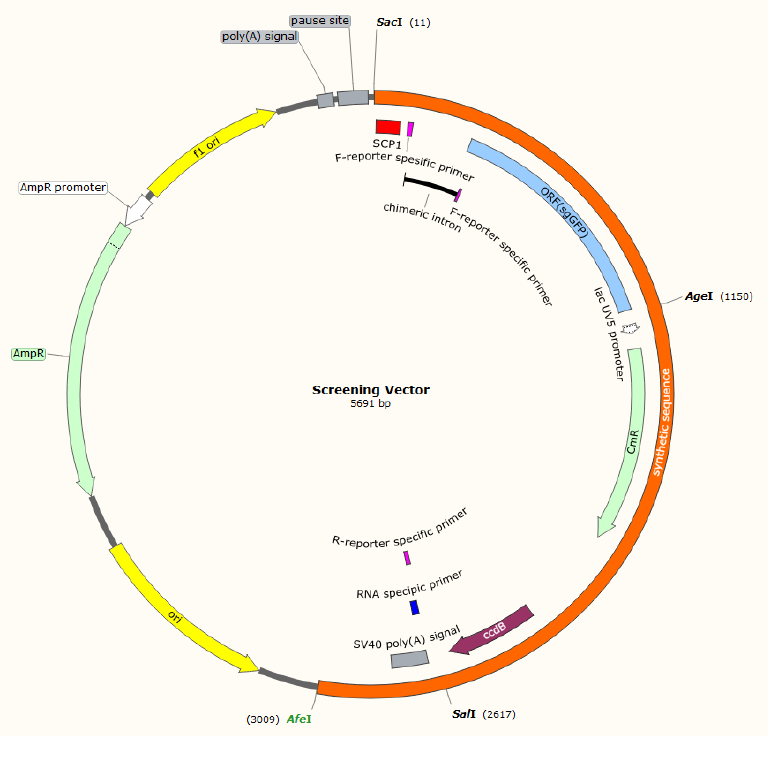


Supplemental Fig. S15. Map of the screening vector used for functional analyses of isolated DNA segments**.** The sequence between the SacI and the AfeI sites in the original pGL3-promoter vector (Promega, GenBank accession number U47298) was replaced with the sequences shown here. The modified vector produced a certain amount of basal transcription when no regulatory elements was presented. To evaluate regulatory functionality, putative silencer or enhancer elements were incorporated between the AgeI and the SalI sites.


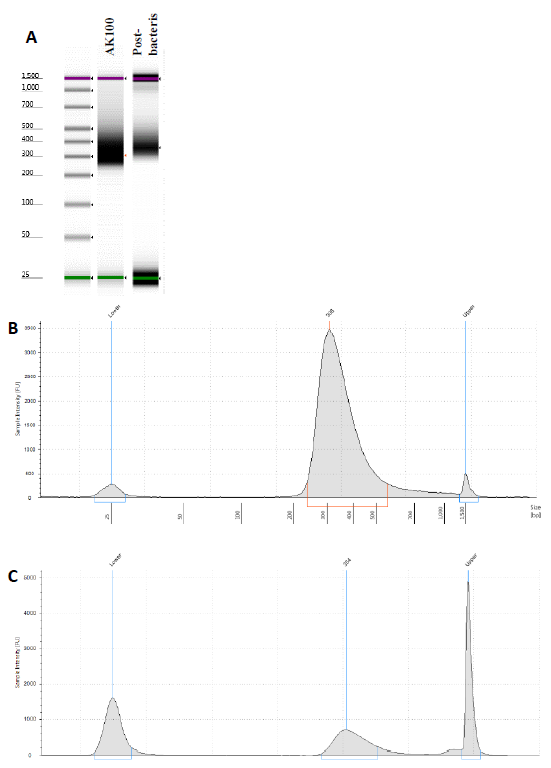


Supplemental Fig. S16**.** Gel images **(A)** and size distributions of sample #100 target-enriched library before **(B)** and following **(C)** propagation in bacteria

Supplemental Fig. S17**.** Efficiency of the in-vitro methylation assay confirmed by digestion with the methyl-sensetive HpaII restriction enzyme of libraries of captured genomic segments following amplification and propagation in bacteria (de-methylation) or following amplification, propogation in bacteria, and in-vitro methylation (re-methylation).

De-methylation

De-methylation

Re-methylation

**HpaII**

**+**

**+**

-

**-**


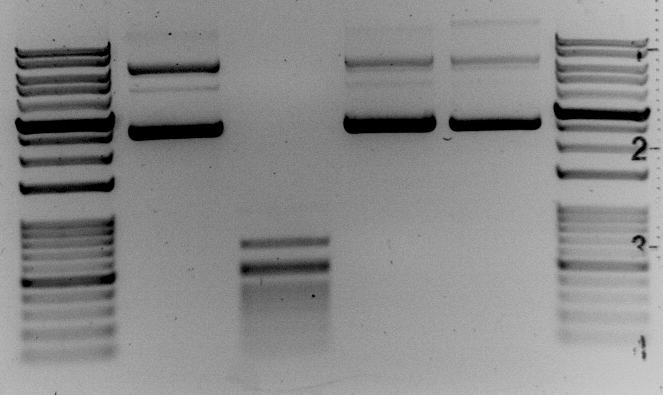


Re-methylation


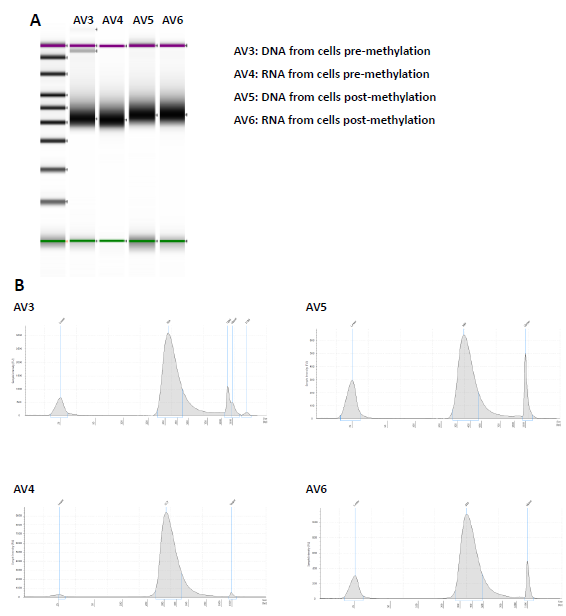


Supplemental Fig. S18. Gel image **(A)** and size distribution **(B)** of plasmid DNAs and RNAs extracted from T98G cells following transfection with methylated or un-methylated sample #100 libraries.
