# Supplementary material for: Methylation-directed regulatory networks determine enhancing and silencing of mutation disease driver genes and explain inter-patient expression variation
Source: Genome Biol. 2023 Nov 28;24:264. doi: 10.1186/s13059-023-03094-6 (PMC10683314; doi:10.1186/s13059-023-03094-6)
Supplement: Supplementary file 3 — Additional file 3. Methylation-methylation correlation for genes with multiple regulatory circuits. Matrixes presenting the methylation versus methylation correlation (R) between two of the associated sites. Genomic locations of the associated sites are given to the left, the sites chosen by best prediction models (Fig. 6) are highlighted. Genomic maps of the associated sites (red bars), units (letters) and genes (purple) are given as well. [file 13059_2023_3094_MOESM3_ESM.pdf]

ABL1

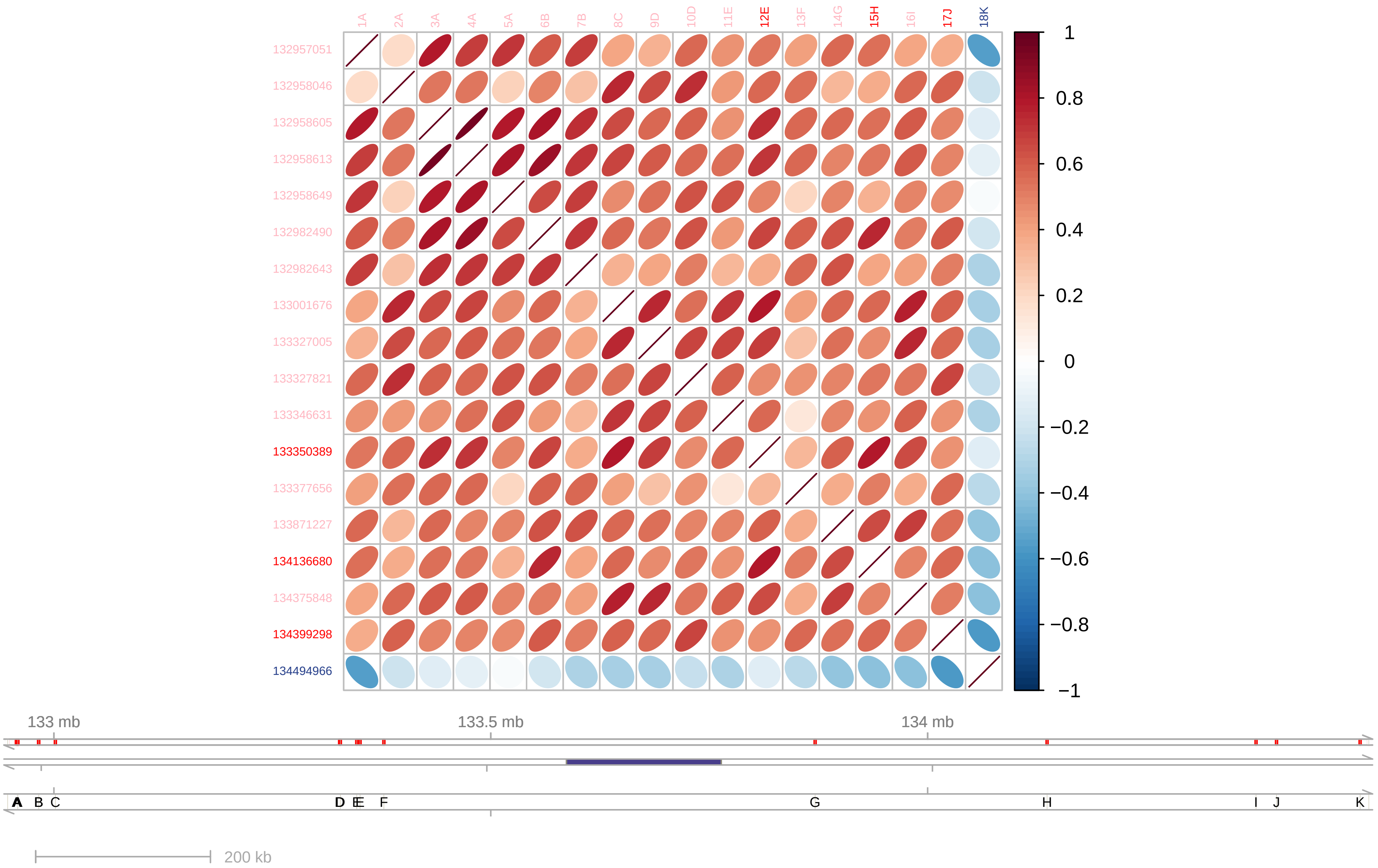

1A
2A
3A
4A
5A
6B
7B
8C
9D
10D
11E
12E
13F
14G
15H
16I
17J
18K
132957051
132958046
132958605
132958613
132958649
132982490
132982643
133001676
133327005
133327821
133346631
133350389
133377656
133871227
134136680
134375848
134399298
134494966
1
0.8
0.6
0.4
0.2
0
−0.2
−0.4
−0.6
−0.8
−1
133 mb
133.5 mb
134 mb
A B C
D E E F
G
H
I J
K
200 kb

ACVR1B

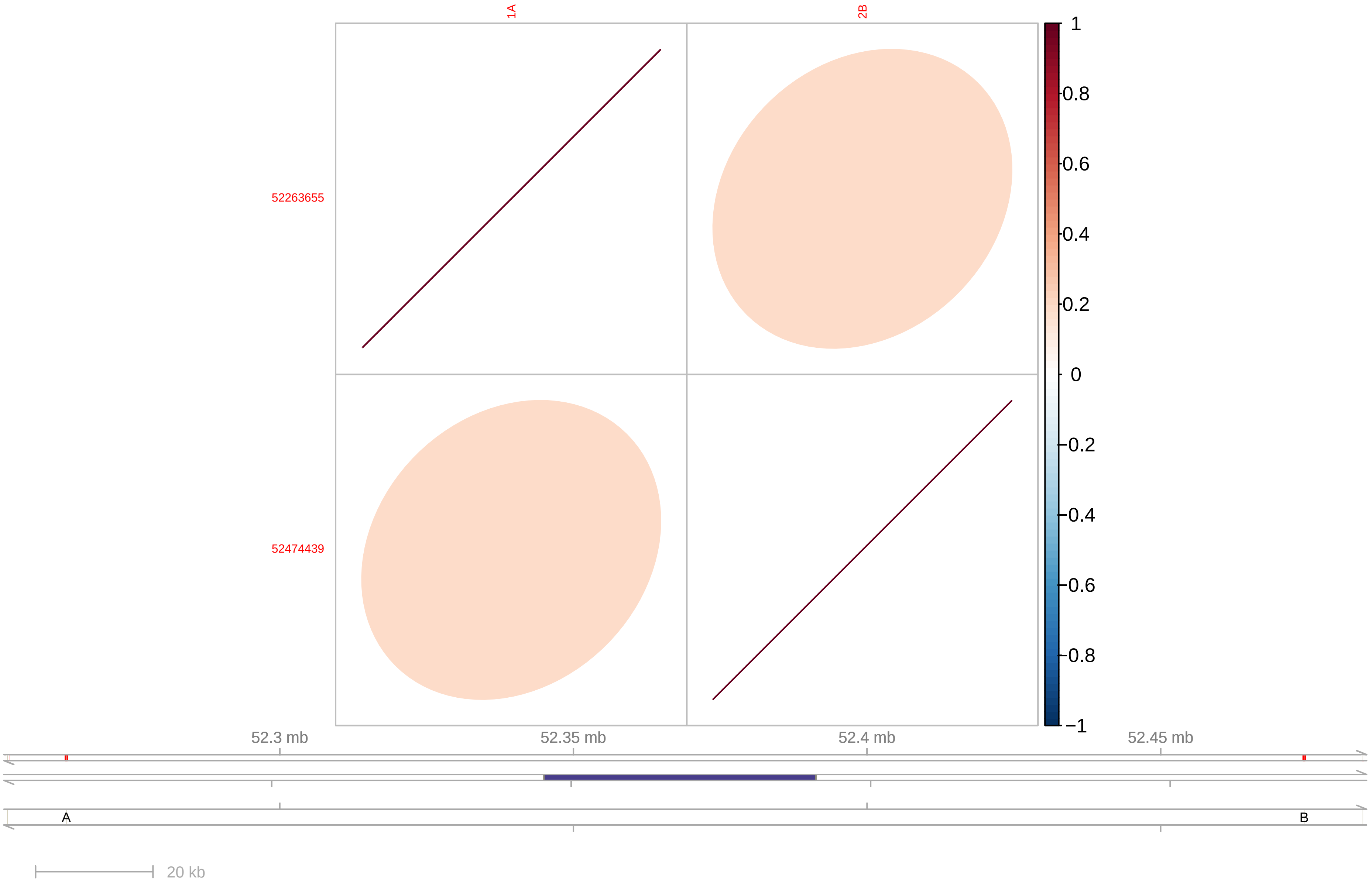

1A
2B
52263655
52474439
1
0.8
0.6
0.4
0.2
0
−0.2
−0.4
−0.6
−0.8
−1
52.3 mb
52.35 mb
52.4 mb
52.45 mb
A
B
20 kb

AKT1

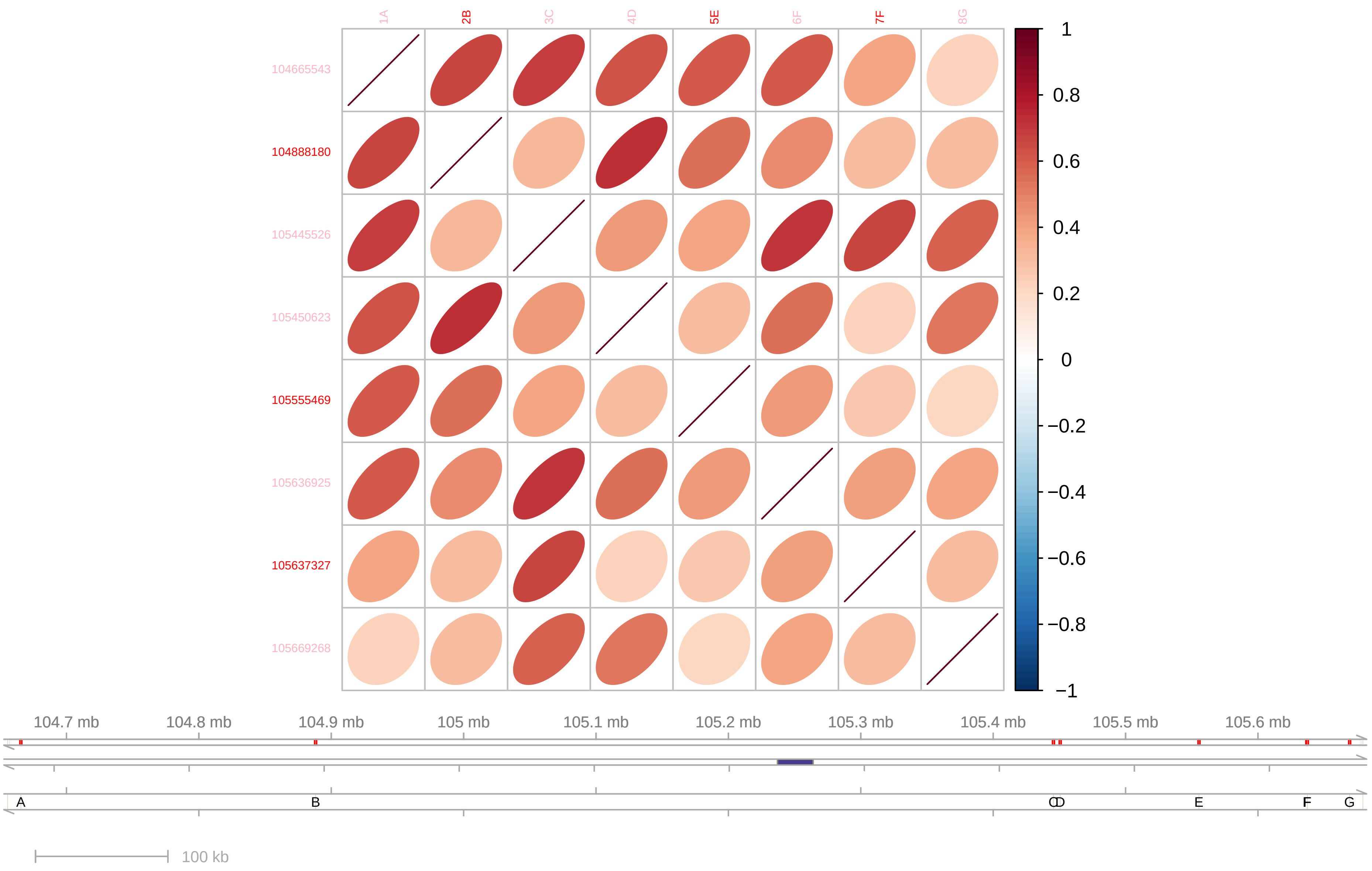

1A
2B
3C
4D
5E
6F
7F
8G
104665543
104888180
105445526
105450623
105555469
105636925
105637327
105669268
1
0.8
0.6
0.4
0.2
0
−0.2
−0.4
−0.6
−0.8
−1
104.7 mb
104.8 mb
104.9 mb
105 mb
105.1 mb
105.2 mb
105.3 mb
105.4 mb
105.5 mb
105.6 mb
A
B
CD
E
F
G
100 kb

AKT2

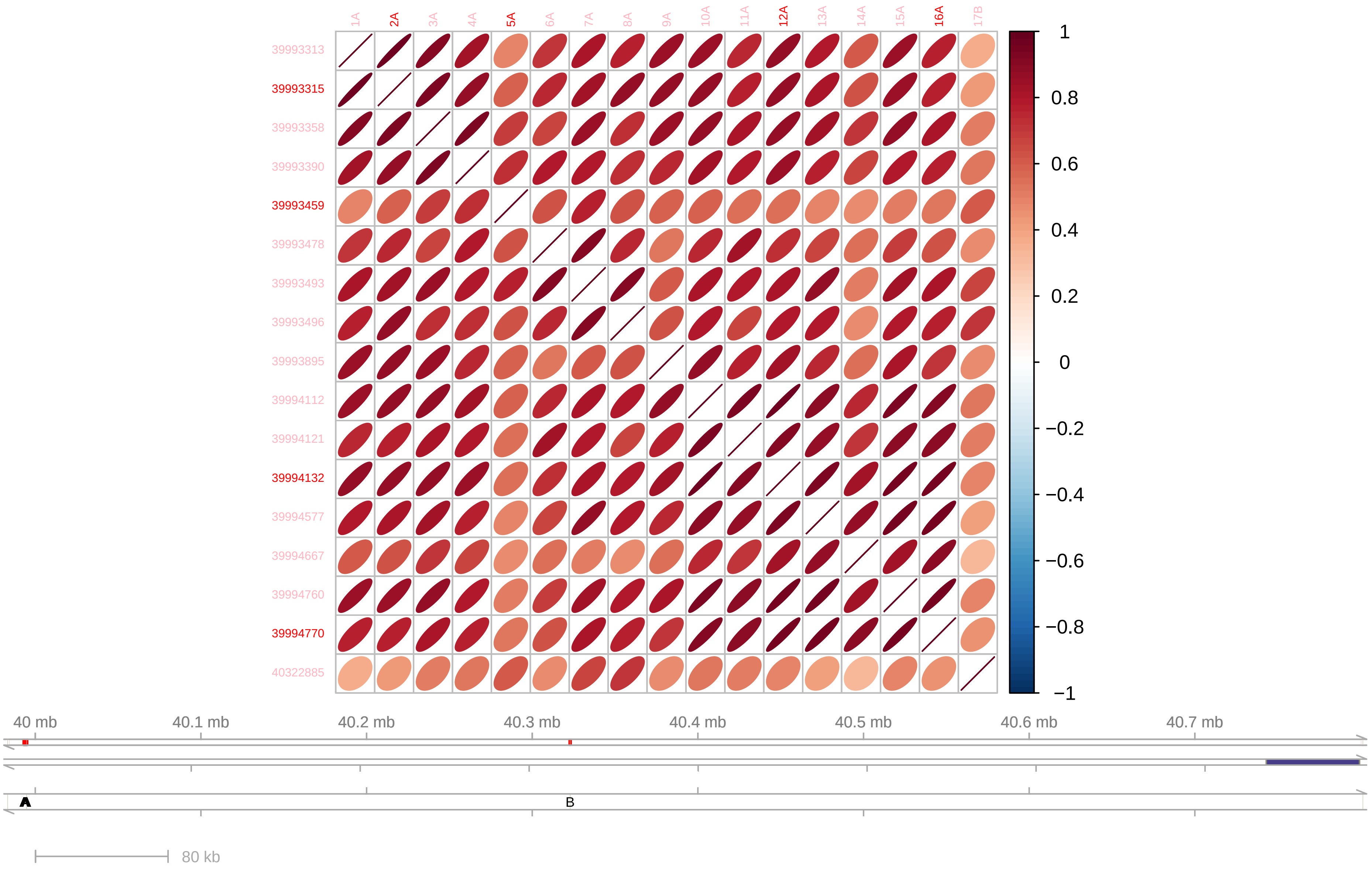

1A
2A
3A
4A
5A
6A
7A
8A
9A
10A
11A
12A
13A
14A
15A
16A
17B
39993313
39993315
39993358
39993390
39993459
39993478
39993493
39993496
39993895
39994112
39994121
39994132
39994577
39994667
39994760
39994770
40322885
1
0.8
0.6
0.4
0.2
0
−0.2
−0.4
−0.6
−0.8
−1
40 mb
40.1 mb
40.2 mb
40.3 mb
40.4 mb
40.5 mb
40.6 mb
40.7 mb
A
B
80 kb

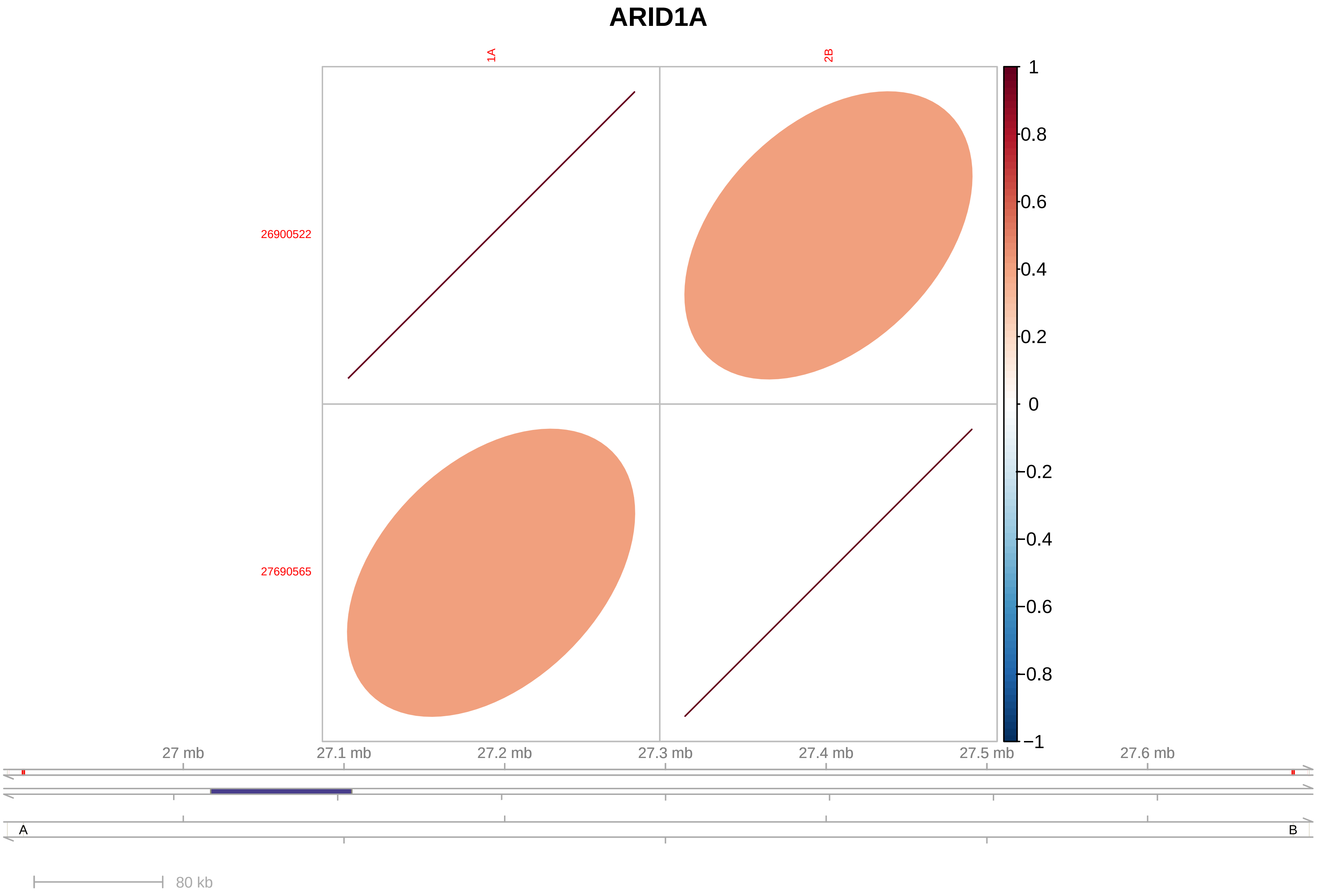

ARID1A
1A
2B
26900522
27690565
1
0.8
0.6
0.4
0.2
0
−0.2
−0.4
−0.6
−0.8
−1
27 mb
27.1 mb
27.2 mb
27.3 mb
27.4 mb
27.5 mb
27.6 mb
A
B
80 kb

ASXL1

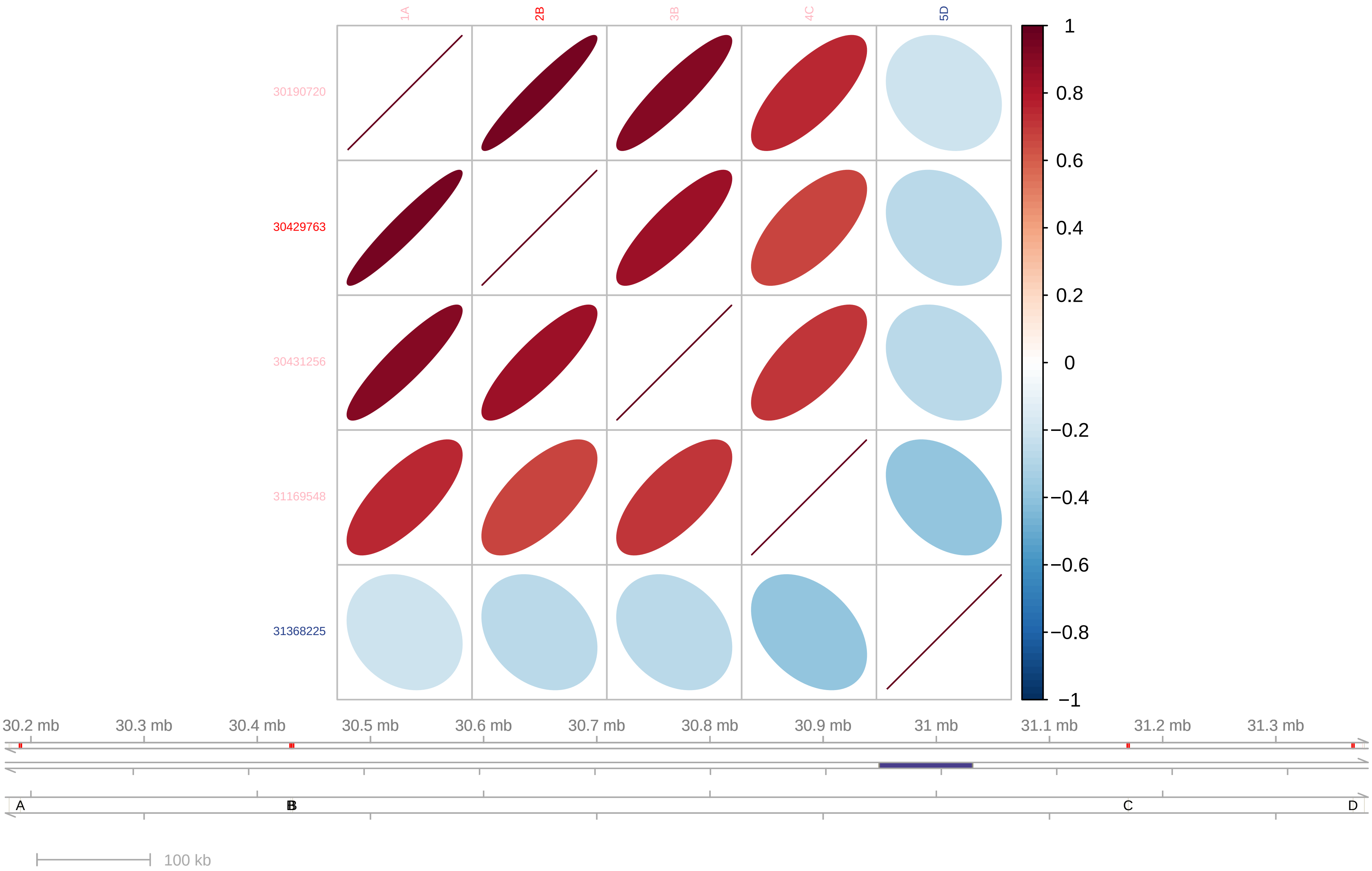

1A
2B
3B
4C
5D
30190720
30429763
30431256
31169548
31368225
1
0.8
0.6
0.4
0.2
0
−0.2
−0.4
−0.6
−0.8
−1
30.2 mb
30.3 mb
30.4 mb
30.5 mb
30.6 mb
30.7 mb
30.8 mb
30.9 mb
31 mb
31.1 mb
31.2 mb
31.3 mb
A
B
C
D
100 kb

AXIN1

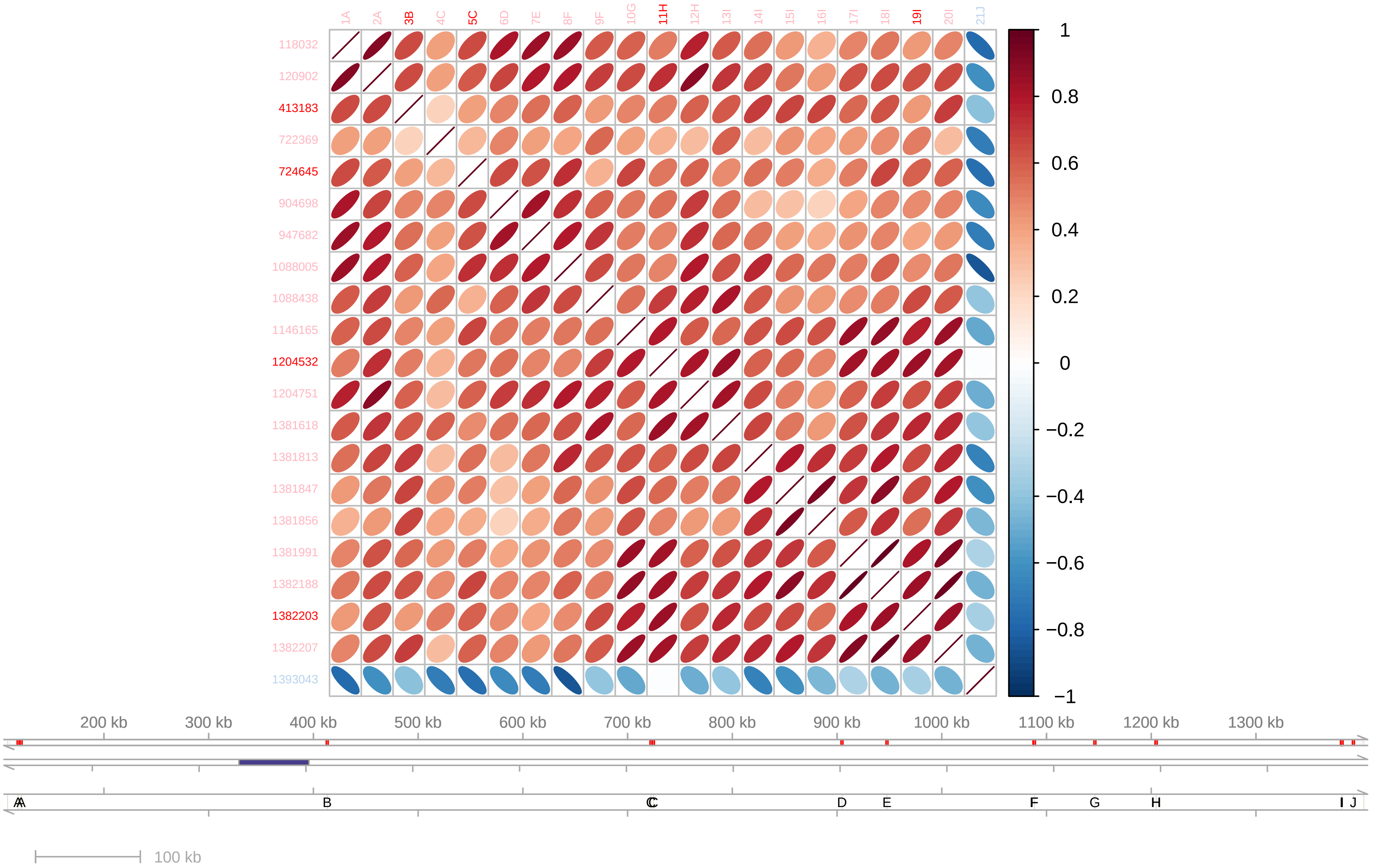

1A
2A
3B
4C
5C
6D
7E
8F
9F
10G
11H
12H
13I
14I
15I
16I
17I
18I
19I
20I
21J
118032
120902
413183
722369
724645
904698
947682
1088005
1088438
1146165
1204532
1204751
1381618
1381813
1381847
1381856
1381991
1382188
1382203
1382207
1393043
1
0.8
0.6
0.4
0.2
0
−0.2
−0.4
−0.6
−0.8
−1
200 kb
300 kb
400 kb
500 kb
600 kb
700 kb
800 kb
900 kb
1000 kb
1100 kb
1200 kb
1300 kb
A
B
C
D
E
F
G
H
I
J
100 kb

BCOR

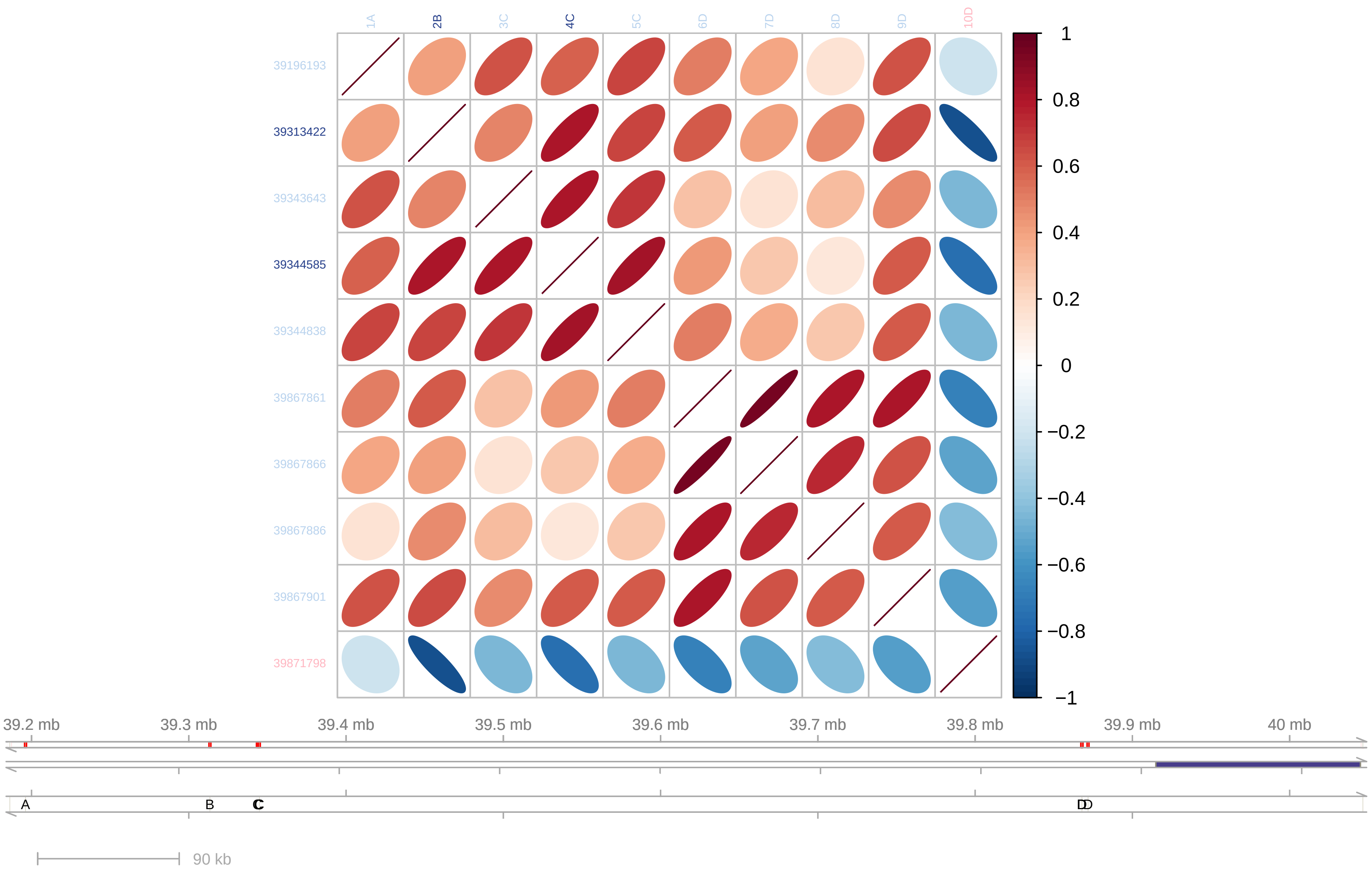

1A
2B
3C
4C
5C
6D
7D
8D
9D
10D
39196193
39313422
39343643
39344585
39344838
39867861
39867866
39867886
39867901
39871798
1
0.8
0.6
0.4
0.2
0
−0.2
−0.4
−0.6
−0.8
−1
39.2 mb
39.3 mb
39.4 mb
39.5 mb
39.6 mb
39.7 mb
39.8 mb
39.9 mb
40 mb
A
B
C
D
90 kb

BRCA1

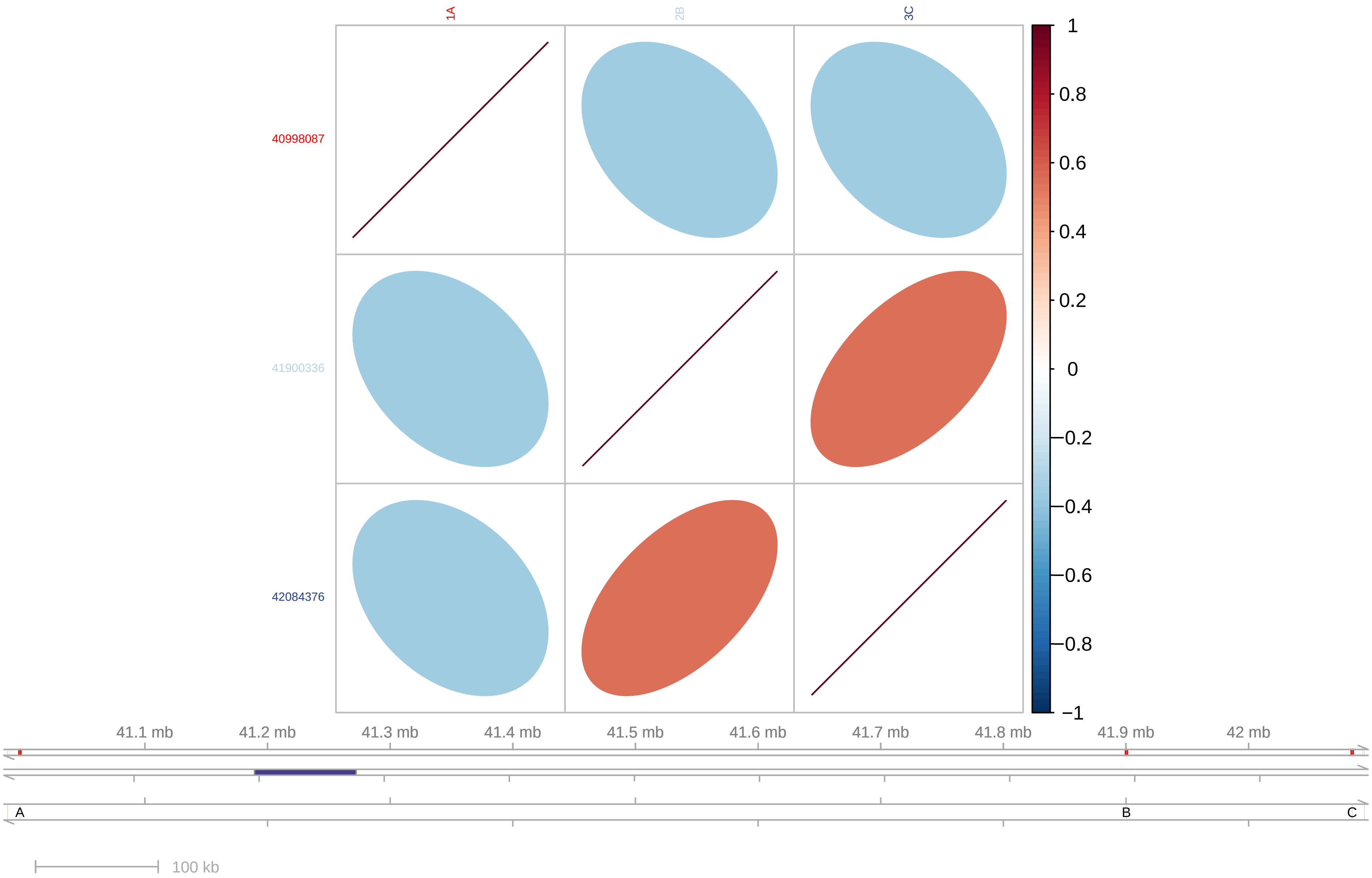

1A
2B
3C
40998087
41900336
42084376
1
0.8
0.6
0.4
0.2
0
−0.2
−0.4
−0.6
−0.8
−1
41.1 mb
41.2 mb
41.3 mb
41.4 mb
41.5 mb
41.6 mb
41.7 mb
41.8 mb
41.9 mb
42 mb
A
B
C
100 kb

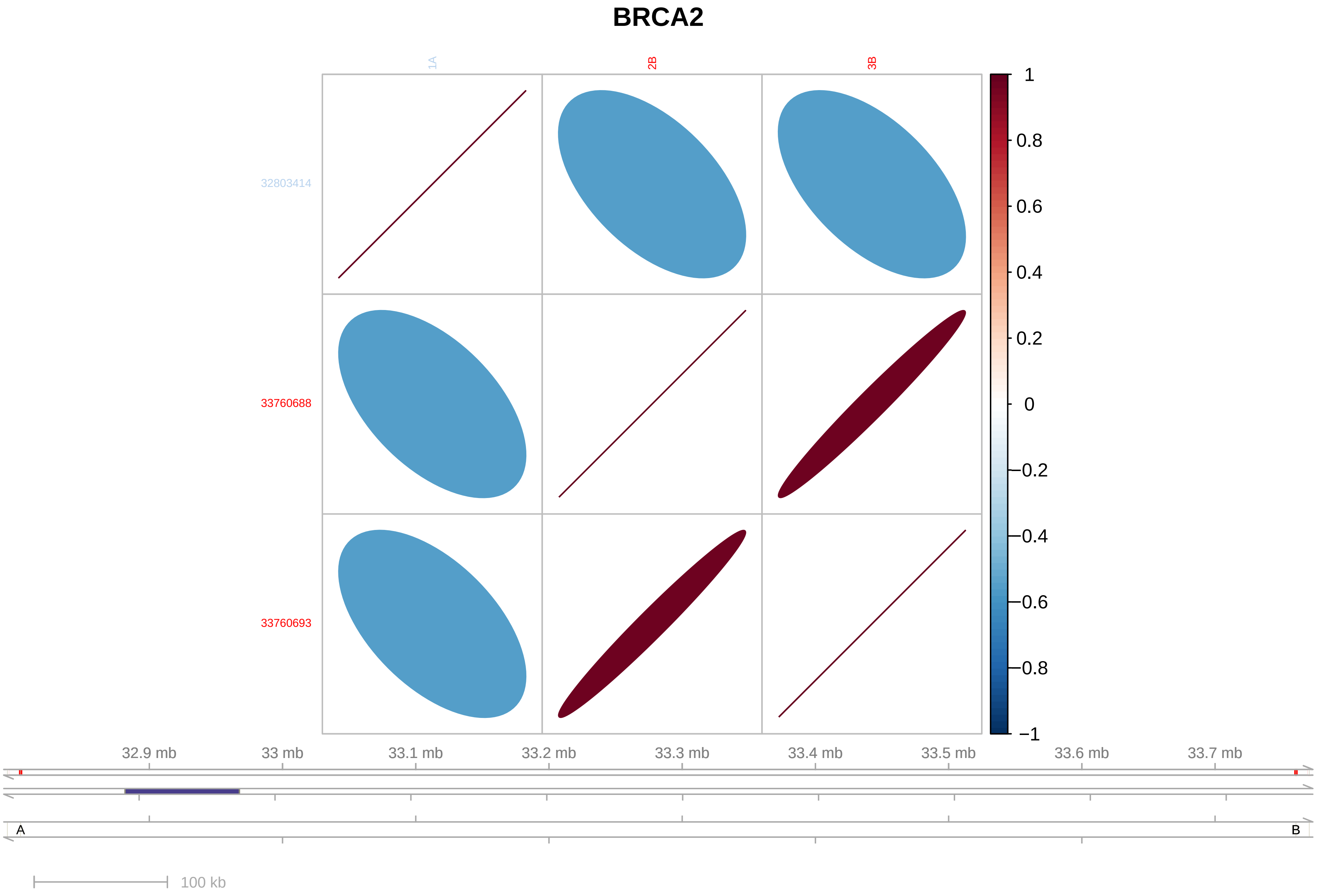

BRCA2
1A
2B
3B
32803414
33760688
33760693
1
0.8
0.6
0.4
0.2
0
−0.2
−0.4
−0.6
−0.8
−1
32.9 mb
33 mb
33.1 mb
33.2 mb
33.3 mb
33.4 mb
33.5 mb
33.6 mb
33.7 mb
A
B
100 kb

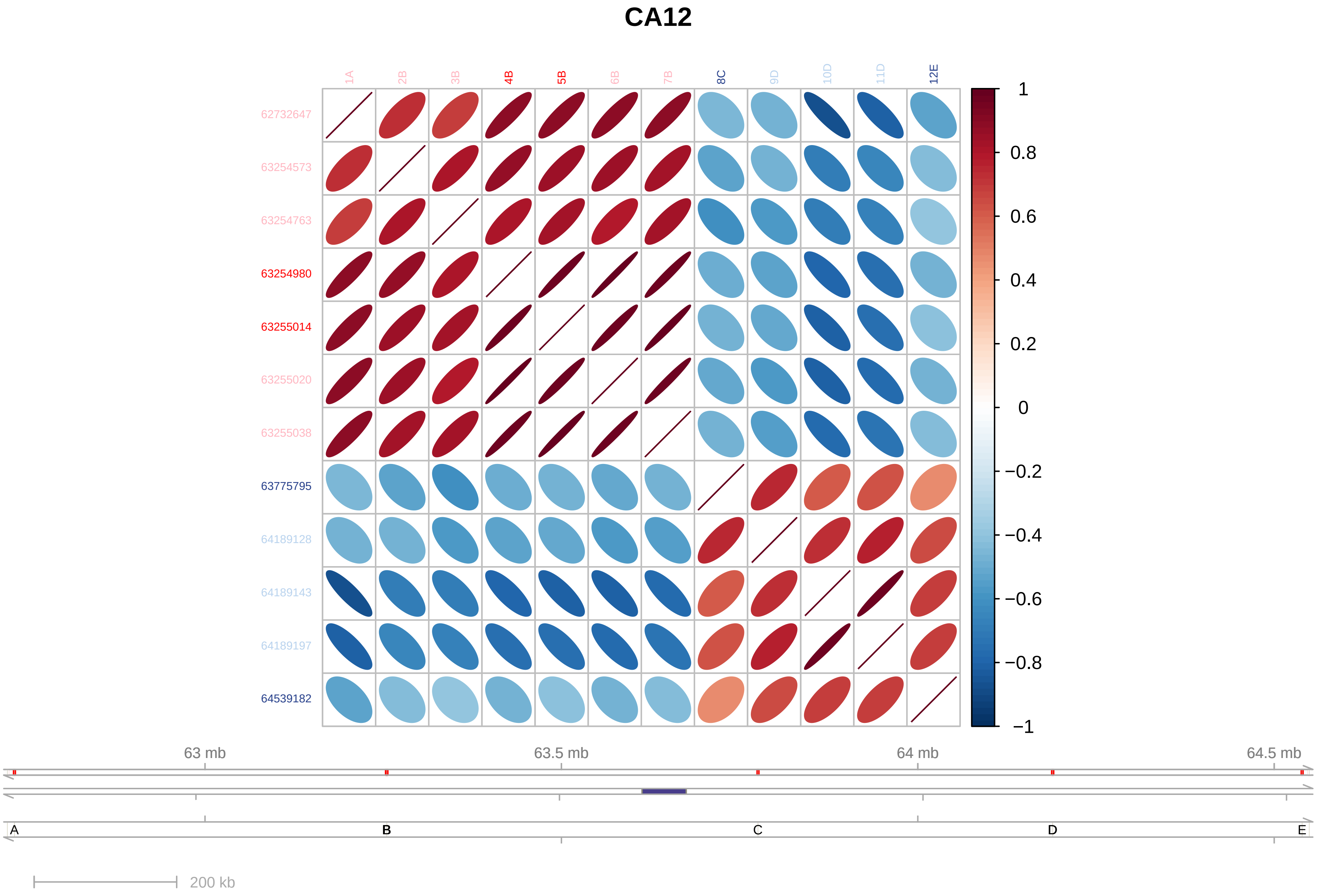
CA12
1A
2B
3B
4B
5B
6B
7B
8C
9D
10D
11D
12E
62732647
63254573
63254763
63254980
63255014
63255020
63255038
63775795
64189128
64189143
64189197
64539182
1
0.8
0.6
0.4
0.2
0
−0.2
−0.4
−0.6
−0.8
−1
63 mb
63.5 mb
64 mb
64.5 mb
A
B
C
D
E
200 kb

CD68

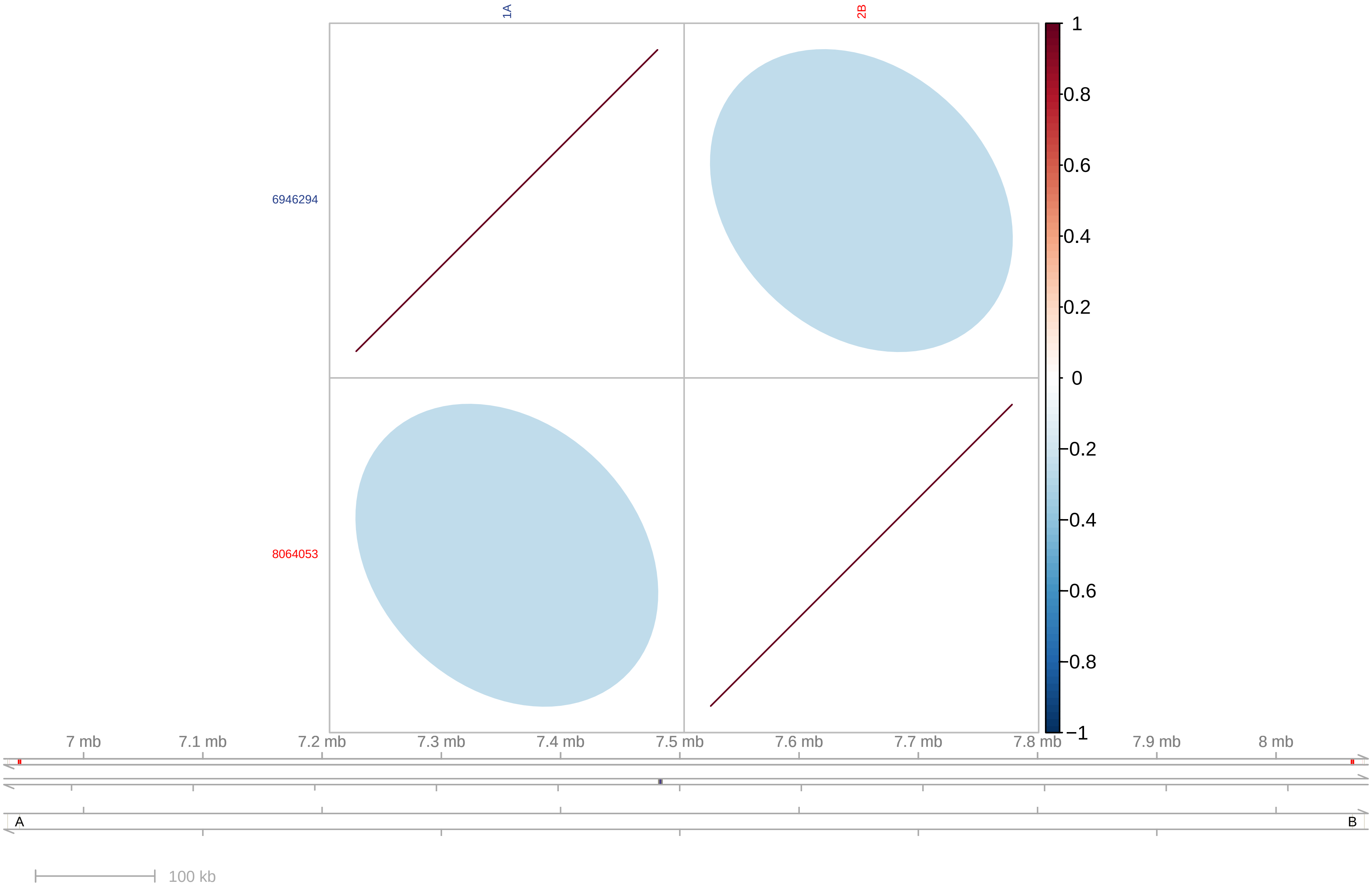

1A
2B
6946294
8064053
1
0.8
0.6
0.4
0.2
0
−0.2
−0.4
−0.6
−0.8
−1
7 mb
7.1 mb
7.2 mb
7.3 mb
7.4 mb
7.5 mb
7.6 mb
7.7 mb
7.8 mb
7.9 mb
8 mb
A
B
100 kb

CDKN2A

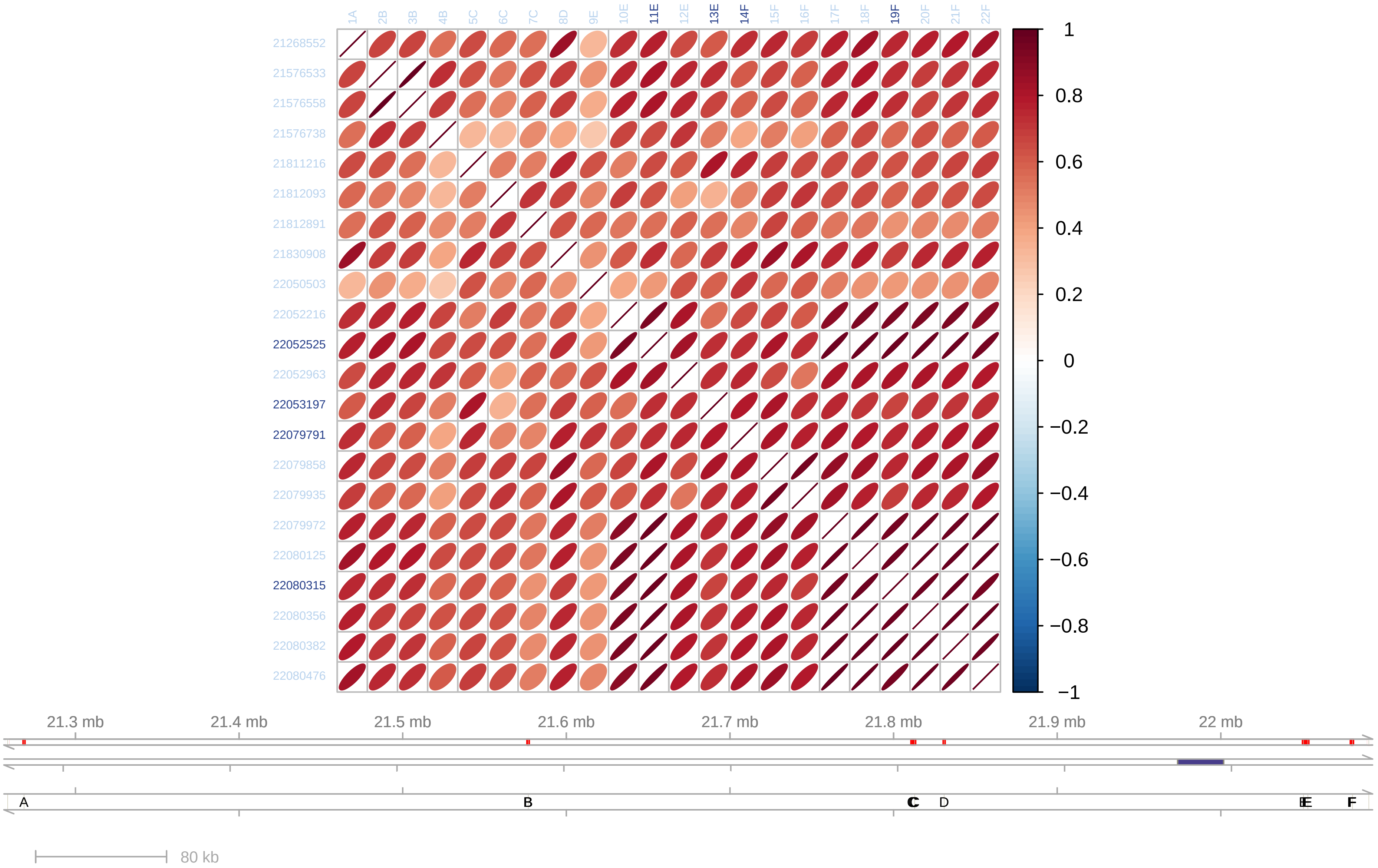
1A
2B
3B
4B
5C
6C
7C
8D
9E
10E
11E
12E
13E
14F
15F
16F
17F
18F
19F
20F
21F
22F
21268552
21576533
21576558
21576738
21811216
21812093
21812891
21830908
22050503
22052216
22052525
22052963
22053197
22079791
22079858
22079935
22079972
22080125
22080315
22080356
22080382
22080476
1
0.8
0.6
0.4
0.2
0
−0.2
−0.4
−0.6
−0.8
−1
21.3 mb
21.4 mb
21.5 mb
21.6 mb
21.7 mb
21.8 mb
21.9 mb
22 mb
A
B
C
D
E
F
80 kb

CHEK2

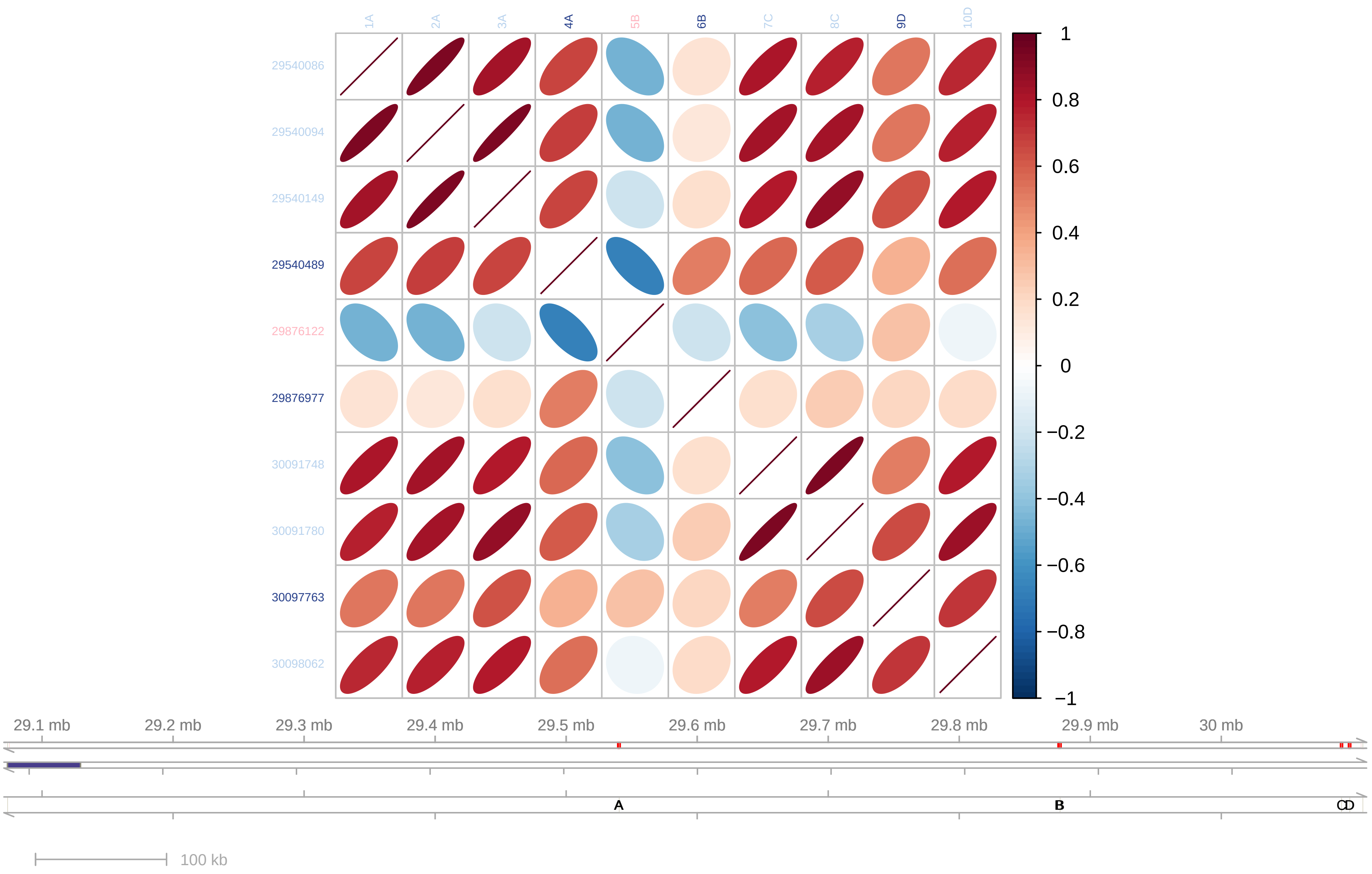

1A
2A
3A
4A
5B
6B
7C
8C
9D
10D
29540086
29540094
29540149
29540489
29876122
29876977
30091748
30091780
30097763
30098062
1
0.8
0.6
0.4
0.2
0
−0.2
−0.4
−0.6
−0.8
−1
29.1 mb
29.2 mb
29.3 mb
29.4 mb
29.5 mb
29.6 mb
29.7 mb
29.8 mb
29.9 mb
30 mb
A
B
CD
100 kb

CHI3L1

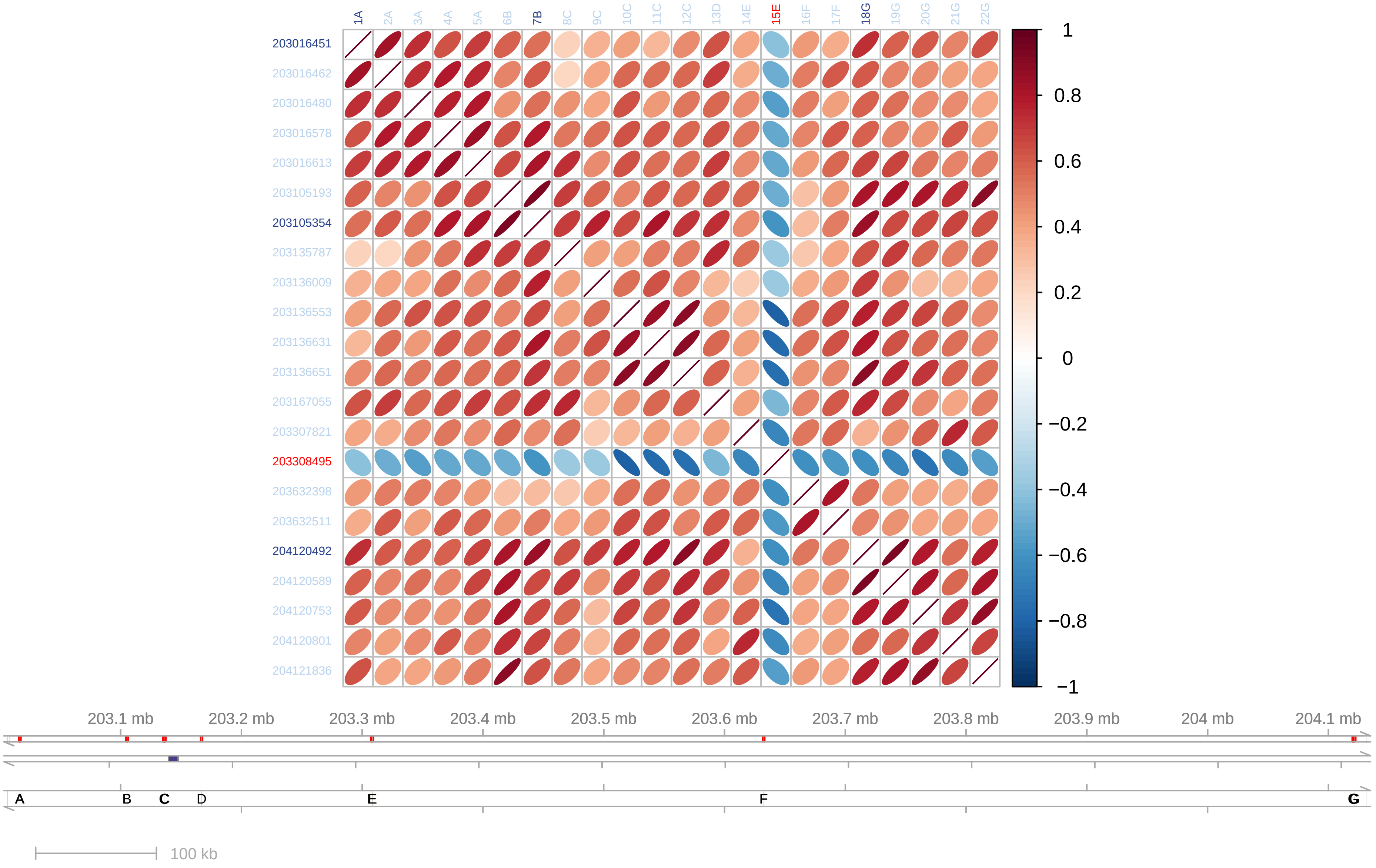

1A
2A
3A
4A
5A
6B
7B
8C
9C
10C
11C
12C
13D
14E
15E
16F
17F
18G
19G
20G
21G
22G
203016451
203016462
203016480
203016578
203016613
203105193
203105354
203135787
203136009
203136553
203136631
203136651
203167055
203307821
203308495
203632398
203632511
204120492
204120589
204120753
204120801
204121836
1
0.8
0.6
0.4
0.2
0
−0.2
−0.4
−0.6
−0.8
−1
203.1 mb
203.2 mb
203.3 mb
203.4 mb
203.5 mb
203.6 mb
203.7 mb
203.8 mb
203.9 mb
204 mb
204.1 mb
A
B
C
D
E
F
G
100 kb

CIC

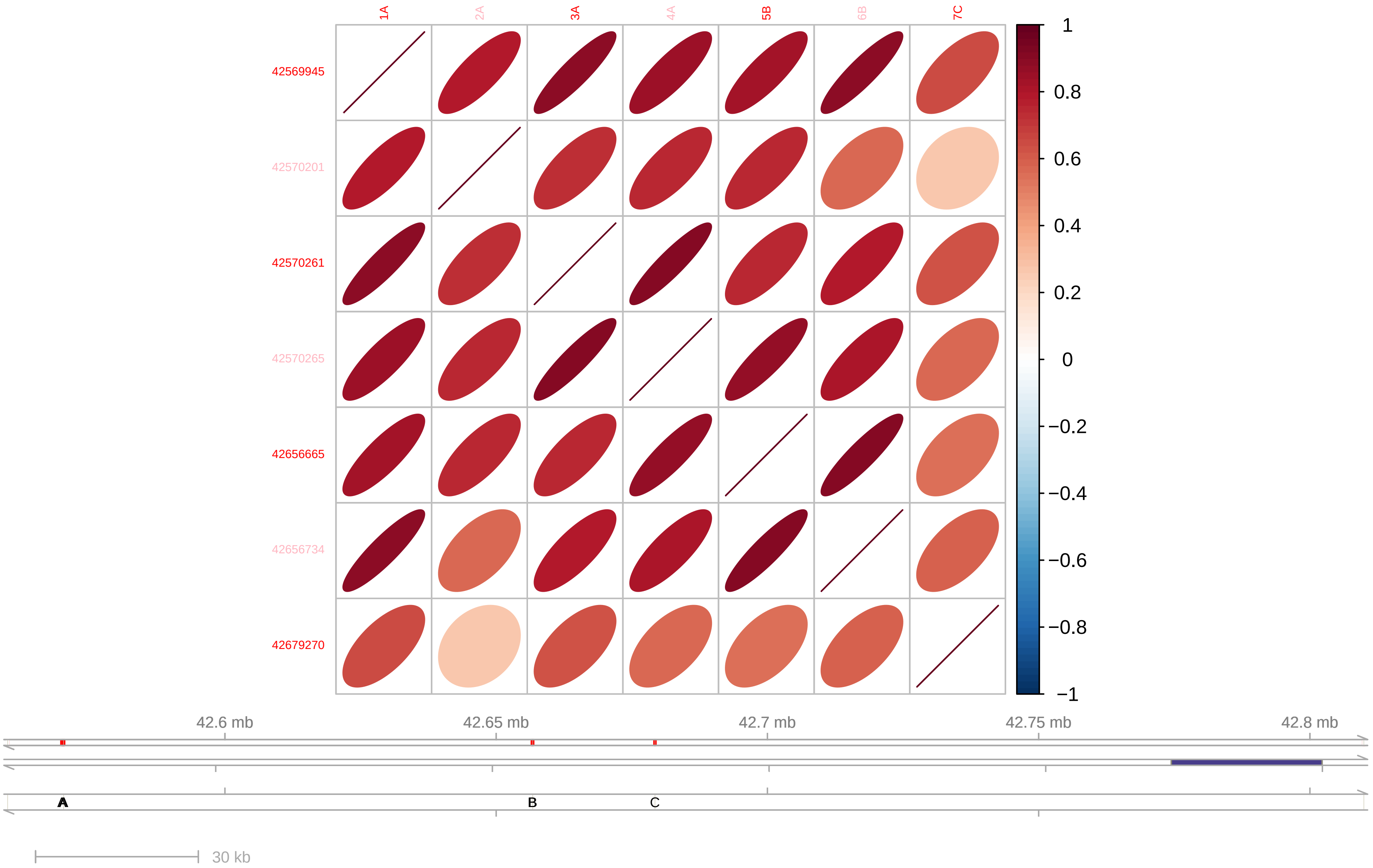

1A
2A
3A
4A
5B
6B
7C
42569945
42570201
42570261
42570265
42656665
42656734
42679270
1
0.8
0.6
0.4
0.2
0
−0.2
−0.4
−0.6
−0.8
−1
42.6 mb
42.65 mb
42.7 mb
42.75 mb
42.8 mb
A
B
C
30 kb

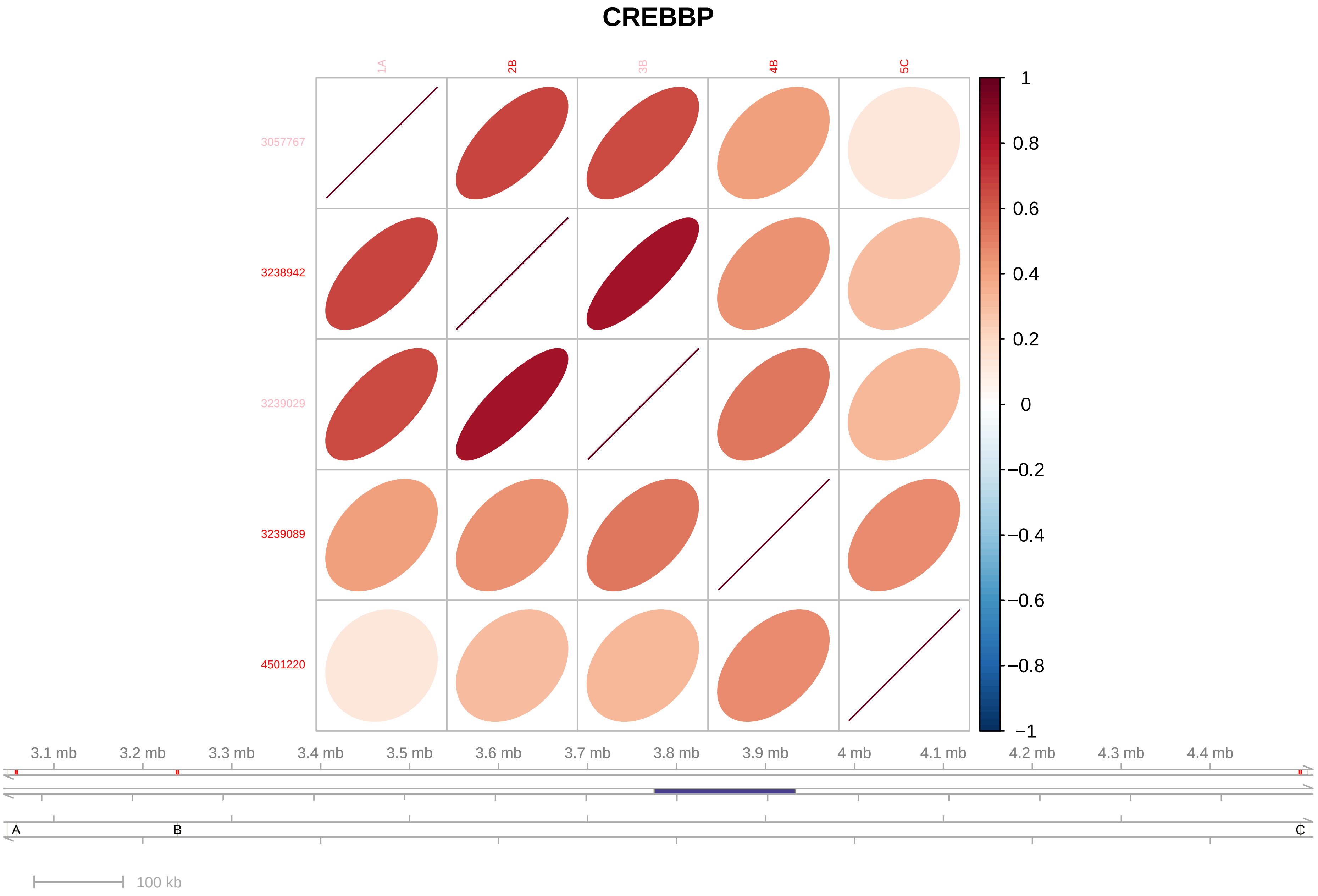

CREBBP
1A
2B
3B
4B
5C
3057767
3238942
3239029
3239089
4501220
1
0.8
0.6
0.4
0.2
0
−0.2
−0.4
−0.6
−0.8
−1
3.1 mb
3.2 mb
3.3 mb
3.4 mb
3.5 mb
3.6 mb
3.7 mb
3.8 mb
3.9 mb
4 mb
4.1 mb
4.2 mb
4.3 mb
4.4 mb
A
B
C
100 kb

CTNNB1

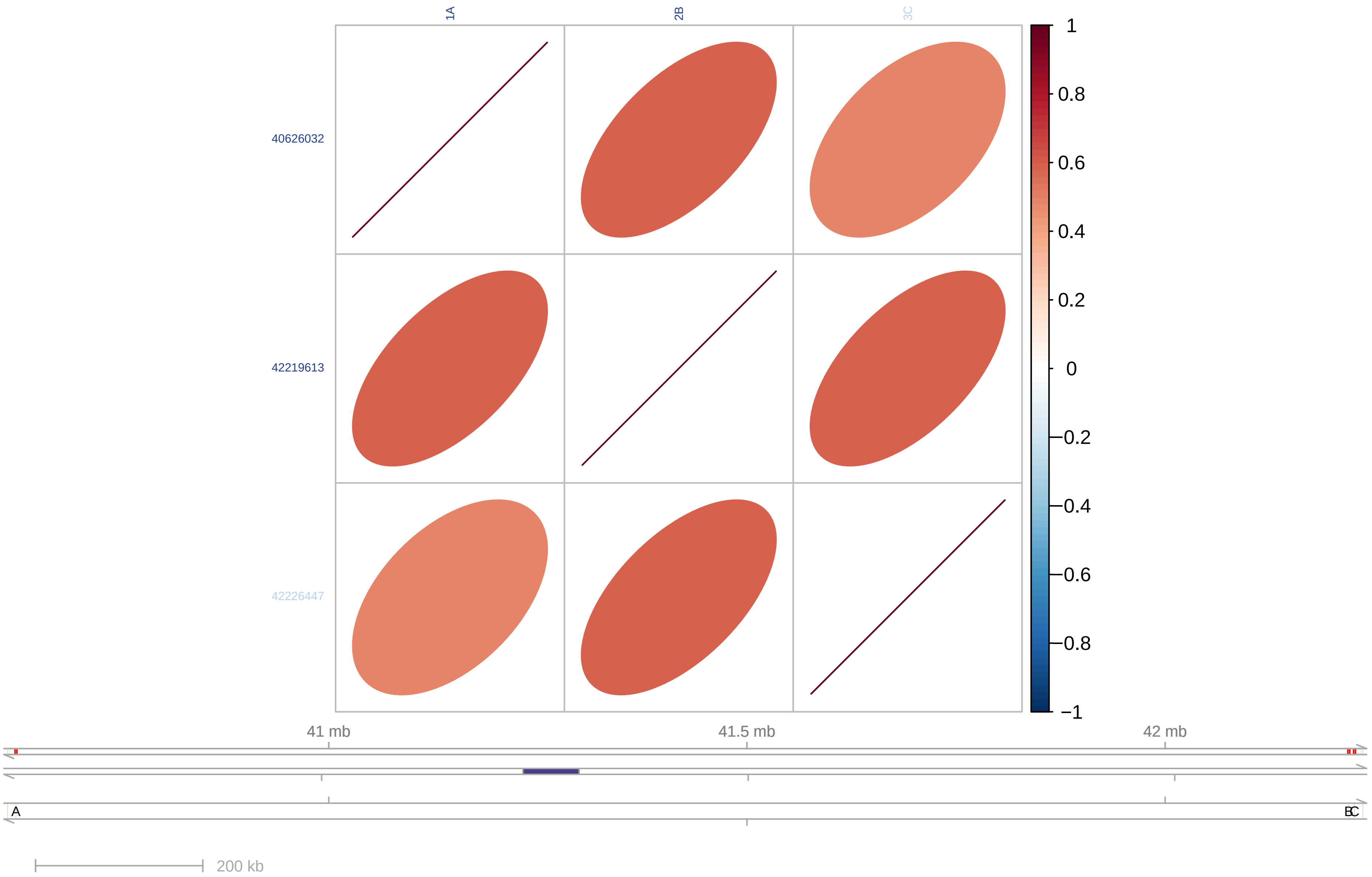

1A
2B
3C
40626032
42219613
42226447
1
0.8
0.6
0.4
0.2
0
−0.2
−0.4
−0.6
−0.8
−1
41 mb
41.5 mb
42 mb
A
BC
200 kb

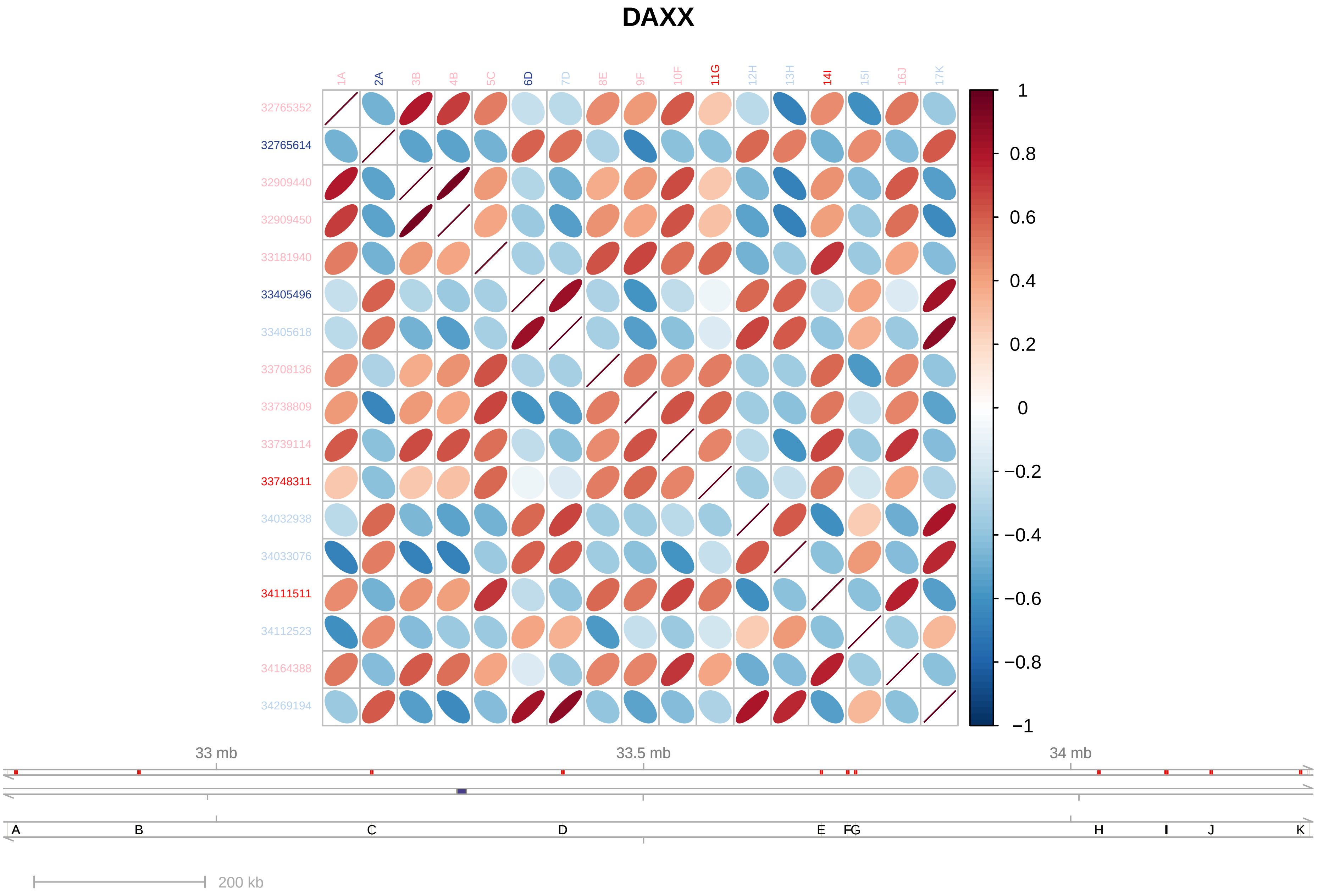
DAXX
1A
2A
3B
4B
5C
6D
7D
8E
9F
10F
11G
12H
13H
14I
15I
16J
17K
32765352
32765614
32909440
32909450
33181940
33405496
33405618
33708136
33738809
33739114
33748311
34032938
34033076
34111511
34112523
34164388
34269194
1
0.8
0.6
0.4
0.2
0
−0.2
−0.4
−0.6
−0.8
−1
33 mb
33.5 mb
34 mb
A
B
C
D
E
FG
H
I
J
K
200 kb

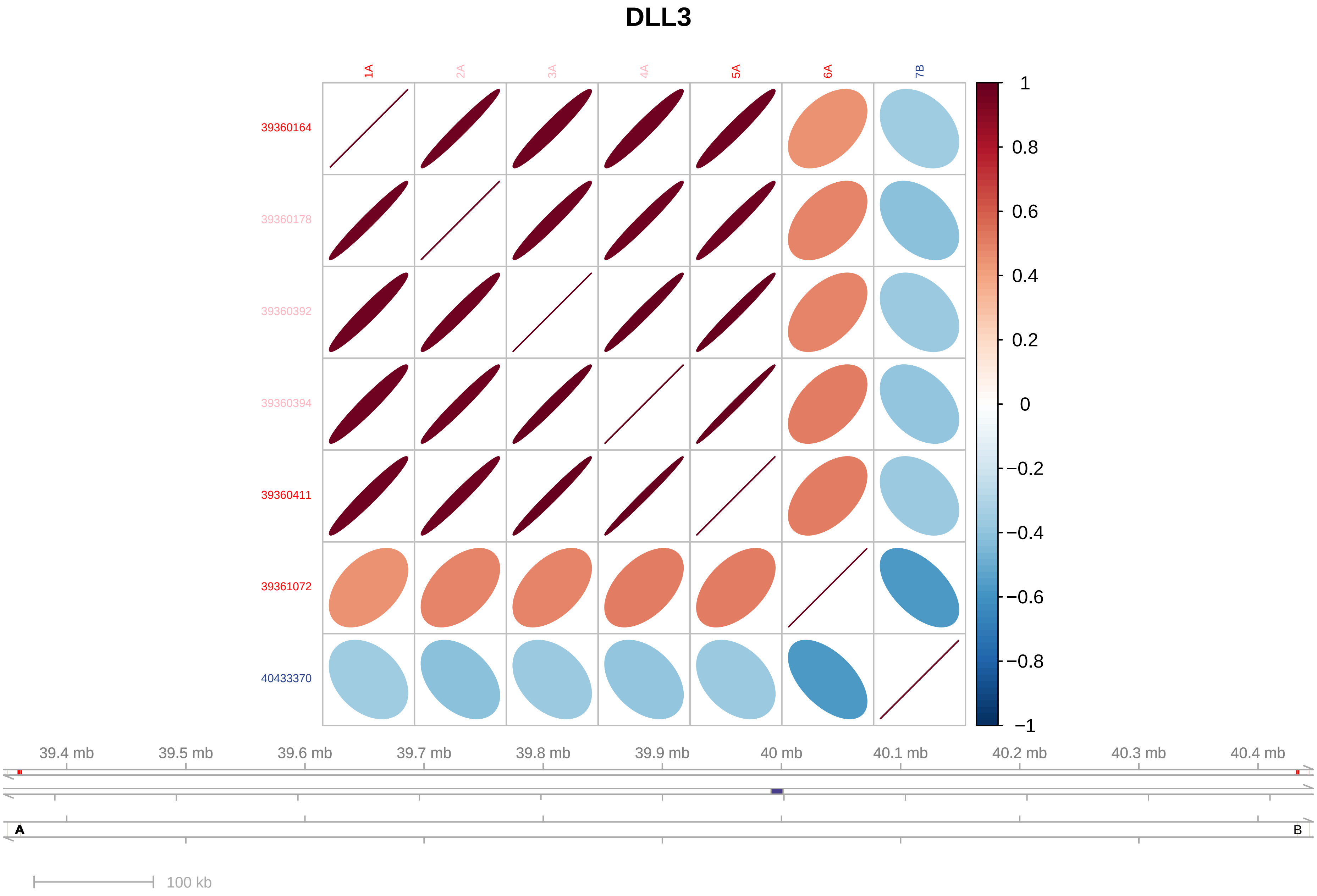

DLL3
1A
2A
3A
4A
5A
6A
7B
39360164
39360178
39360392
39360394
39360411
39361072
40433370
1
0.8
0.6
0.4
0.2
0
−0.2
−0.4
−0.6
−0.8
−1
39.4 mb
39.5 mb
39.6 mb
39.7 mb
39.8 mb
39.9 mb
40 mb
40.1 mb
40.2 mb
40.3 mb
40.4 mb
A
B
100 kb

DNMT3A

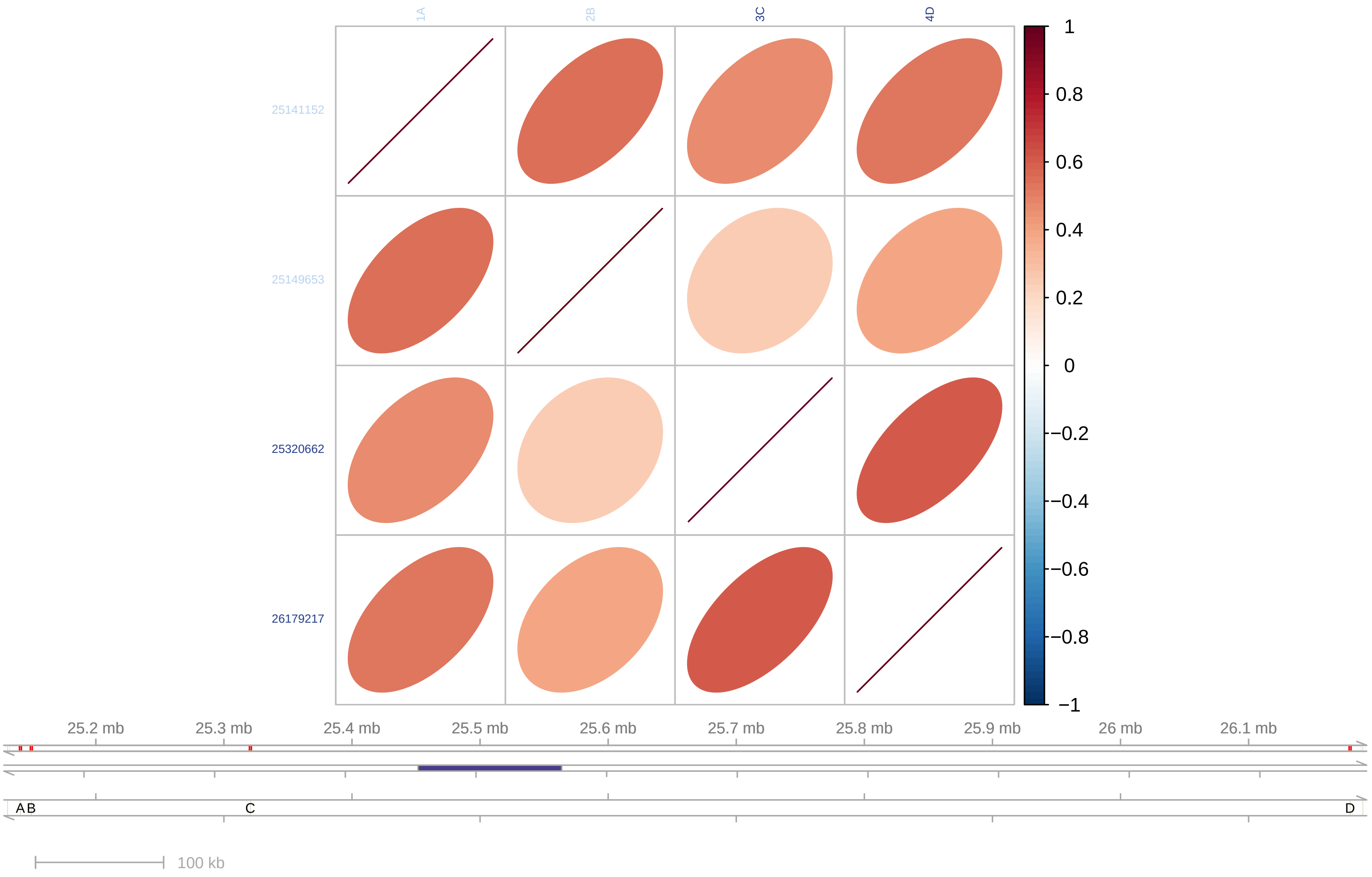
1A
2B
3C
4D
25141152
25149653
25320662
26179217
1
0.8
0.6
0.4
0.2
0
−0.2
−0.4
−0.6
−0.8
−1
25.2 mb
25.3 mb
25.4 mb
25.5 mb
25.6 mb
25.7 mb
25.8 mb
25.9 mb
26 mb
26.1 mb
A B
C
D
100 kb

DSCAML1

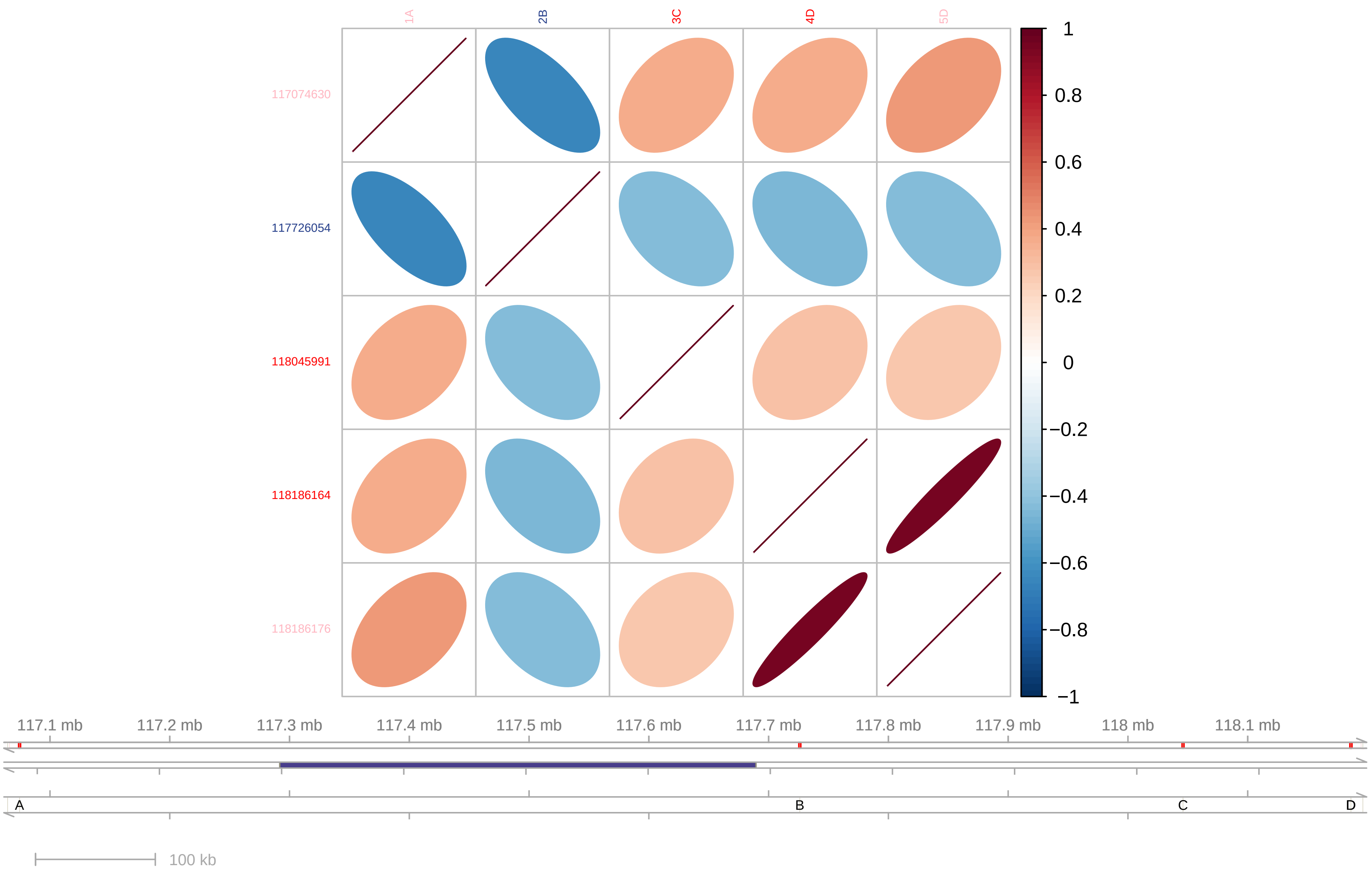

1A
2B
3C
4D
5D
117074630
117726054
118045991
118186164
118186176
1
0.8
0.6
0.4
0.2
0
−0.2
−0.4
−0.6
−0.8
−1
117.1 mb
117.2 mb
117.3 mb
117.4 mb
117.5 mb
117.6 mb
117.7 mb
117.8 mb
117.9 mb
118 mb
118.1 mb
A
B
C
D
100 kb

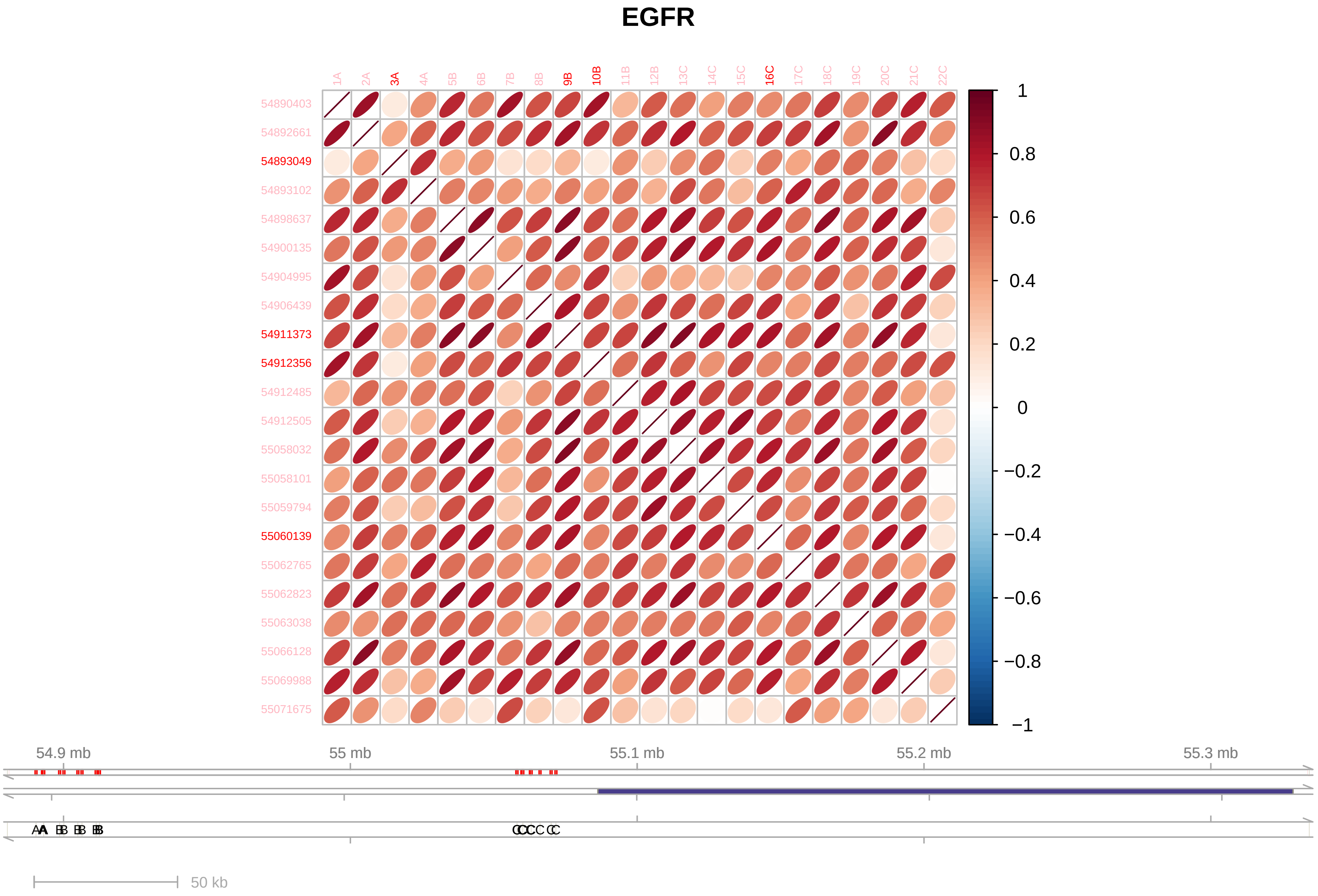
EGFR
1A
2A
3A
4A
5B
6B
7B
8B
9B
10B
11B
12B
13C
14C
15C
16C
17C
18C
19C
20C
21C
22C
54890403
54892661
54893049
54893102
54898637
54900135
54904995
54906439
54911373
54912356
54912485
54912505
55058032
55058101
55059794
55060139
55062765
55062823
55063038
55066128
55069988
55071675
1
0.8
0.6
0.4
0.2
0
−0.2
−0.4
−0.6
−0.8
−1
54.9 mb
55 mb
55.1 mb
55.2 mb
55.3 mb
50 kb

EN1

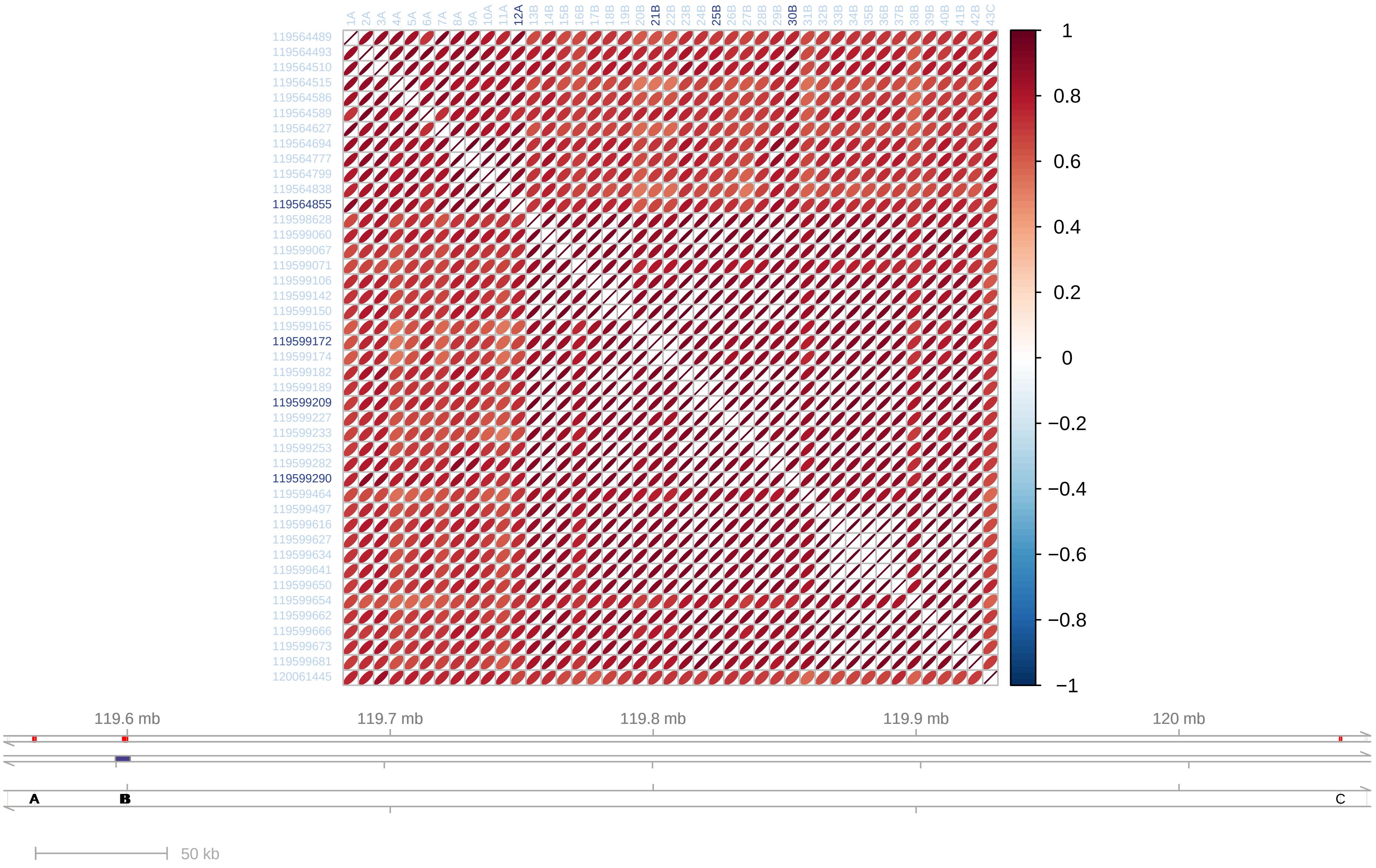
1A
2A
3A
4A
5A
6A
7A
8A
9A
10A
11A
12A
13B
14B
15B
16B
17B
18B
19B
20B
21B
22B
23B
24B
25B
26B
27B
28B
29B
30B
31B
32B
33B
34B
35B
36B
37B
38B
39B
40B
41B
42B
43C
119564489
119564493
119564510
119564515
119564586
119564589
119564627
119564694
119564777
119564799
119564838
119564855
119598628
119599060
119599067
119599071
119599106
119599142
119599150
119599165
119599172
119599174
119599182
119599189
119599209
119599227
119599233
119599253
119599282
119599290
119599464
119599497
119599616
119599627
119599634
119599641
119599650
119599654
119599662
119599666
119599673
119599681
120061445
1
0.8
0.6
0.4
0.2
0
−0.2
−0.4
−0.6
−0.8
−1
119.6 mb
119.7 mb
119.8 mb
119.9 mb
120 mb
A
B
C
50 kb

EP300

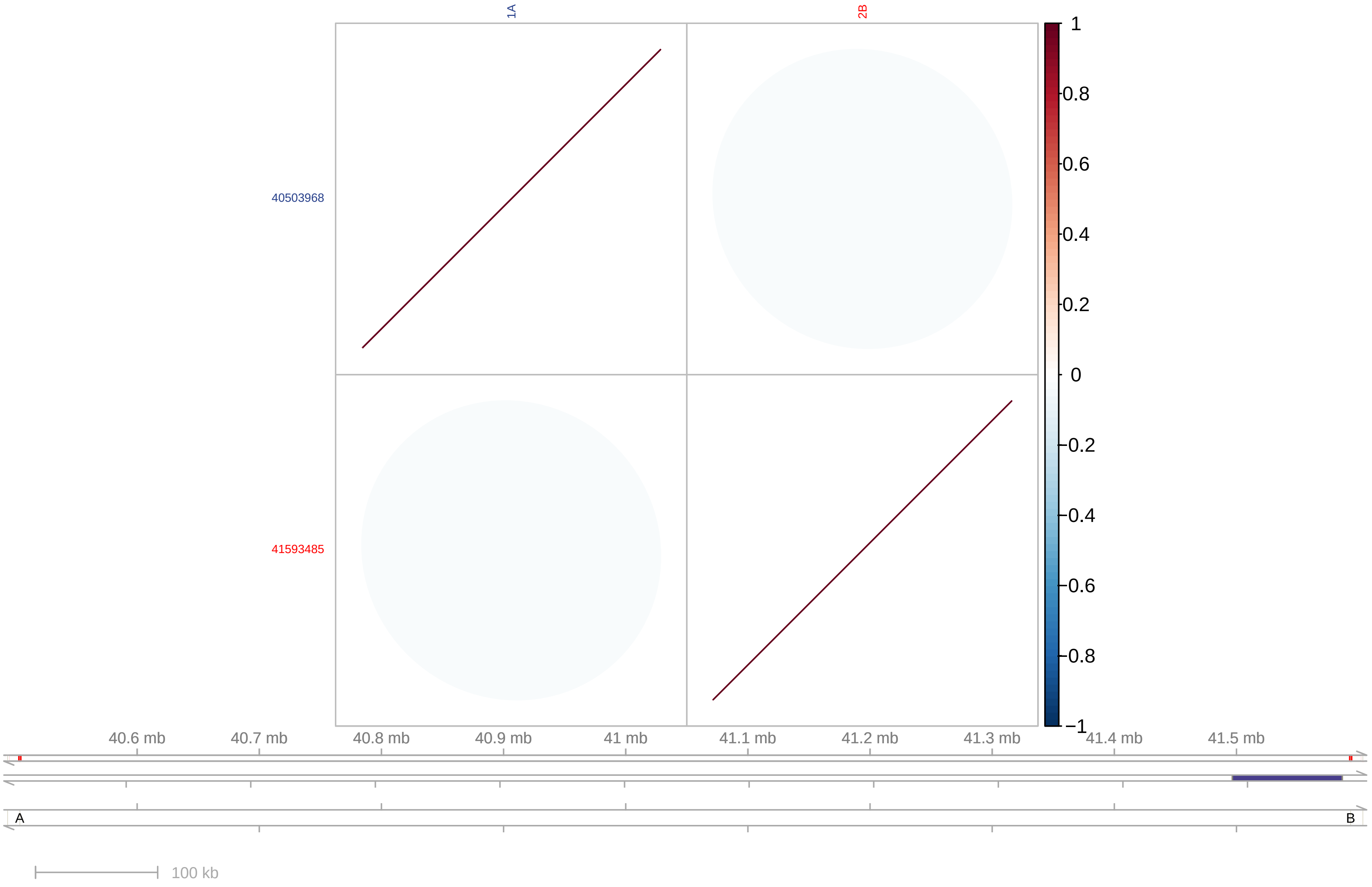

1A
2B
40503968
41593485
1
0.8
0.6
0.4
0.2
0
−0.2
−0.4
−0.6
−0.8
−1
40.6 mb
40.7 mb
40.8 mb
40.9 mb
41 mb
41.1 mb
41.2 mb
41.3 mb
41.4 mb
41.5 mb
A
B
100 kb

ERBB2

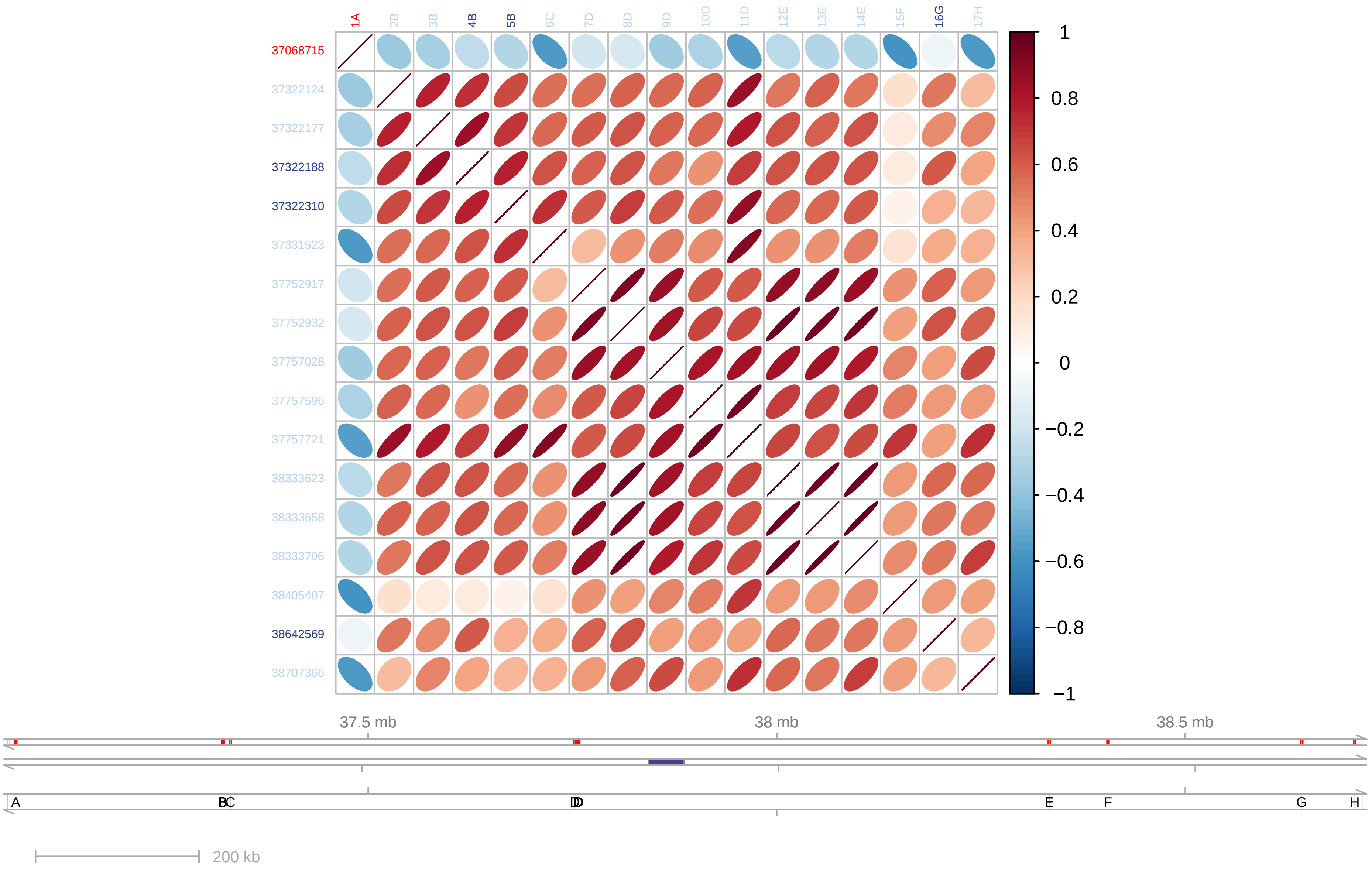

1A 2B 3B 4B 5B 6C 7D 8D 9D 10D 11D 12E 13E 14E 15F 16G 17H
37068715
37322124
37322177
37322188
37322310
37331523
37752917
37752932
37757028
37757596
37757721
38333623
38333658
38333706
38405407
38642569
38707366
1
0.8
0.6
0.4
0.2
0
−0.2
−0.4
−0.6
−0.8
−1
37.5 mb
38 mb
38.5 mb
A
BC
D
E
F
G
H
200 kb

ERBB3

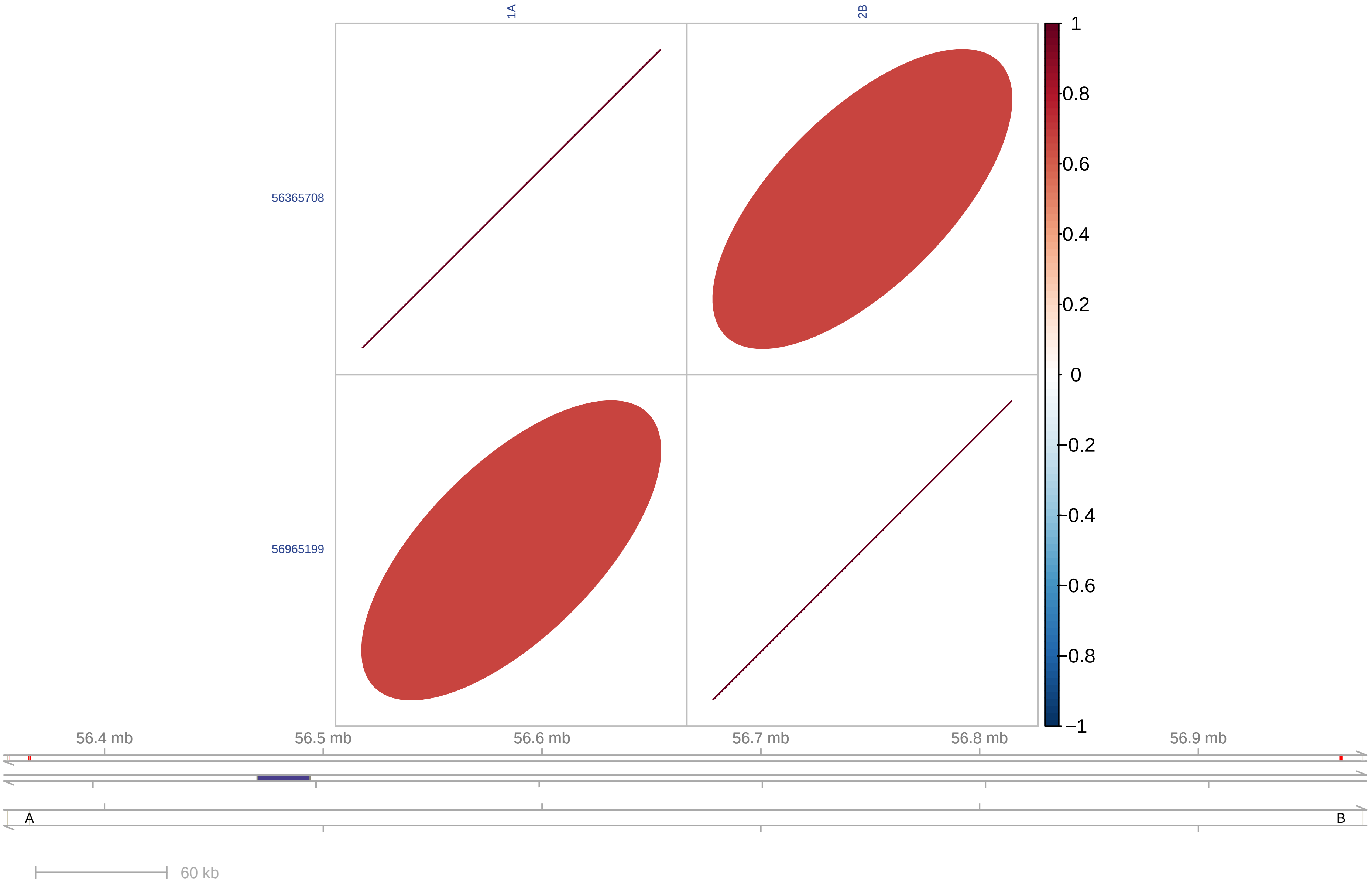
1A
2B
56365708
56965199
1
0.8
0.6
0.4
0.2
0
−0.2
−0.4
−0.6
−0.8
−1
56.4 mb
56.5 mb
56.6 mb
56.7 mb
56.8 mb
56.9 mb
A
B
60 kb

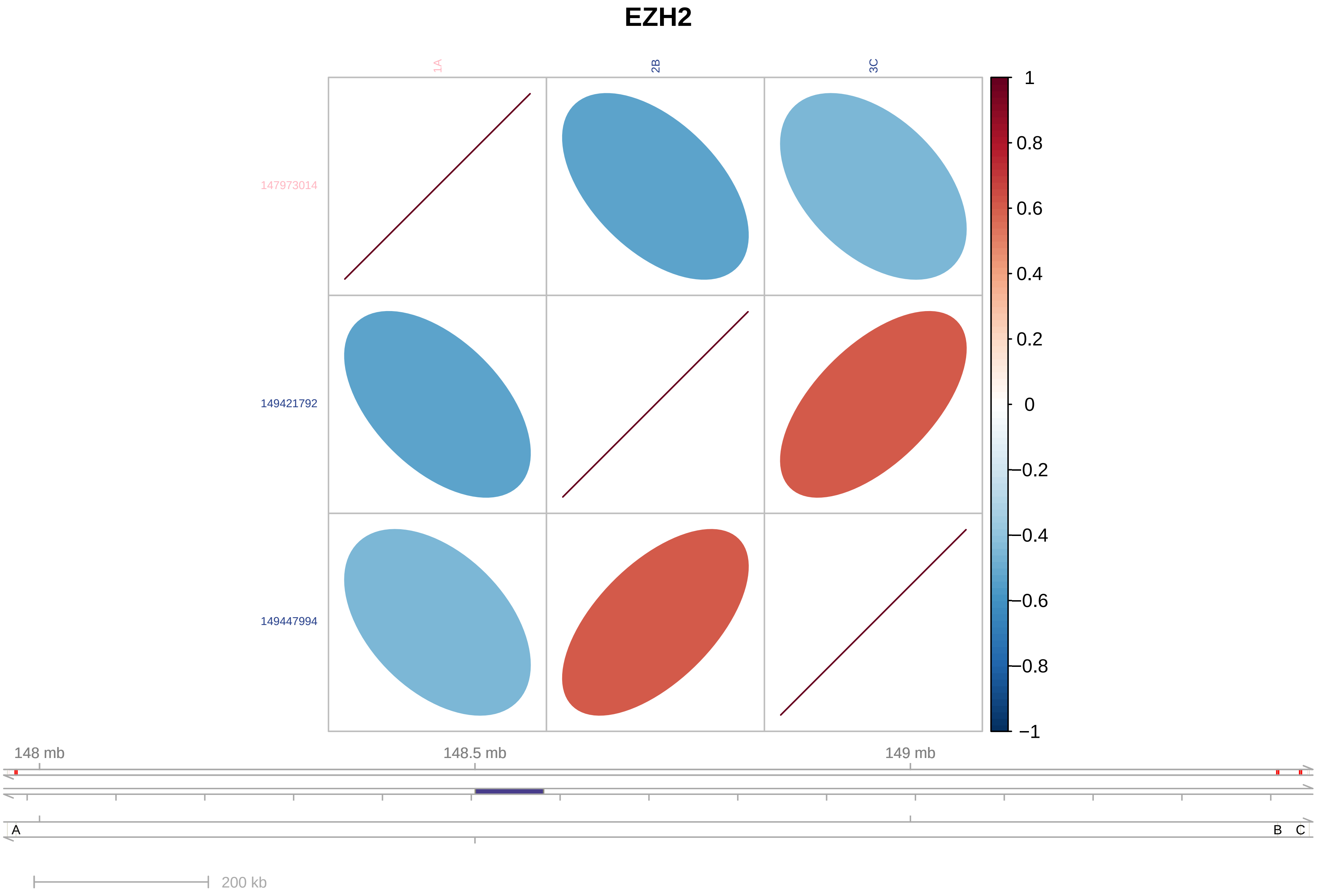

EZH2
1A
2B
3C
147973014
149421792
149447994
1
0.8
0.6
0.4
0.2
0
−0.2
−0.4
−0.6
−0.8
−1
148 mb
148.5 mb
149 mb
A
B
C
200 kb

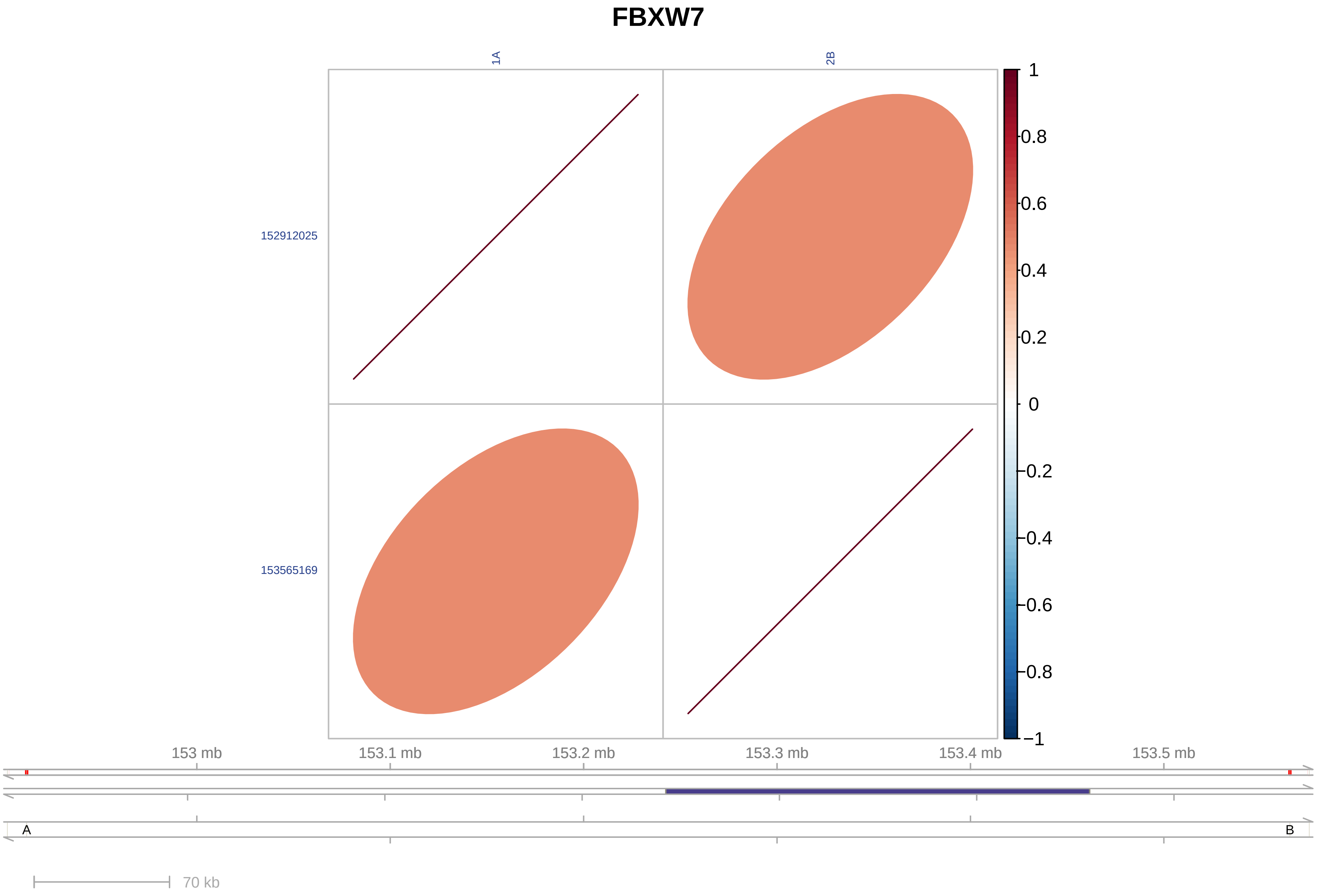

FBXW7
1A
2B
152912025
153565169
1
0.8
0.6
0.4
0.2
0
−0.2
−0.4
−0.6
−0.8
−1
153 mb
153.1 mb
153.2 mb
153.3 mb
153.4 mb
153.5 mb
A
B
70 kb

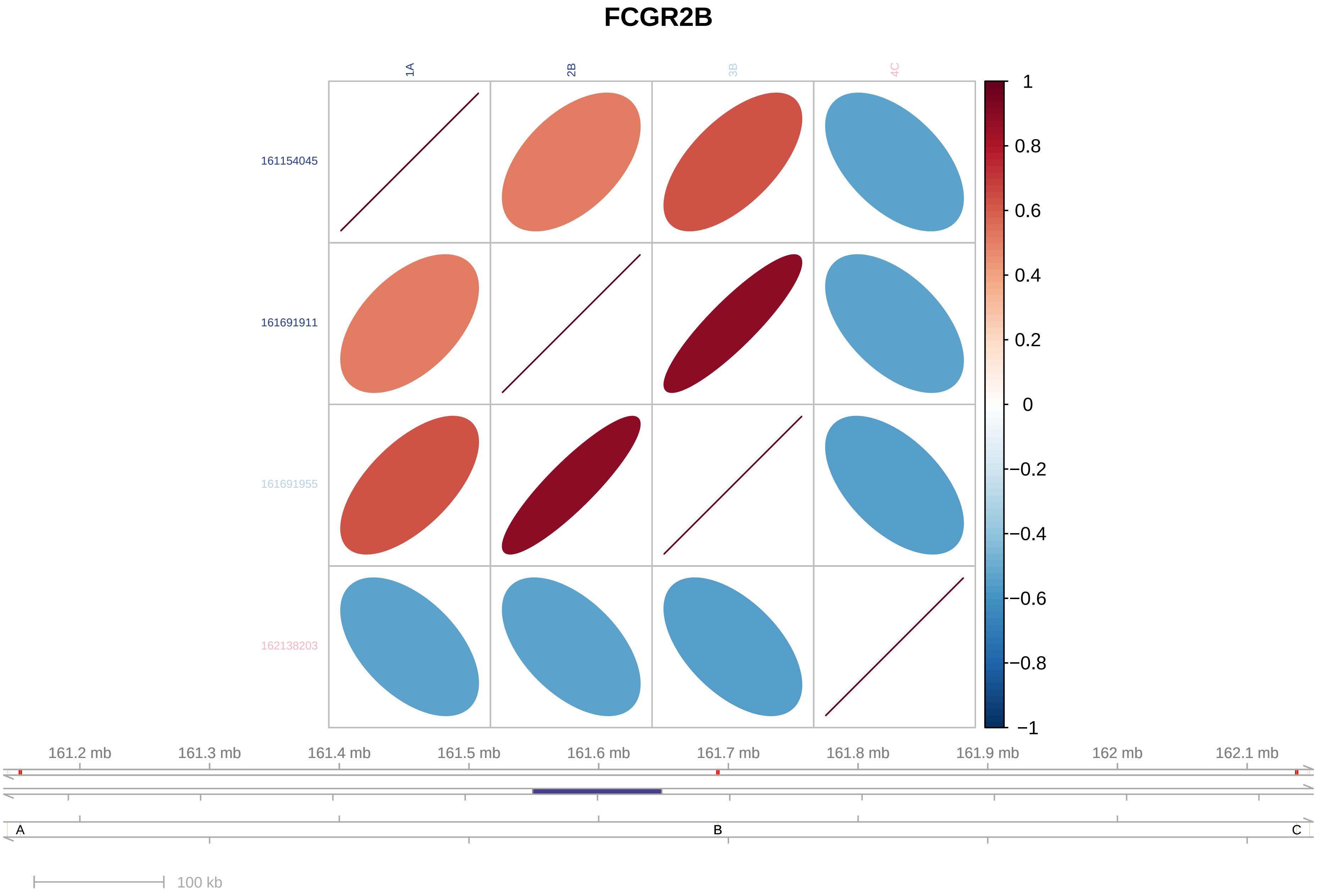
FCGR2B
1A
2B
3B
4C
161154045
161691911
161691955
162138203
1
0.8
0.6
0.4
0.2
0
−0.2
−0.4
−0.6
−0.8
−1
161.2 mb
161.3 mb
161.4 mb
161.5 mb
161.6 mb
161.7 mb
161.8 mb
161.9 mb
162 mb
162.1 mb
A
B
C
100 kb

FGF17

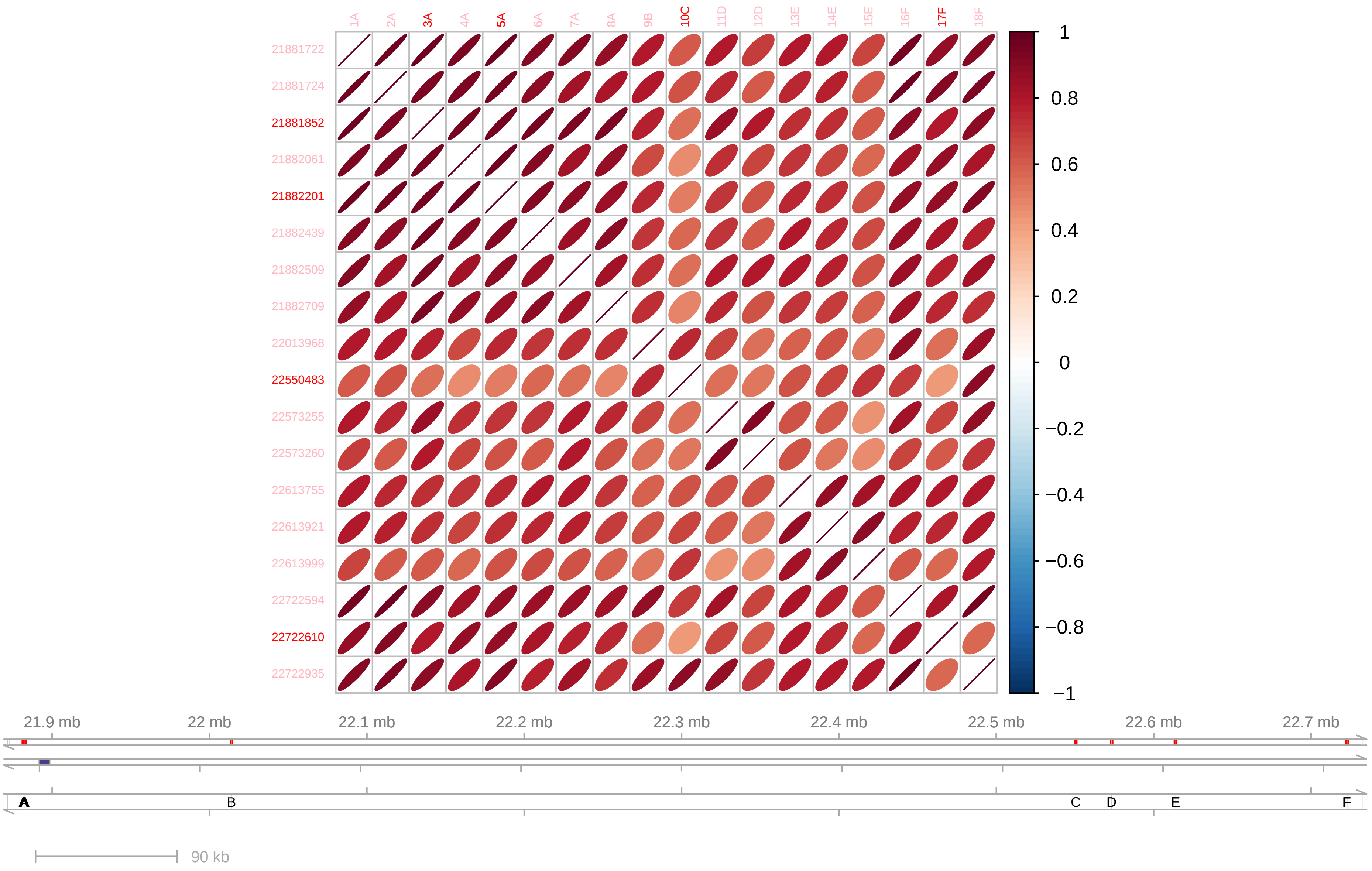

1A
2A
3A
4A
5A
6A
7A
8A
9B
10C
11D
12D
13E
14E
15E
16F
17F
18F
21881722
21881724
21881852
21882061
21882201
21882439
21882509
21882709
22013968
22550483
22573255
22573260
22613755
22613921
22613999
22722594
22722610
22722935
1
0.8
0.6
0.4
0.2
0
−0.2
−0.4
−0.6
−0.8
−1
21.9 mb
22 mb
22.1 mb
22.2 mb
22.3 mb
22.4 mb
22.5 mb
22.6 mb
22.7 mb
A
B
C
D
E
F
90 kb

FGFR2

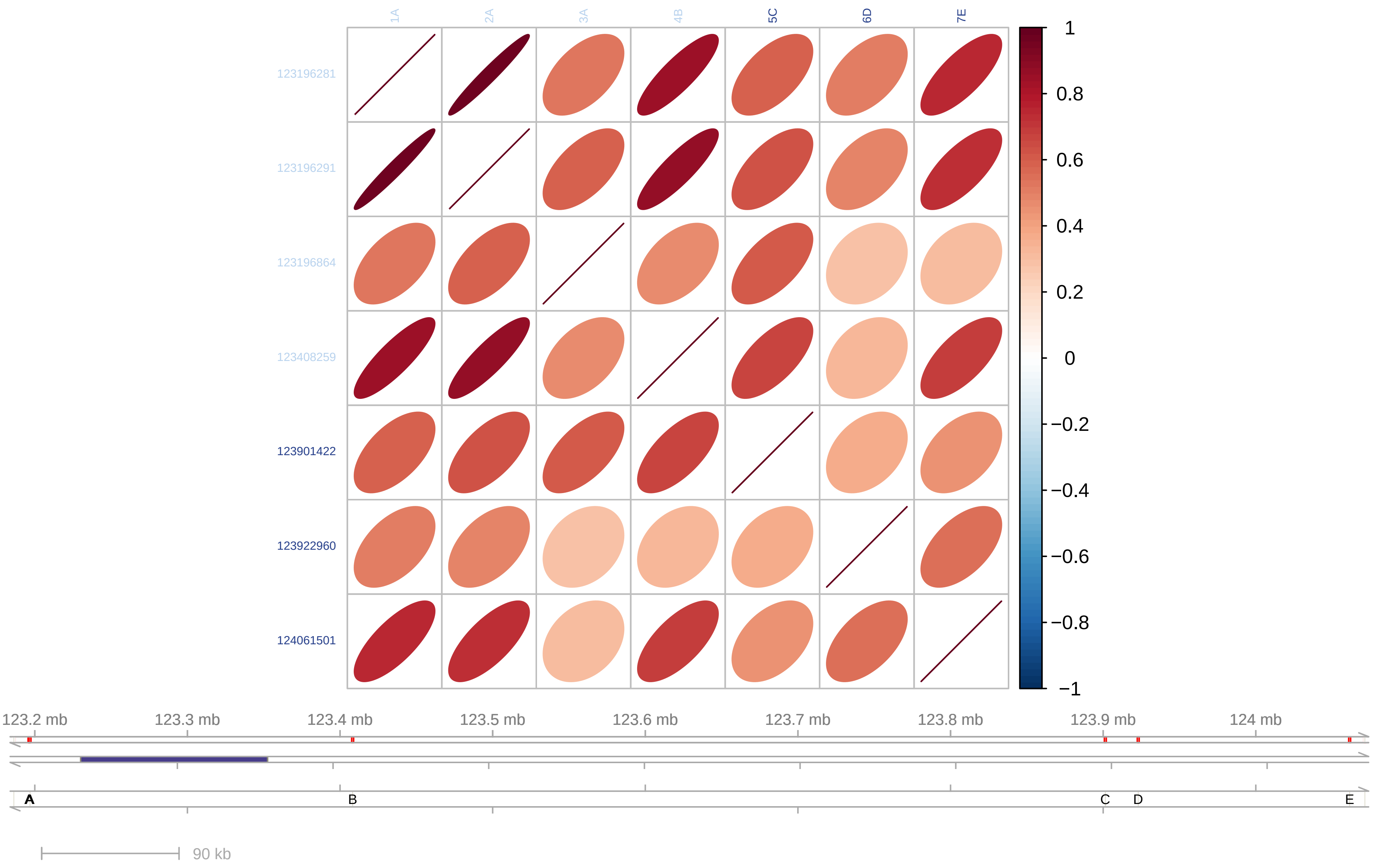

1A
2A
3A
4B
5C
6D
7E
123196281
123196291
123196864
123408259
123901422
123922960
124061501
1
0.8
0.6
0.4
0.2
0
−0.2
−0.4
−0.6
−0.8
−1
123.2 mb
123.3 mb
123.4 mb
123.5 mb
123.6 mb
123.7 mb
123.8 mb
123.9 mb
124 mb
A
B
C
D
E
90 kb

FGFR3

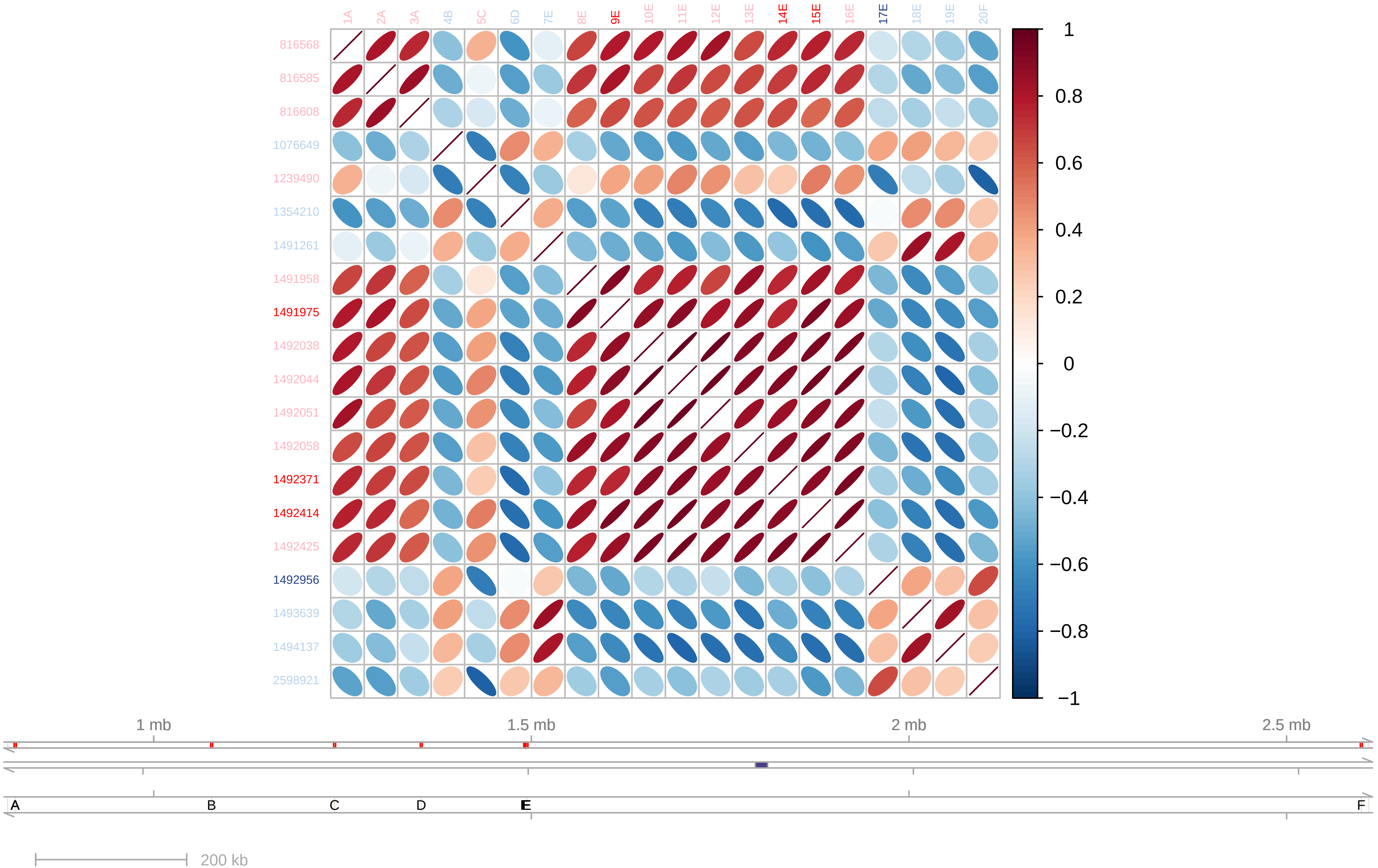

1A
2A
3A
4B
5C
6D
7E
8E
9E
10E
11E
12E
13E
14E
15E
16E
17E
18E
19E
20F
816568
816585
816608
1076649
1239490
1354210
1491261
1491958
1491975
1492038
1492044
1492051
1492058
1492371
1492414
1492425
1492956
1493639
1494137
2598921
1
0.8
0.6
0.4
0.2
0
−0.2
−0.4
−0.6
−0.8
−1
1 mb
1.5 mb
2 mb
2.5 mb
A
B
C
D
E
F
200 kb

FUBP1

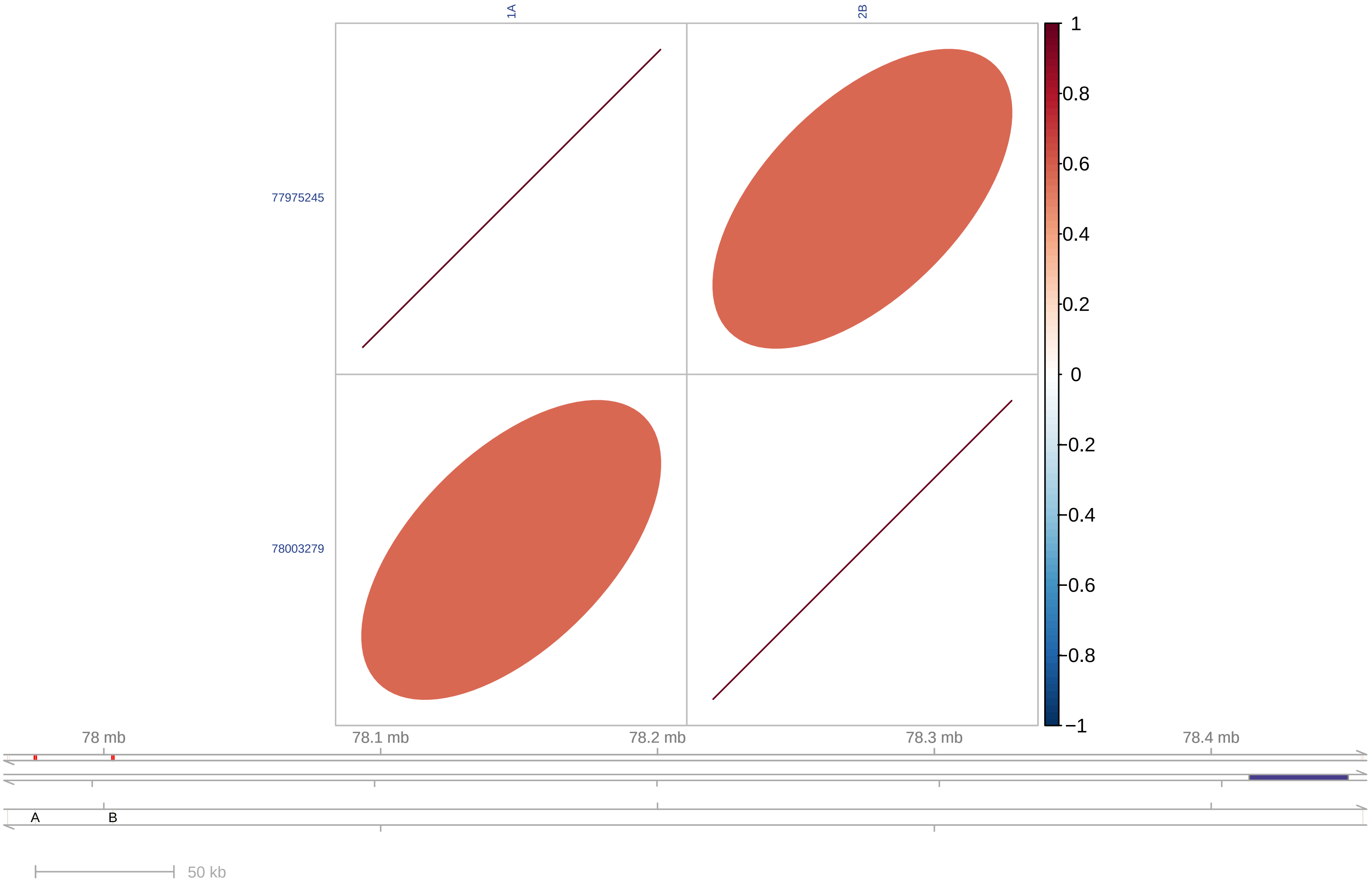

1A
2B
77975245
78003279
1
0.8
0.6
0.4
0.2
0
−0.2
−0.4
−0.6
−0.8
−1
78 mb
78.1 mb
78.2 mb
78.3 mb
78.4 mb
A
B
50 kb

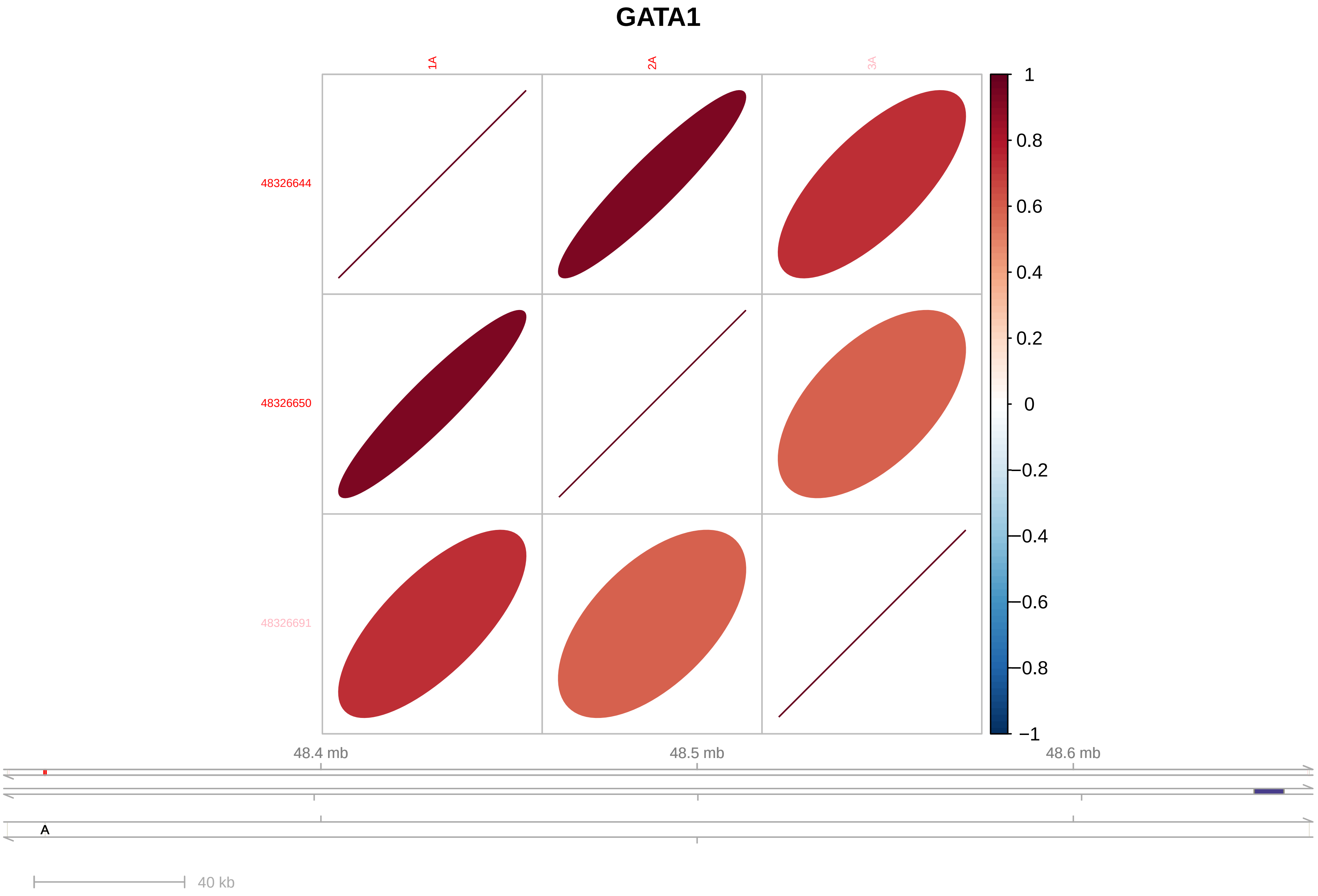
GATA1
1A
2A
3A
48326644
48326650
48326691
1
0.8
0.6
0.4
0.2
0
−0.2
−0.4
−0.6
−0.8
−1
48.4 mb
48.5 mb
48.6 mb
A
40 kb

GDF15

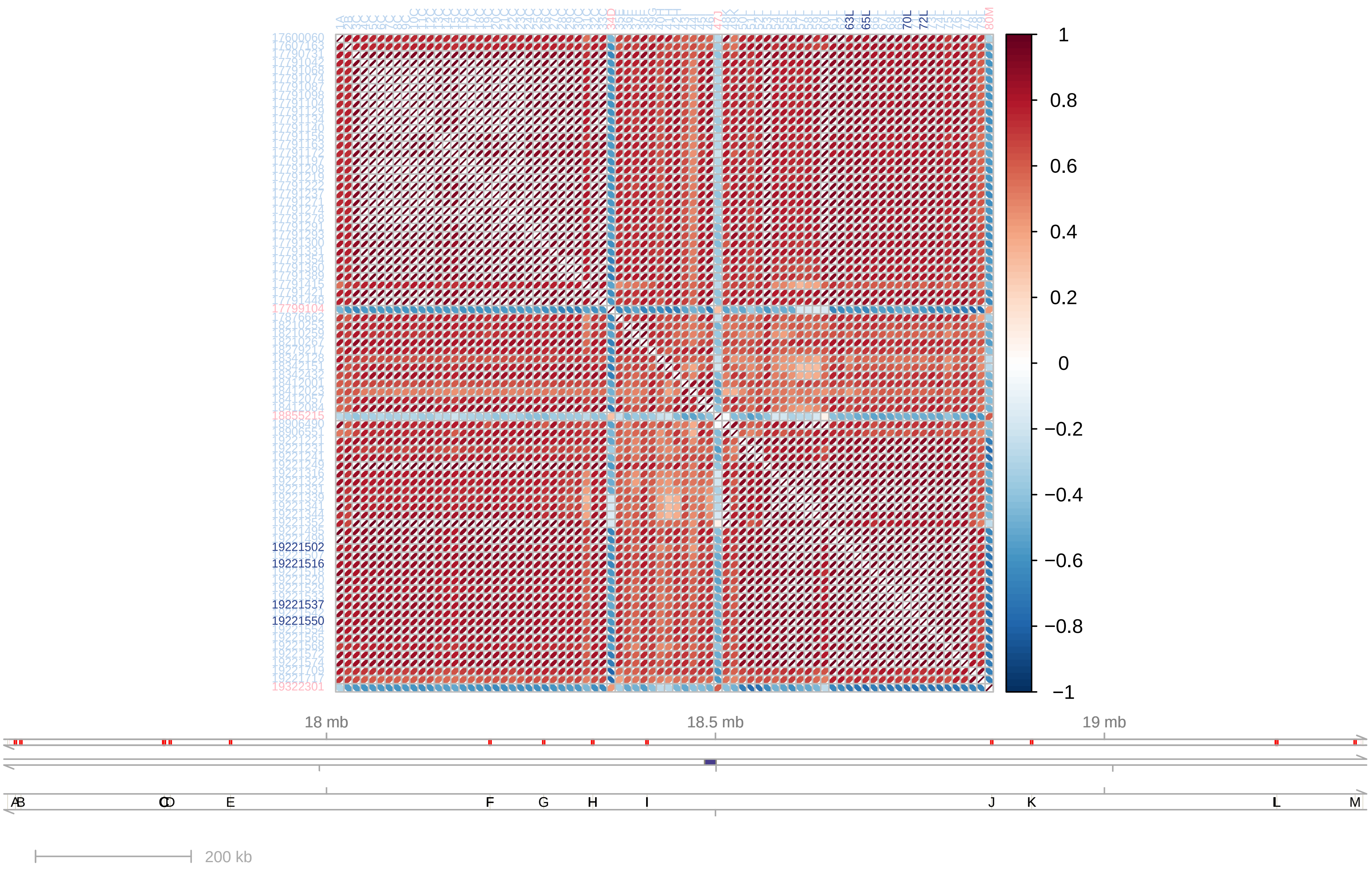
63L
65L
70L
72L
19221502
19221516
19221537
19221550
1
0.8
0.6
0.4
0.2
0
−0.2
−0.4
−0.6
−0.8
−1
18 mb
18.5 mb
19 mb
A
B
C
D
E
F
G
H
I
J
K
L
M
200 kb

GNA11

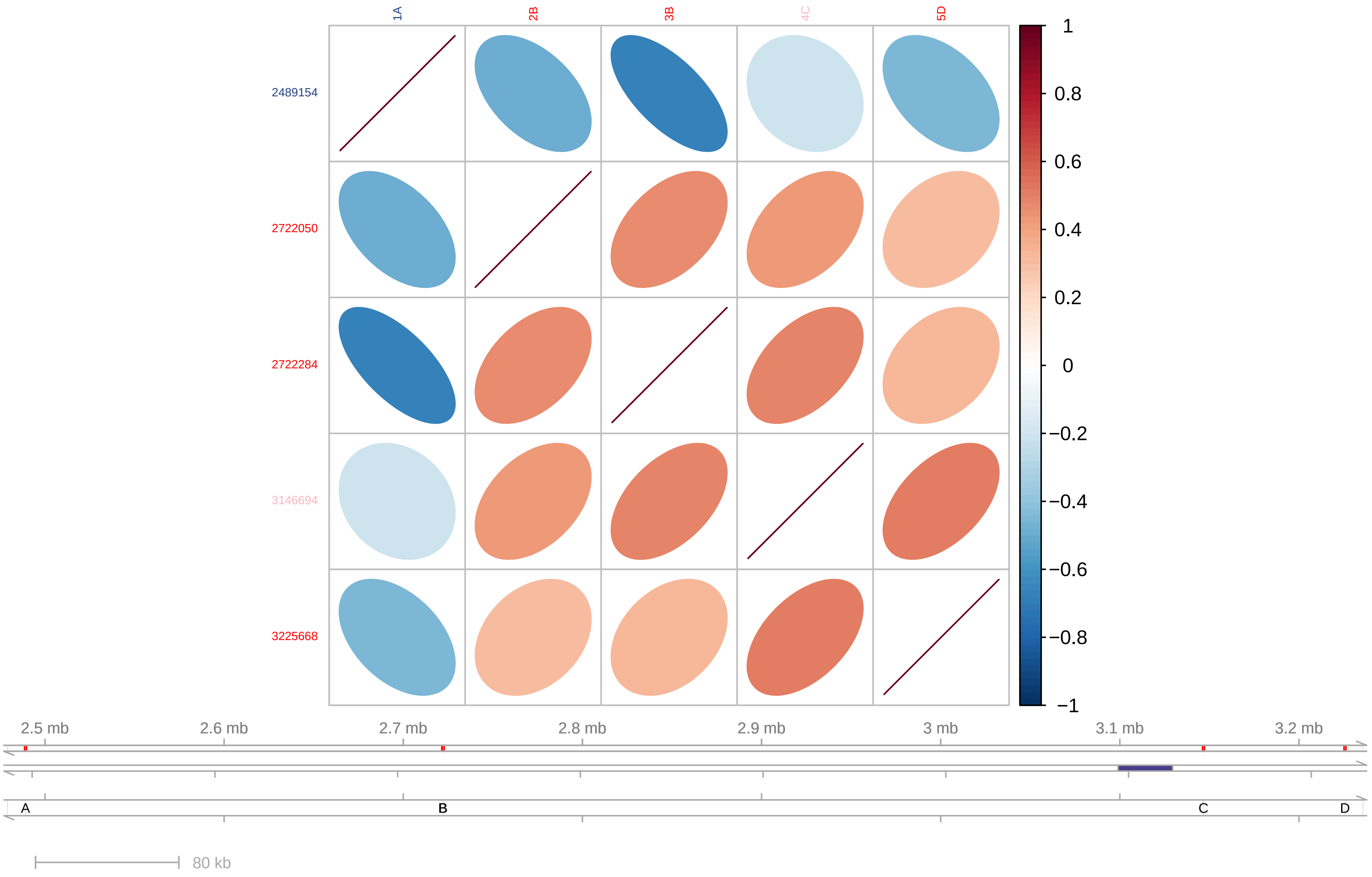
1A
2B
3B
4C
5D
2489154
2722050
2722284
3146694
3225668
1
0.8
0.6
0.4
0.2
0
−0.2
−0.4
−0.6
−0.8
−1
2.5 mb
2.6 mb
2.7 mb
2.8 mb
2.9 mb
3 mb
3.1 mb
3.2 mb
A
B
C
D
80 kb

GNAS

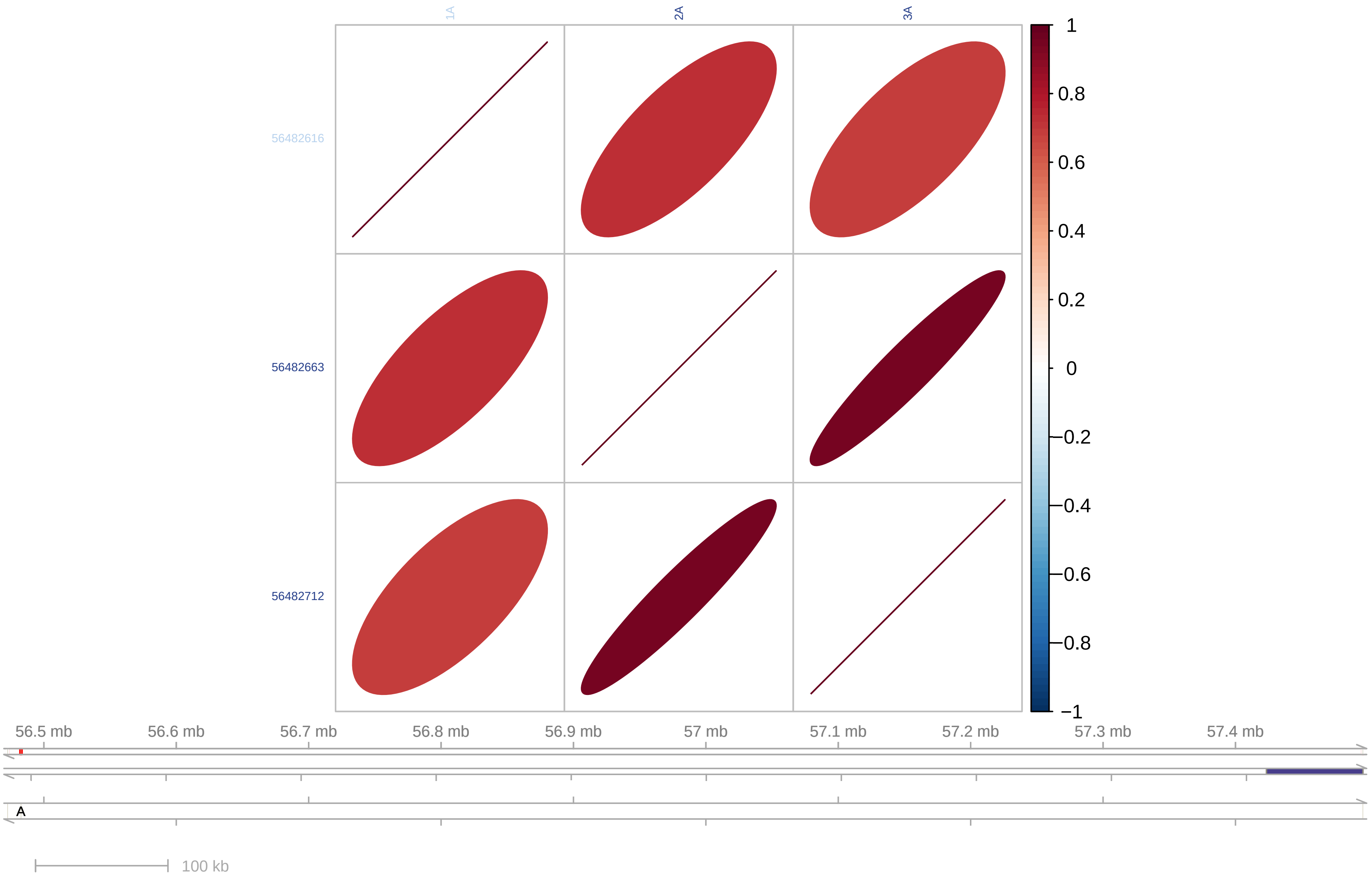
1A
2A
3A
56482616
56482663
56482712
1
0.8
0.6
0.4
0.2
0
−0.2
−0.4
−0.6
−0.8
−1
56.5 mb
56.6 mb
56.7 mb
56.8 mb
56.9 mb
57 mb
57.1 mb
57.2 mb
57.3 mb
57.4 mb
A
100 kb

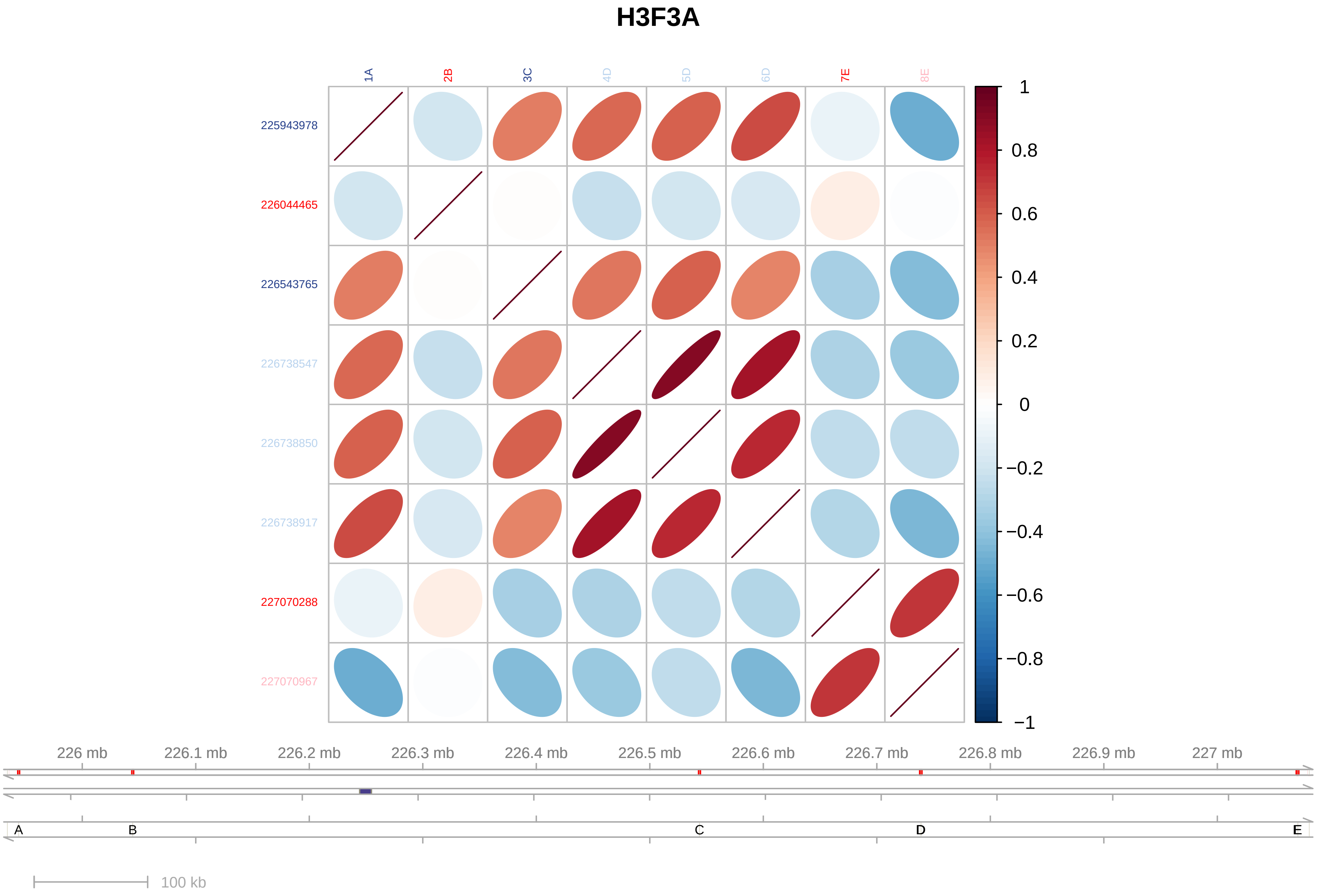

H3F3A
1A
2B
3C
4D
5D
6D
7E
8E
225943978
226044465
226543765
226738547
226738850
226738917
227070288
227070967
1
0.8
0.6
0.4
0.2
0
−0.2
−0.4
−0.6
−0.8
−1
226 mb
226.1 mb
226.2 mb
226.3 mb
226.4 mb
226.5 mb
226.6 mb
226.7 mb
226.8 mb
226.9 mb
227 mb
A
B
C
D
E
100 kb

HK3

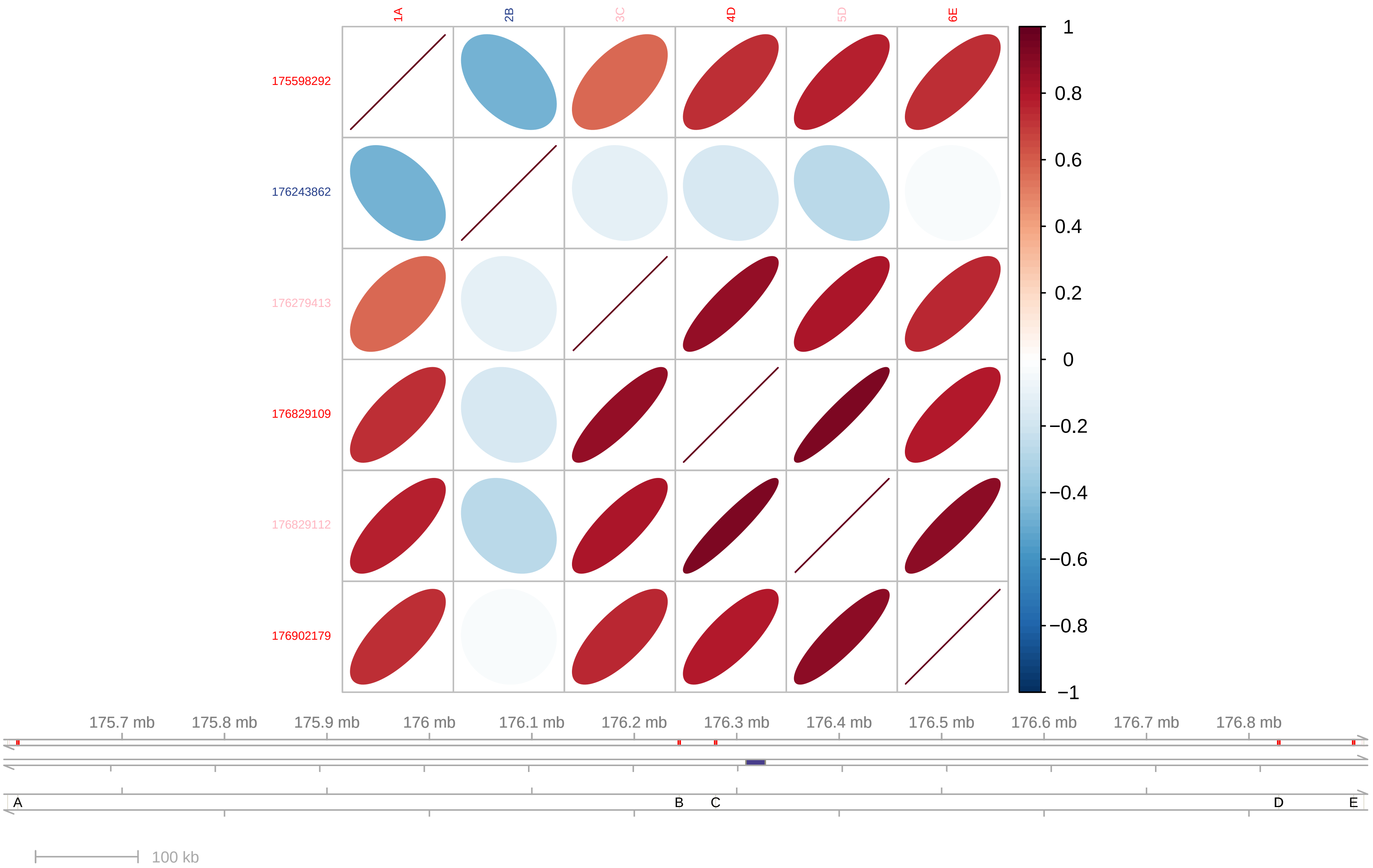

1A
2B
3C
4D
5D
6E
175598292
176243862
176279413
176829109
176829112
176902179
1
0.8
0.6
0.4
0.2
0
−0.2
−0.4
−0.6
−0.8
−1
175.7 mb
175.8 mb
175.9 mb
176 mb
176.1 mb
176.2 mb
176.3 mb
176.4 mb
176.5 mb
176.6 mb
176.7 mb
176.8 mb
A
B
C
D
E
100 kb

HRAS

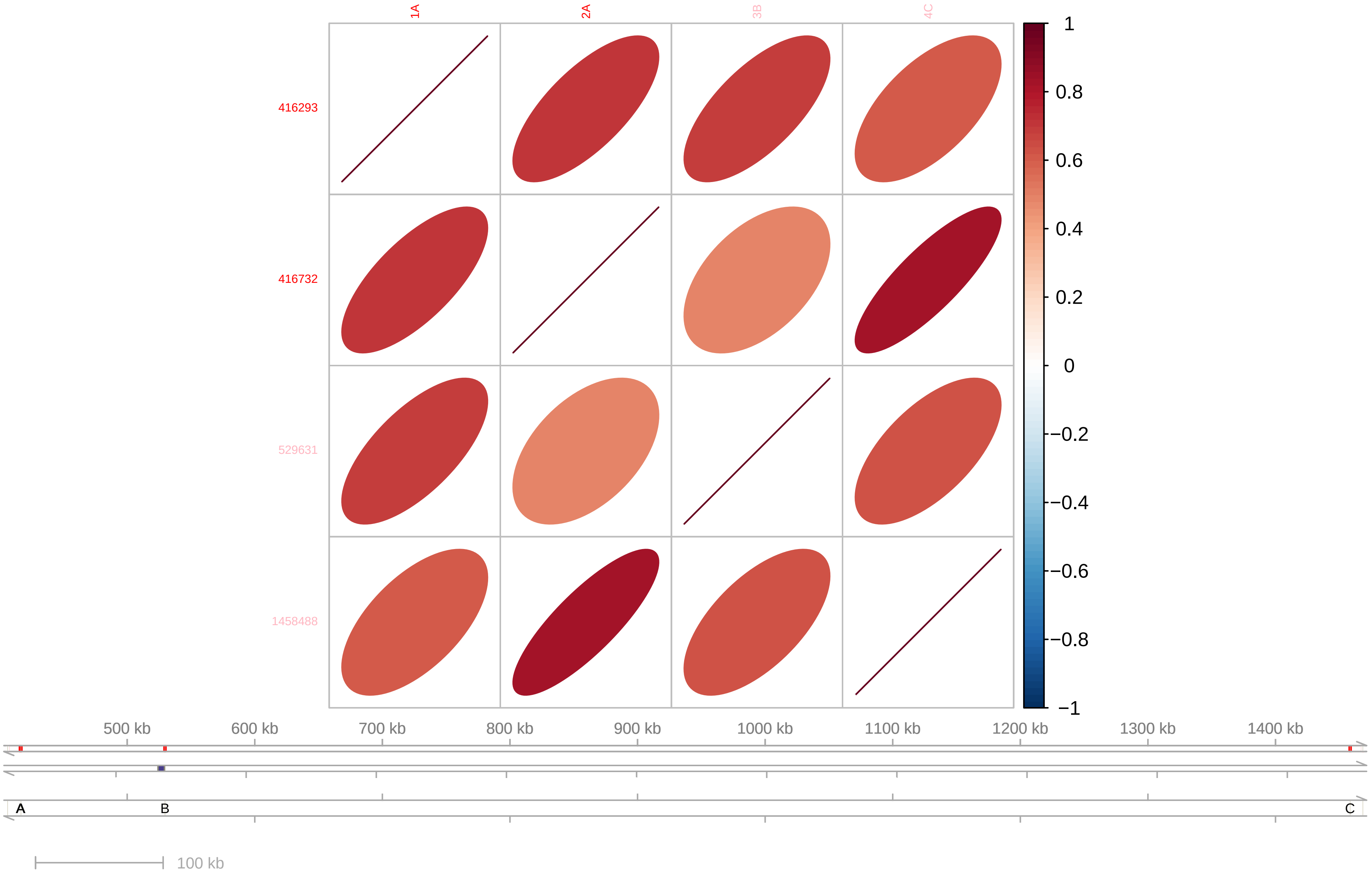
1A
2A
3B
4C
416293
416732
529631
1458488
1
0.8
0.6
0.4
0.2
0
−0.2
−0.4
−0.6
−0.8
−1
500 kb
600 kb
700 kb
800 kb
900 kb
1000 kb
1100 kb
1200 kb
1300 kb
1400 kb
A
B
C
100 kb

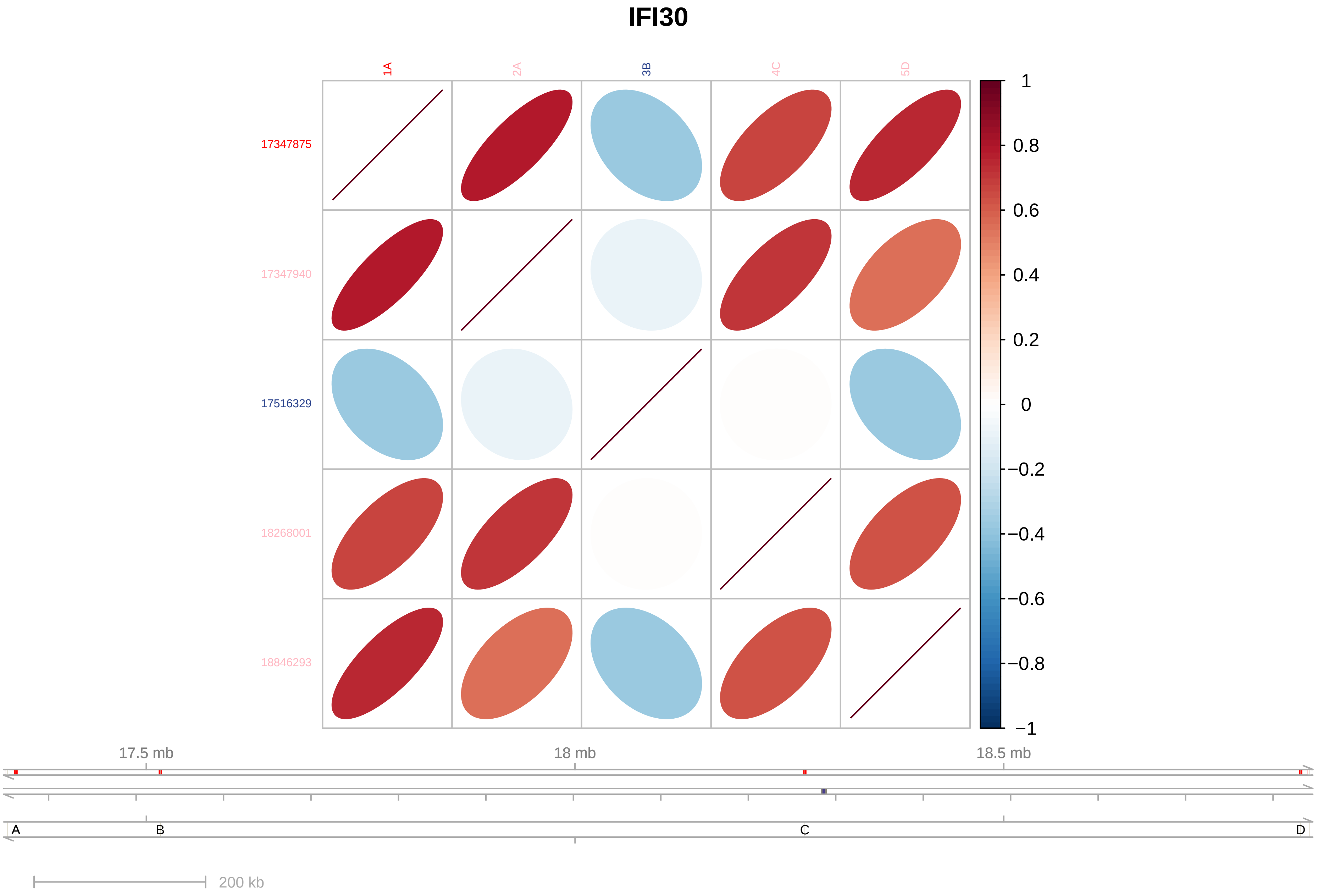
IFI30
1A
2A
3B
4C
5D
17347875
17347940
17516329
18268001
18846293
1
0.8
0.6
0.4
0.2
0
−0.2
−0.4
−0.6
−0.8
−1
17.5 mb
18 mb
18.5 mb
A
B
C
D
200 kb

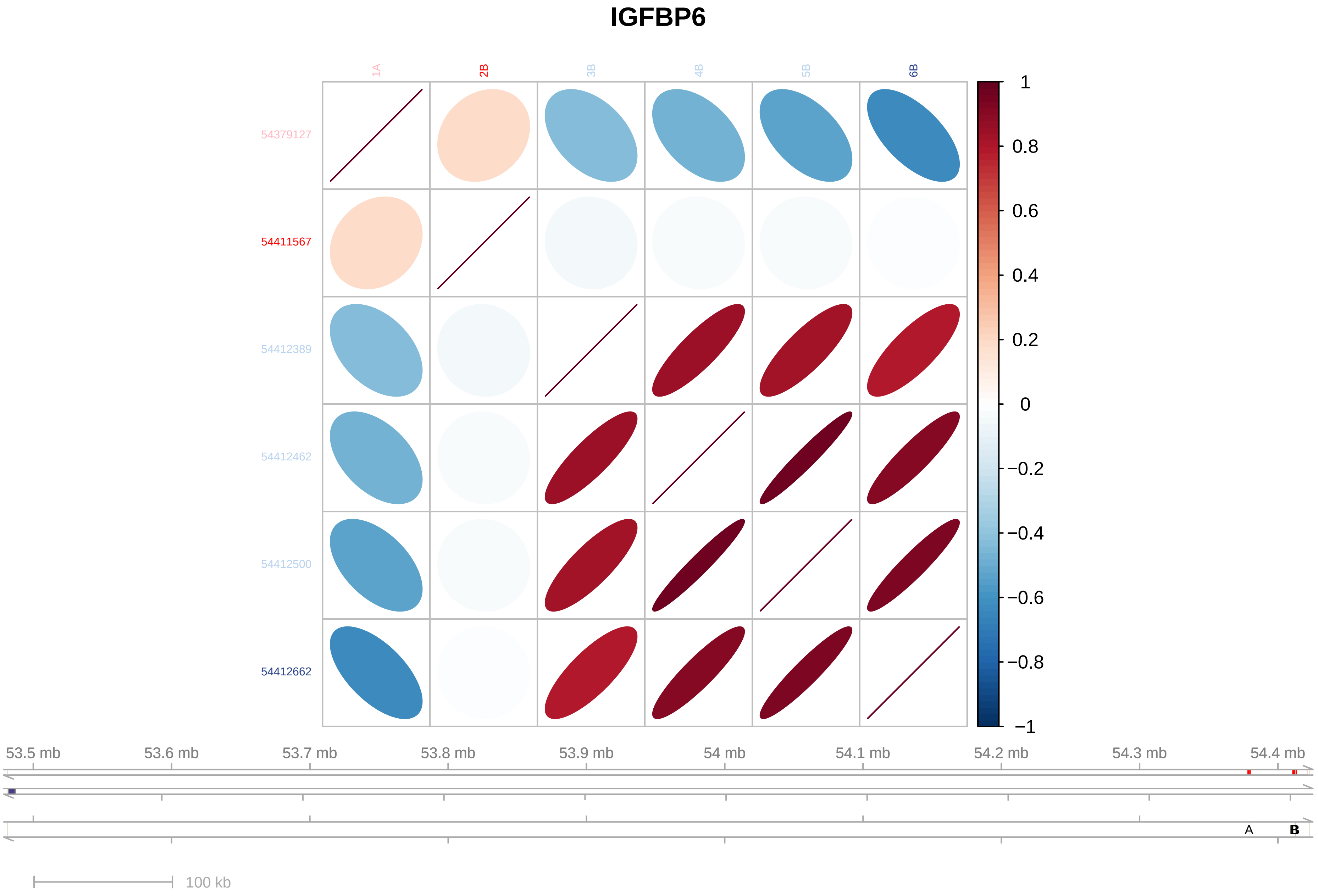
IGFBP6
1A
2B
3B
4B
5B
6B
54379127
54411567
54412389
54412462
54412500
54412662
1
0.8
0.6
0.4
0.2
0
−0.2
−0.4
−0.6
−0.8
−1
53.5 mb
53.6 mb
53.7 mb
53.8 mb
53.9 mb
54 mb
54.1 mb
54.2 mb
54.3 mb
54.4 mb
A
B
100 kb

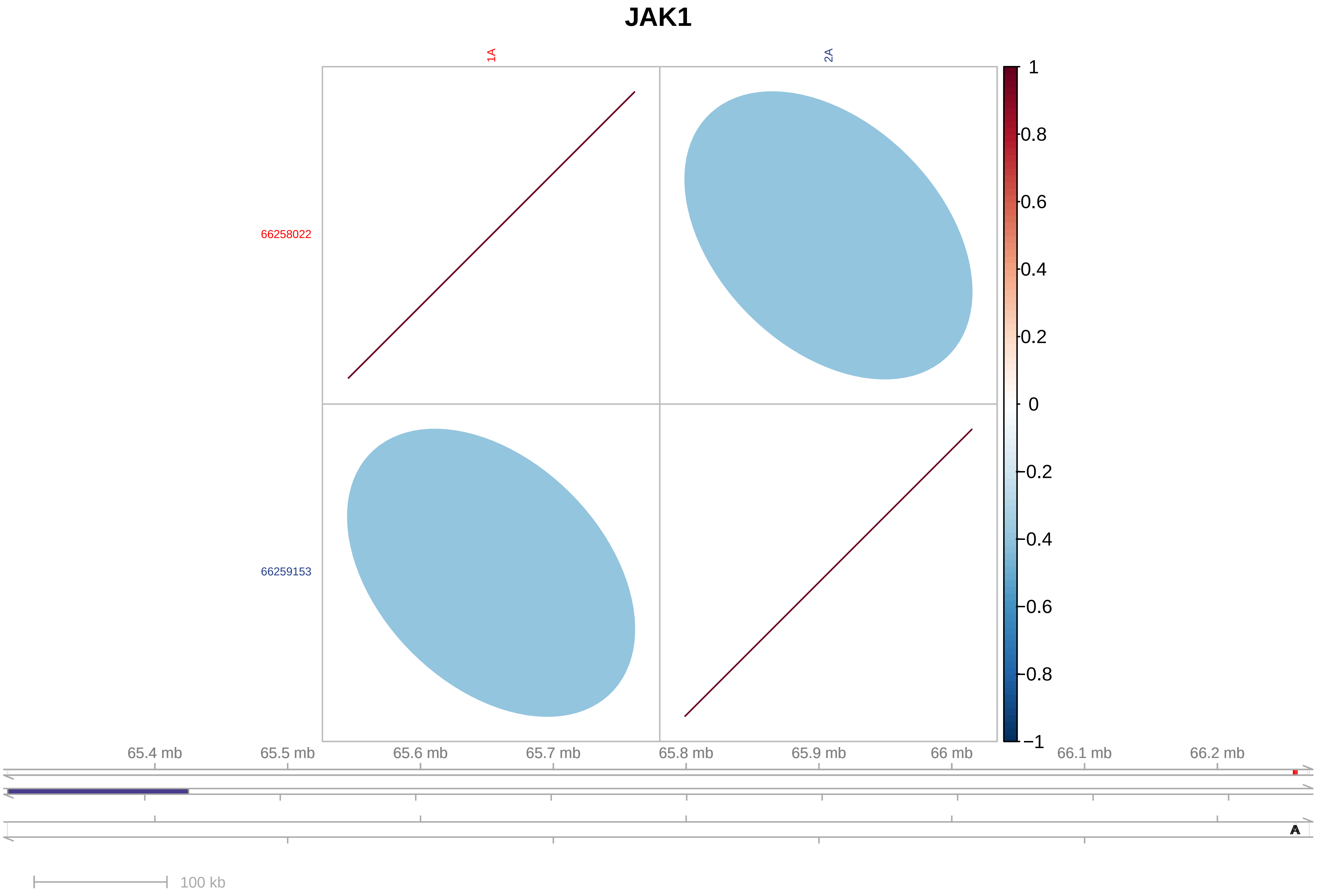

JAK1
1A
2A
66258022
66259153
1
0.8
0.6
0.4
0.2
0
−0.2
−0.4
−0.6
−0.8
−1
65.4 mb
65.5 mb
65.6 mb
65.7 mb
65.8 mb
65.9 mb
66 mb
66.1 mb
66.2 mb
100 kb

KDM5C

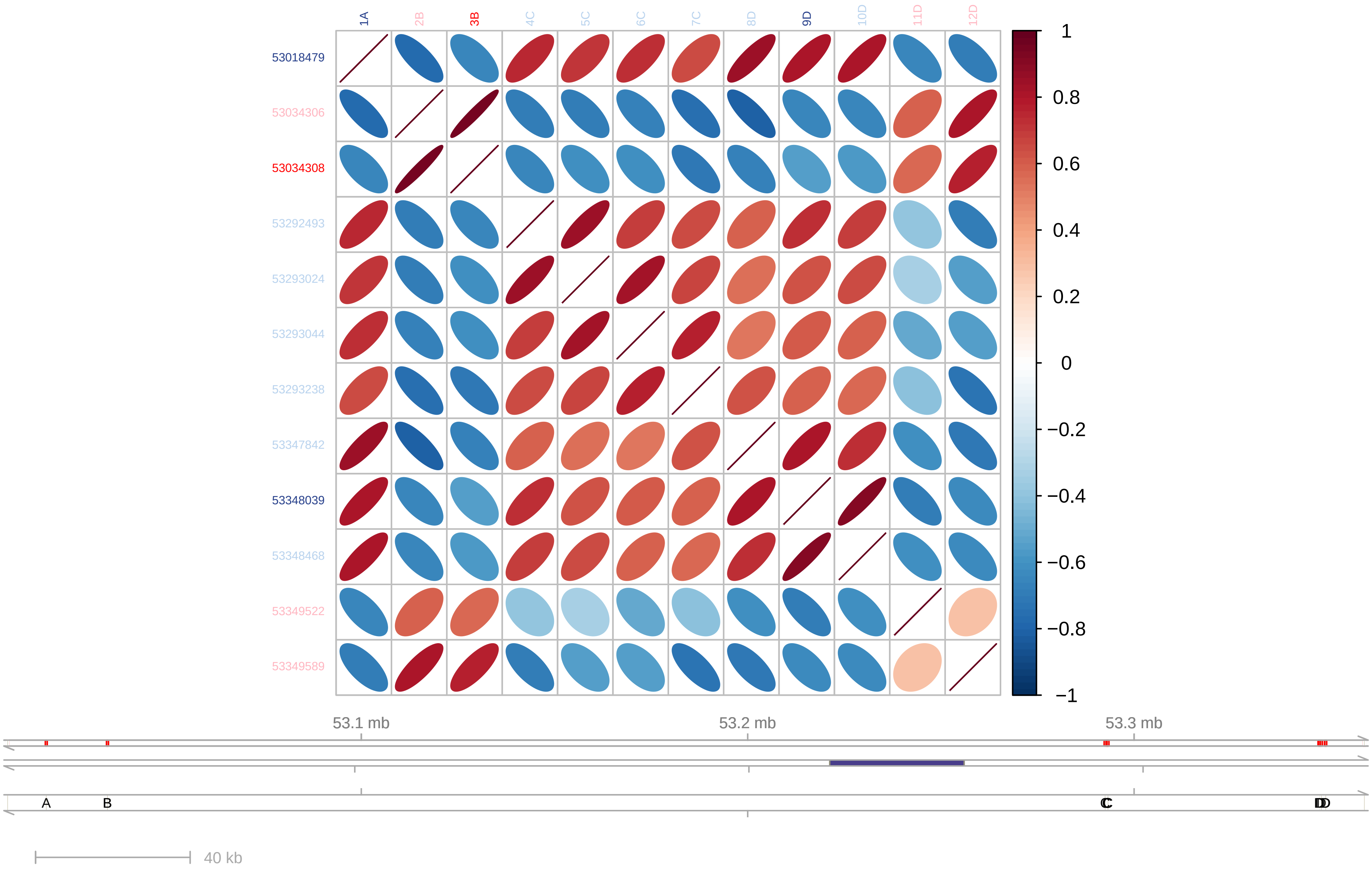

1A
2B
3B
4C
5C
6C
7C
8D
9D
10D
11D
12D
53018479
53034306
53034308
53292493
53293024
53293044
53293238
53347842
53348039
53348468
53349522
53349589
1
0.8
0.6
0.4
0.2
0
−0.2
−0.4
−0.6
−0.8
−1
53.1 mb
53.2 mb
53.3 mb
A
B
C
D
40 kb

KLF4

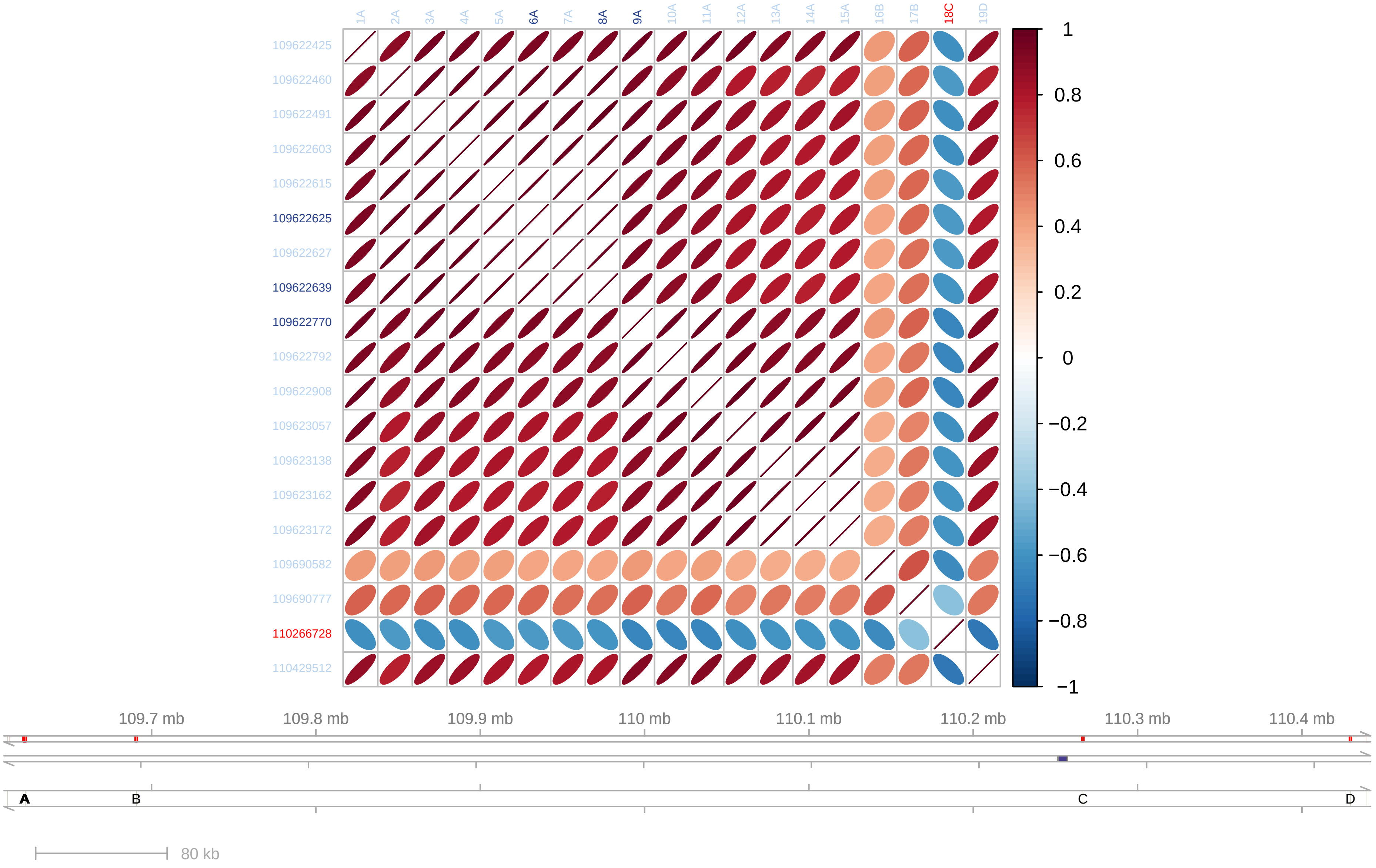

1A
2A
3A
4A
5A
6A
7A
8A
9A
10A
11A
12A
13A
14A
15A
16B
17B
18C
19D
109622425
109622460
109622491
109622603
109622615
109622625
109622627
109622639
109622770
109622792
109622908
109623057
109623138
109623162
109623172
109690582
109690777
110266728
110429512
1
0.8
0.6
0.4
0.2
0
−0.2
−0.4
−0.6
−0.8
−1
109.7 mb
109.8 mb
109.9 mb
110 mb
110.1 mb
110.2 mb
110.3 mb
110.4 mb
A
B
C
D
80 kb

KMT2D

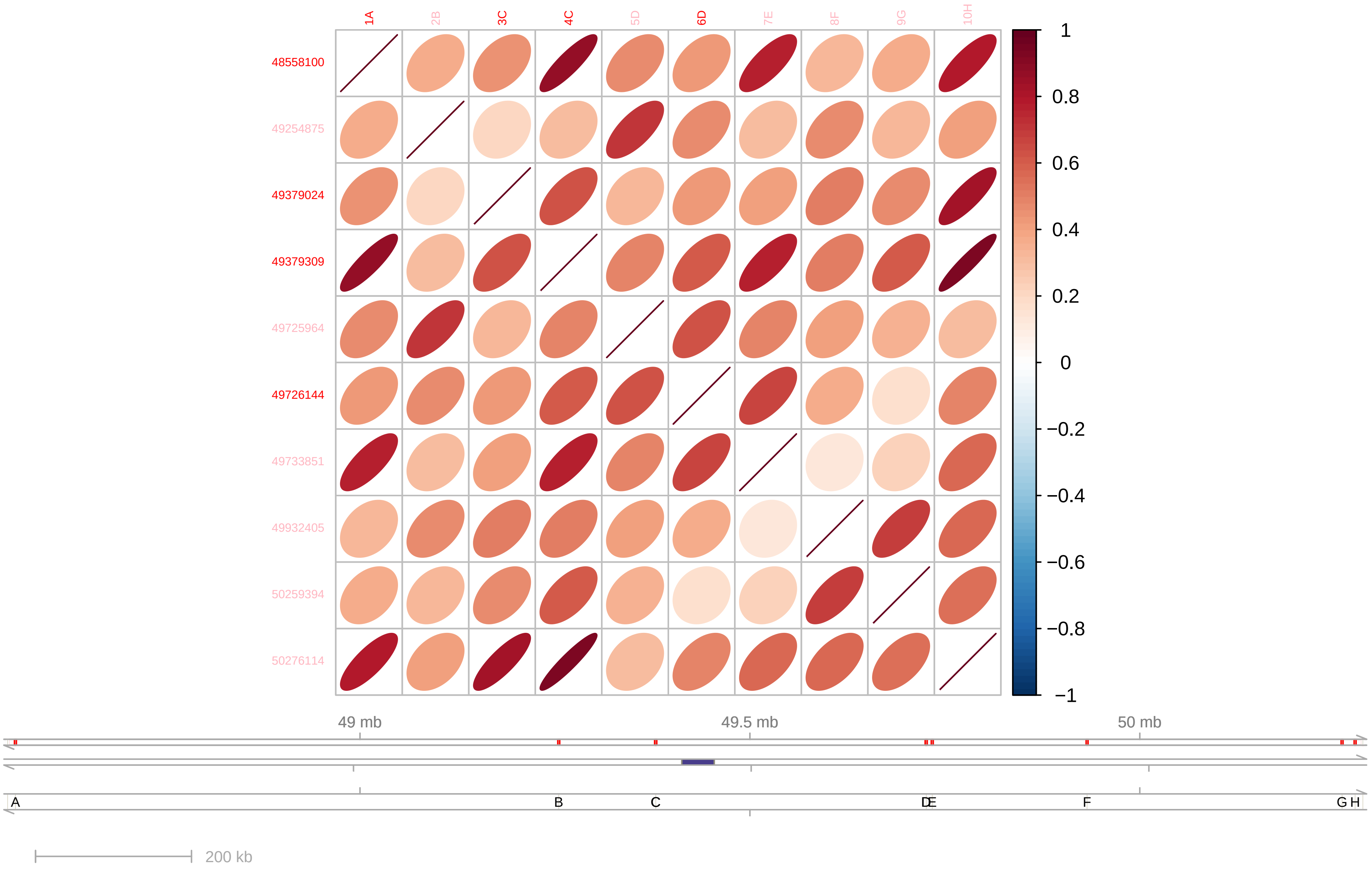

1A
2B
3C
4C
5D
6D
7E
8F
9G
10H
48558100
49254875
49379024
49379309
49725964
49726144
49733851
49932405
50259394
50276114
1
0.8
0.6
0.4
0.2
0
−0.2
−0.4
−0.6
−0.8
−1
49 mb
49.5 mb
50 mb
A
B
C
DE
F
G H
200 kb

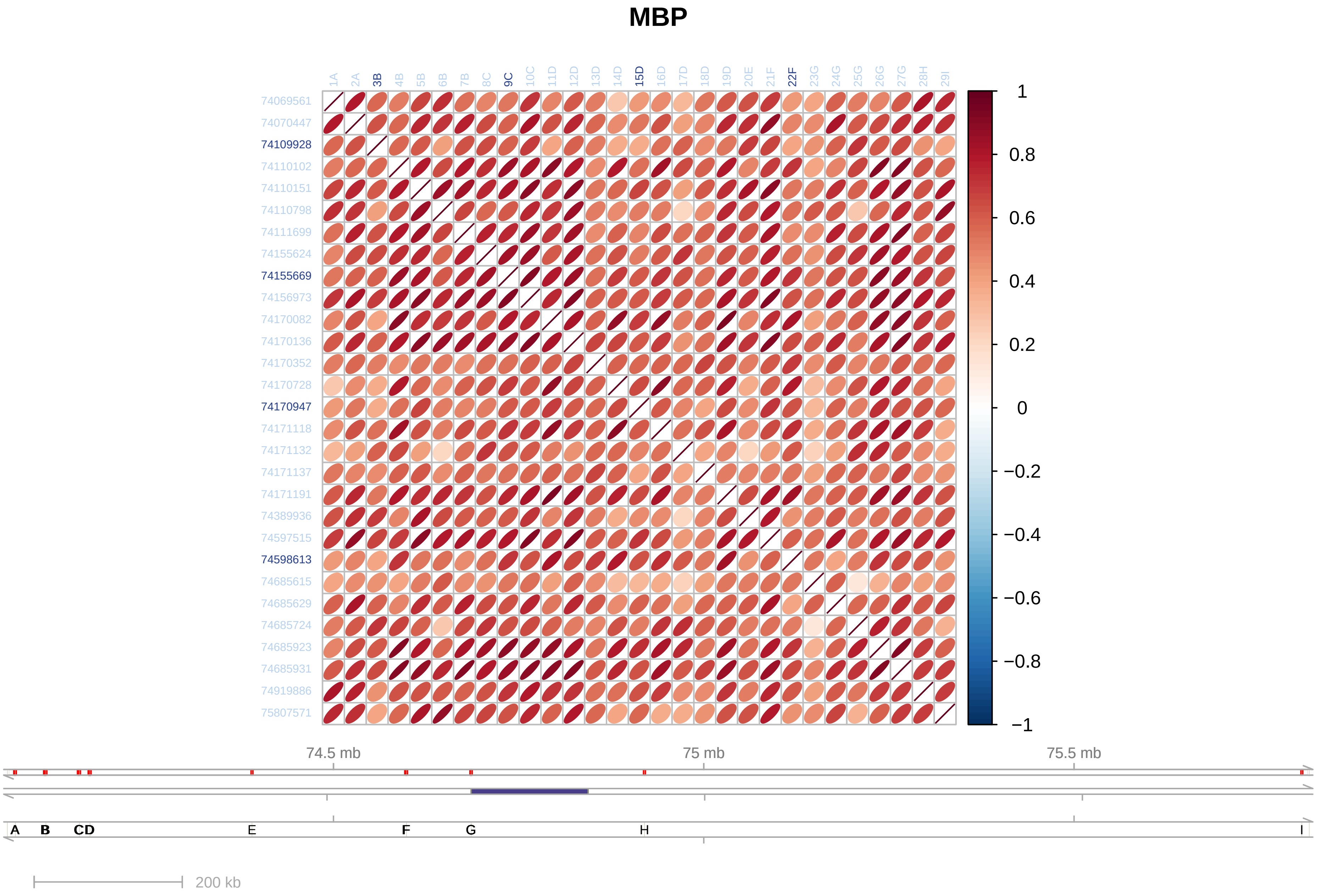
MBP
1A
2A
3B
4B
5B
6B
7B
8C
9C
10C
11D
12D
13D
14D
15D
16D
17D
18D
19D
20E
21F
22F
23G
24G
25G
26G
27G
28H
29I
74069561
74070447
74109928
74110102
74110151
74110798
74111699
74155624
74155669
74156973
74170082
74170136
74170352
74170728
74170947
74171118
74171132
74171137
74171191
74389936
74597515
74598613
74685615
74685629
74685724
74685923
74685931
74919886
75807571
1
0.8
0.6
0.4
0.2
0
−0.2
−0.4
−0.6
−0.8
−1
74.5 mb
75 mb
75.5 mb
A
B
CD
E
F
G
H
I
200 kb

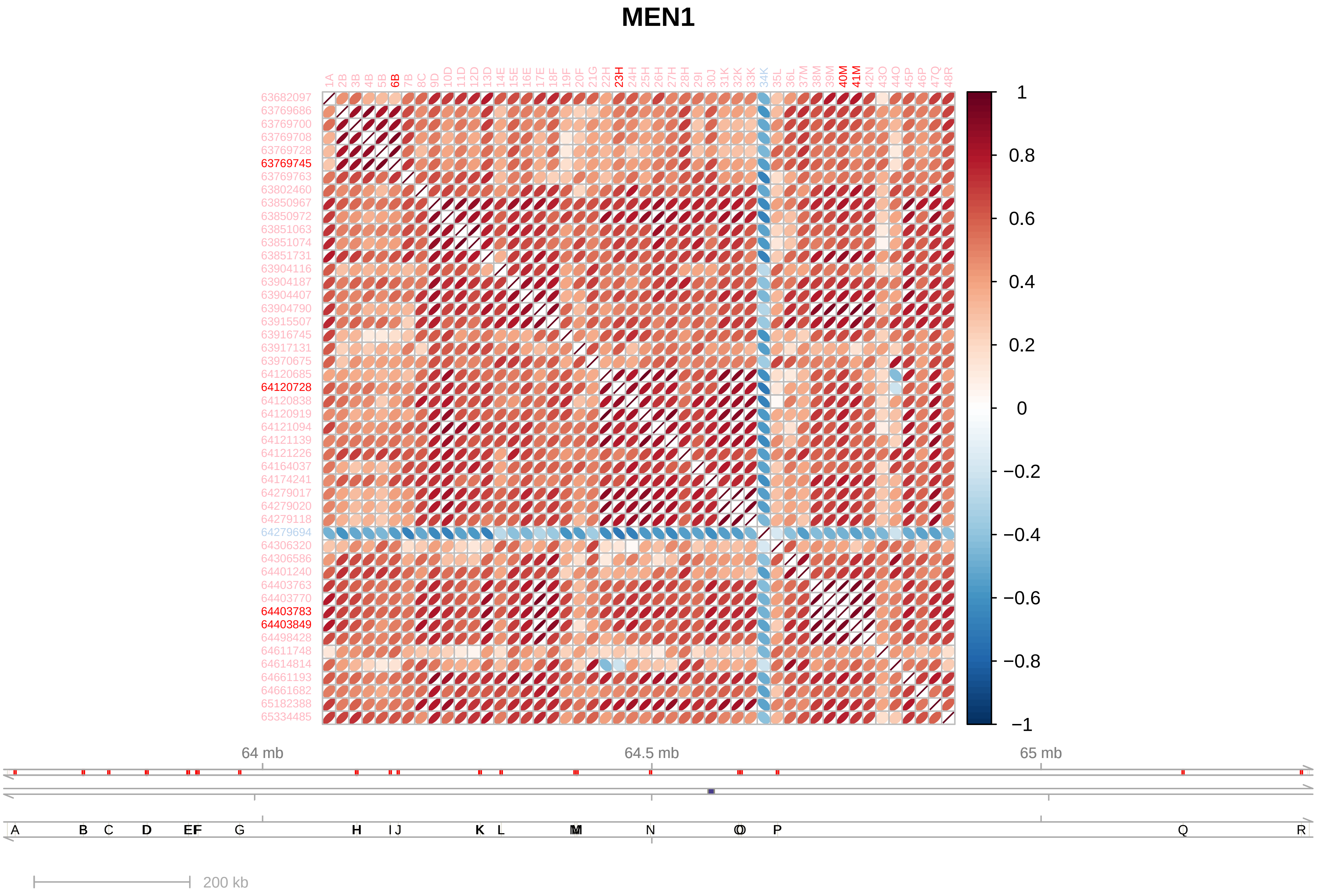
MEN1
1
0.8
0.6
0.4
0.2
0
−0.2
−0.4
−0.6
−0.8
−1
64 mb
64.5 mb
65 mb
A B C D EF G H I J K L M N O P Q R
200 kb

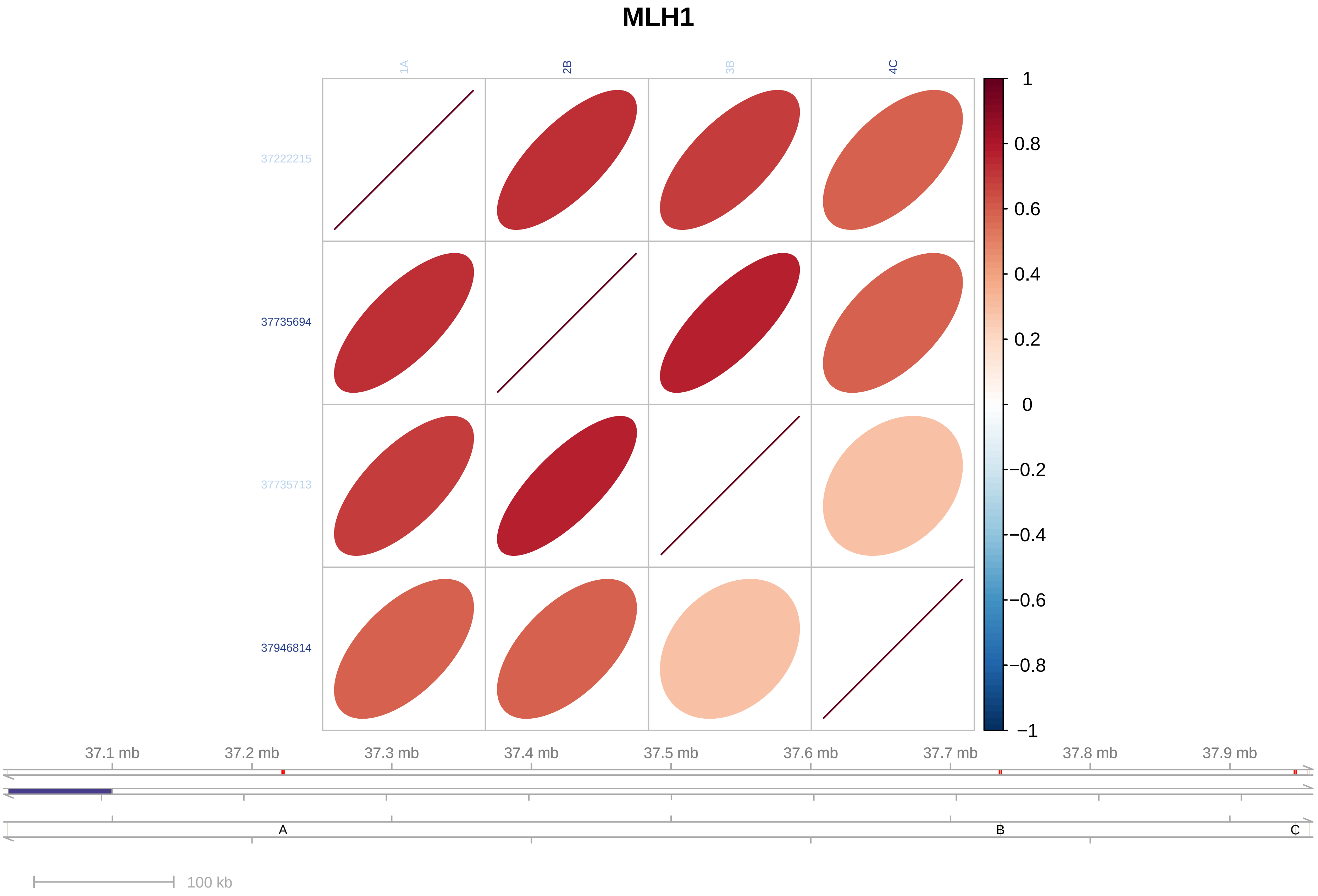

MLH1
1A
2B
3B
4C
37222215
37735694
37735713
37946814
1
0.8
0.6
0.4
0.2
0
−0.2
−0.4
−0.6
−0.8
−1
37.1 mb
37.2 mb
37.3 mb
37.4 mb
37.5 mb
37.6 mb
37.7 mb
37.8 mb
37.9 mb
A
B
C
100 kb

MSH2

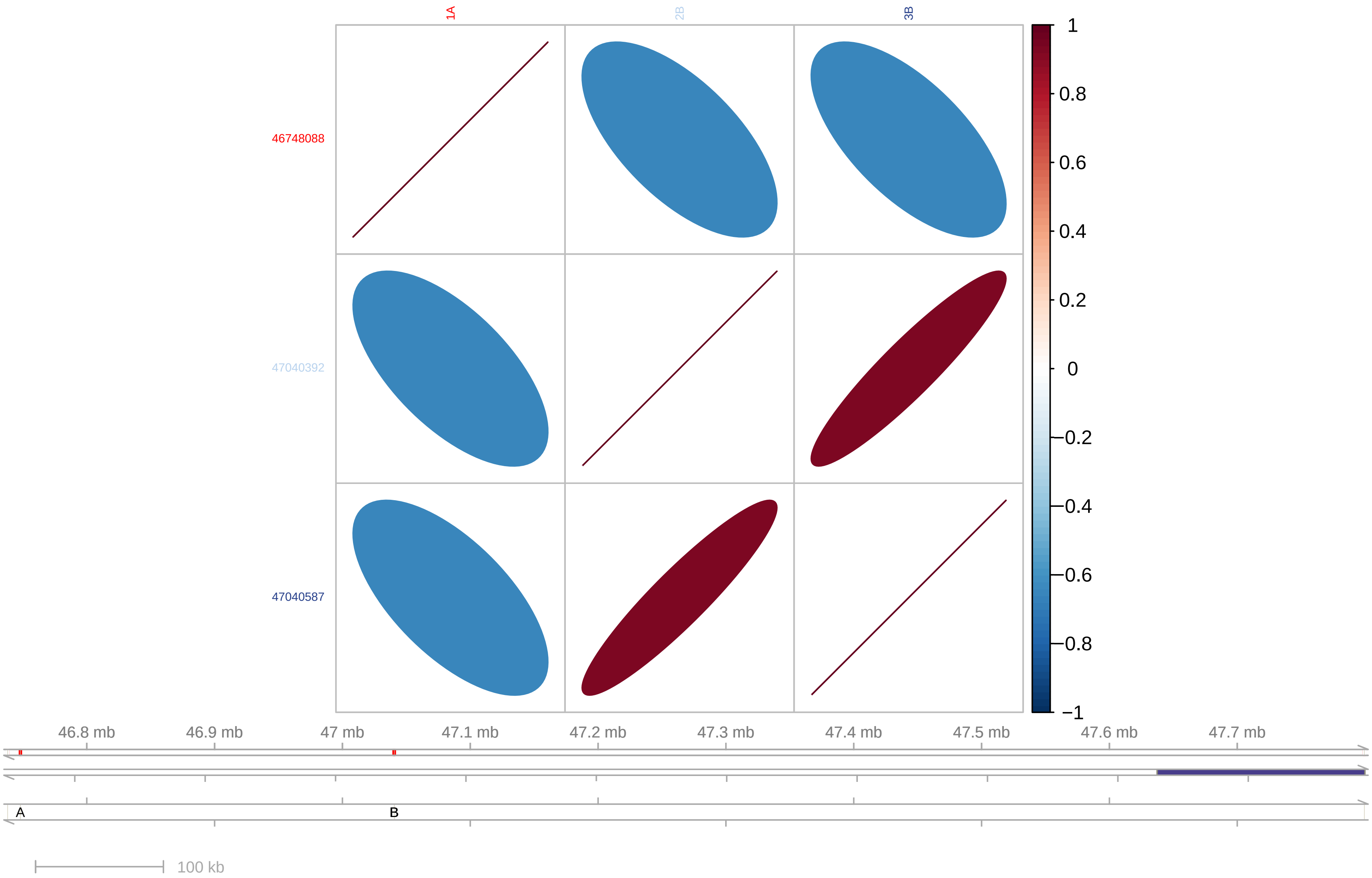
1A
2B
3B
46748088
47040392
47040587
1
0.8
0.6
0.4
0.2
0
−0.2
−0.4
−0.6
−0.8
−1
46.8 mb
46.9 mb
47 mb
47.1 mb
47.2 mb
47.3 mb
47.4 mb
47.5 mb
47.6 mb
47.7 mb
A
B
100 kb

MYD88

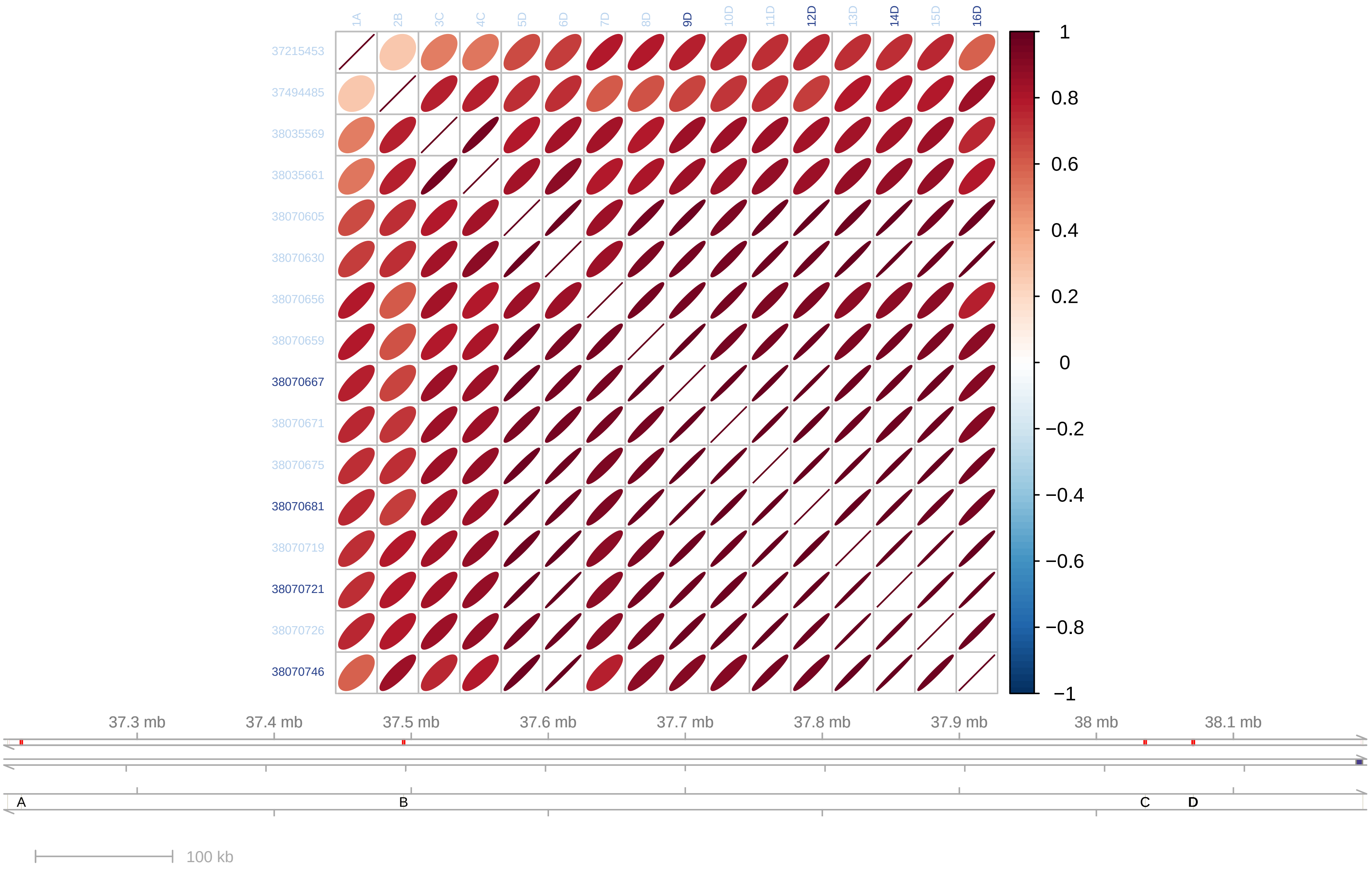

1A
2B
3C
4C
5D
6D
7D
8D
9D
10D
11D
12D
13D
14D
15D
16D
37215453
37494485
38035569
38035661
38070605
38070630
38070656
38070659
38070667
38070671
38070675
38070681
38070719
38070721
38070726
38070746
1
0.8
0.6
0.4
0.2
0
−0.2
−0.4
−0.6
−0.8
−1
37.3 mb
37.4 mb
37.5 mb
37.6 mb
37.7 mb
37.8 mb
37.9 mb
38 mb
38.1 mb
A
B
C
D
100 kb

NES

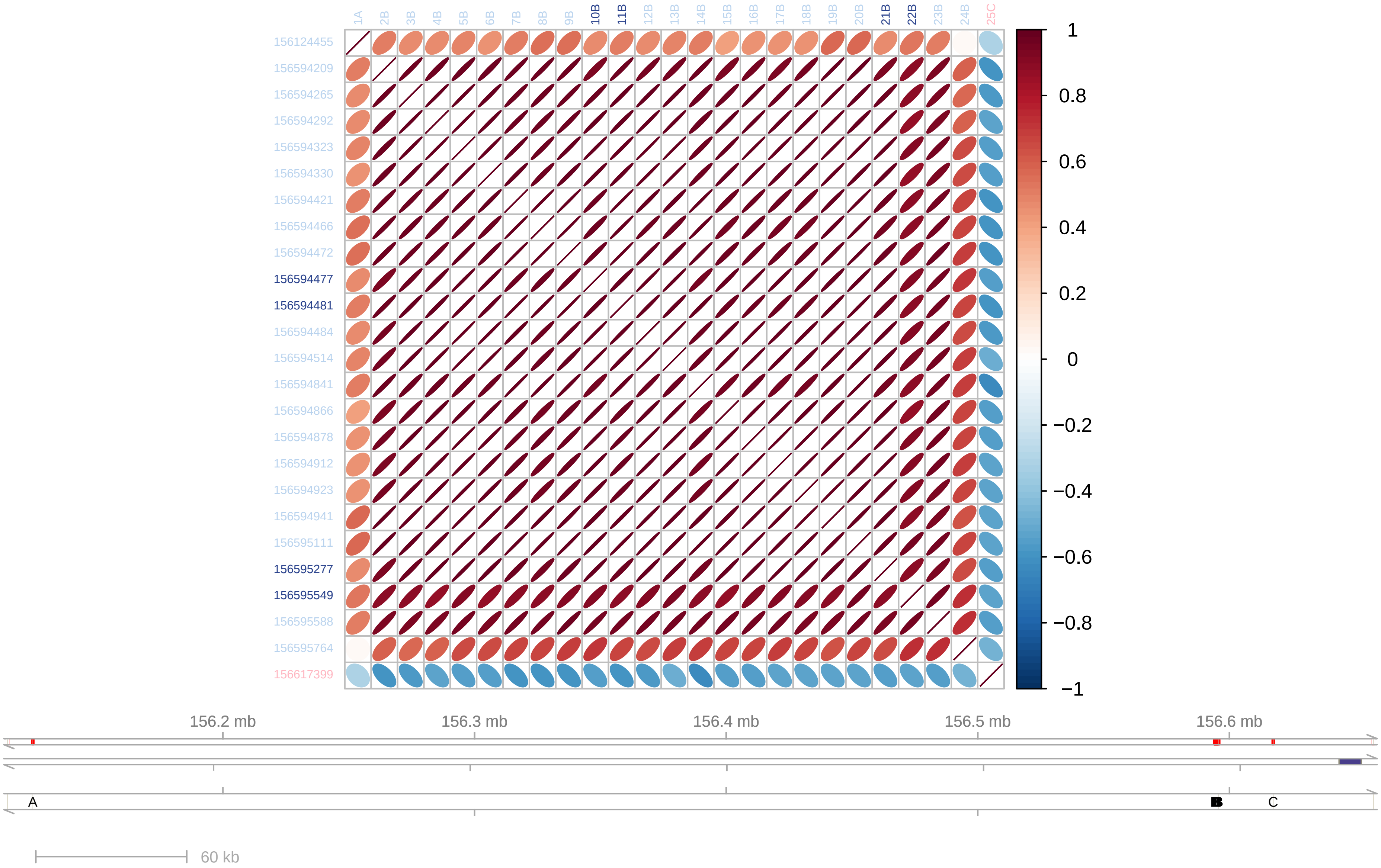
1A
2B
3B
4B
5B
6B
7B
8B
9B
10B
11B
12B
13B
14B
15B
16B
17B
18B
19B
20B
21B
22B
23B
24B
25C
156124455
156594209
156594265
156594292
156594323
156594330
156594421
156594466
156594472
156594477
156594481
156594484
156594514
156594841
156594866
156594878
156594912
156594923
156594941
156595111
156595277
156595549
156595588
156595764
156617399
1
0.8
0.6
0.4
0.2
0
−0.2
−0.4
−0.6
−0.8
−1
156.2 mb
156.3 mb
156.4 mb
156.5 mb
156.6 mb
A
B
C
60 kb

NOTCH1

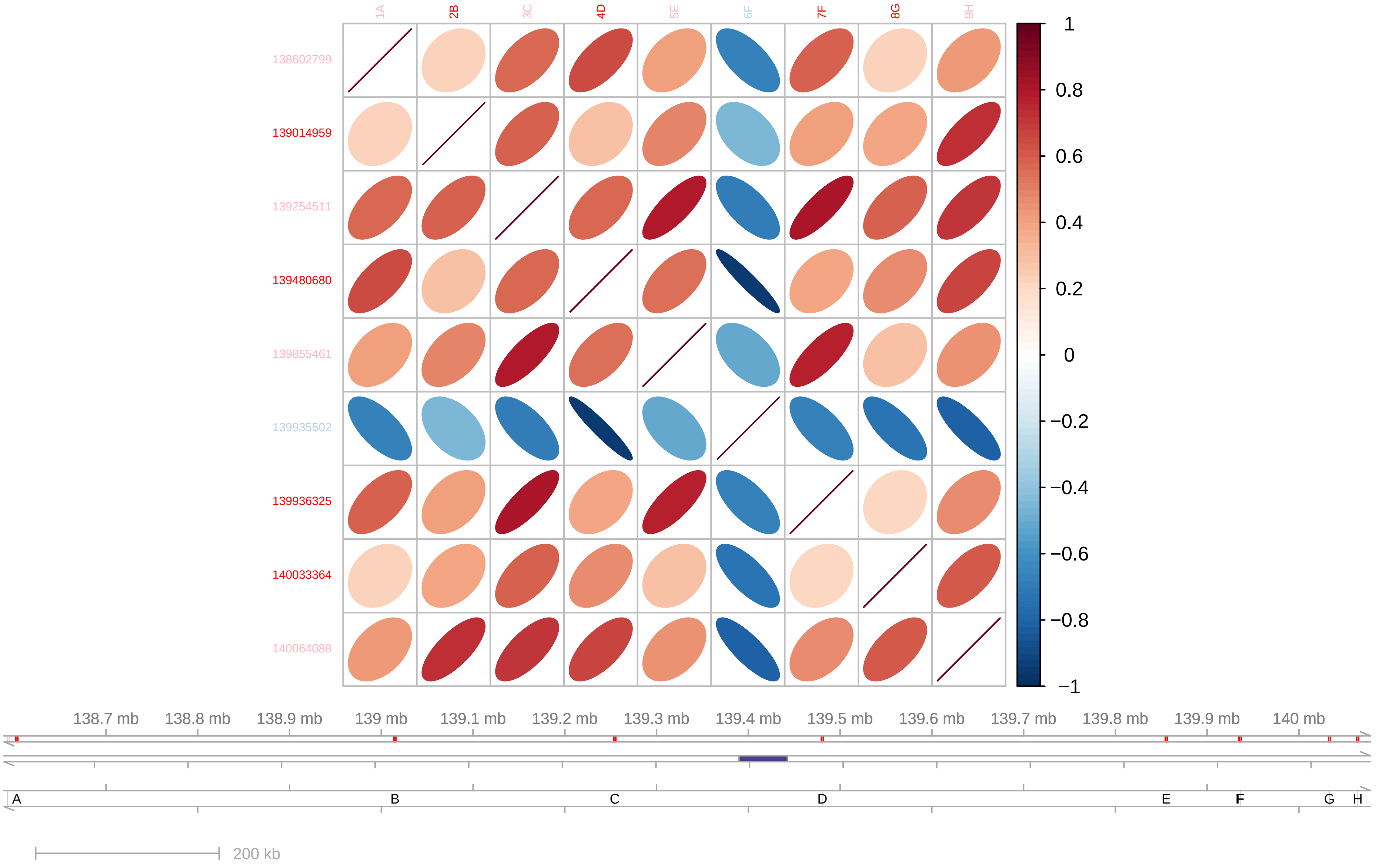

1A
2B
3C
4D
5E
6F
7F
8G
9H
138602799
139014959
139254511
139480680
139855461
139935502
139936325
140033364
140064088
1
0.8
0.6
0.4
0.2
0
−0.2
−0.4
−0.6
−0.8
−1
138.7 mb
138.8 mb
138.9 mb
139 mb
139.1 mb
139.2 mb
139.3 mb
139.4 mb
139.5 mb
139.6 mb
139.7 mb
139.8 mb
139.9 mb
140 mb
A
B
C
D
E
F
G
H
200 kb

OLIG2

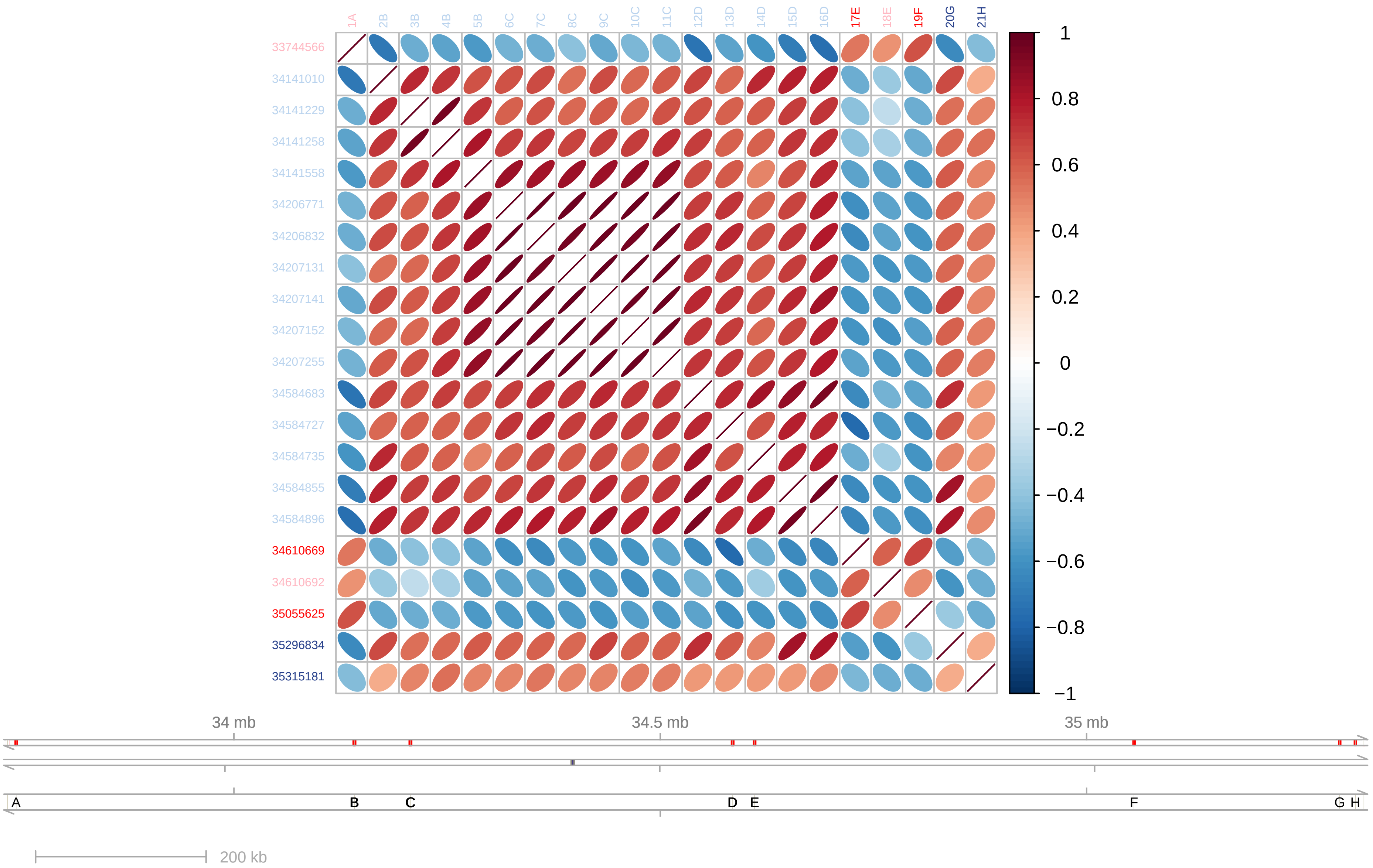

1A
2B
3B
4B
5B
6C
7C
8C
9C
10C
11C
12D
13D
14D
15D
16D
17E
18E
19F
20G
21H
33744566
34141010
34141229
34141258
34141558
34206771
34206832
34207131
34207141
34207152
34207255
34584683
34584727
34584735
34584855
34584896
34610669
34610692
35055625
35296834
35315181
1
0.8
0.6
0.4
0.2
0
−0.2
−0.4
−0.6
−0.8
−1
34 mb
34.5 mb
35 mb
A
B
C
D
E
F
G
H
200 kb

PBRM1

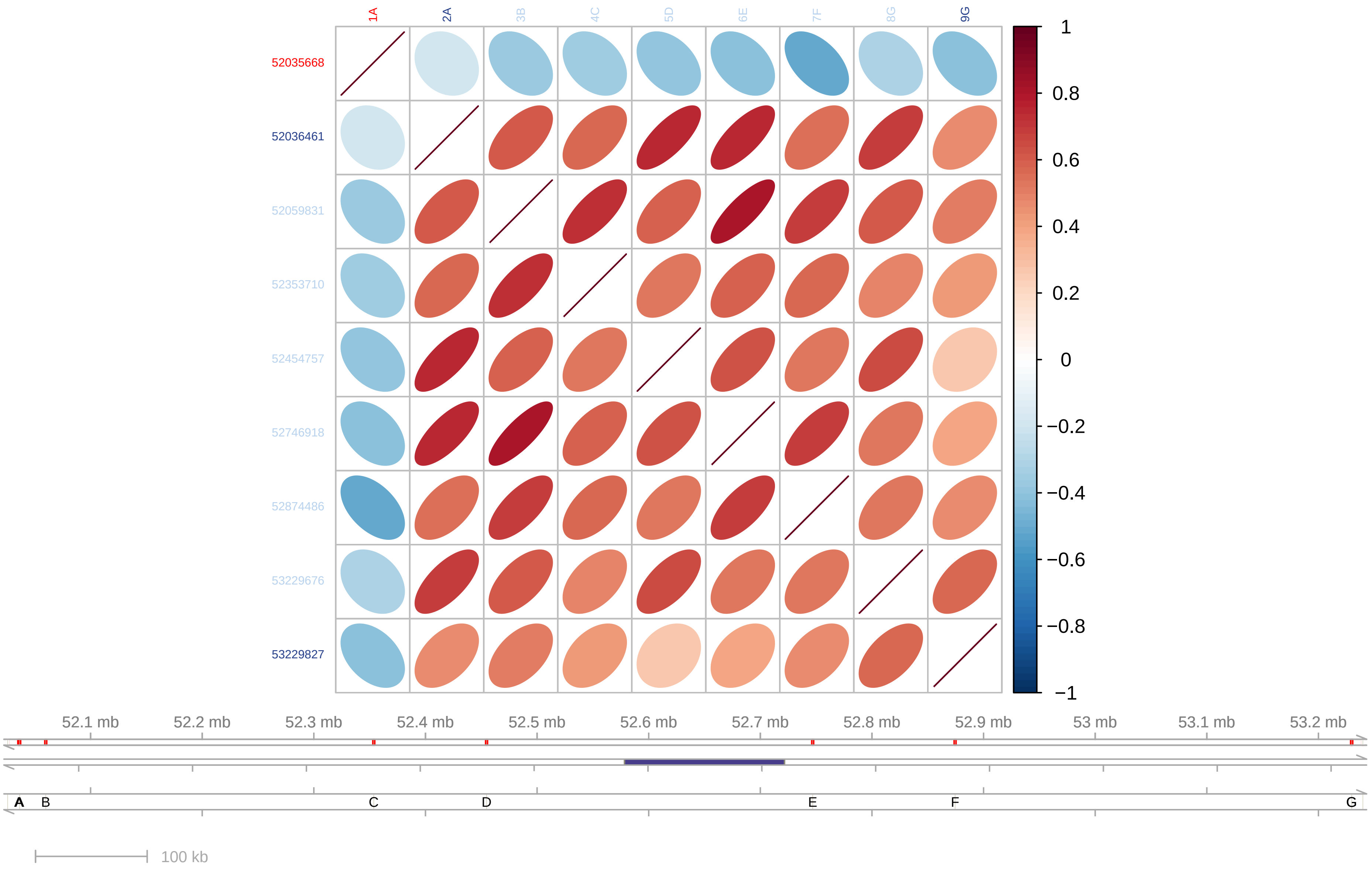

1A
2A
3B
4C
5D
6E
7F
8G
9G
52035668
52036461
52059831
52353710
52454757
52746918
52874486
53229676
53229827
1
0.8
0.6
0.4
0.2
0
−0.2
−0.4
−0.6
−0.8
−1
52.1 mb
52.2 mb
52.3 mb
52.4 mb
52.5 mb
52.6 mb
52.7 mb
52.8 mb
52.9 mb
53 mb
53.1 mb
53.2 mb
A
B
C
D
E
F
G
100 kb

PDGFA

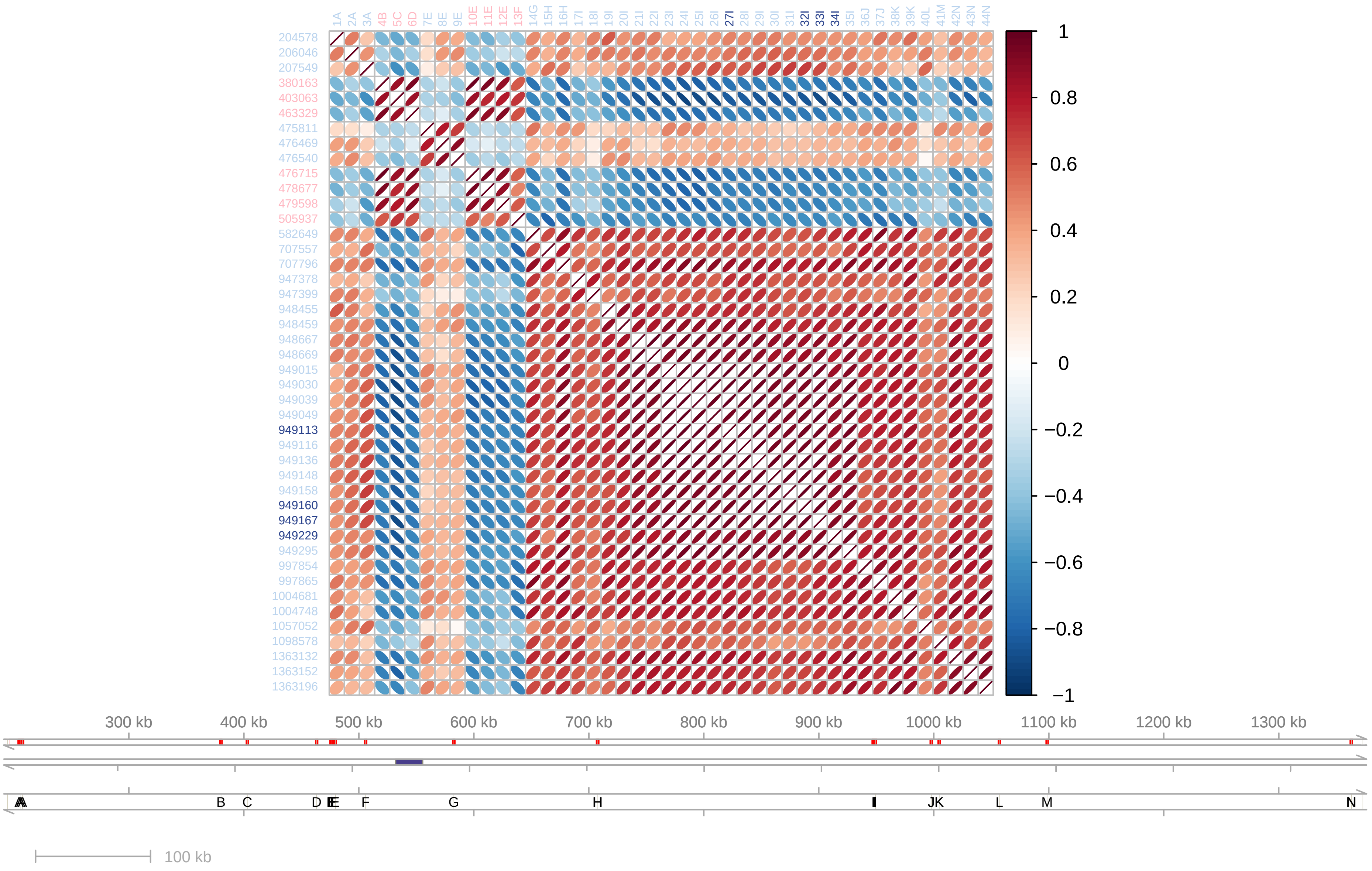

1A 2A 3A 4B 5C 6D 7E 8E 9E 10E 11E 12E 13F 14G 15H 16H 17I 18I 19I 20I 21I 22I 23I 24I 25I 26I 27I 28I 29I 30I 31I 32I 33I 34I 35I 36J 37J 38K 39K 40L 41M 42N 43N 44N
204578
206046
207549
380163
403063
463329
475811
476469
476540
476715
478677
479598
505937
582649
707557
707796
947378
947399
948455
948459
948667
948669
949015
949030
949039
949049
949113
949116
949136
949148
949158
949160
949167
949229
949295
997854
997865
1004681
1004748
1057052
1098578
1363132
1363152
1363196
1
0.8
0.6
0.4
0.2
0
−0.2
−0.4
−0.6
−0.8
−1
300 kb
400 kb
500 kb
600 kb
700 kb
800 kb
900 kb
1000 kb
1100 kb
1200 kb
1300 kb
A
B
C
D
E
F
G
H
I
JK
L
M
N
100 kb

PDGFRA

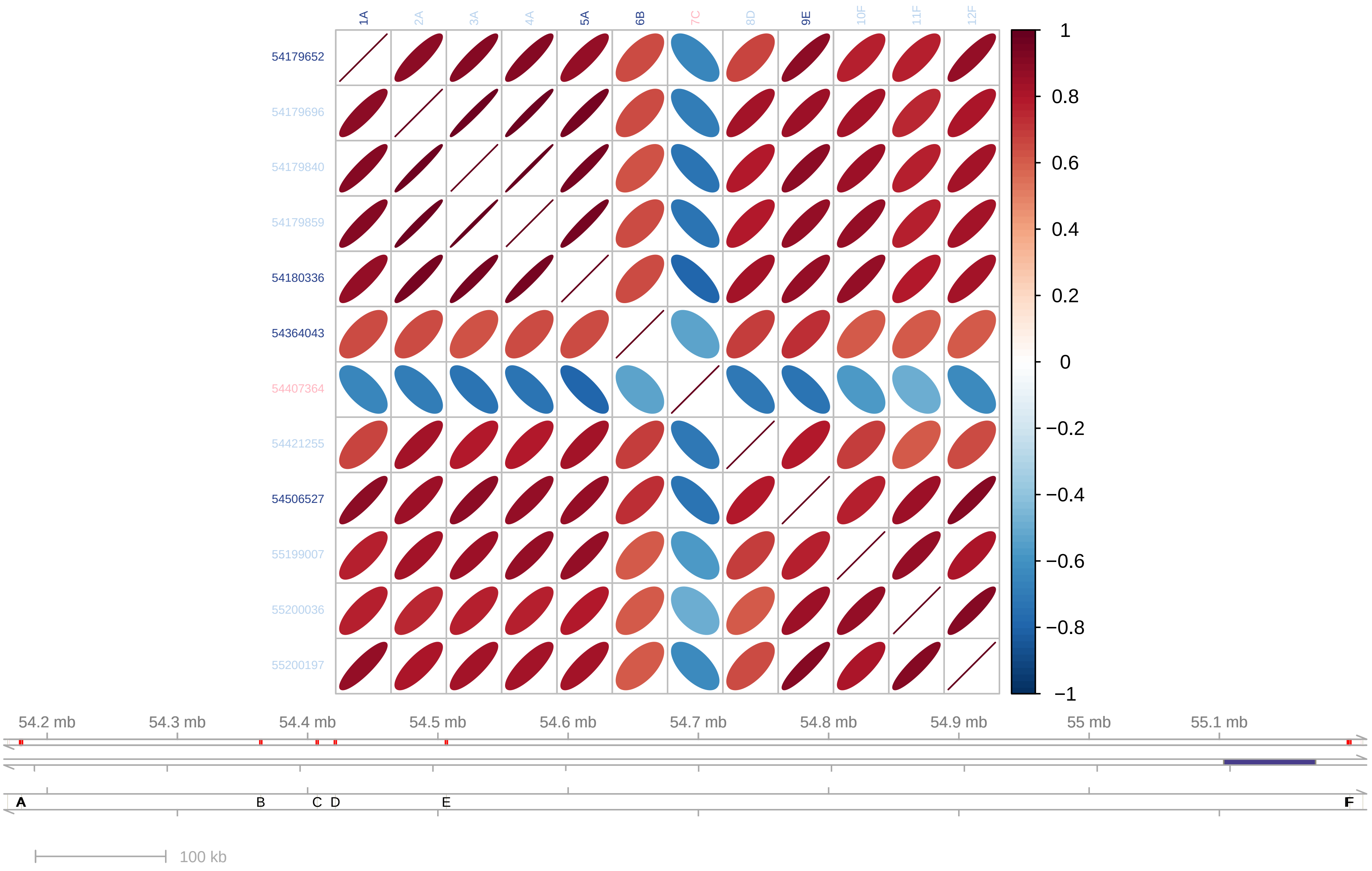
1A
2A
3A
4A
5A
6B
7C
8D
9E
10F
11F
12F
54179652
54179696
54179840
54179859
54180336
54364043
54407364
54421255
54506527
55199007
55200036
55200197
1
0.8
0.6
0.4
0.2
0
−0.2
−0.4
−0.6
−0.8
−1
54.2 mb
54.3 mb
54.4 mb
54.5 mb
54.6 mb
54.7 mb
54.8 mb
54.9 mb
55 mb
55.1 mb
A
B
C
D
E
F
100 kb

PRDM1

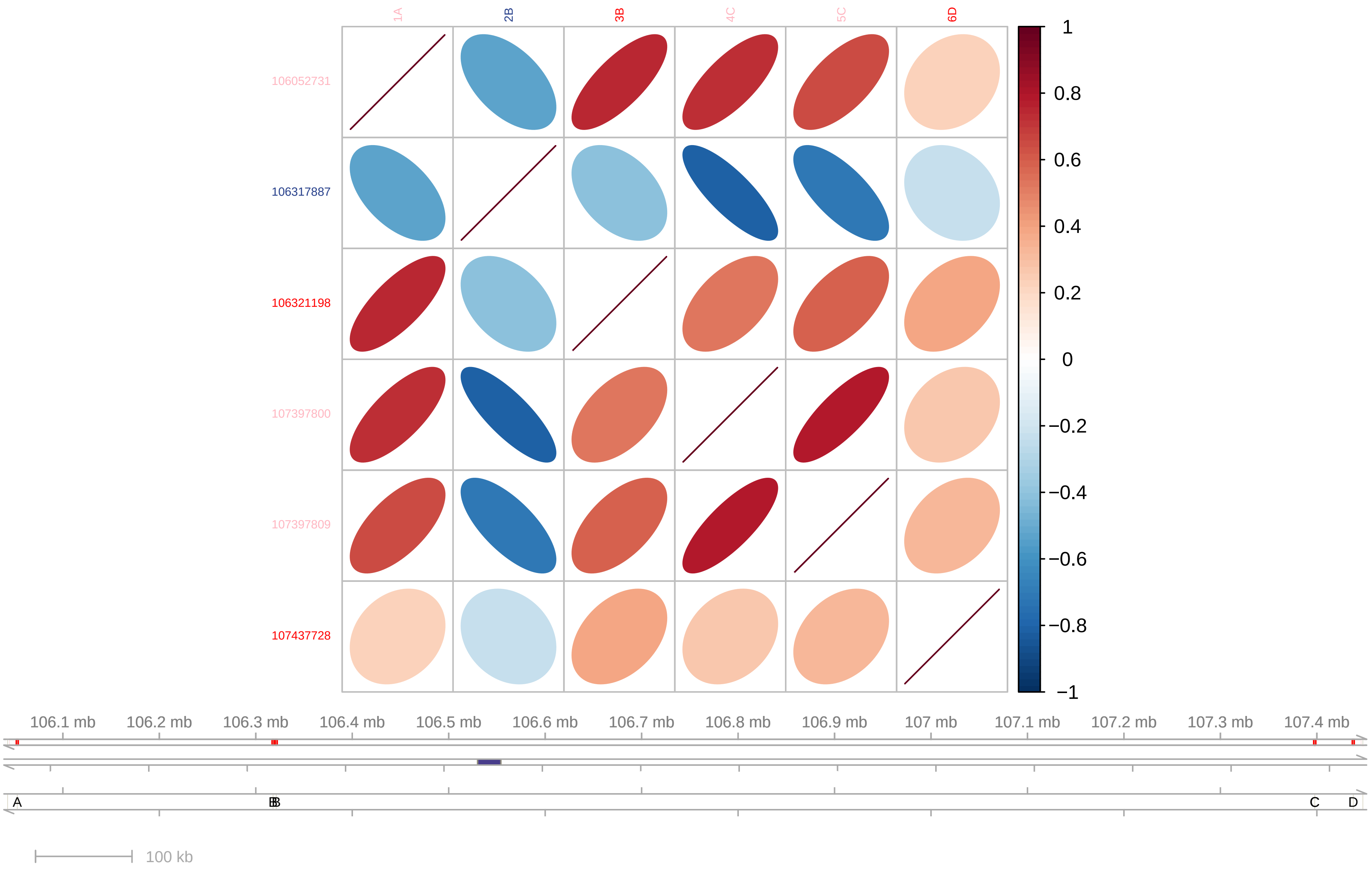

1A
2B
3B
4C
5C
6D
106052731
106317887
106321198
107397800
107397809
107437728
1
0.8
0.6
0.4
0.2
0
−0.2
−0.4
−0.6
−0.8
−1
106.1 mb
106.2 mb
106.3 mb
106.4 mb
106.5 mb
106.6 mb
106.7 mb
106.8 mb
106.9 mb
107 mb
107.1 mb
107.2 mb
107.3 mb
107.4 mb
A
B
C
D
100 kb

PTEN

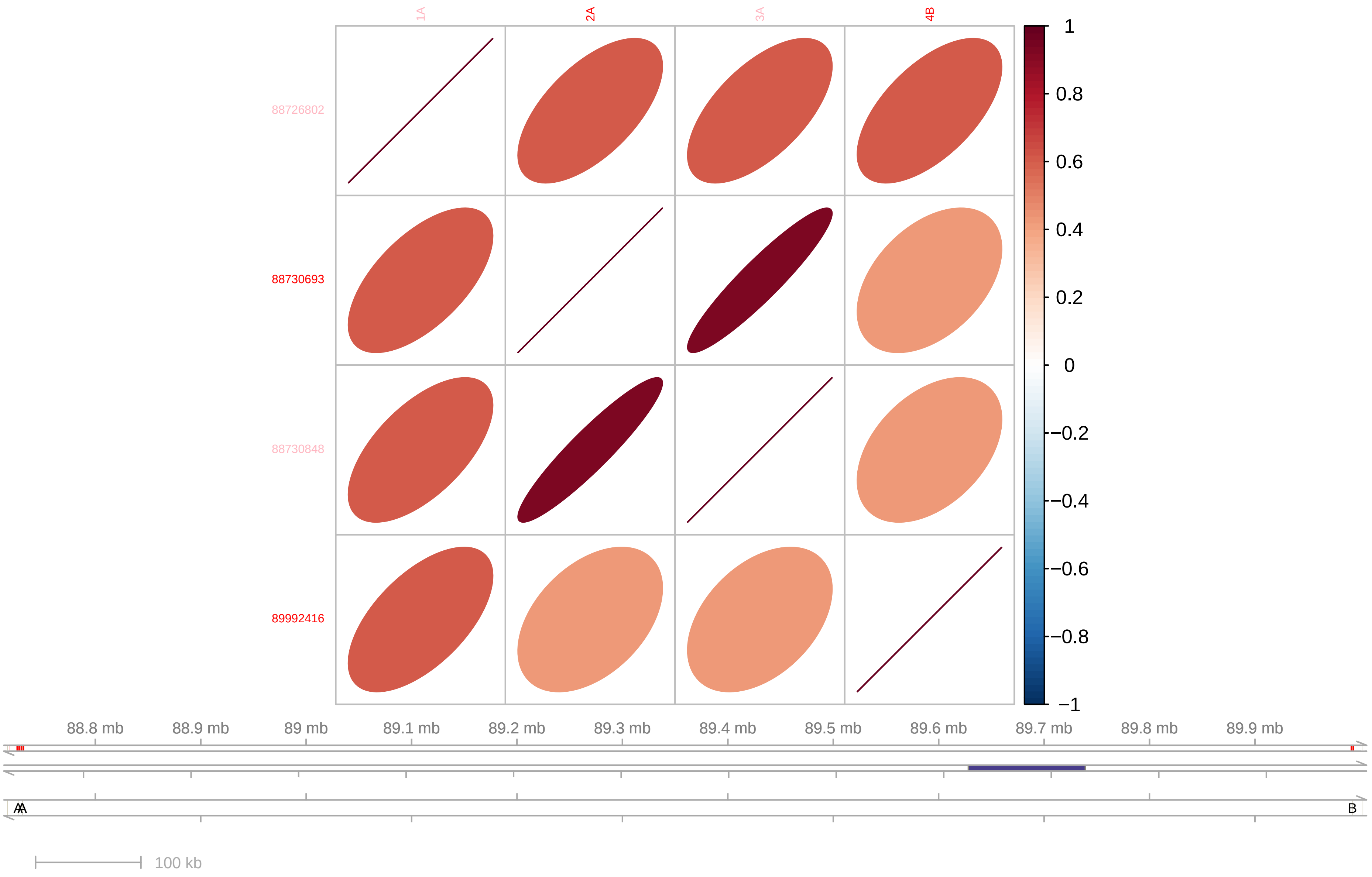
1A
2A
3A
4B
88726802
88730693
88730848
89992416
1
0.8
0.6
0.4
0.2
0
−0.2
−0.4
−0.6
−0.8
−1
88.8 mb
88.9 mb
89 mb
89.1 mb
89.2 mb
89.3 mb
89.4 mb
89.5 mb
89.6 mb
89.7 mb
89.8 mb
89.9 mb
A
B
100 kb

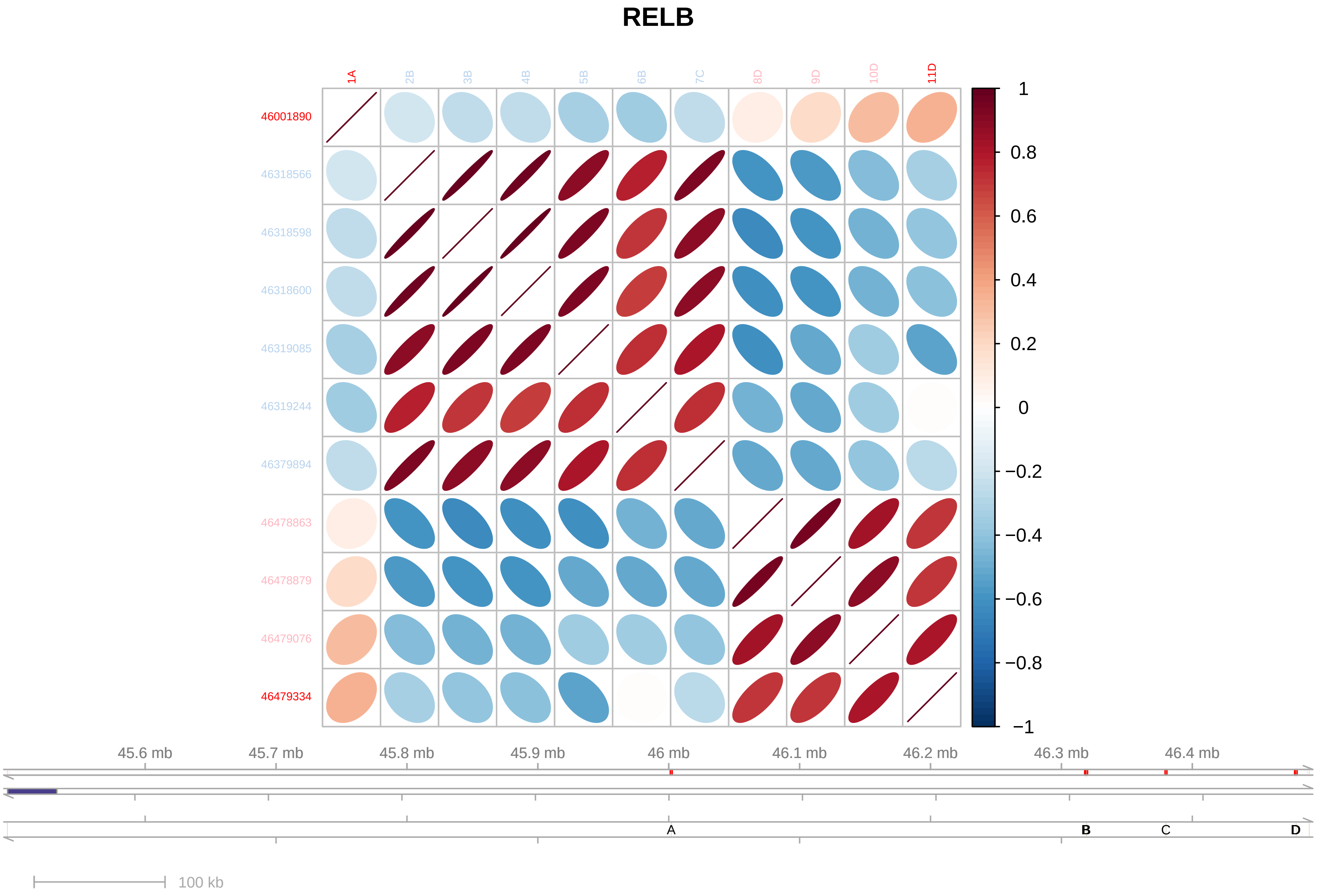
RELB
1A
2B
3B
4B
5B
6B
7C
8D
9D
10D
11D
46001890
46318566
46318598
46318600
46319085
46319244
46379894
46478863
46478879
46479076
46479334
1
0.8
0.6
0.4
0.2
0
−0.2
−0.4
−0.6
−0.8
−1
45.6 mb
45.7 mb
45.8 mb
45.9 mb
46 mb
46.1 mb
46.2 mb
46.3 mb
46.4 mb
A
B
C
D
100 kb

RNF43

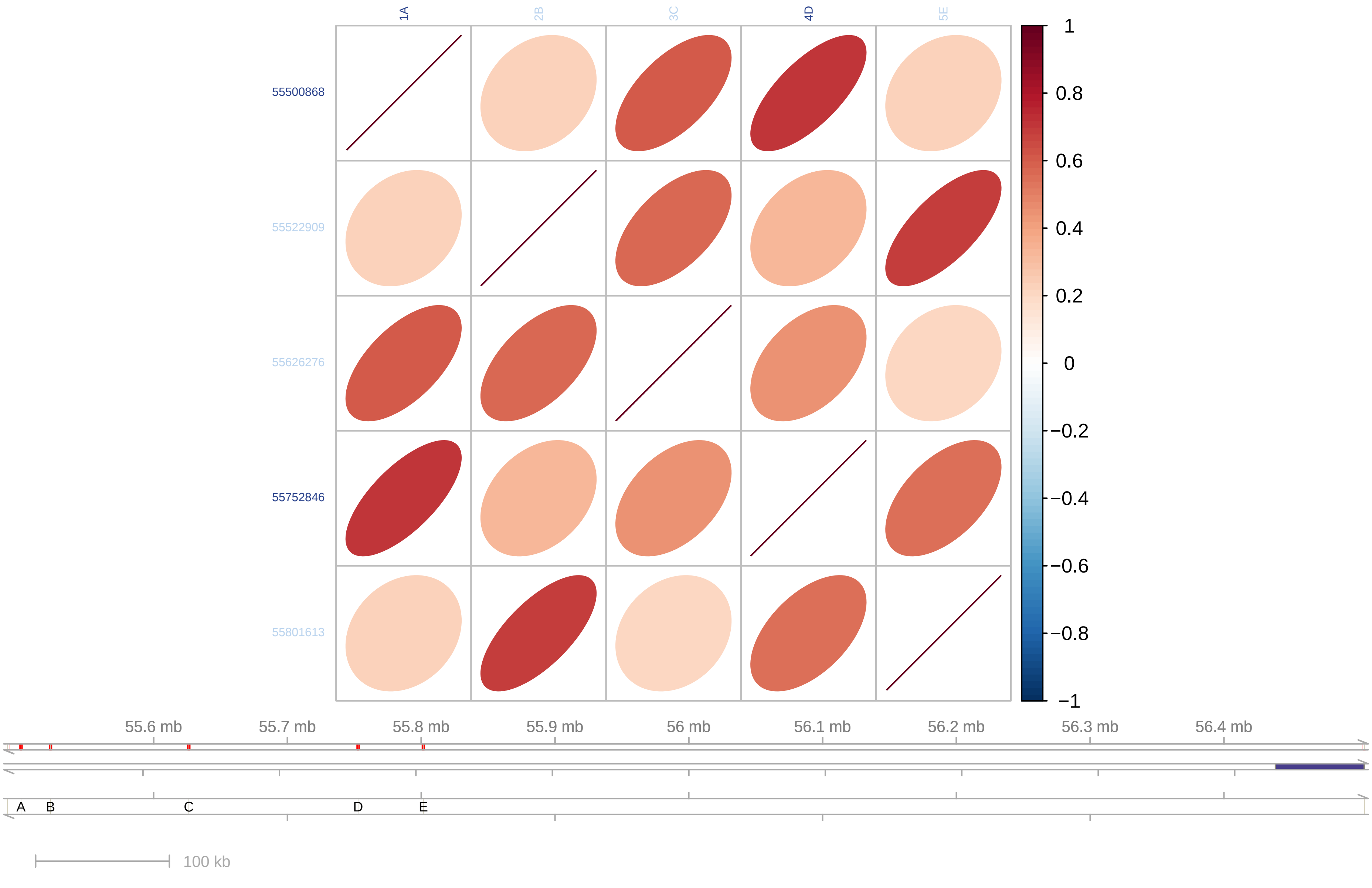
1A
2B
3C
4D
5E
55500868
55522909
55626276
55752846
55801613
1
0.8
0.6
0.4
0.2
0
−0.2
−0.4
−0.6
−0.8
−1
55.6 mb
55.7 mb
55.8 mb
55.9 mb
56 mb
56.1 mb
56.2 mb
56.3 mb
56.4 mb
A
B
C
D
E
100 kb

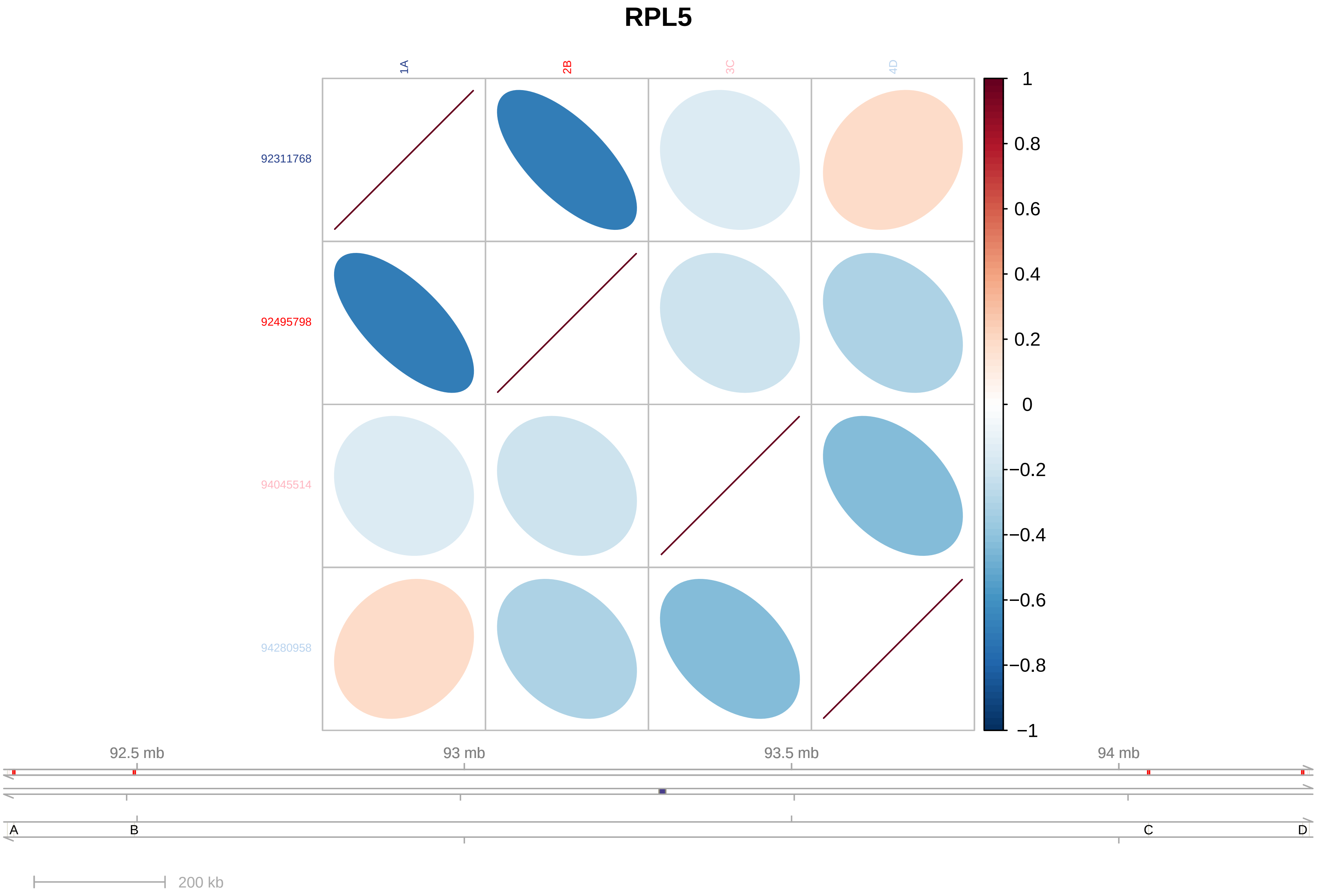

RPL5
1A
2B
3C
4D
92311768
92495798
94045514
94280958
1
0.8
0.6
0.4
0.2
0
−0.2
−0.4
−0.6
−0.8
−1
92.5 mb
93 mb
93.5 mb
94 mb
A
B
C
D
200 kb

SGCD

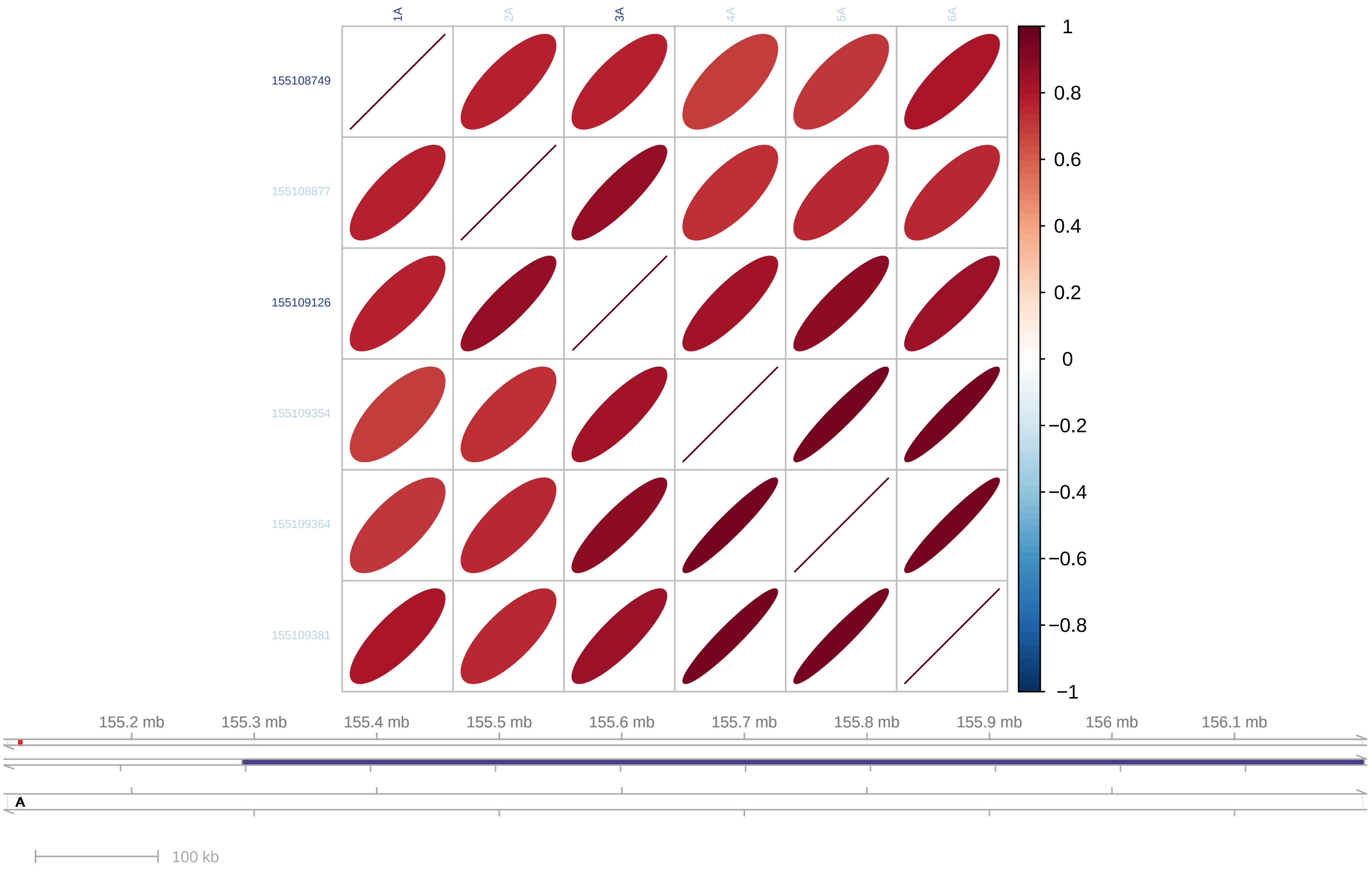
1A
2A
3A
4A
5A
6A
155108749
155108877
155109126
155109354
155109364
155109381
1
0.8
0.6
0.4
0.2
0
−0.2
−0.4
−0.6
−0.8
−1
155.2 mb
155.3 mb
155.4 mb
155.5 mb
155.6 mb
155.7 mb
155.8 mb
155.9 mb
156 mb
156.1 mb
A
100 kb

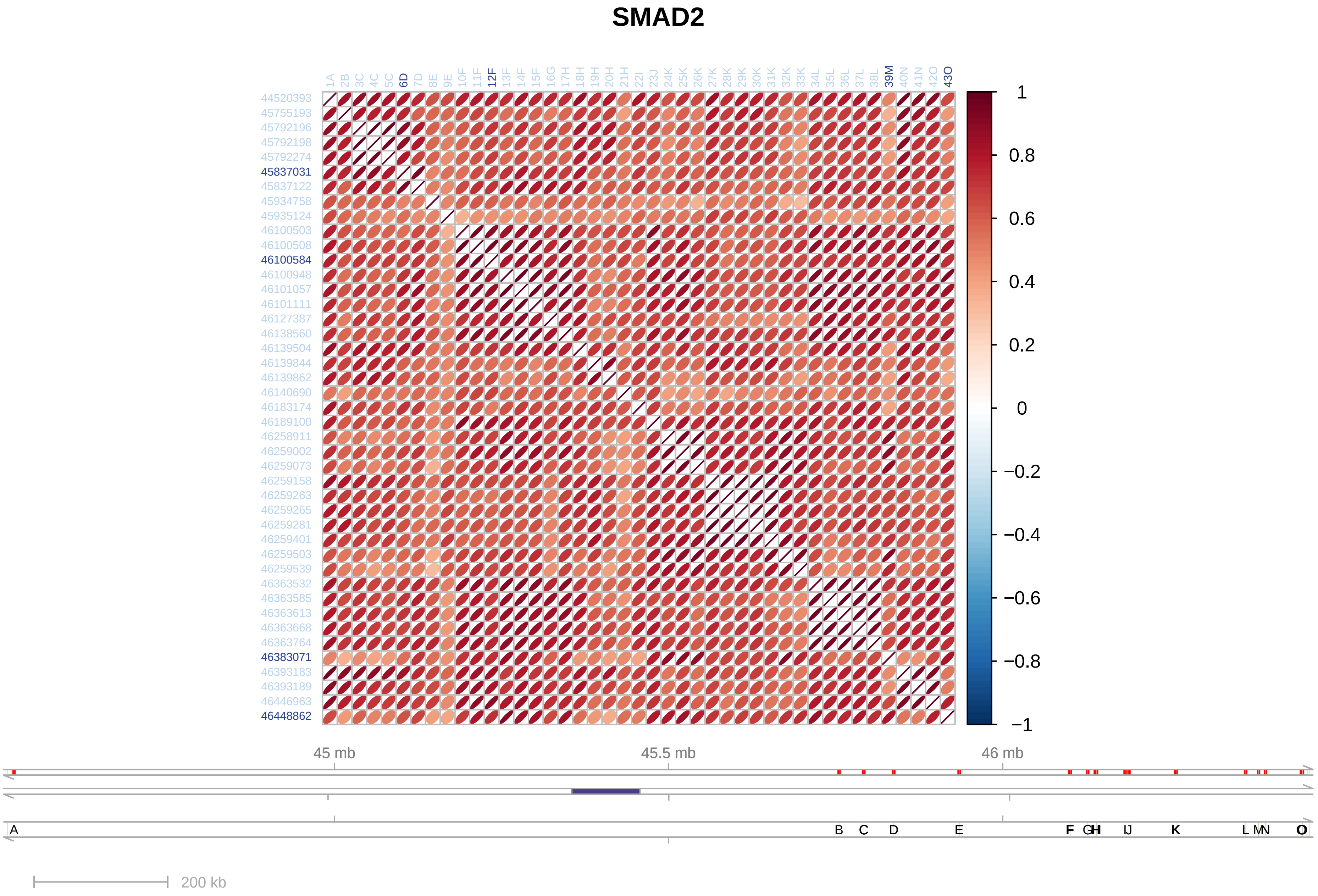
SMAD2
1A 2B 3C 4C 5C 6D 7D 8E 9E 10F 11F 12F 13F 14F 15F 16G 17H 18H 19H 20H 21H 22I 23J 24K 25K 26K 27K 28K 29K 30K 31K 32K 33K 34L 35L 36L 37L 38L 39M 40N 41N 42O 43O
44520393
45755193
45792196
45792198
45792274
45837031
45837122
45934758
45935124
46100503
46100508
46100584
46100948
46101057
46101111
46127387
46138560
46139504
46139844
46139862
46140690
46183174
46189100
46258911
46259002
46259073
46259158
46259263
46259265
46259281
46259401
46259503
46259539
46363532
46363585
46363613
46363668
46363764
46383071
46393183
46393189
46446963
46448862
1 0.8 0.6 0.4 0.2 0 −0.2 −0.4 −0.6 −0.8 −1
45 mb
45.5 mb
46 mb
A B C D E F GH IJ K L MN O
200 kb

SMAD4

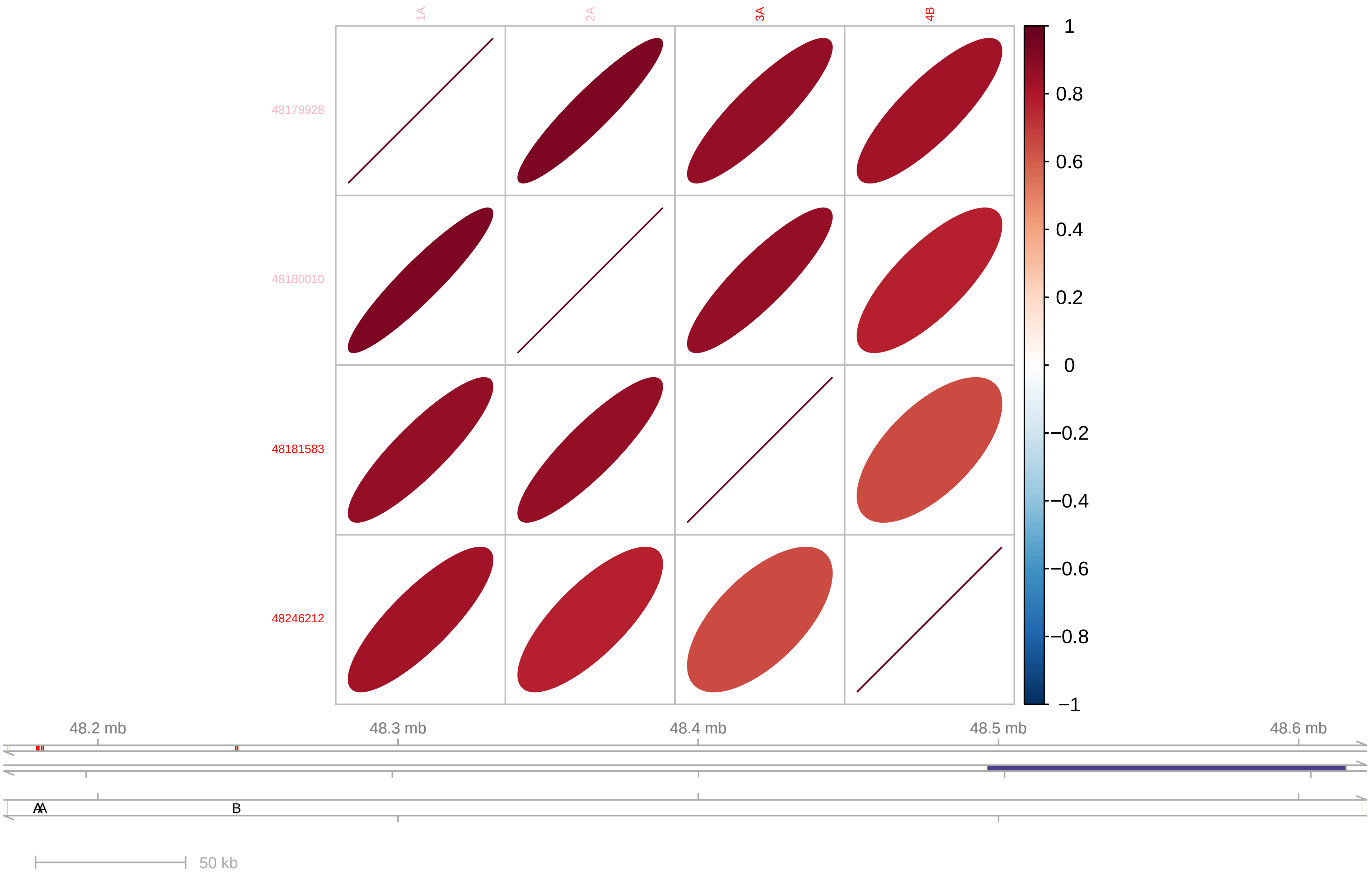

1A
2A
3A
4B
48179928
48180010
48181583
48246212
1
0.8
0.6
0.4
0.2
0
−0.2
−0.4
−0.6
−0.8
−1
48.2 mb
48.3 mb
48.4 mb
48.5 mb
48.6 mb
A
A
B
50 kb

SMARCB1

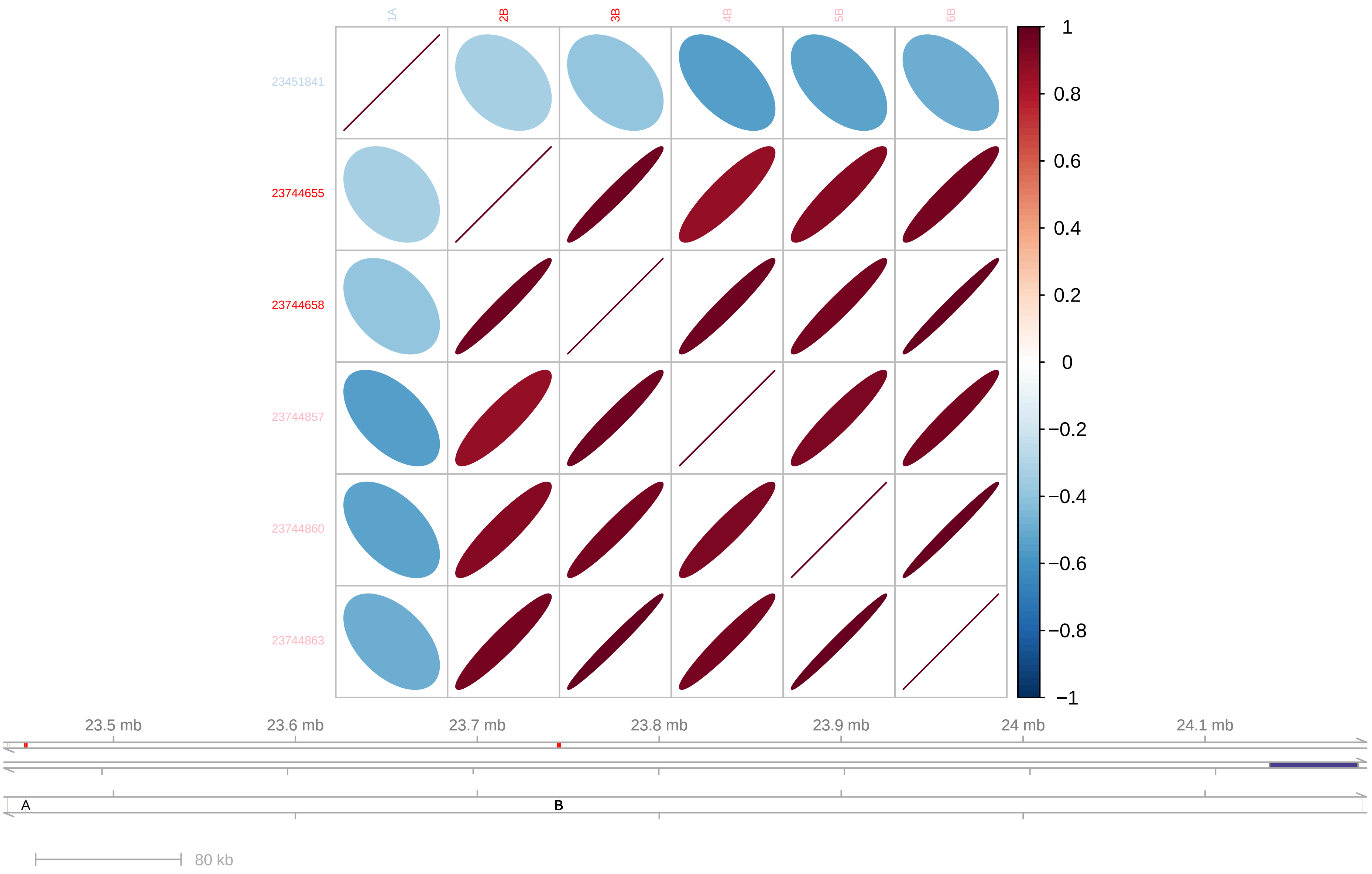

1A
2B
3B
4B
5B
6B
23451841
23744655
23744658
23744857
23744860
23744863
1
0.8
0.6
0.4
0.2
0
−0.2
−0.4
−0.6
−0.8
−1
23.5 mb
23.6 mb
23.7 mb
23.8 mb
23.9 mb
24 mb
24.1 mb
A
B
80 kb

SMO

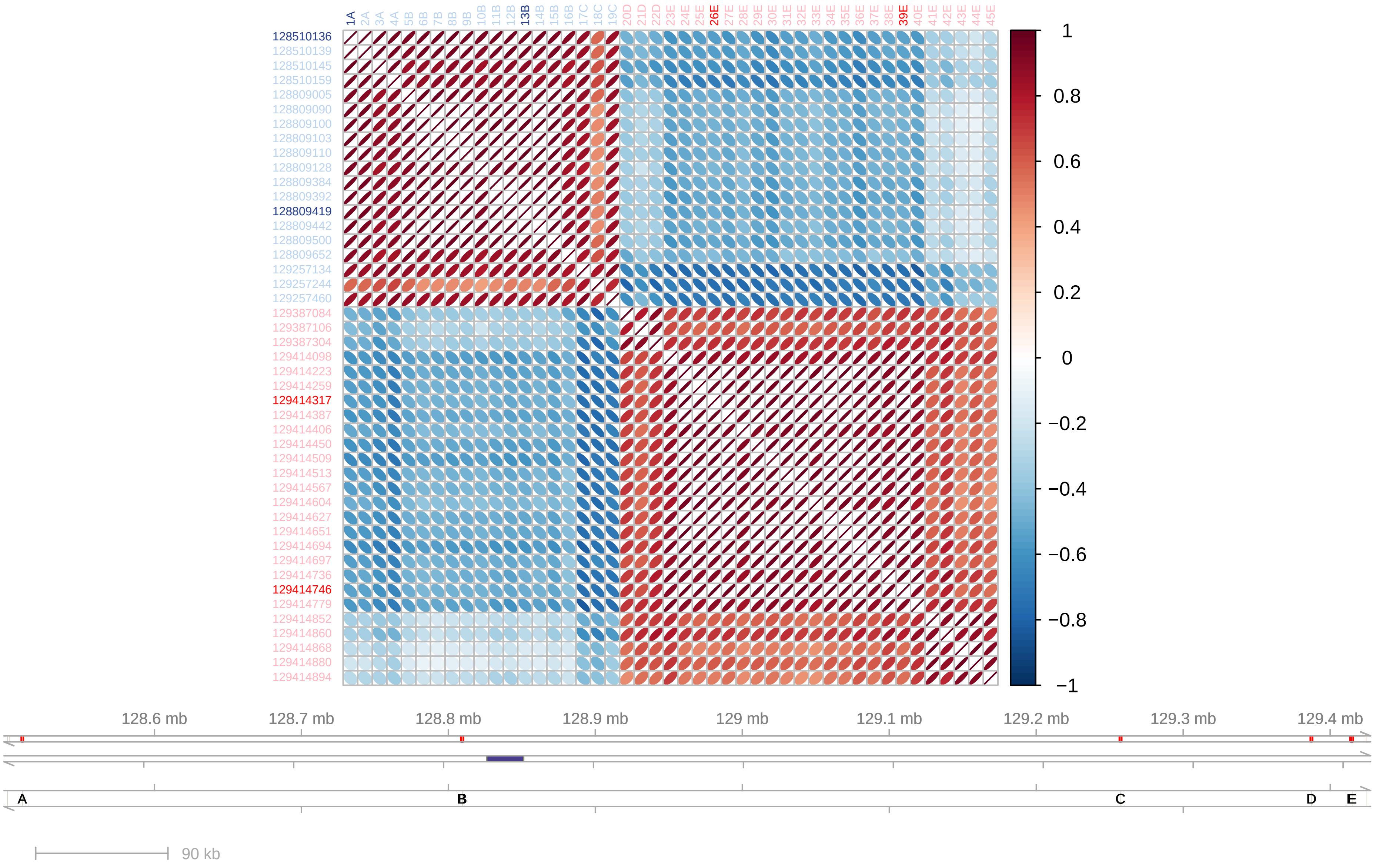

1A 2A 3A 4A 5B 6B 7B 8B 9B 10B 11B 12B 13B 14B 15B 16B 17C 18C 19C 20D 21D 22D 23E 24E 25E 26E 27E 28E 29E 30E 31E 32E 33E 34E 35E 36E 37E 38E 39E 40E 41E 42E 43E 44E 45E
128510136
128510139
128510145
128510159
128809005
128809090
128809100
128809103
128809110
128809128
128809384
128809392
128809419
128809442
128809500
128809652
129257134
129257244
129257460
129387084
129387106
129387304
129414098
129414223
129414259
129414317
129414387
129414406
129414450
129414509
129414513
129414567
129414604
129414627
129414651
129414694
129414697
129414736
129414746
129414779
129414852
129414860
129414868
129414880
129414894
1
0.8
0.6
0.4
0.2
0
−0.2
−0.4
−0.6
−0.8
−1
128.6 mb
128.7 mb
128.8 mb
128.9 mb
129 mb
129.1 mb
129.2 mb
129.3 mb
129.4 mb
A
B
C
D
E
90 kb

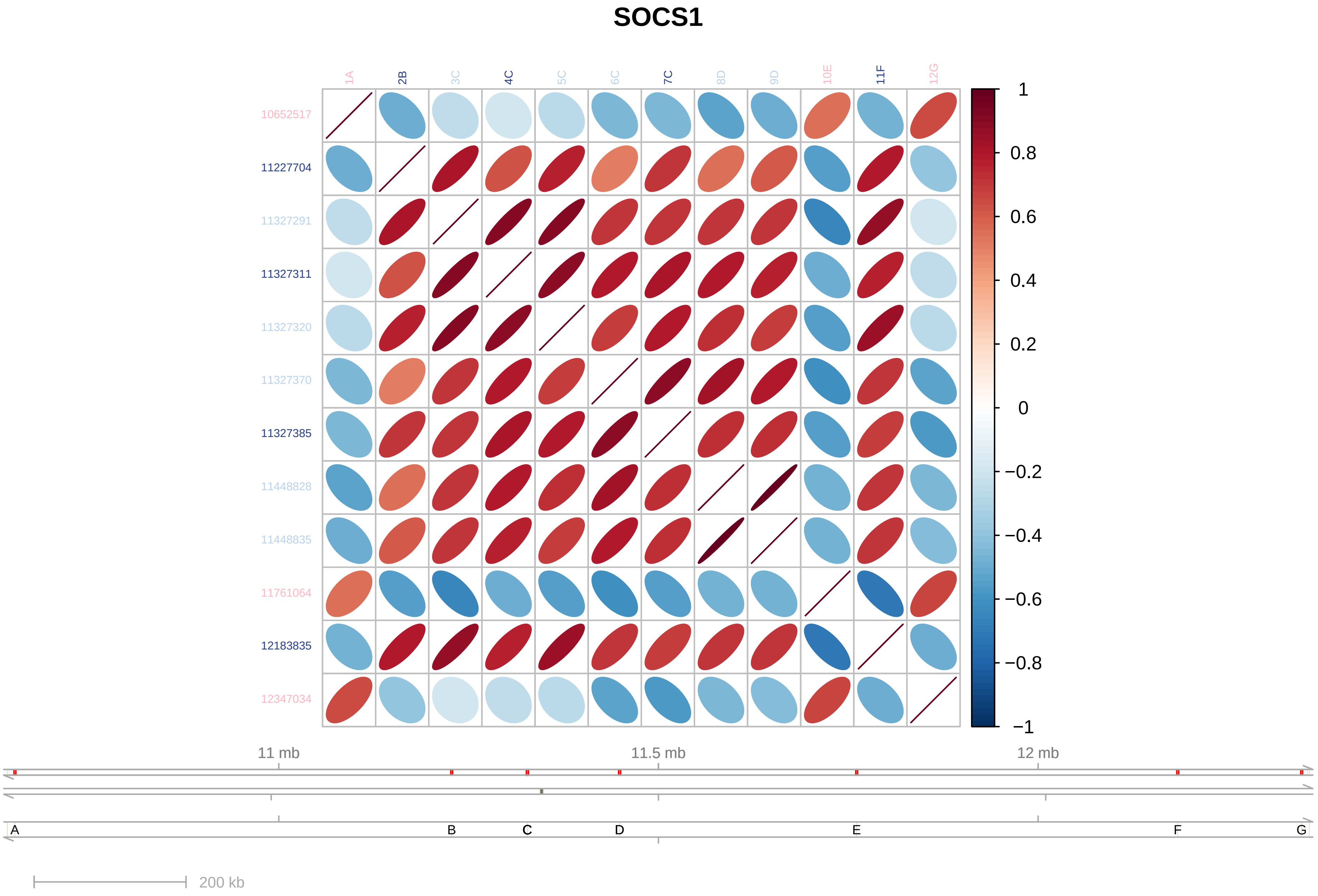

SOCS1
1A
2B
3C
4C
5C
6C
7C
8D
9D
10E
11F
12G
10652517
11227704
11327291
11327311
11327320
11327370
11327385
11448828
11448835
11761064
12183835
12347034
1
0.8
0.6
0.4
0.2
0
−0.2
−0.4
−0.6
−0.8
−1
11 mb
11.5 mb
12 mb
A
B
C
D
E
F
G
200 kb

SOX10

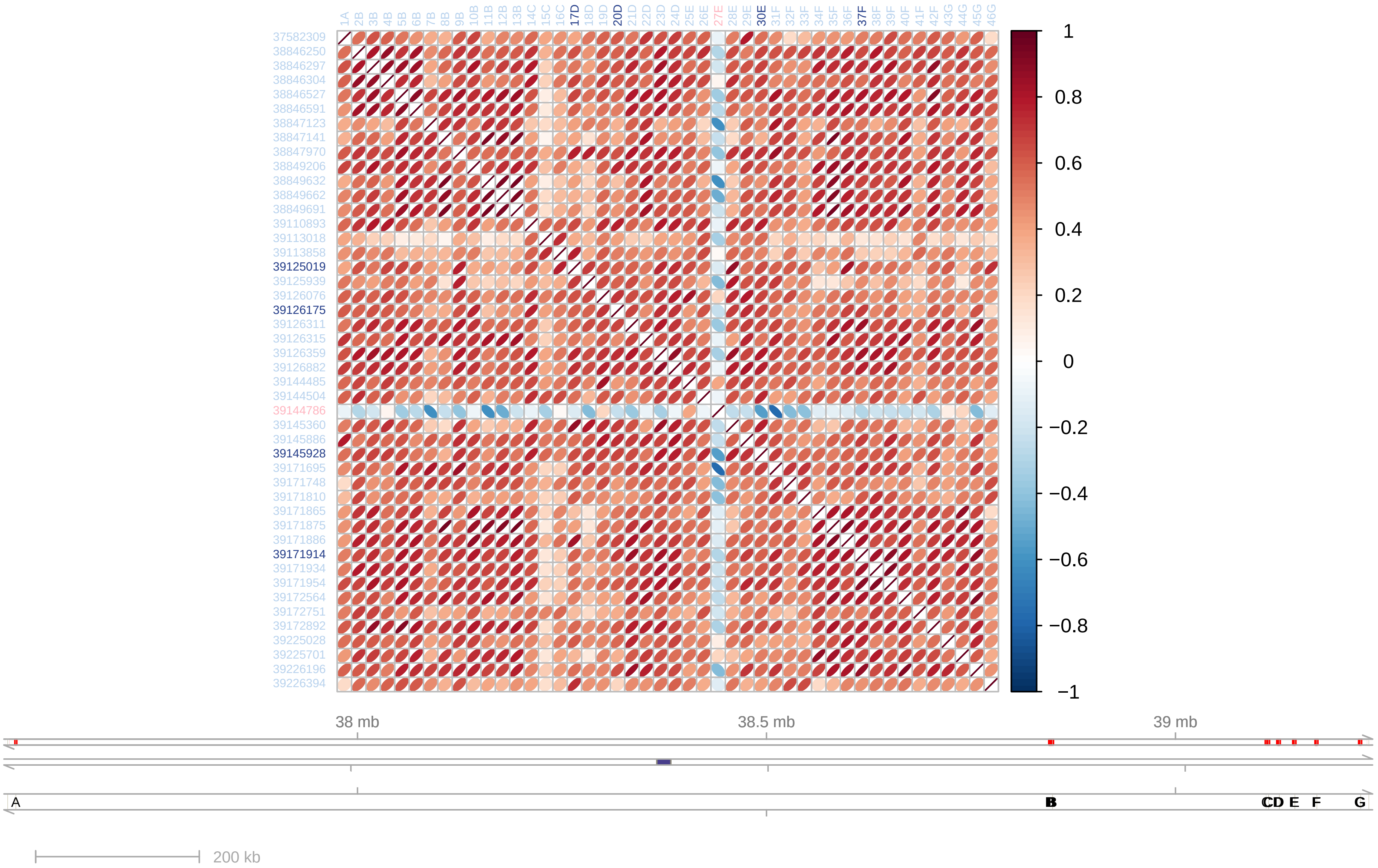

1A
2B
3B
4B
5B
6B
7B
8B
9B
10B
11B
12B
13B
14C
15C
16C
17D
18D
19D
20D
21D
22D
23D
24D
25E
26E
27E
28E
29E
30E
31F
32F
33F
34F
35F
36F
37F
38F
39F
40F
41F
42F
43G
44G
45G
46G
37582309
38846250
38846297
38846304
38846527
38846591
38847123
38847141
38847970
38849206
38849632
38849662
38849691
39110893
39113018
39113858
39125019
39125939
39126076
39126175
39126311
39126315
39126359
39126882
39144485
39144504
39144786
39145360
39145886
39145928
39171695
39171748
39171810
39171865
39171875
39171886
39171914
39171934
39171954
39172564
39172751
39172892
39225028
39225701
39226196
39226394
1
0.8
0.6
0.4
0.2
0
−0.2
−0.4
−0.6
−0.8
−1
38 mb
38.5 mb
39 mb
A
B
CD
E
F
G
200 kb

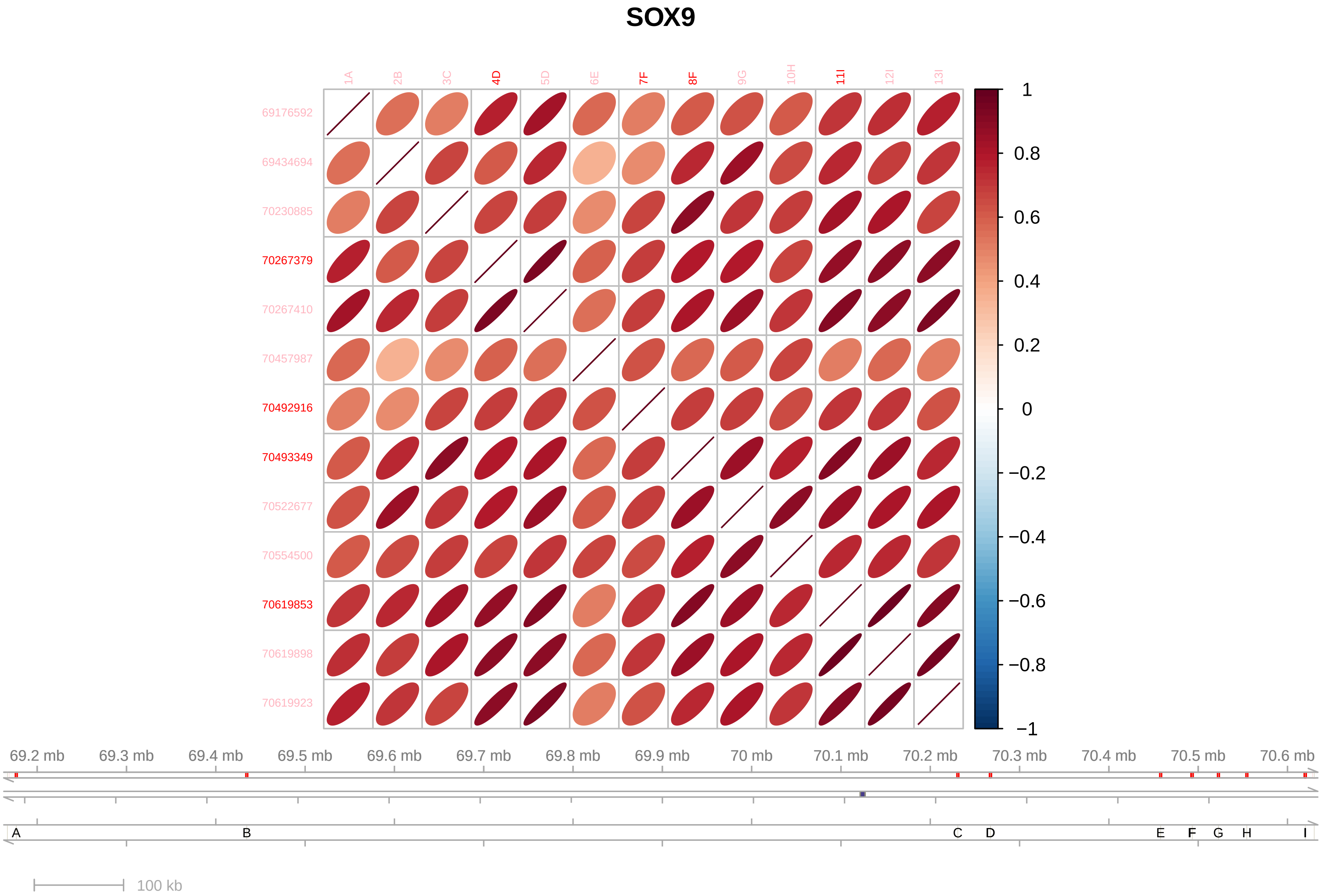
SOX9
1A
2B
3C
4D
5D
6E
7F
8F
9G
10H
11I
12I
13I
69176592
69434694
70230885
70267379
70267410
70457987
70492916
70493349
70522677
70554500
70619853
70619898
70619923
1
0.8
0.6
0.4
0.2
0
−0.2
−0.4
−0.6
−0.8
−1
69.2 mb
69.3 mb
69.4 mb
69.5 mb
69.6 mb
69.7 mb
69.8 mb
69.9 mb
70 mb
70.1 mb
70.2 mb
70.3 mb
70.4 mb
70.5 mb
70.6 mb
A
B
C
D
E
F
G
H
I
100 kb

SPOP

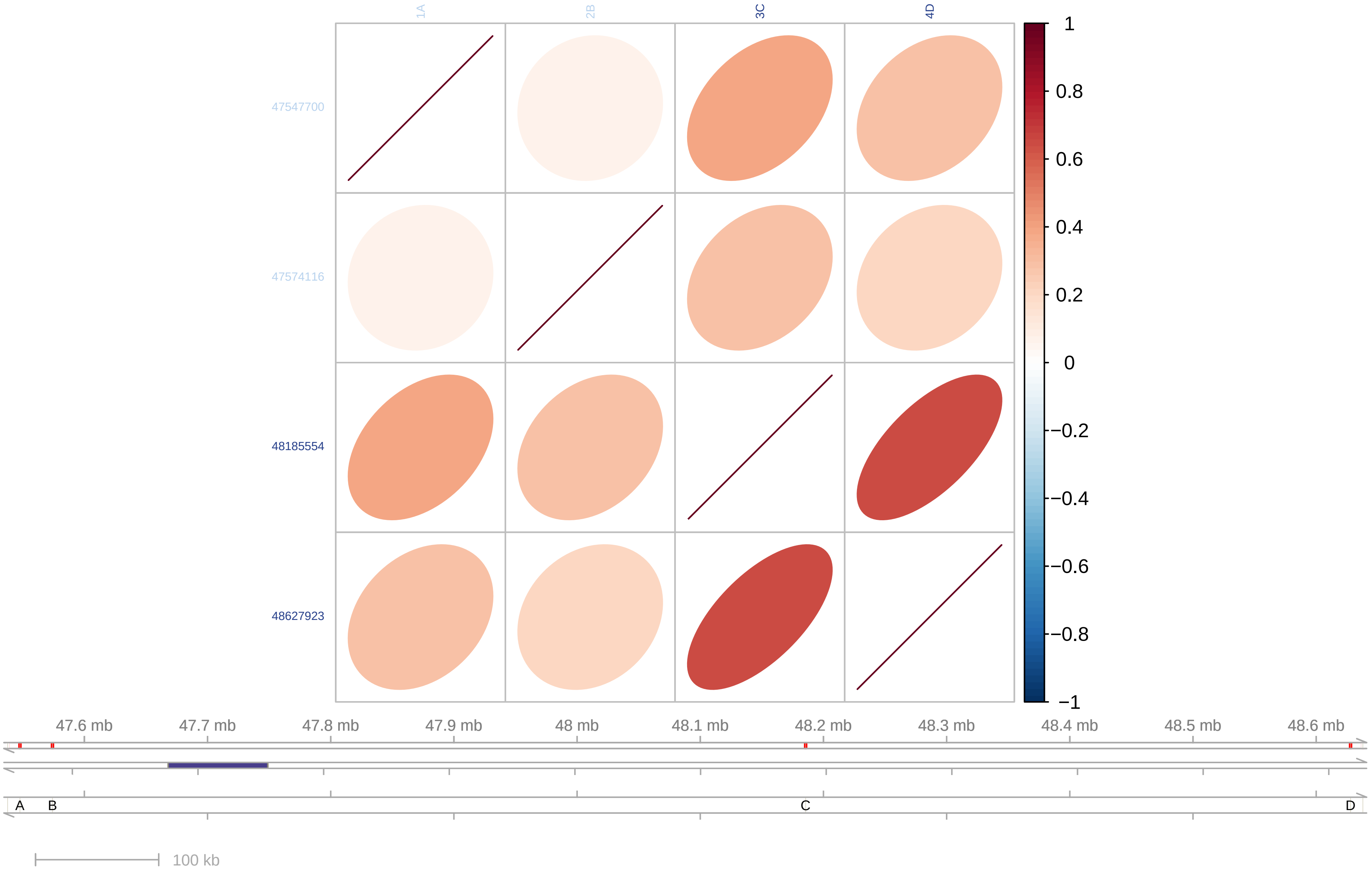

1A
2B
3C
4D
47547700
47574116
48185554
48627923
1
0.8
0.6
0.4
0.2
0
−0.2
−0.4
−0.6
−0.8
−1
47.6 mb
47.7 mb
47.8 mb
47.9 mb
48 mb
48.1 mb
48.2 mb
48.3 mb
48.4 mb
48.5 mb
48.6 mb
A
B
C
D
100 kb

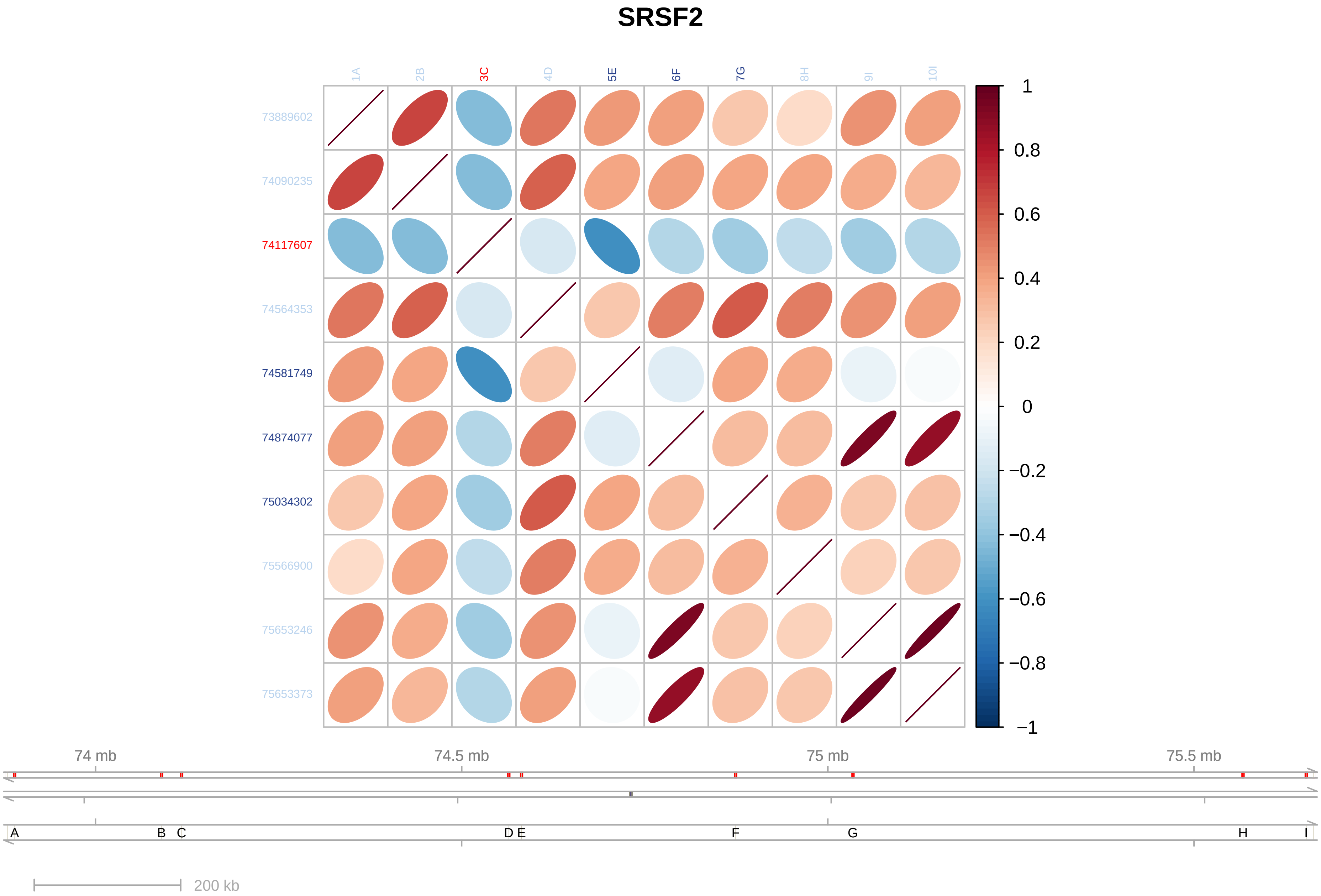
SRSF2
1A
2B
3C
4D
5E
6F
7G
8H
9I
10I
73889602
74090235
74117607
74564353
74581749
74874077
75034302
75566900
75653246
75653373
1
0.8
0.6
0.4
0.2
0
−0.2
−0.4
−0.6
−0.8
−1
74 mb
74.5 mb
75 mb
75.5 mb
A
B
C
D
E
F
G
H
I
200 kb

STK11

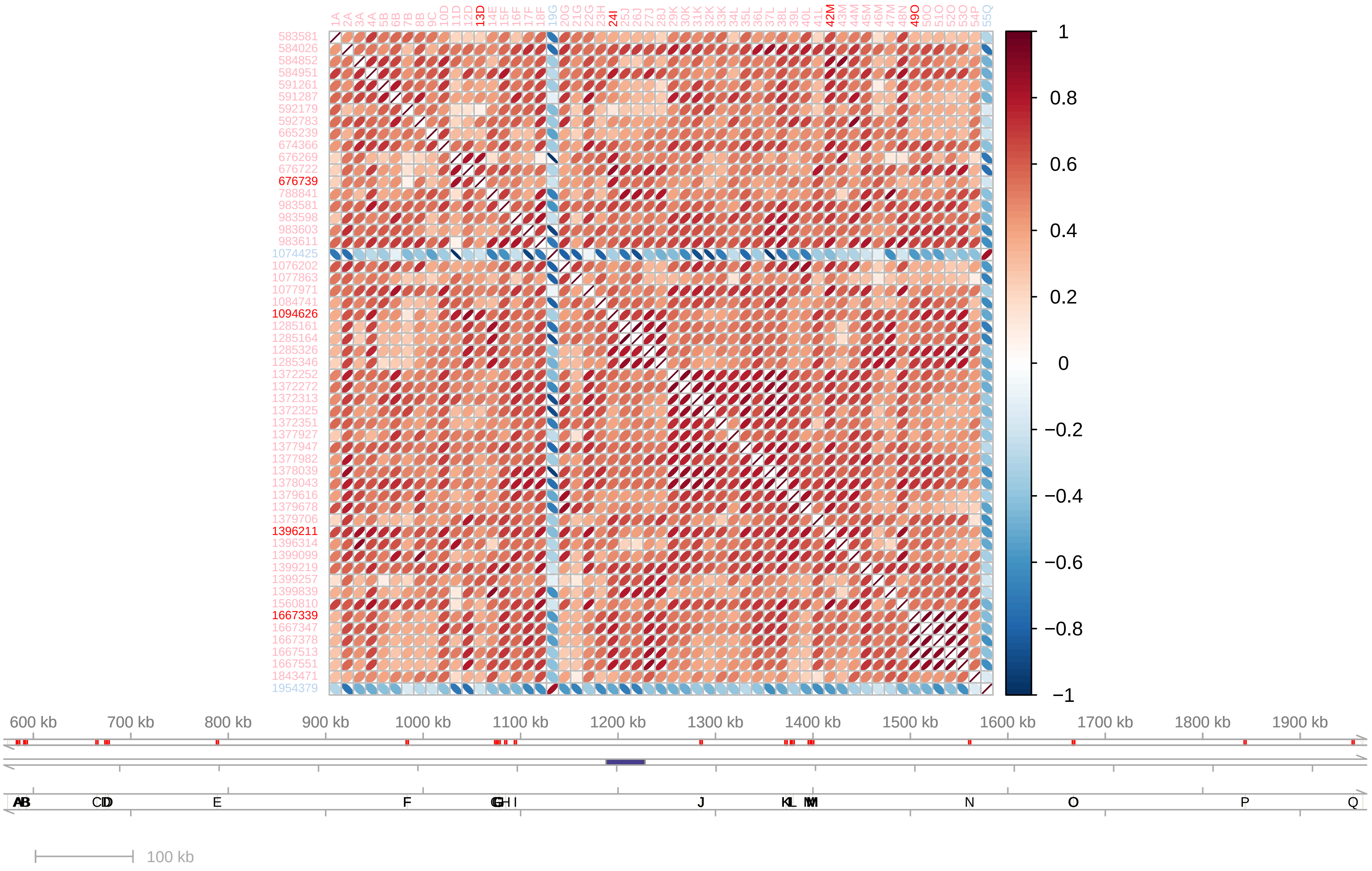

1A 2A 3A 4A 5B 6B 7B 8B 9C 10D 11D 12D 13D 14E 15F 16F 17F 18F 19G 20G 21G 22G 23H 24I 25J 26J 27J 28J 29K 30K 31K 32K 33K 34L 35L 36L 37L 38L 39L 40L 41L 42M 43M 44M 45M 46M 47M 48N 49O 50O 51O 52O 53O 54P 55Q
583581
584026
584852
584951
591261
591287
592179
592783
665239
674366
676269
676722
676739
788841
983581
983598
983603
983611
1074425
1076202
1077863
1077971
1084741
1094626
1285161
1285164
1285326
1285346
1372252
1372272
1372313
1372325
1372351
1377927
1377947
1377982
1378039
1378043
1379616
1379678
1379706
1396211
1396314
1399099
1399219
1399257
1399839
1560810
1667339
1667347
1667378
1667513
1667551
1843471
1954379
1
0.8
0.6
0.4
0.2
0
−0.2
−0.4
−0.6
−0.8
−1
600 kb 700 kb 800 kb 900 kb 1000 kb 1100 kb 1200 kb 1300 kb 1400 kb 1500 kb 1600 kb 1700 kb 1800 kb 1900 kb
AB CD E F GHI J KLM N O P Q
100 kb

TNFAIP3

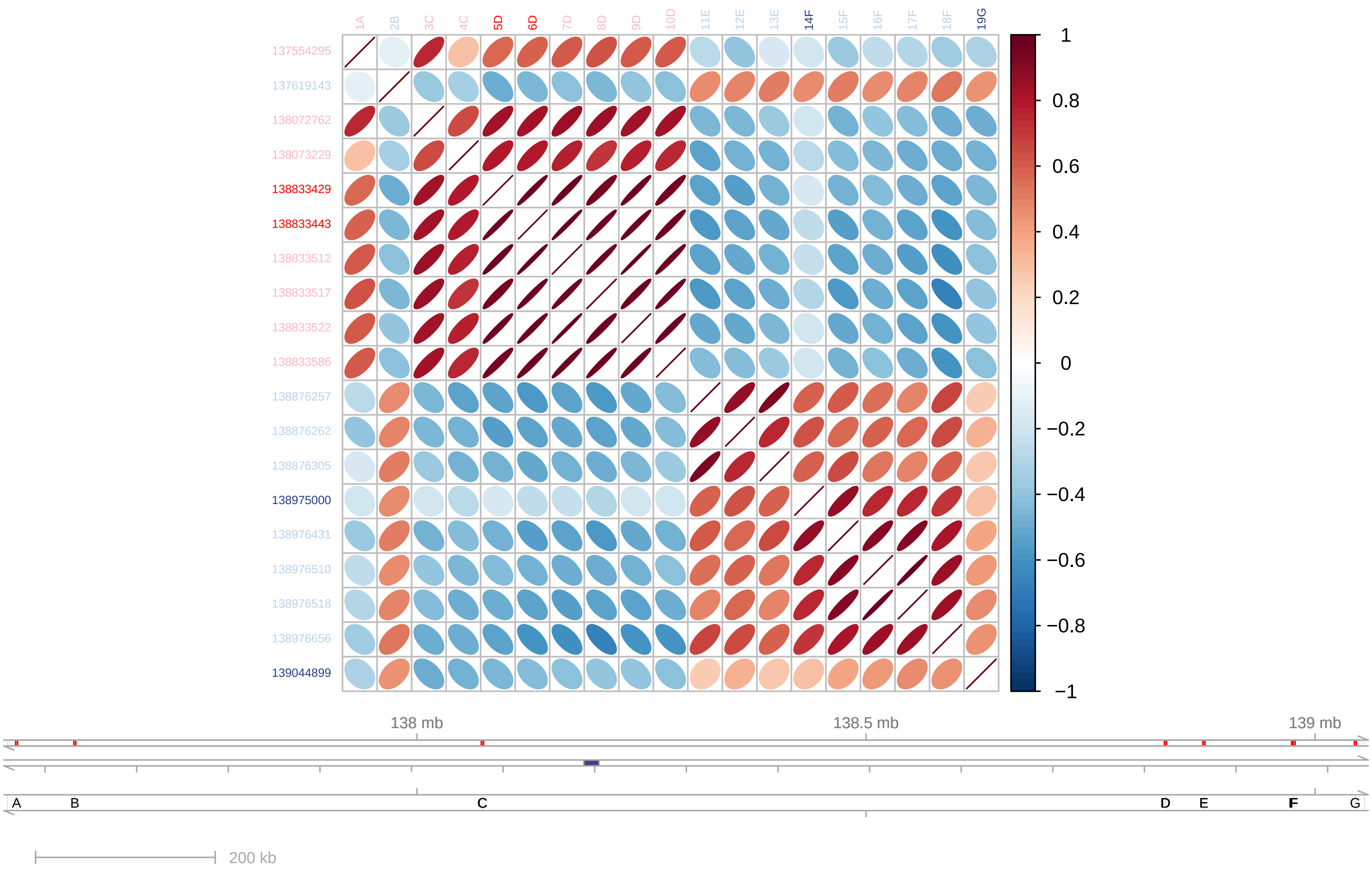

1A
2B
3C
4C
5D
6D
7D
8D
9D
10D
11E
12E
13E
14F
15F
16F
17F
18F
19G
137554295
137619143
138072762
138073229
138833429
138833443
138833512
138833517
138833522
138833586
138876257
138876262
138876305
138975000
138976431
138976510
138976518
138976656
139044899
1
0.8
0.6
0.4
0.2
0
−0.2
−0.4
−0.6
−0.8
−1
138 mb
138.5 mb
139 mb
A
B
C
D
E
F
G
200 kb

TRAF7

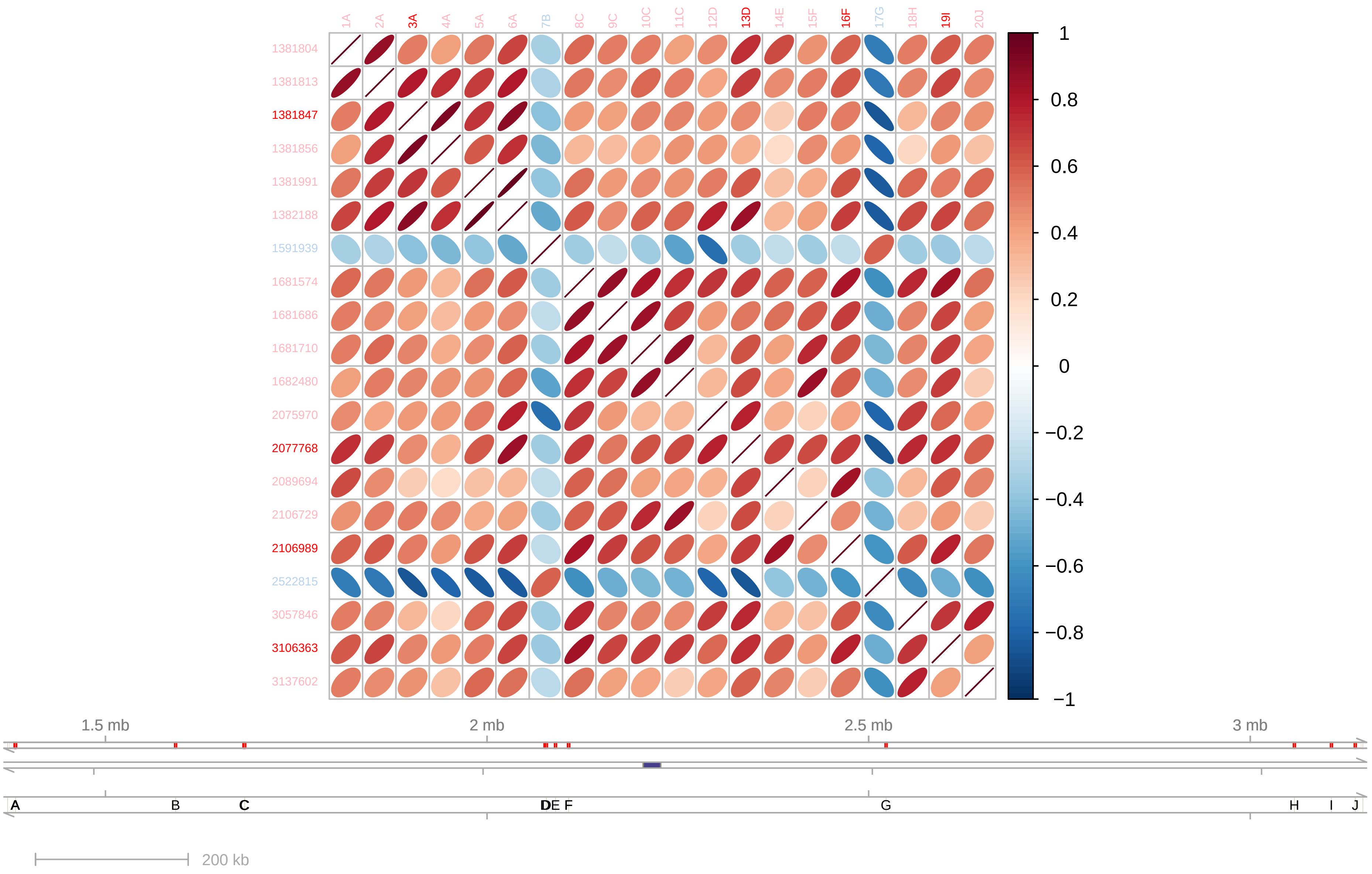
1A 2A 3A 4A 5A 6A 7B 8C 9C 10C 11C 12D 13D 14E 15F 16F 17G 18H 19I 20J
1381804
1381813
1381847
1381856
1381991
1382188
1591939
1681574
1681686
1681710
1682480
2075970
2077768
2089694
2106729
2106989
2522815
3057846
3106363
3137602
1
0.8
0.6
0.4
0.2
0
−0.2
−0.4
−0.6
−0.8
−1
1.5 mb
2 mb
2.5 mb
3 mb
A B C DE F G H I J
200 kb

TSC1

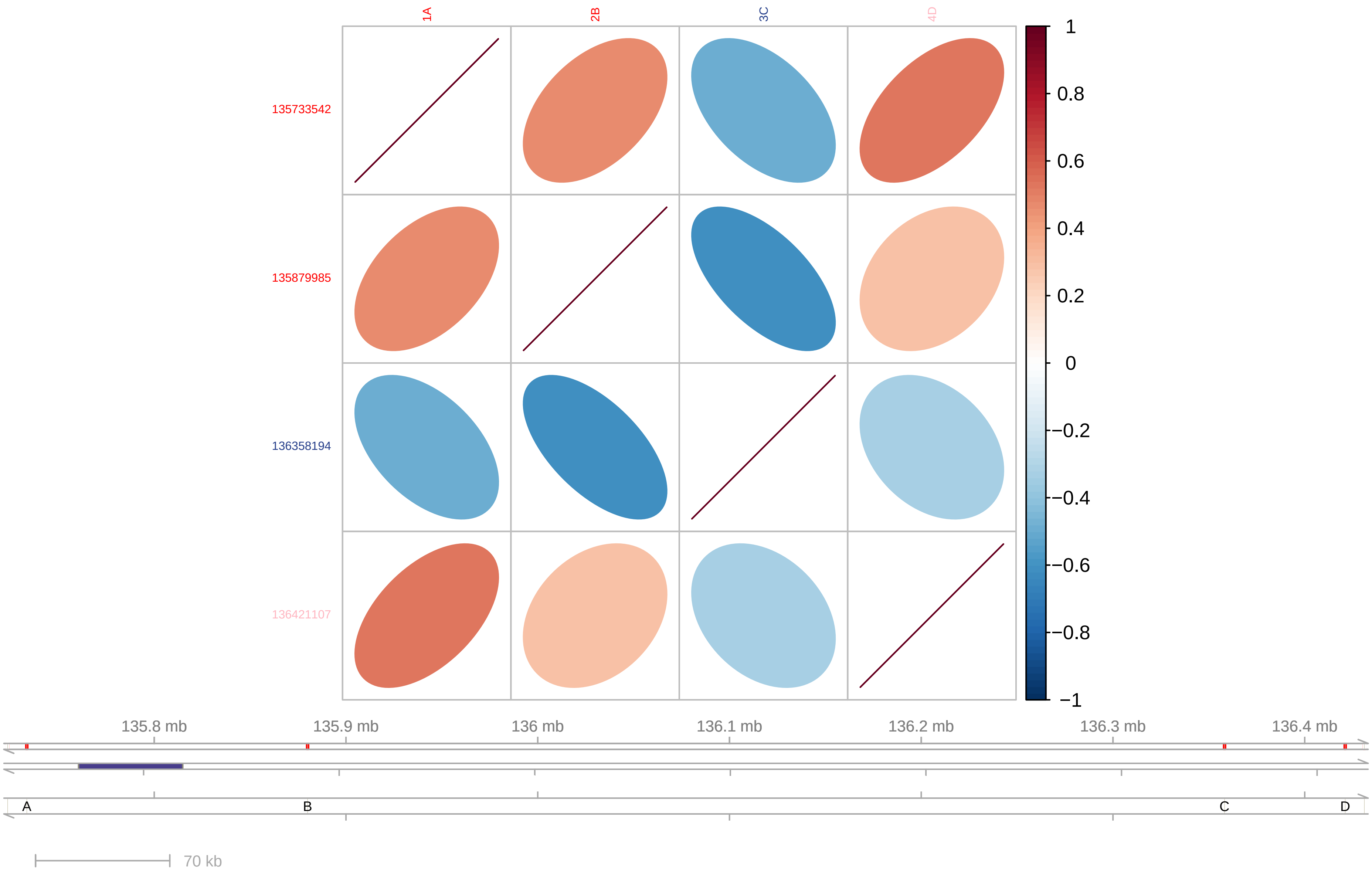

1A
2B
3C
4D
135733542
135879985
136358194
136421107
1
0.8
0.6
0.4
0.2
0
−0.2
−0.4
−0.6
−0.8
−1
135.8 mb
135.9 mb
136 mb
136.1 mb
136.2 mb
136.3 mb
136.4 mb
A
B
C
D
70 kb

U2AF1

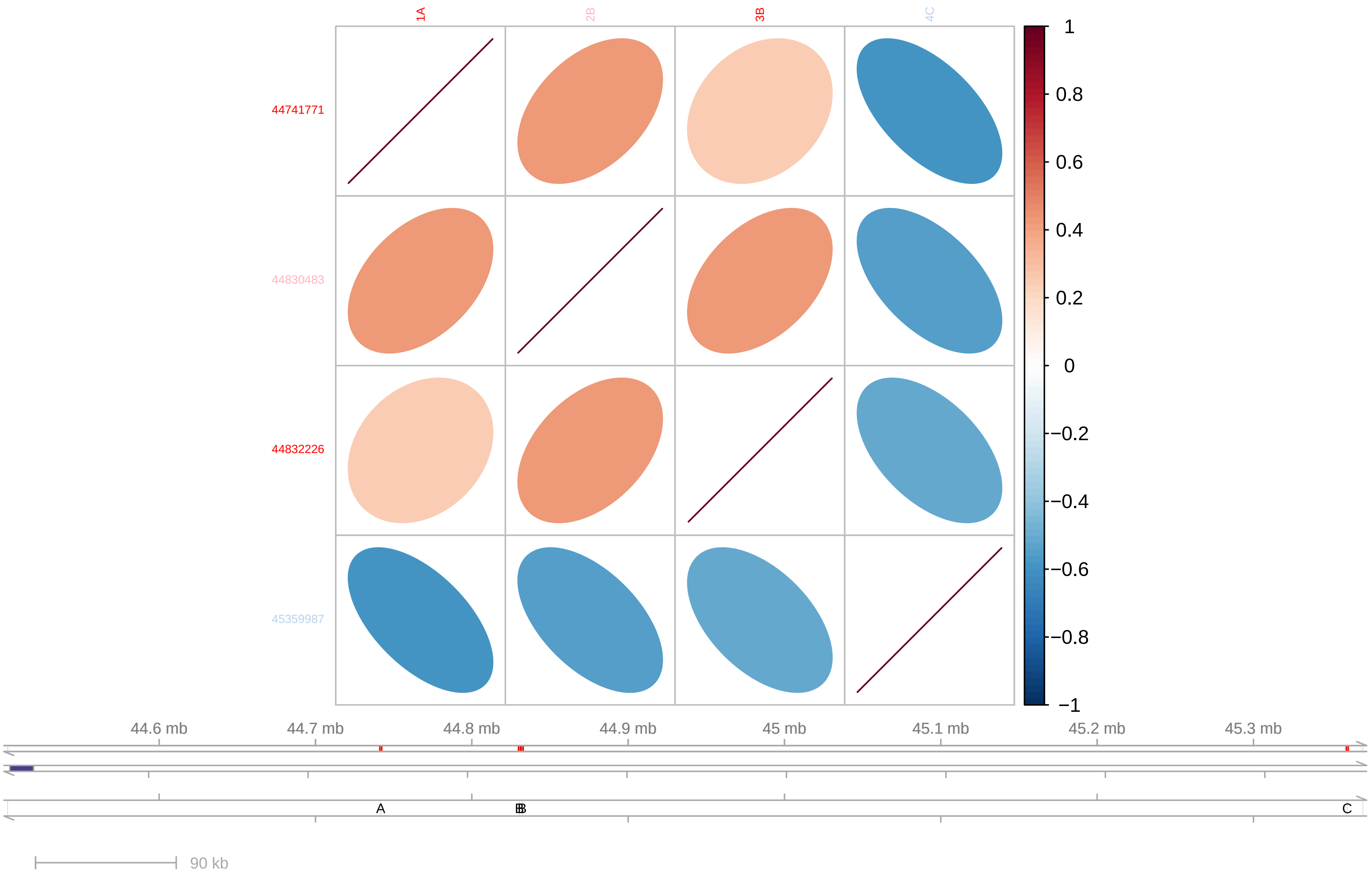
1A
2B
3B
4C
44741771
44830483
44832226
45359987
1
0.8
0.6
0.4
0.2
0
−0.2
−0.4
−0.6
−0.8
−1
44.6 mb
44.7 mb
44.8 mb
44.9 mb
45 mb
45.1 mb
45.2 mb
45.3 mb
A
B
C
90 kb

VHL

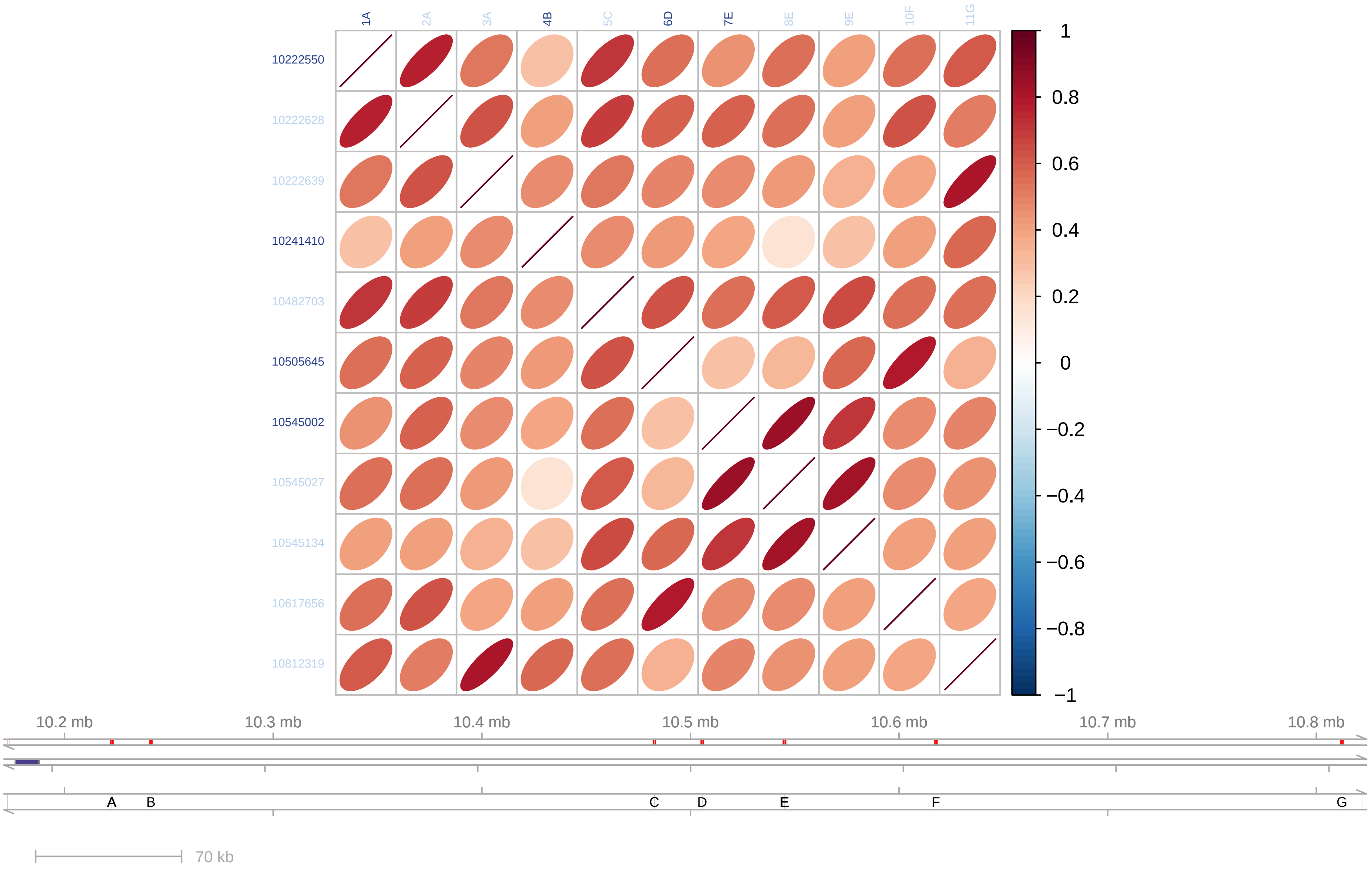

1A
2A
3A
4B
5C
6D
7E
8E
9E
10F
11G
10222550
10222628
10222639
10241410
10482703
10505645
10545002
10545027
10545134
10617656
10812319
1
0.8
0.6
0.4
0.2
0
−0.2
−0.4
−0.6
−0.8
−1
10.2 mb
10.3 mb
10.4 mb
10.5 mb
10.6 mb
10.7 mb
10.8 mb
A
B
C
D
E
F
G
70 kb

VIPR2

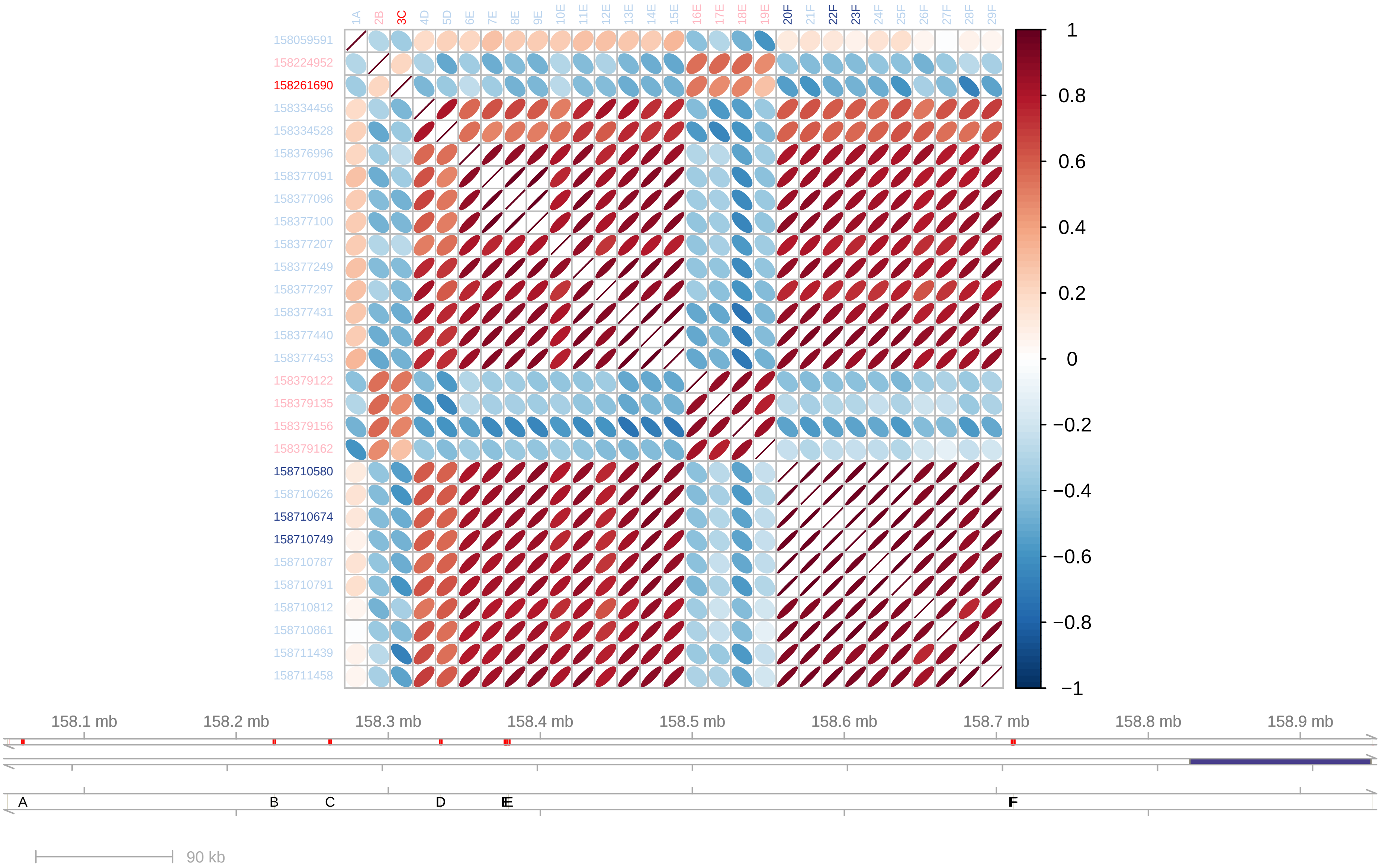

1A
2B
3C
4D
5D
6E
7E
8E
9E
10E
11E
12E
13E
14E
15E
16E
17E
18E
19E
20F
21F
22F
23F
24F
25F
26F
27F
28F
29F
158059591
158224952
158261690
158334456
158334528
158376996
158377091
158377096
158377100
158377207
158377249
158377297
158377431
158377440
158377453
158379122
158379135
158379156
158379162
158710580
158710626
158710674
158710749
158710787
158710791
158710812
158710861
158711439
158711458
1
0.8
0.6
0.4
0.2
0
−0.2
−0.4
−0.6
−0.8
−1
158.1 mb
158.2 mb
158.3 mb
158.4 mb
158.5 mb
158.6 mb
158.7 mb
158.8 mb
158.9 mb
A
B
C
D
E
F
90 kb

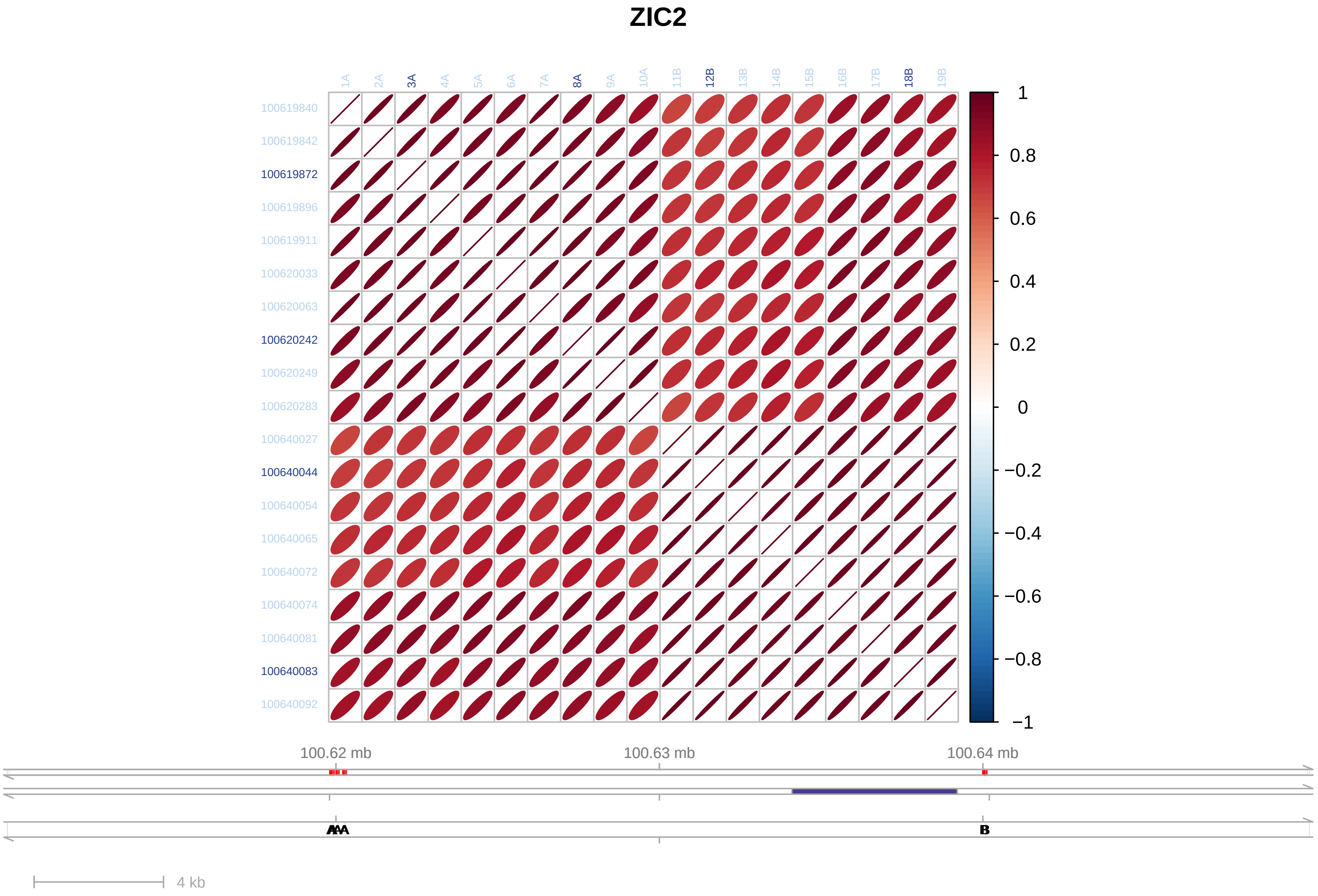

ZIC2
1A
2A
3A
4A
5A
6A
7A
8A
9A
10A
11B
12B
13B
14B
15B
16B
17B
18B
19B
100619840
100619842
100619872
100619896
100619911
100620033
100620063
100620242
100620249
100620283
100640027
100640044
100640054
100640065
100640072
100640074
100640081
100640083
100640092
1
0.8
0.6
0.4
0.2
0
−0.2
−0.4
−0.6
−0.8
−1
100.62 mb
100.63 mb
100.64 mb
AAA
B
4 kb
