# Supplementary figures and images for: Methylation-directed regulatory networks determine enhancing and silencing of mutation disease driver genes and explain inter-patient expression variation
Source: Genome Biol. 2023 Nov 28;24:264. doi: 10.1186/s13059-023-03094-6 (PMC10683314; doi:10.1186/s13059-023-03094-6)

# DLL3

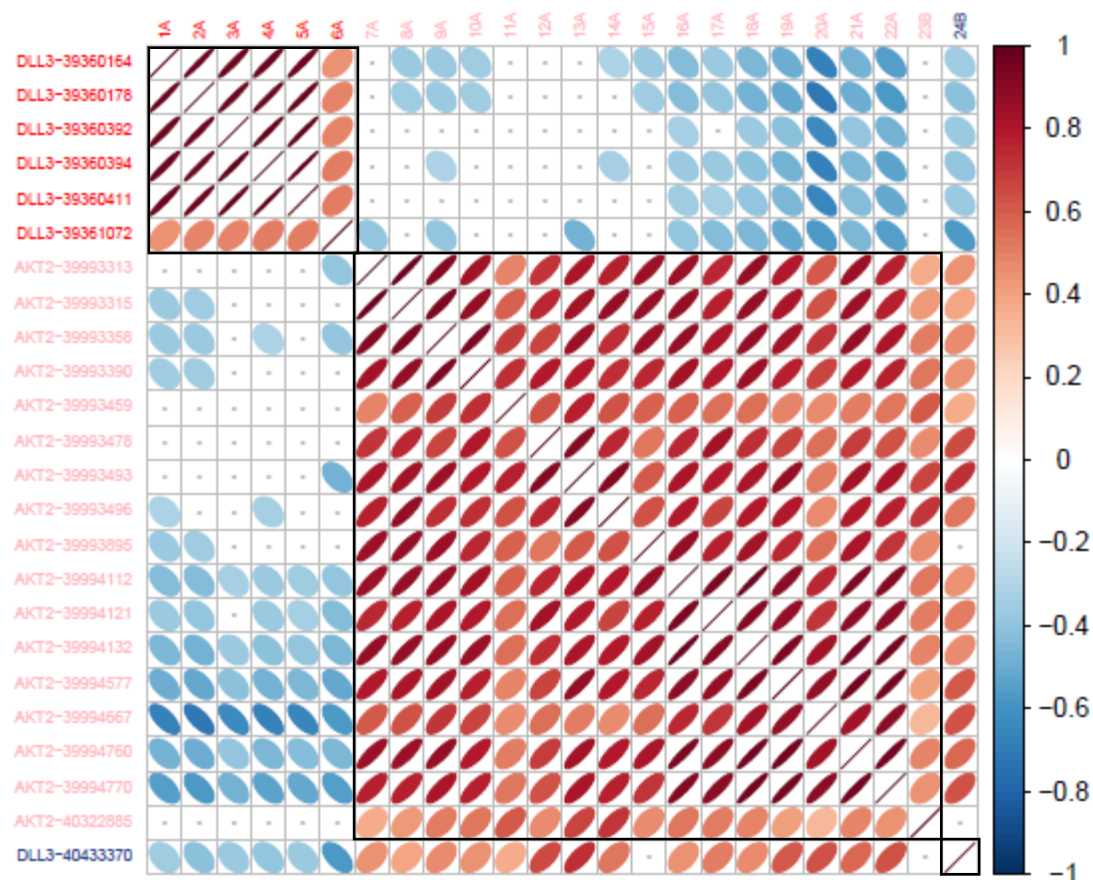

# AKT2

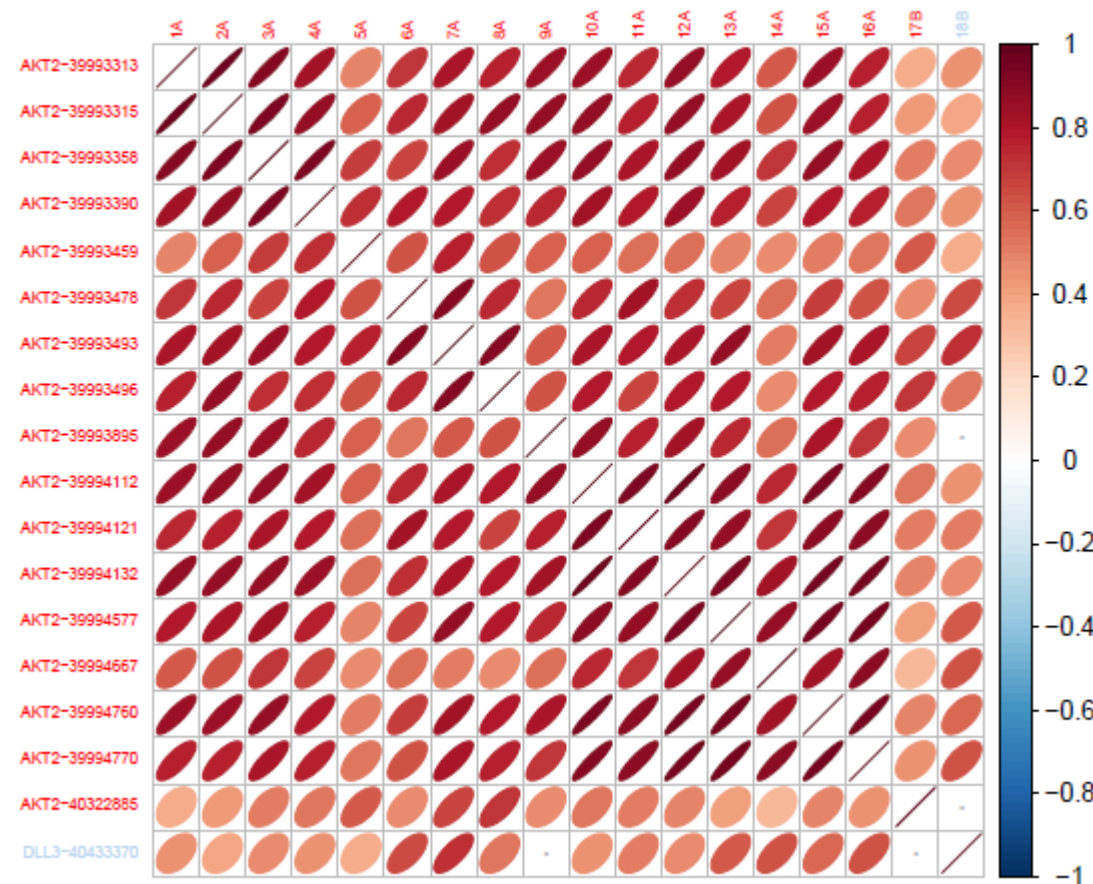

## GDF15

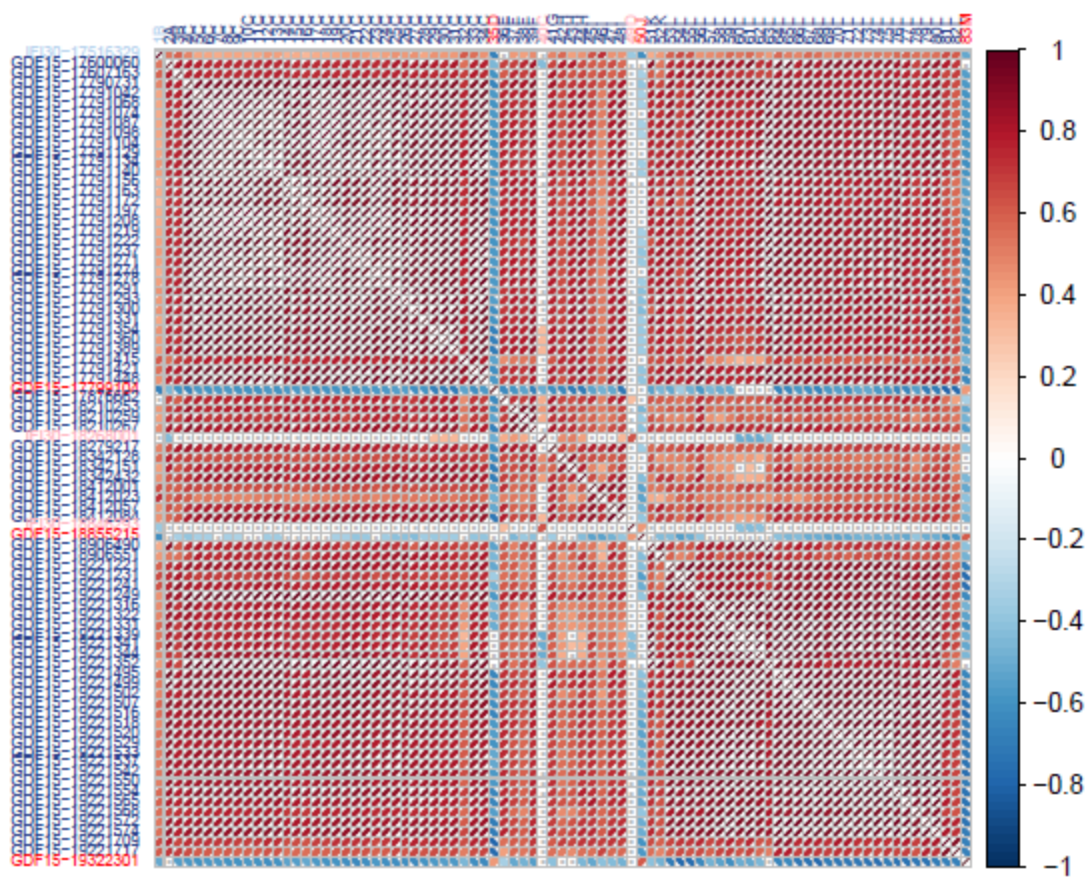

## IFI30

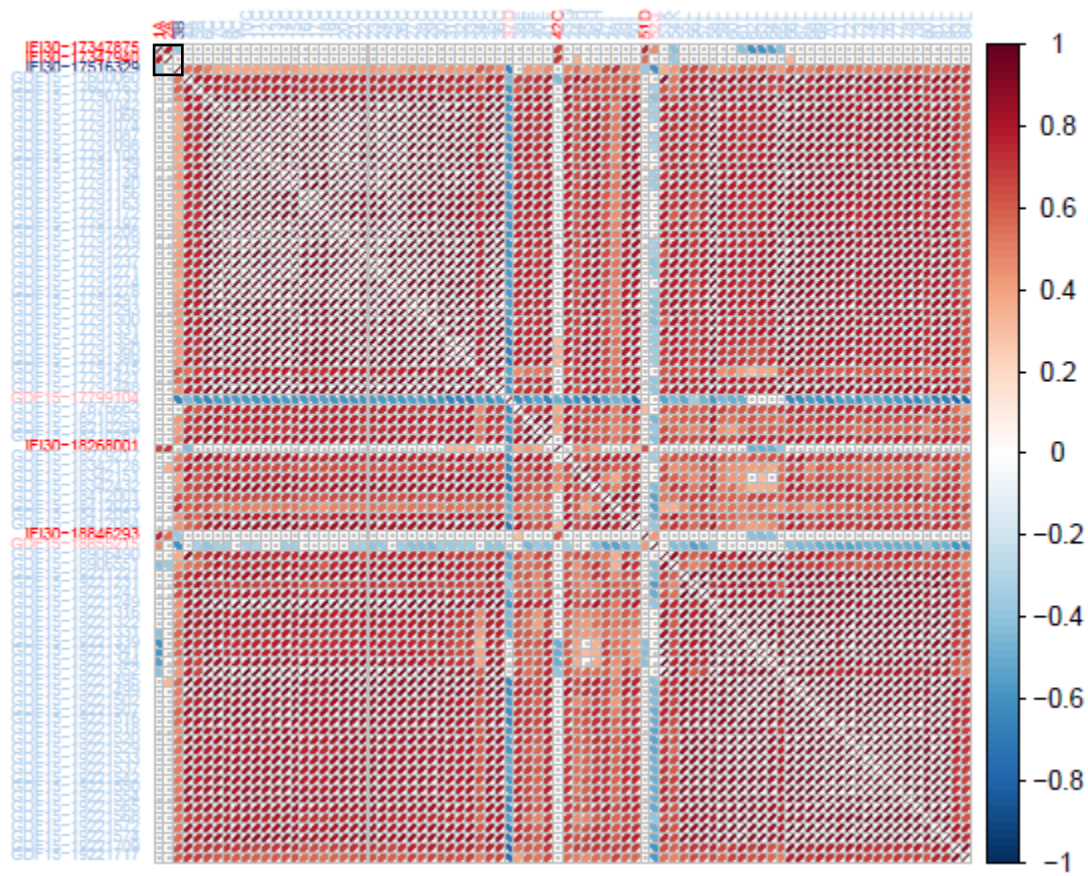

# MYD88

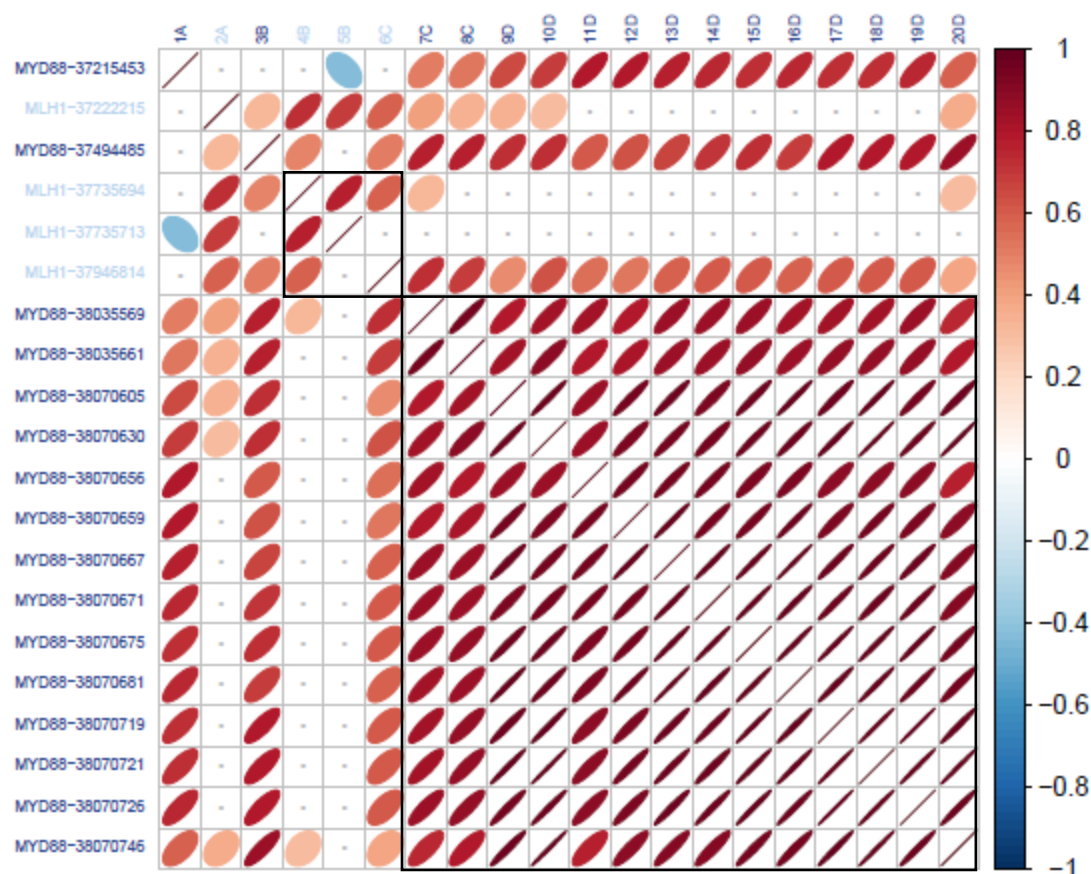

# MLH1

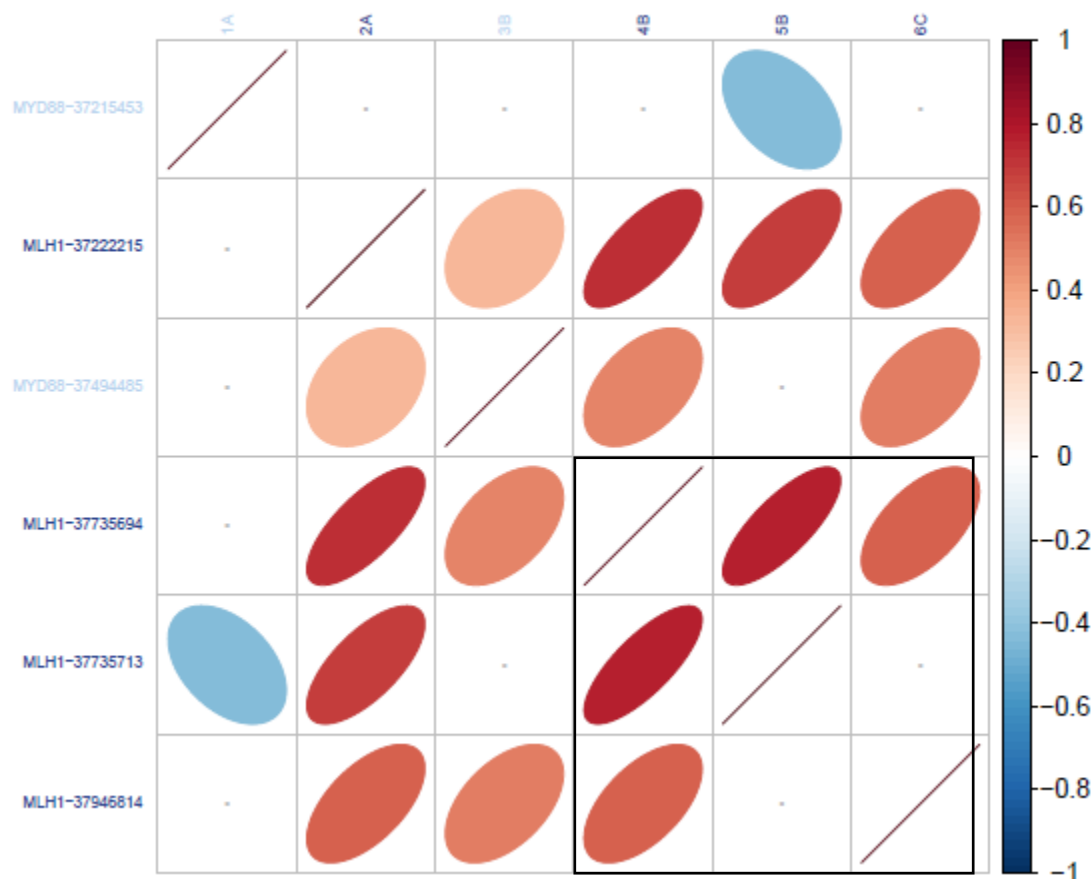

Supplement: Supplementary file 4 — Additional file 4. Methylation-methylation correlation across overlapping gene domains. Matrixes presetting the correlation map of a gene which share its domain with another gene (the other gene’s map is presented below). Each square in the matrixes show the methylation versus methylation correlation (R) between two of the associated sites. Gene symbols and genomic locations of the associated sites are given to the left. White squares denote no correlation (R2 <0.1). [file 13059_2023_3094_MOESM4_ESM.pdf]
