# Supplementary material for: Methylation-directed regulatory networks determine enhancing and silencing of mutation disease driver genes and explain inter-patient expression variation
Source: Genome Biol. 2023 Nov 28;24:264. doi: 10.1186/s13059-023-03094-6 (PMC10683314; doi:10.1186/s13059-023-03094-6)
Supplement: Supplementary file 5 — Additional file 5. Evaluation of methylation-based prediction by 23-leaving-1 tests. Scatter plots showing the predicted versus observed gene expression levels in one of the 24 independent tests. Cancer driver genes (red dots), GBM subtype signature (green), non-driver GBM candidate (blue) and non-driver variable (black) genes are shown. Gene expression values are in log2 RSEM. The number of test sets, name and subtype of the leaving-out tumor, and the obtained correlation value are indicated above each plot. [file 13059_2023_3094_MOESM5_ESM.pdf]

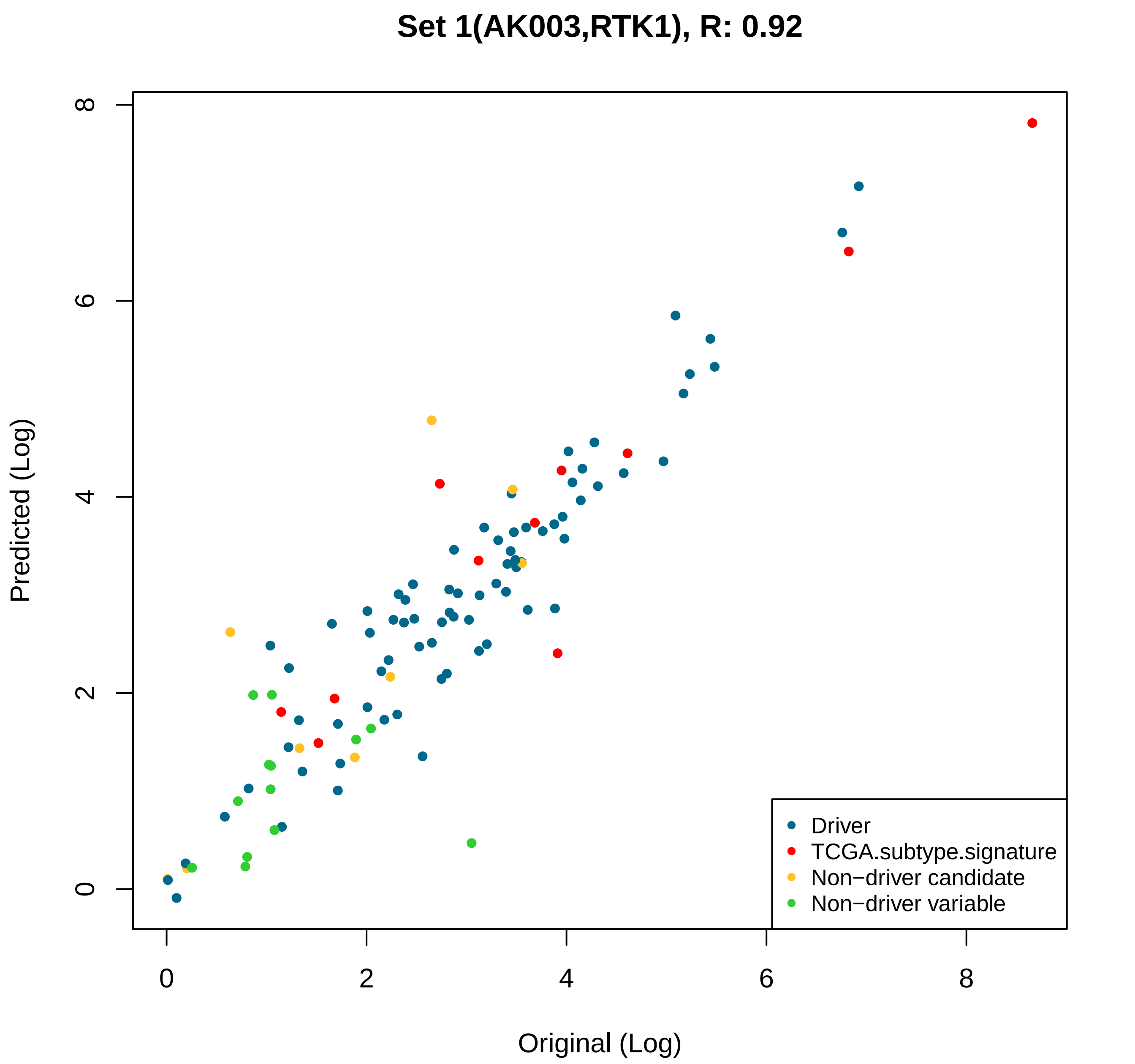

Set 1(AK003,RTK1), R: 0.92
Predicted (Log)
Original (Log)
0
2
4
6
8
Driver
TCGA.subtype.signature
Non−driver candidate
Non−driver variable

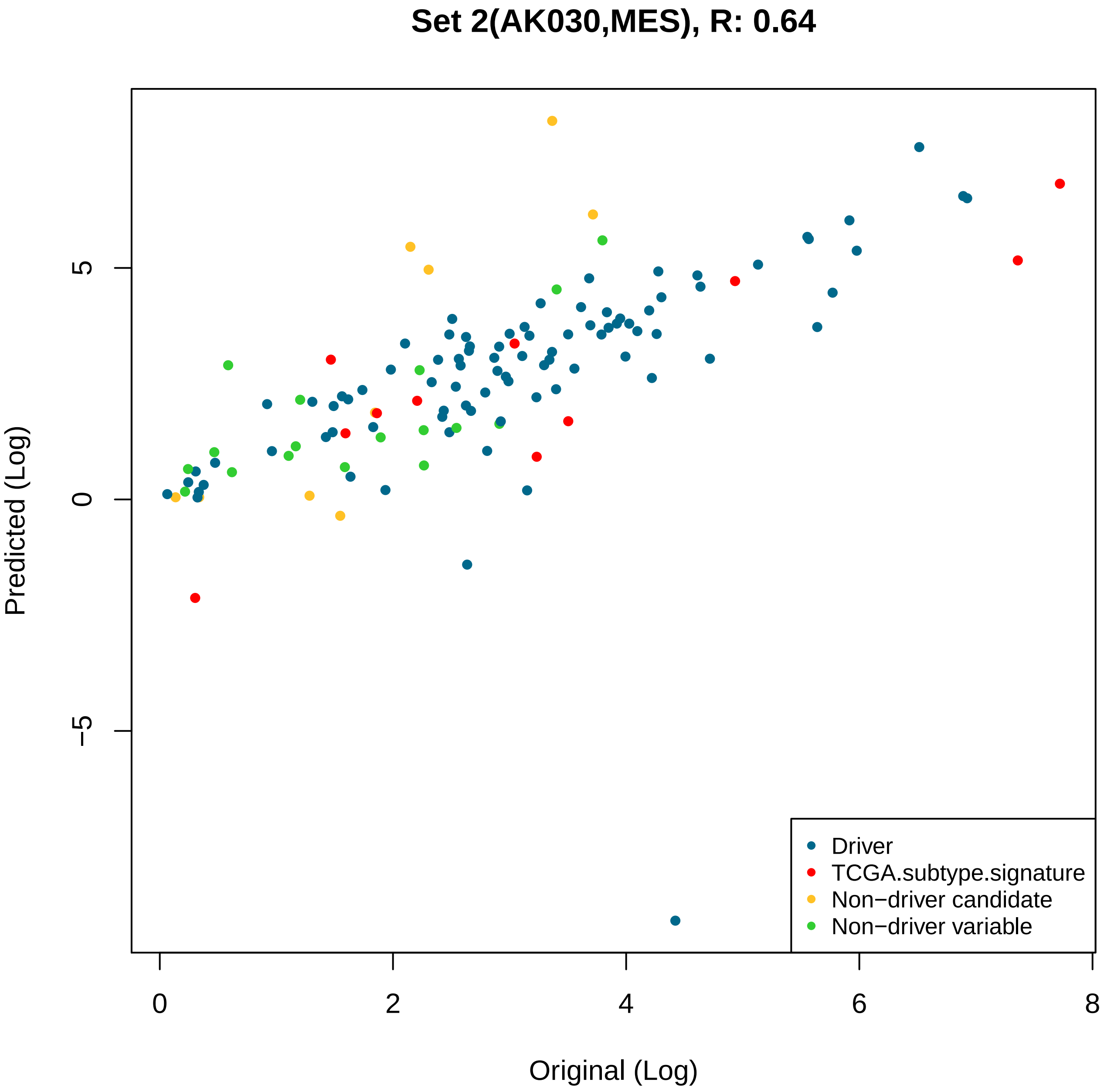

Set 2(AK030,MES), R: 0.64
Predicted (Log)
Original (Log)
−5
0
5
0
2
4
6
8
Driver
TCGA.subtype.signature
Non−driver candidate
Non−driver variable

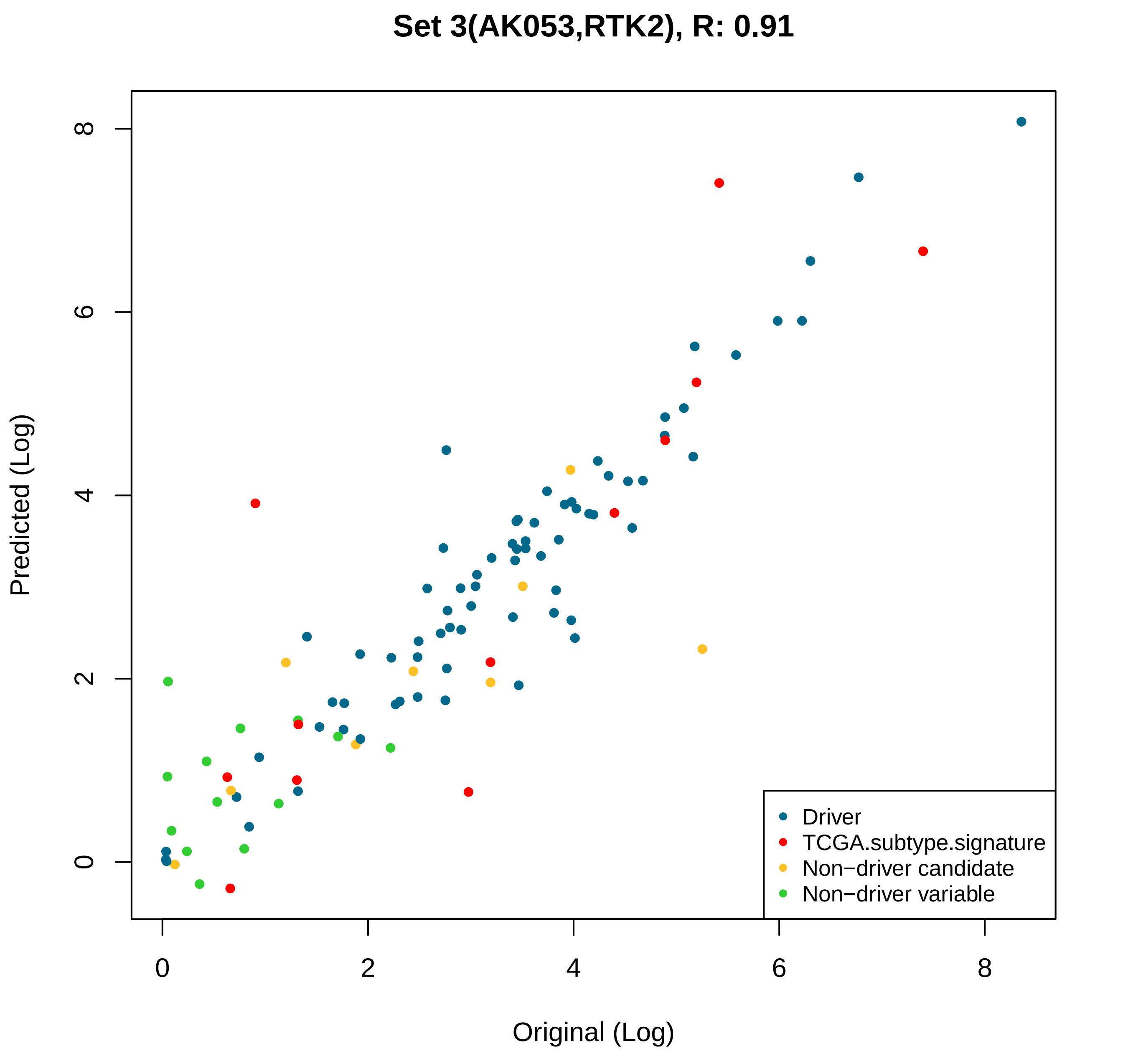
Set 3(AK053,RTK2), R: 0.91
Predicted (Log)
Original (Log)
0
2
4
6
8
Driver
TCGA.subtype.signature
Non−driver candidate
Non−driver variable

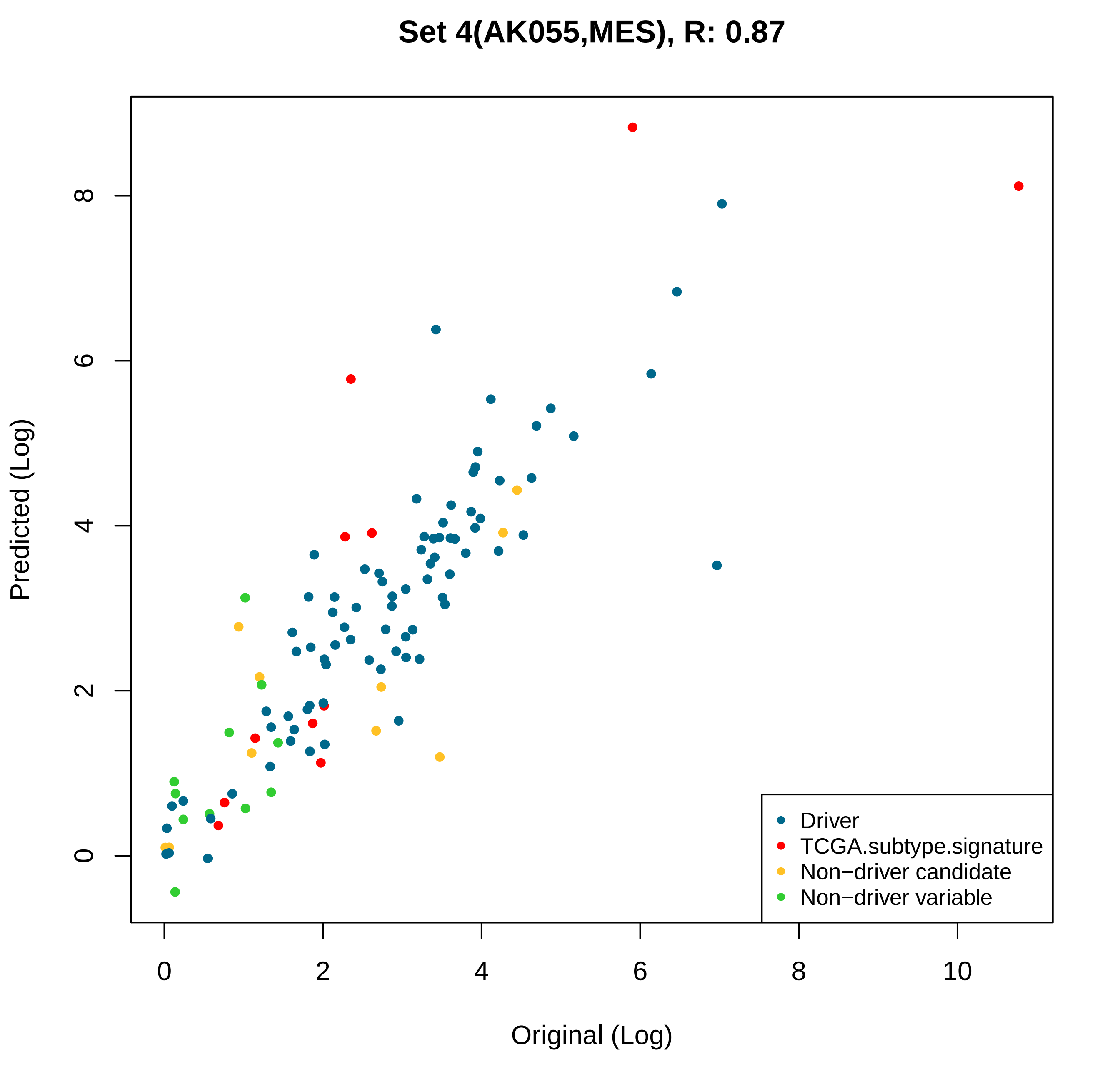
Set 4(AK055,MES), R: 0.87
Predicted (Log)
Original (Log)
0
2
4
6
8
10
Driver
TCGA.subtype.signature
Non−driver candidate
Non−driver variable

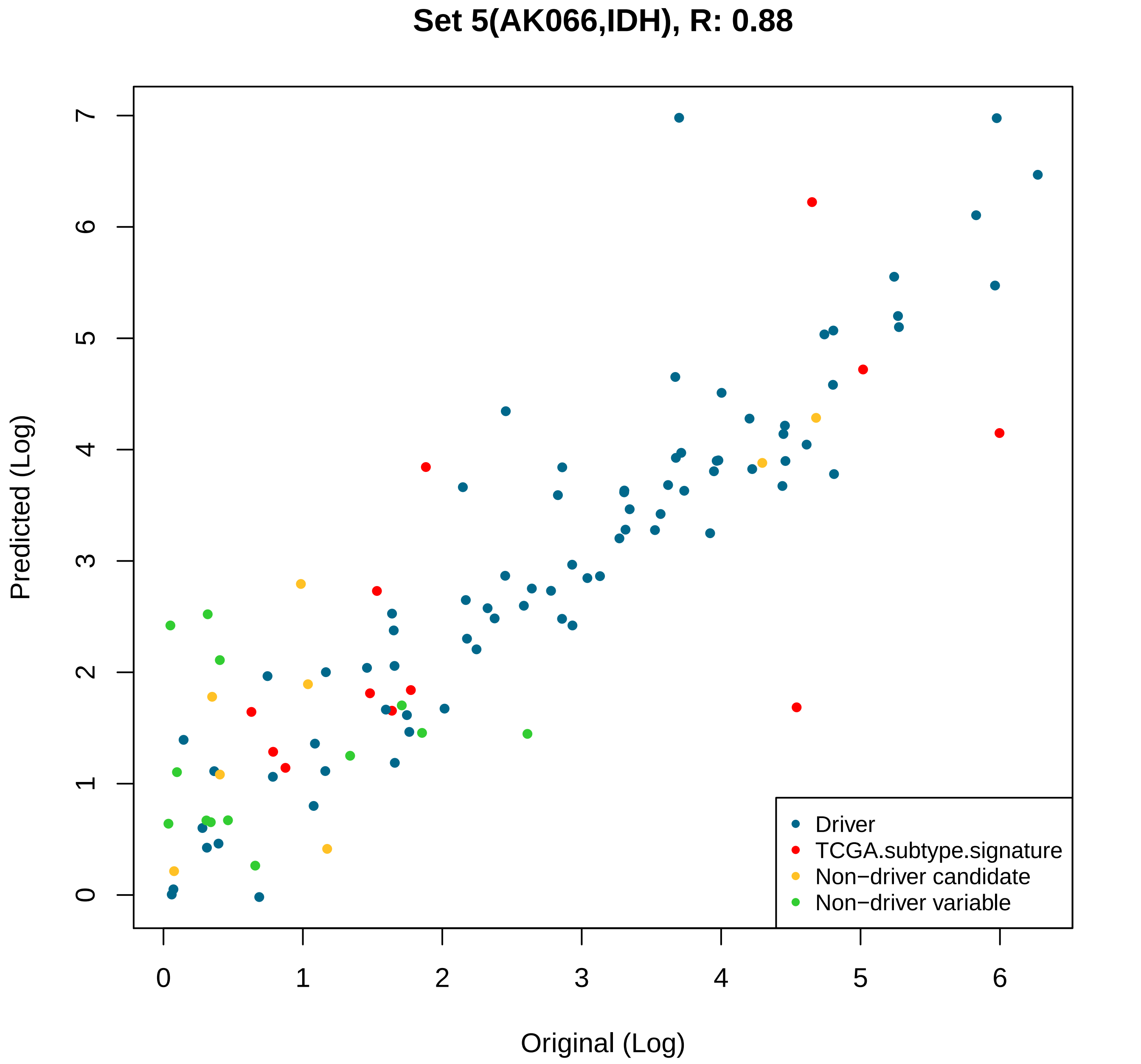
Set 5(AK066,IDH), R: 0.88
Predicted (Log)
Original (Log)
0
1
2
3
4
5
6
7
Driver
TCGA.subtype.signature
Non−driver candidate
Non−driver variable

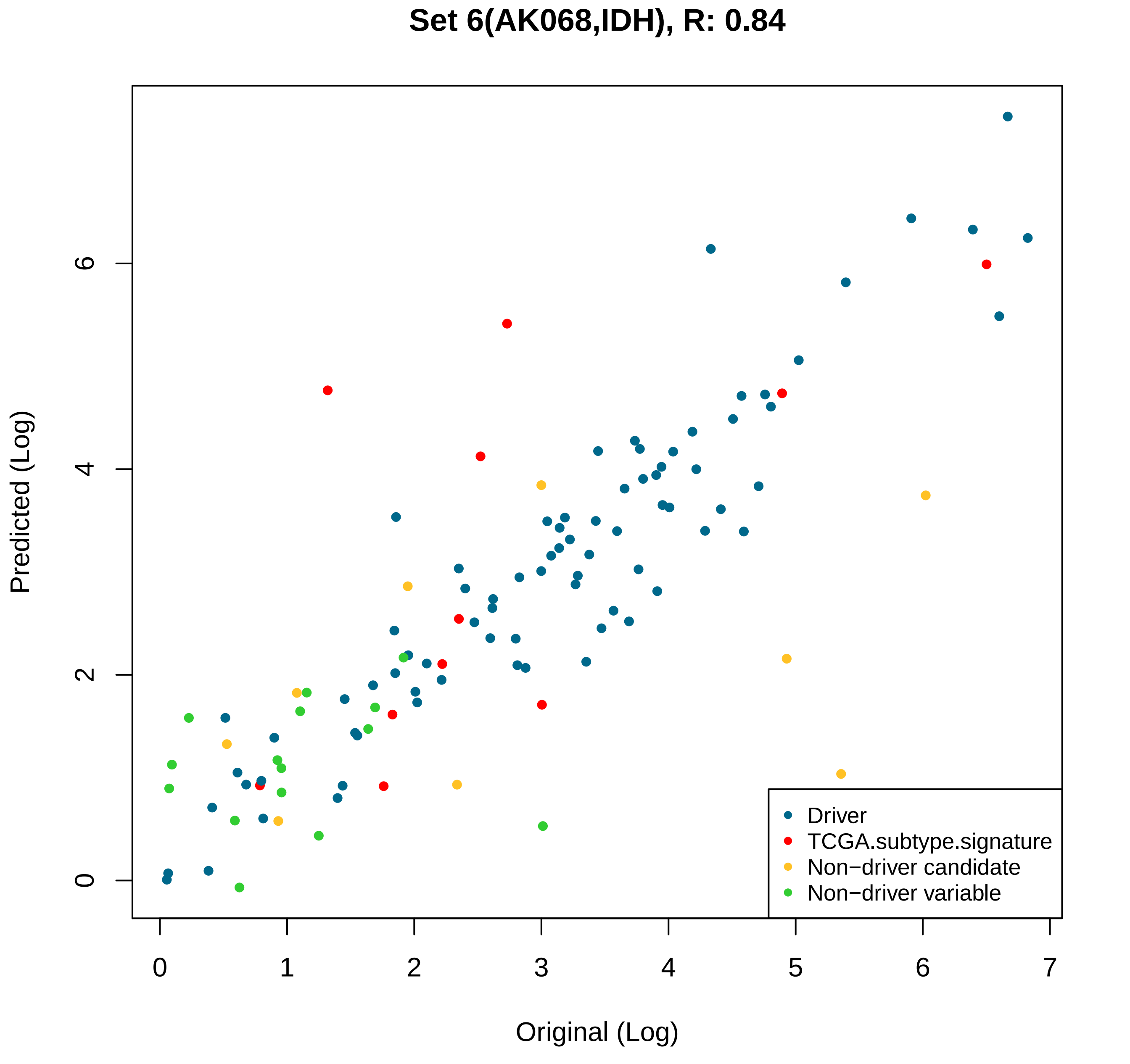

Set 6(AK068,IDH), R: 0.84
Predicted (Log)
Original (Log)
0
2
4
6
0
1
2
3
4
5
6
7
Driver
TCGA.subtype.signature
Non−driver candidate
Non−driver variable

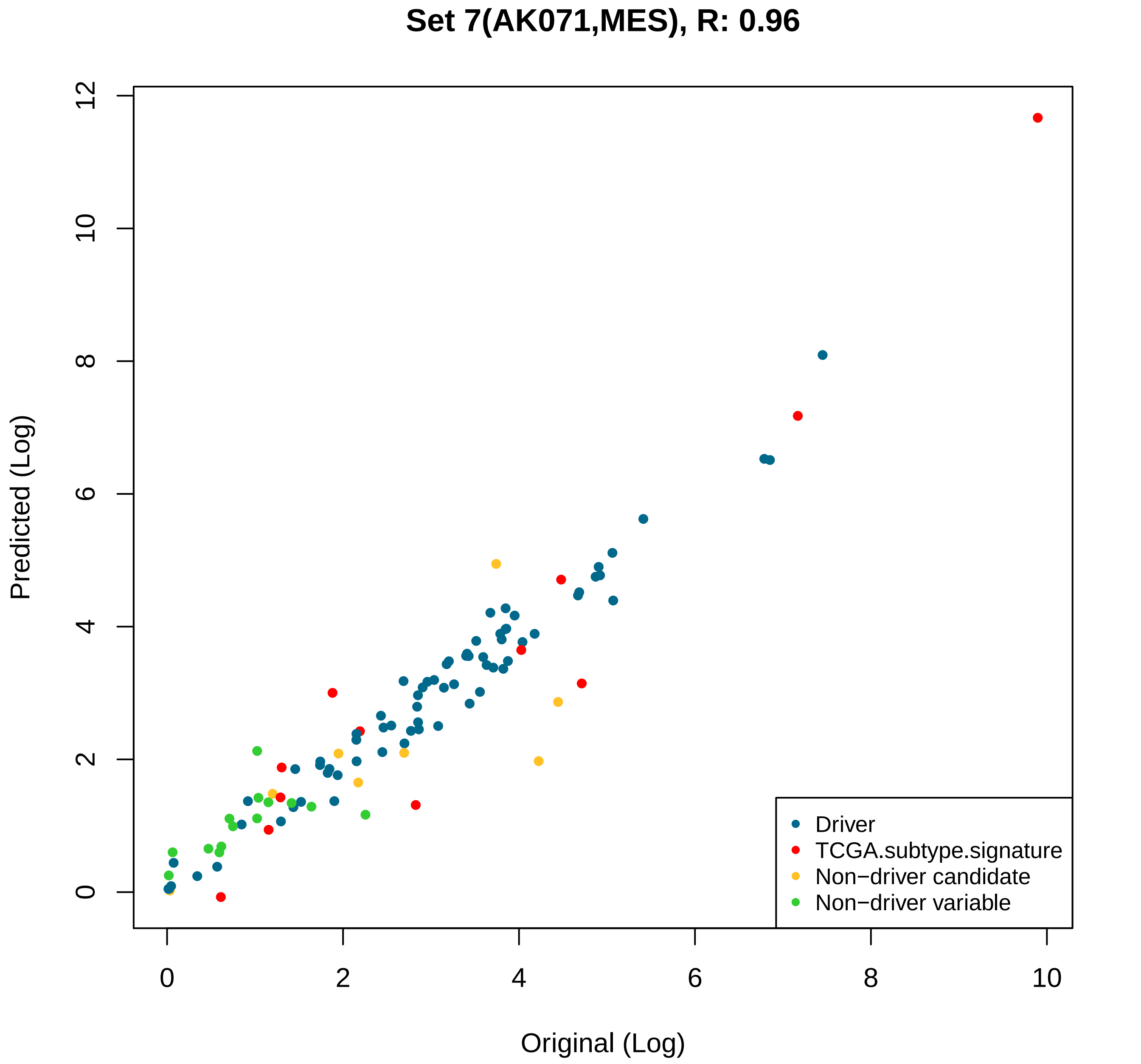

Set 7(AK071,MES), R: 0.96
Predicted (Log)
Original (Log)
0
2
4
6
8
10
12
Driver
TCGA.subtype.signature
Non−driver candidate
Non−driver variable

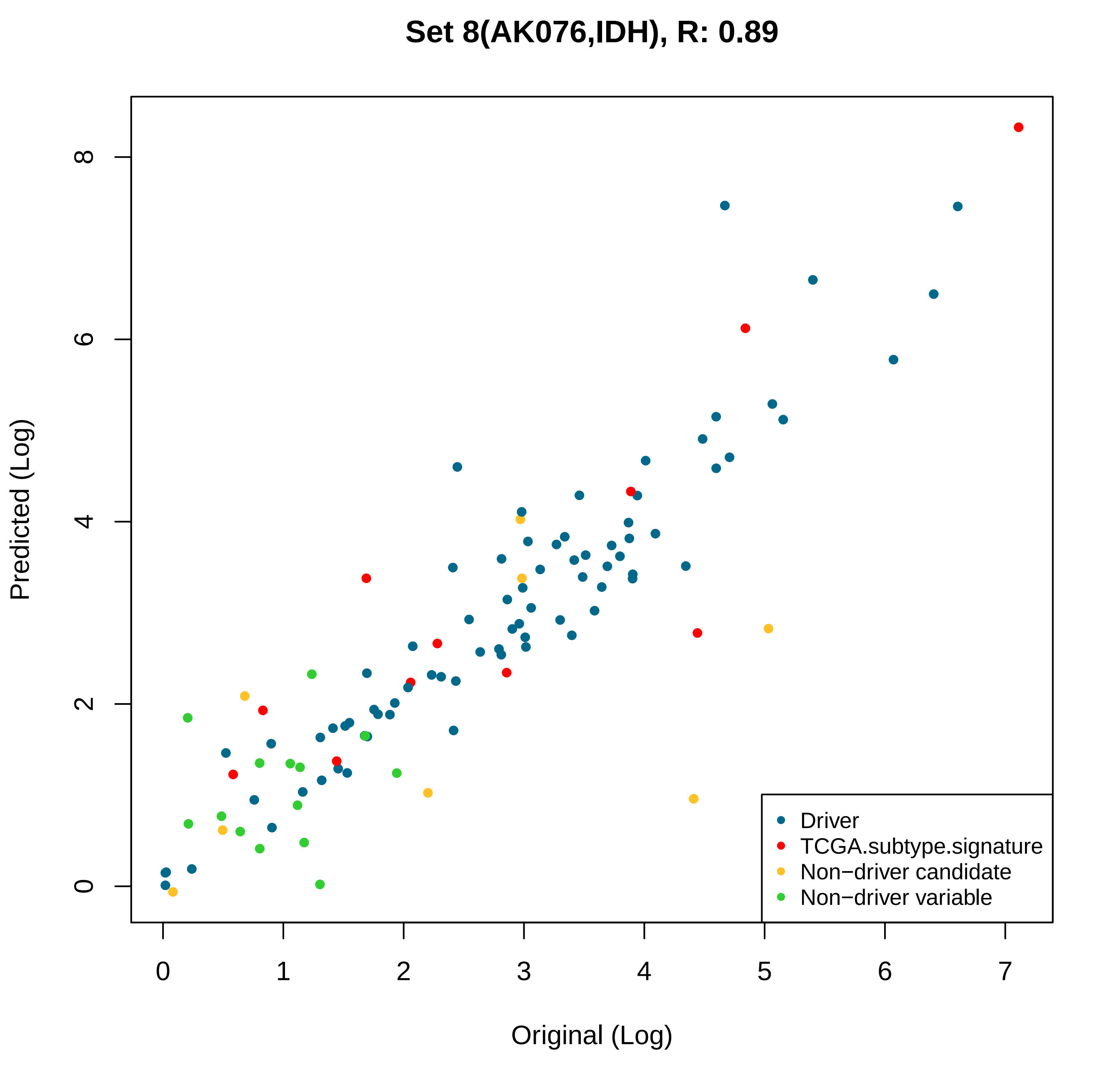
Set 8(AK076,IDH), R: 0.89
Predicted (Log)
Original (Log)
0
2
4
6
8
0
1
2
3
4
5
6
7
Driver
TCGA.subtype.signature
Non−driver candidate
Non−driver variable

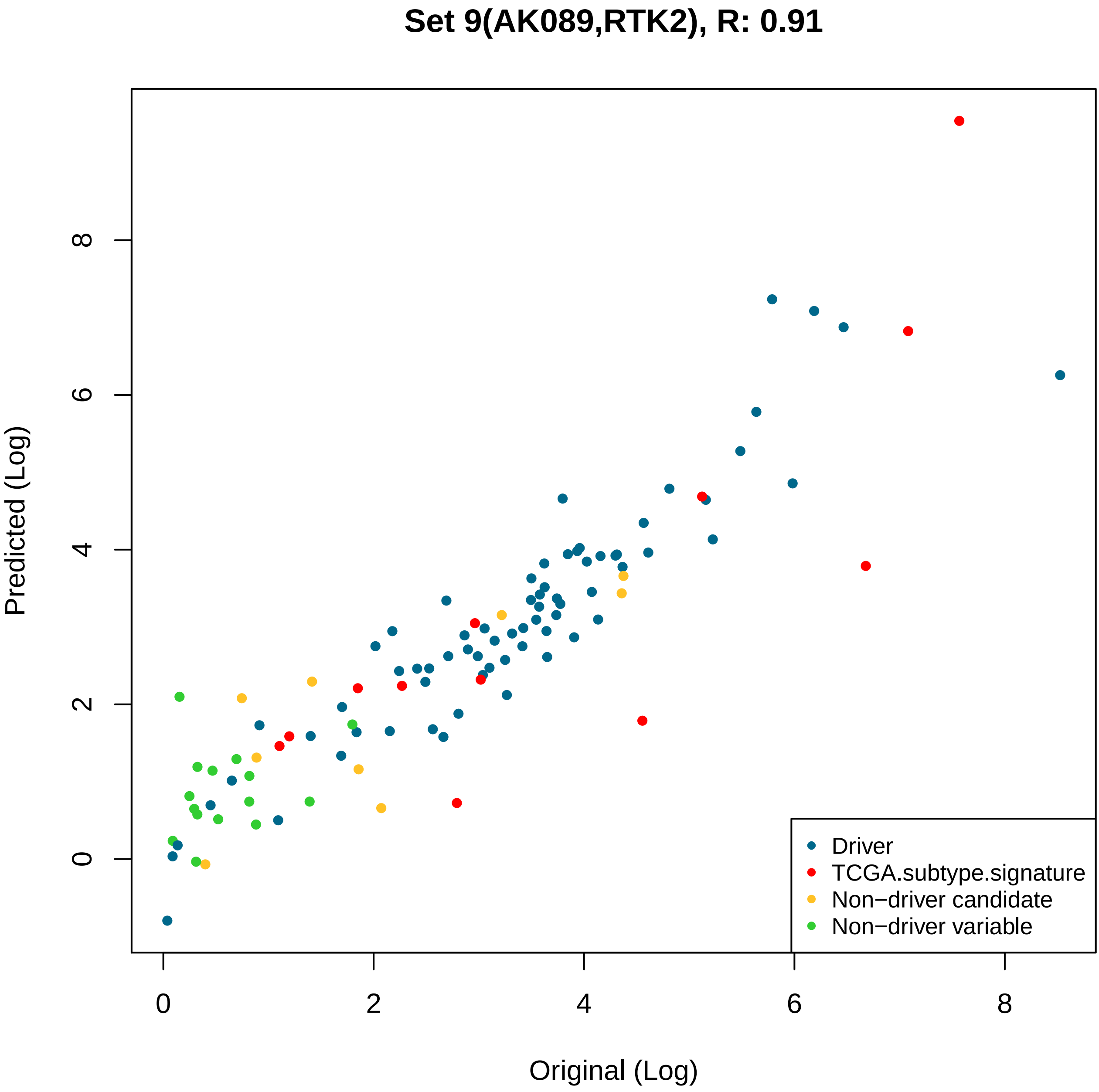

Set 9(AK089,RTK2), R: 0.91
Predicted (Log)
Original (Log)
0
2
4
6
8
0
2
4
6
8
Driver
TCGA.subtype.signature
Non−driver candidate
Non−driver variable

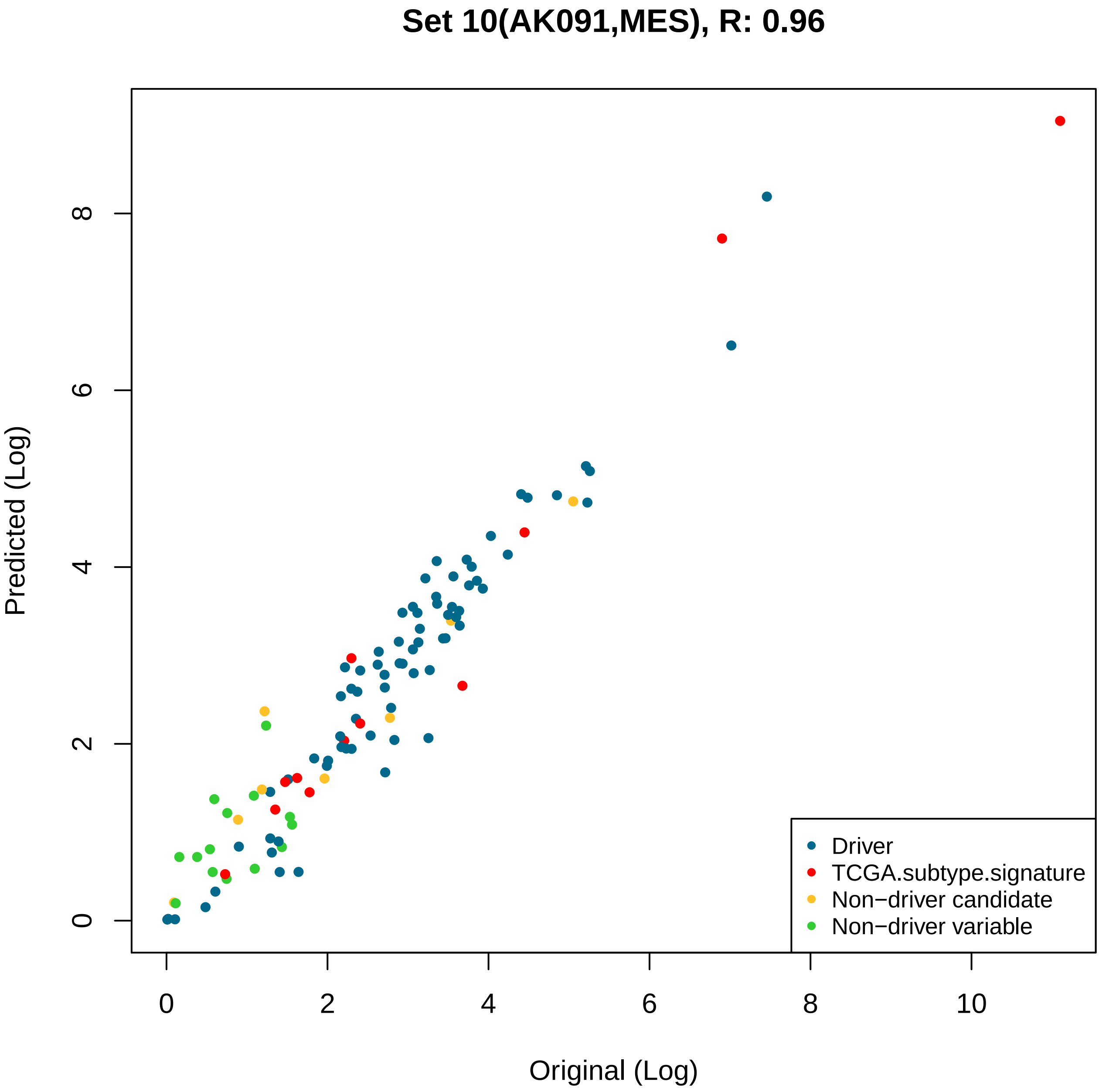

Set 10(AK091,MES), R: 0.96
Predicted (Log)
Original (Log)
0
2
4
6
8
10
Driver
TCGA.subtype.signature
Non−driver candidate
Non−driver variable

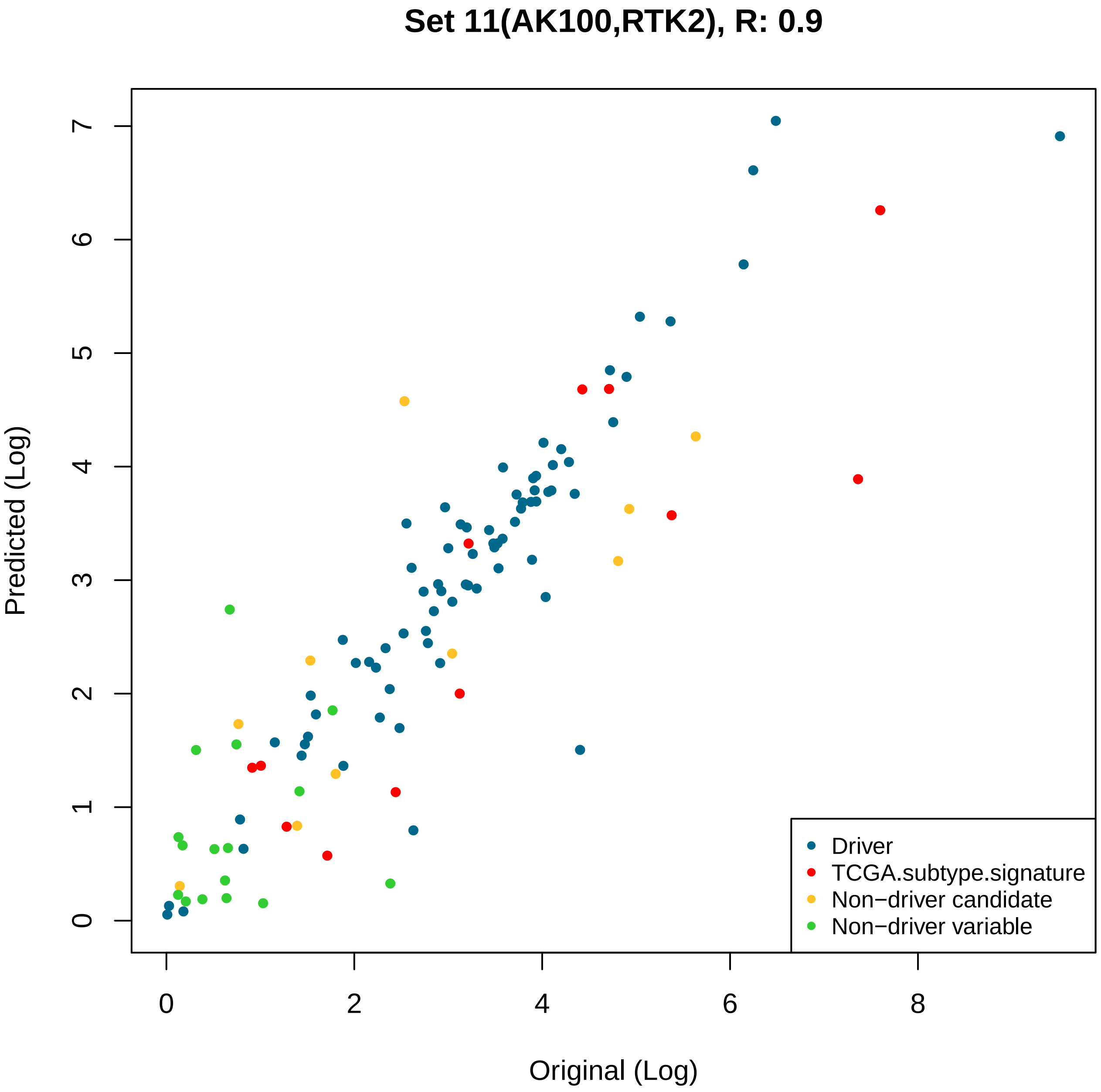

Set 11(AK100,RTK2), R: 0.9
Predicted (Log)
Original (Log)
0
1
2
3
4
5
6
7
0
2
4
6
8
Driver
TCGA.subtype.signature
Non−driver candidate
Non−driver variable

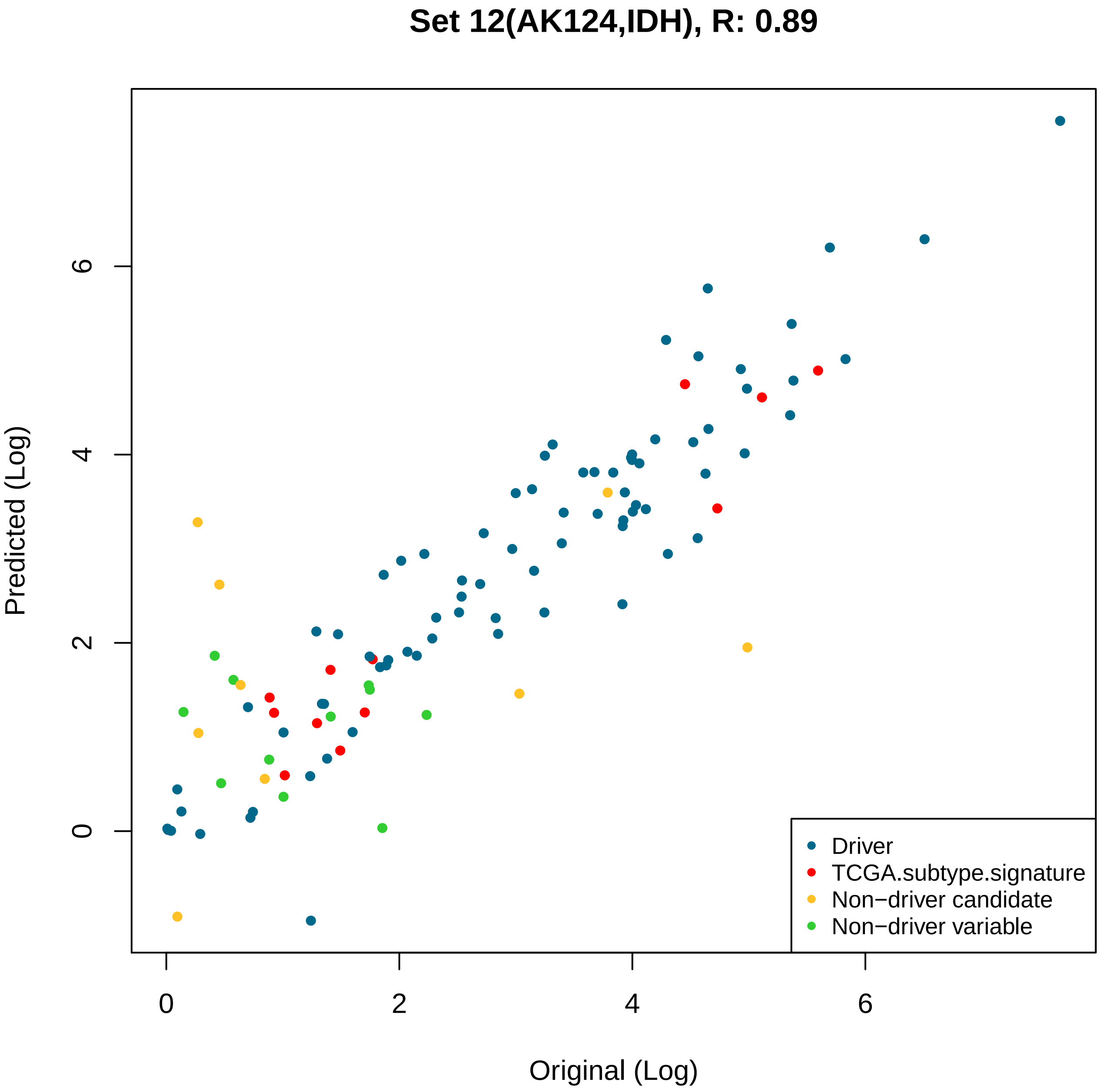

Set 12(AK124,IDH), R: 0.89
Predicted (Log)
Original (Log)
0
2
4
6
0
2
4
6
Driver
TCGA.subtype.signature
Non−driver candidate
Non−driver variable

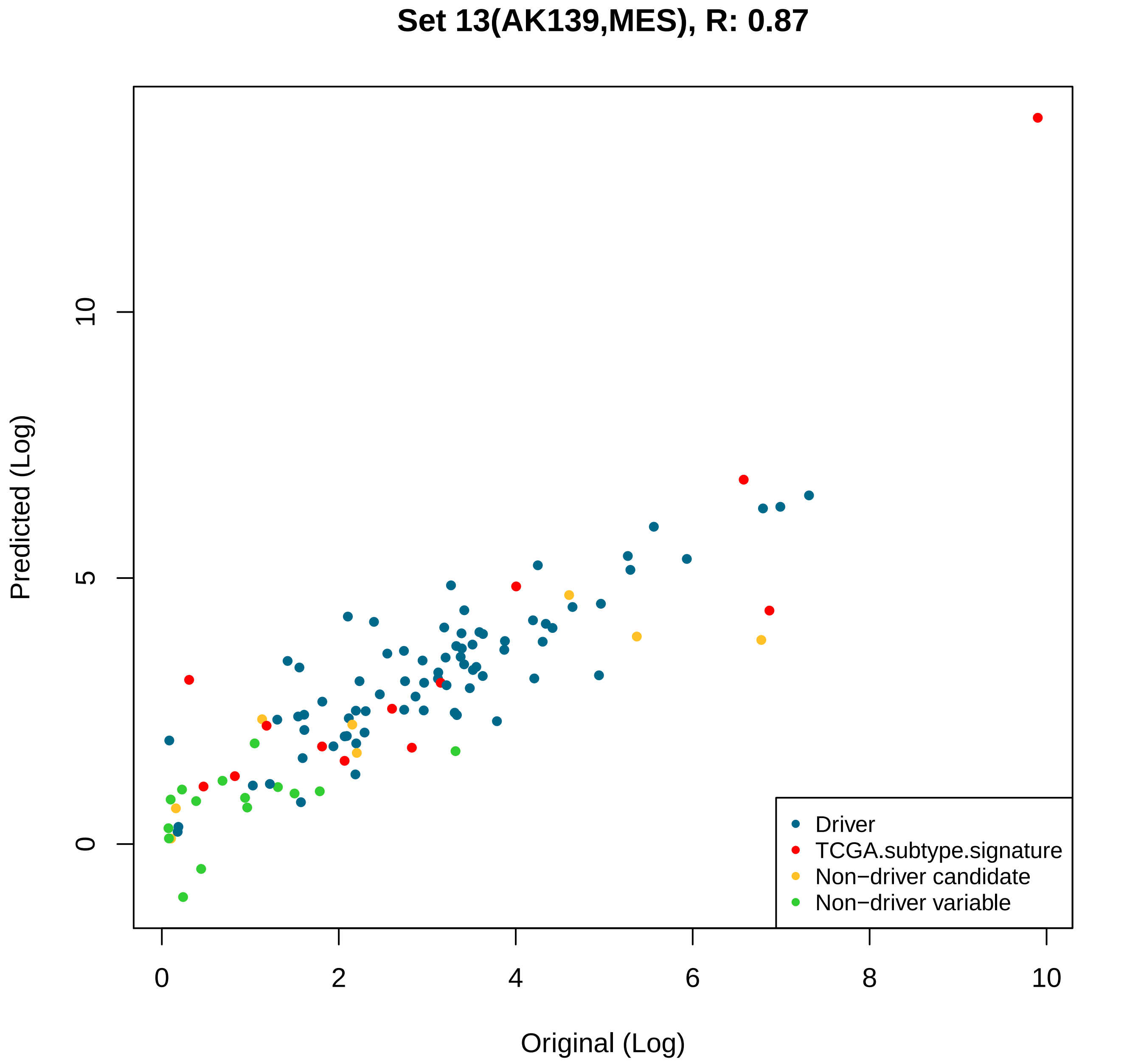

Set 13(AK139,MES), R: 0.87
Predicted (Log)
Original (Log)
0
5
10
0
2
4
6
8
10
Driver
TCGA.subtype.signature
Non−driver candidate
Non−driver variable

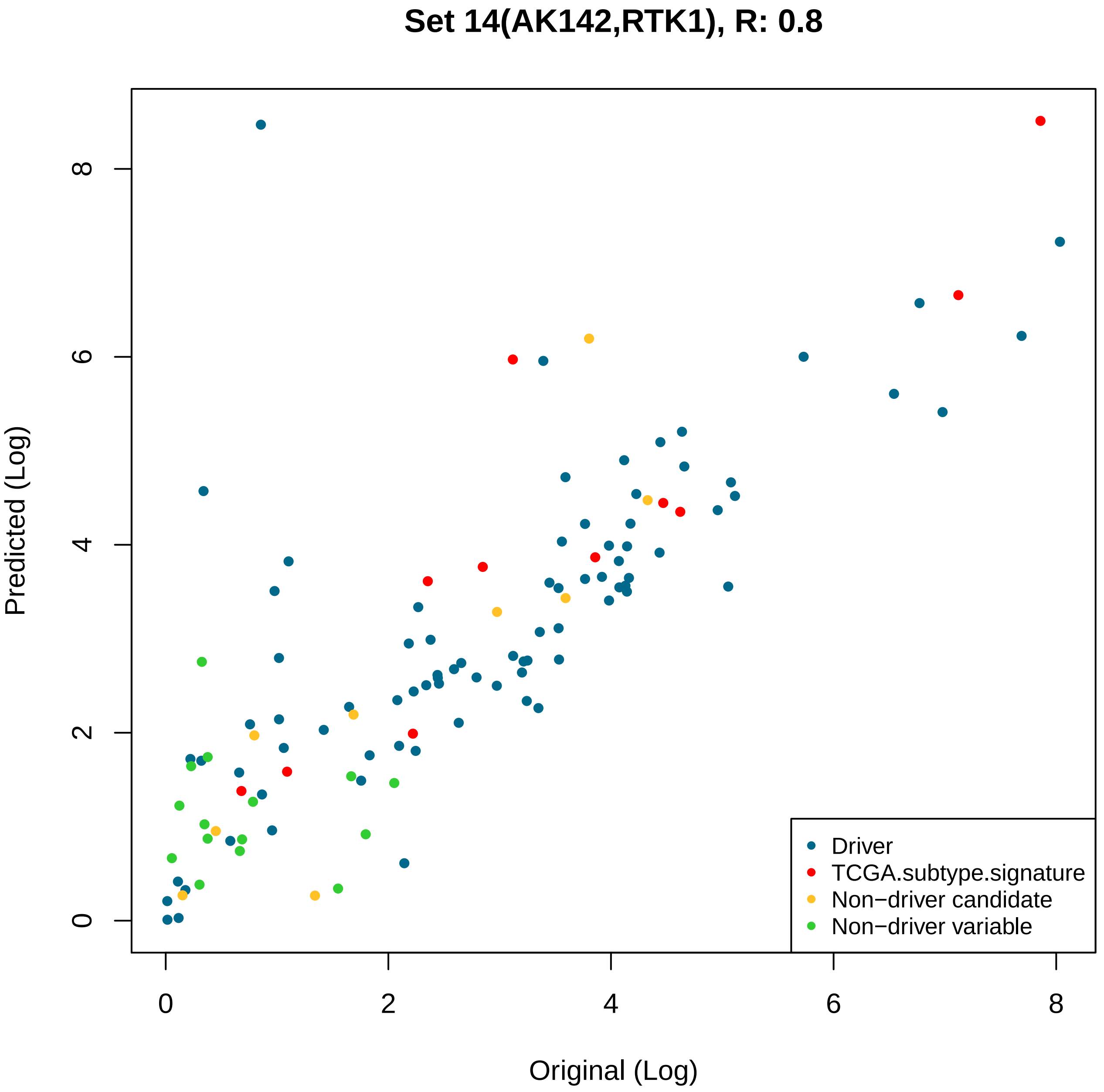
Set 14(AK142,RTK1), R: 0.8
Predicted (Log)
Original (Log)
0
2
4
6
8
Driver
TCGA.subtype.signature
Non−driver candidate
Non−driver variable

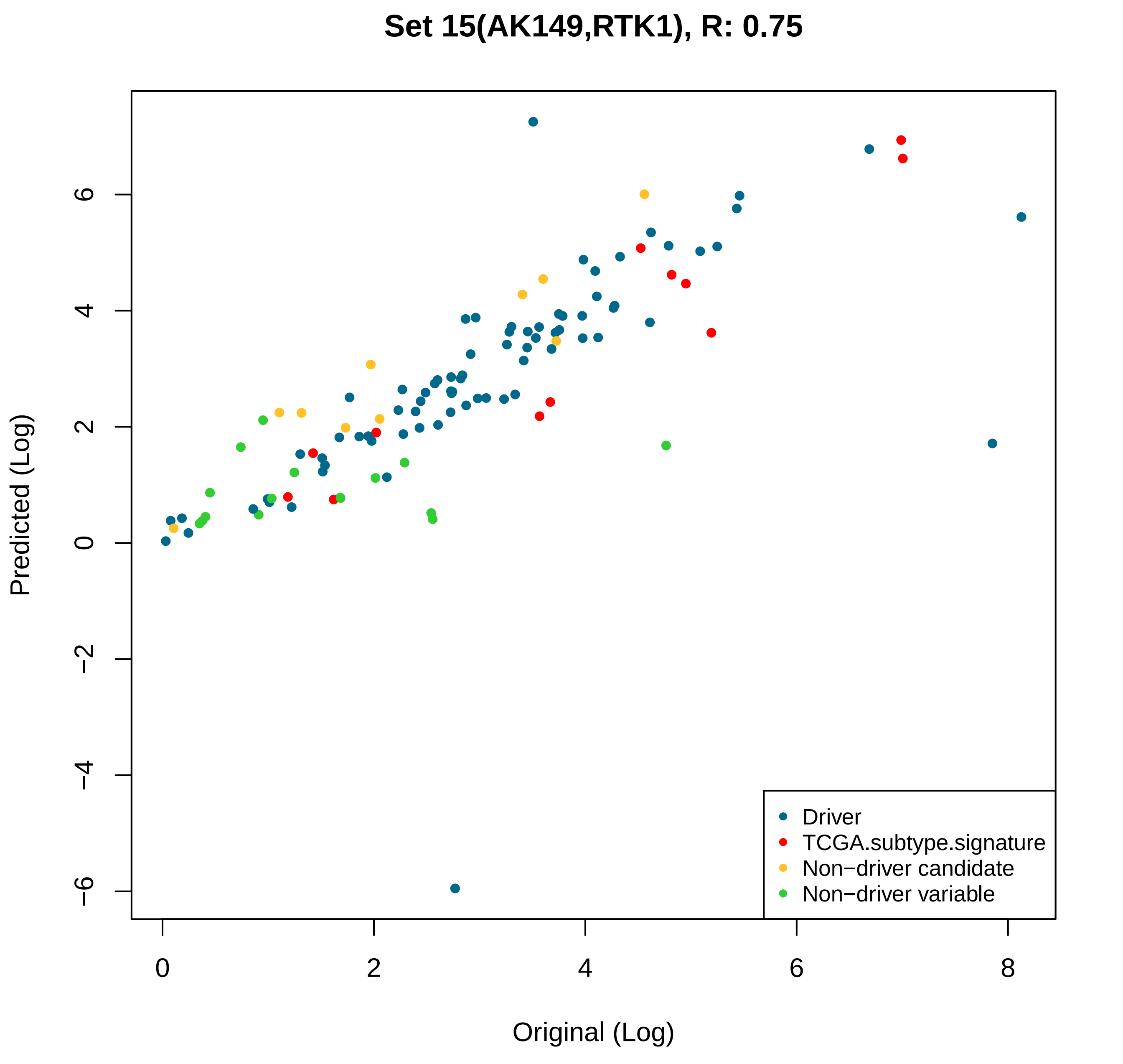

Set 15(AK149,RTK1), R: 0.75
Predicted (Log)
Original (Log)
−6
−4
−2
0
2
4
6
0
2
4
6
8
Driver
TCGA.subtype.signature
Non−driver candidate
Non−driver variable

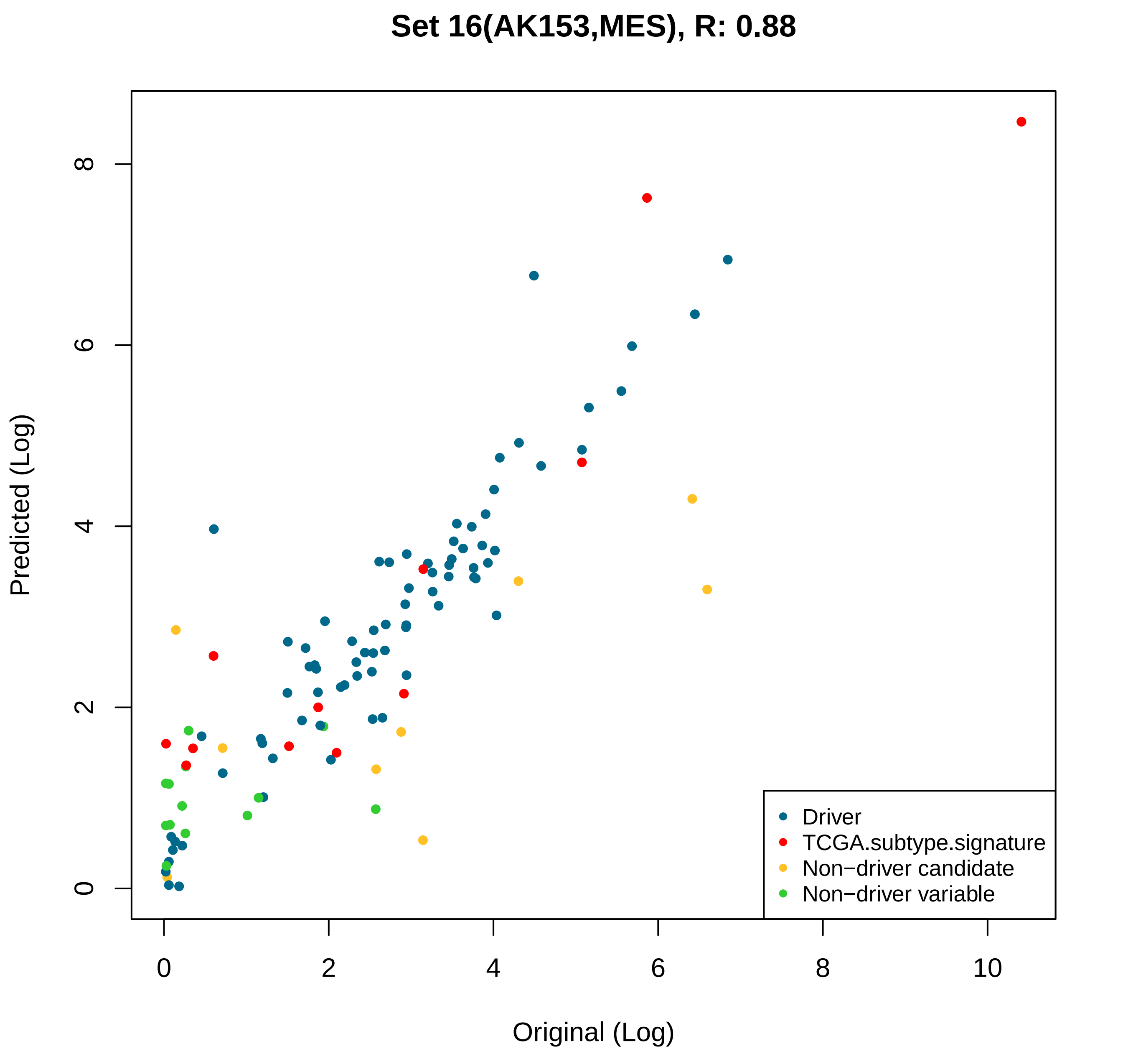
Set 16(AK153,MES), R: 0.88
Predicted (Log)
Original (Log)
0
2
4
6
8
10
Driver
TCGA.subtype.signature
Non−driver candidate
Non−driver variable

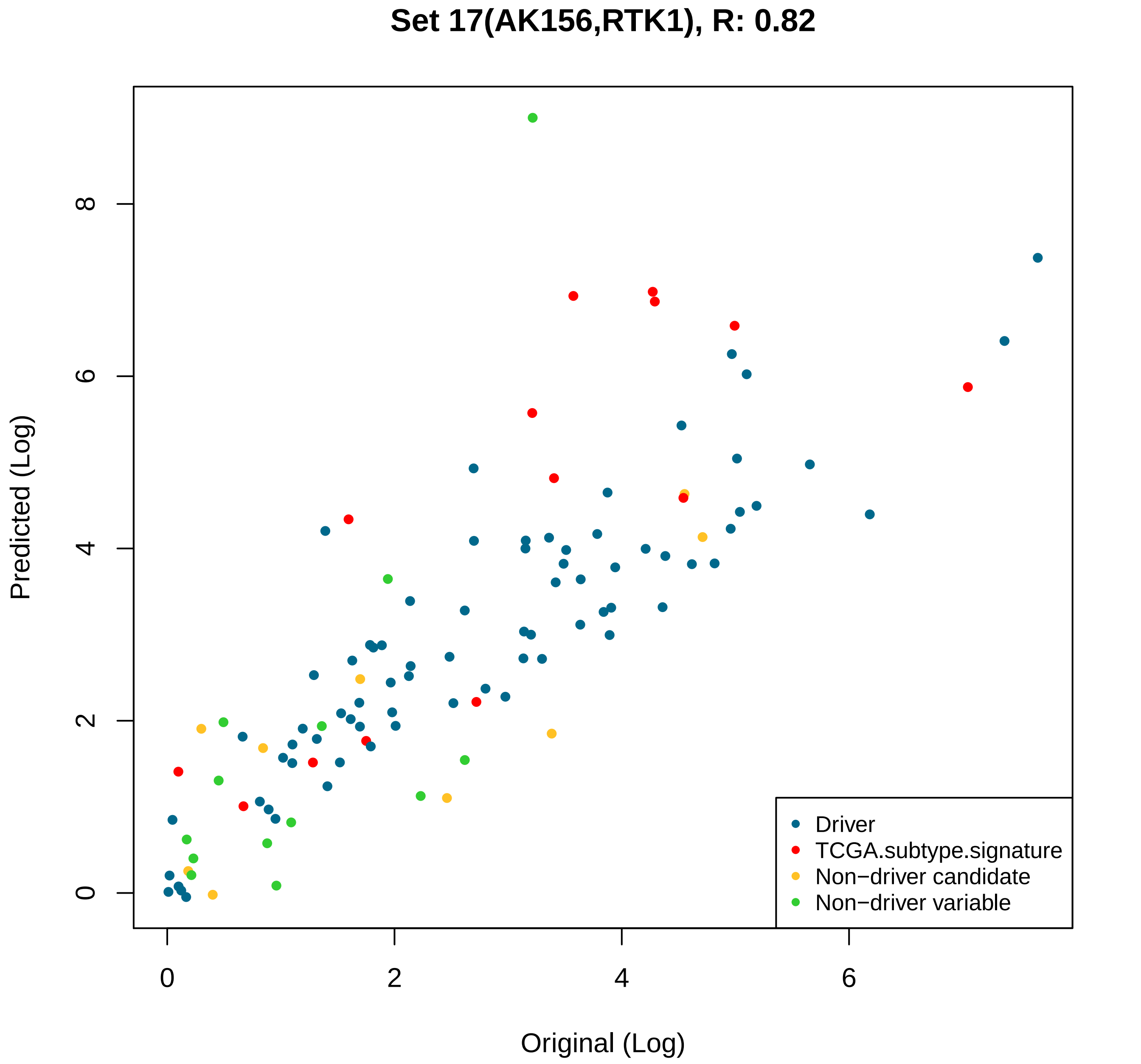

Set 17(AK156,RTK1), R: 0.82
Predicted (Log)
Original (Log)
0
2
4
6
8
0
2
4
6
Driver
TCGA.subtype.signature
Non−driver candidate
Non−driver variable

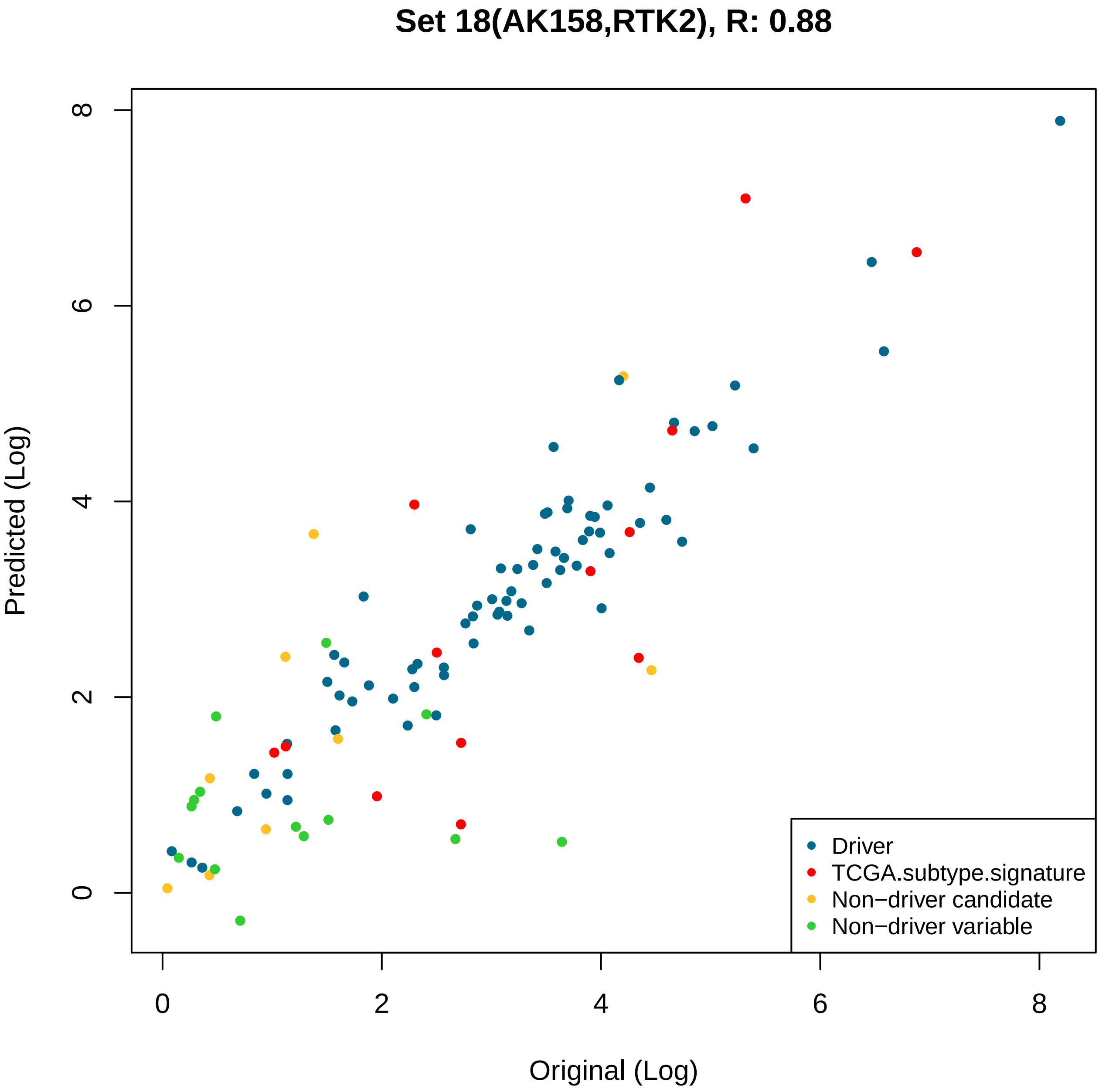
Set 18(AK158,RTK2), R: 0.88
Predicted (Log)
Original (Log)
0
2
4
6
8
0
2
4
6
8
Driver
TCGA.subtype.signature
Non−driver candidate
Non−driver variable

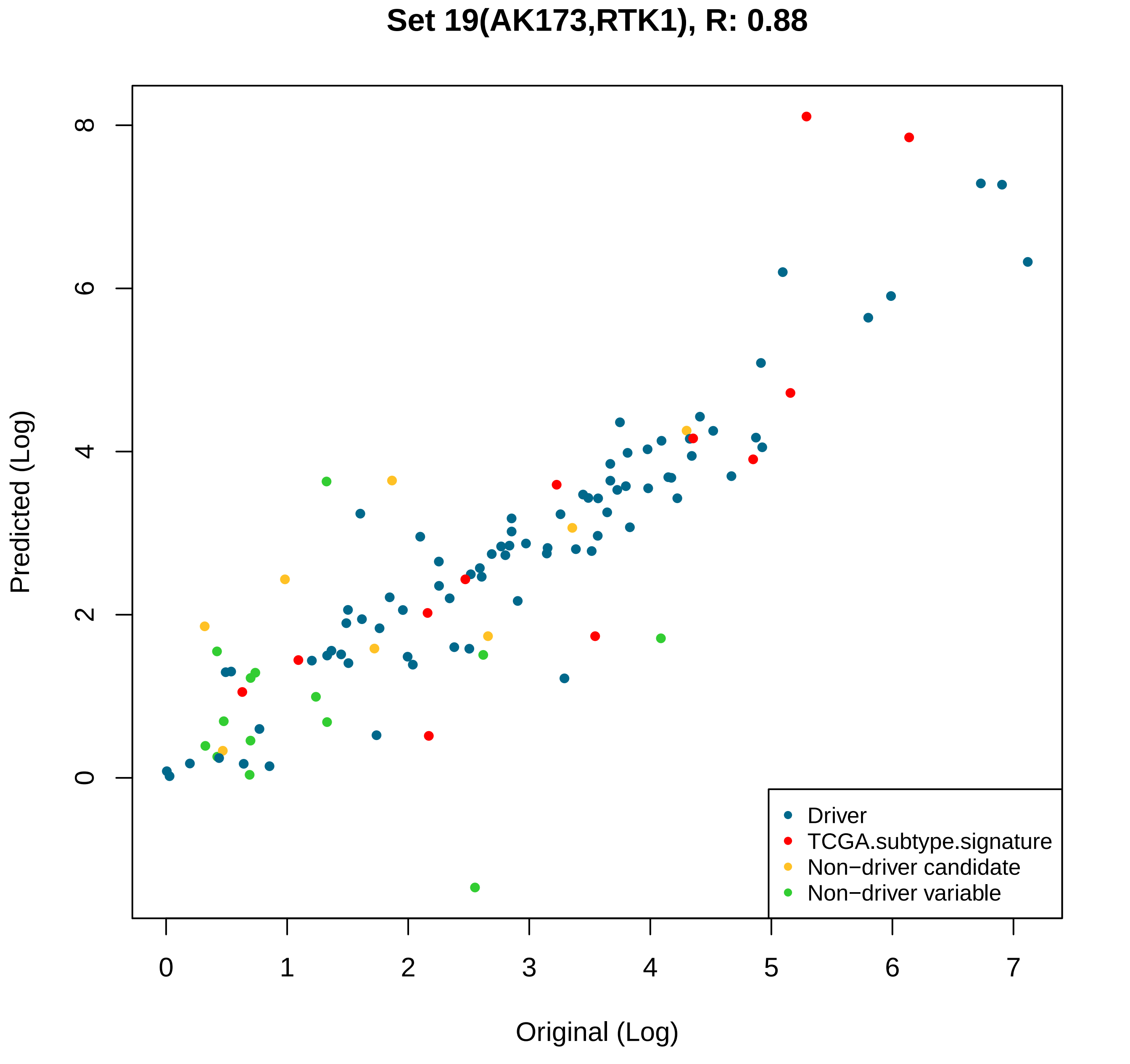

Set 19(AK173,RTK1), R: 0.88
Predicted (Log)
Original (Log)
0
2
4
6
8
0
1
2
3
4
5
6
7
Driver
TCGA.subtype.signature
Non−driver candidate
Non−driver variable

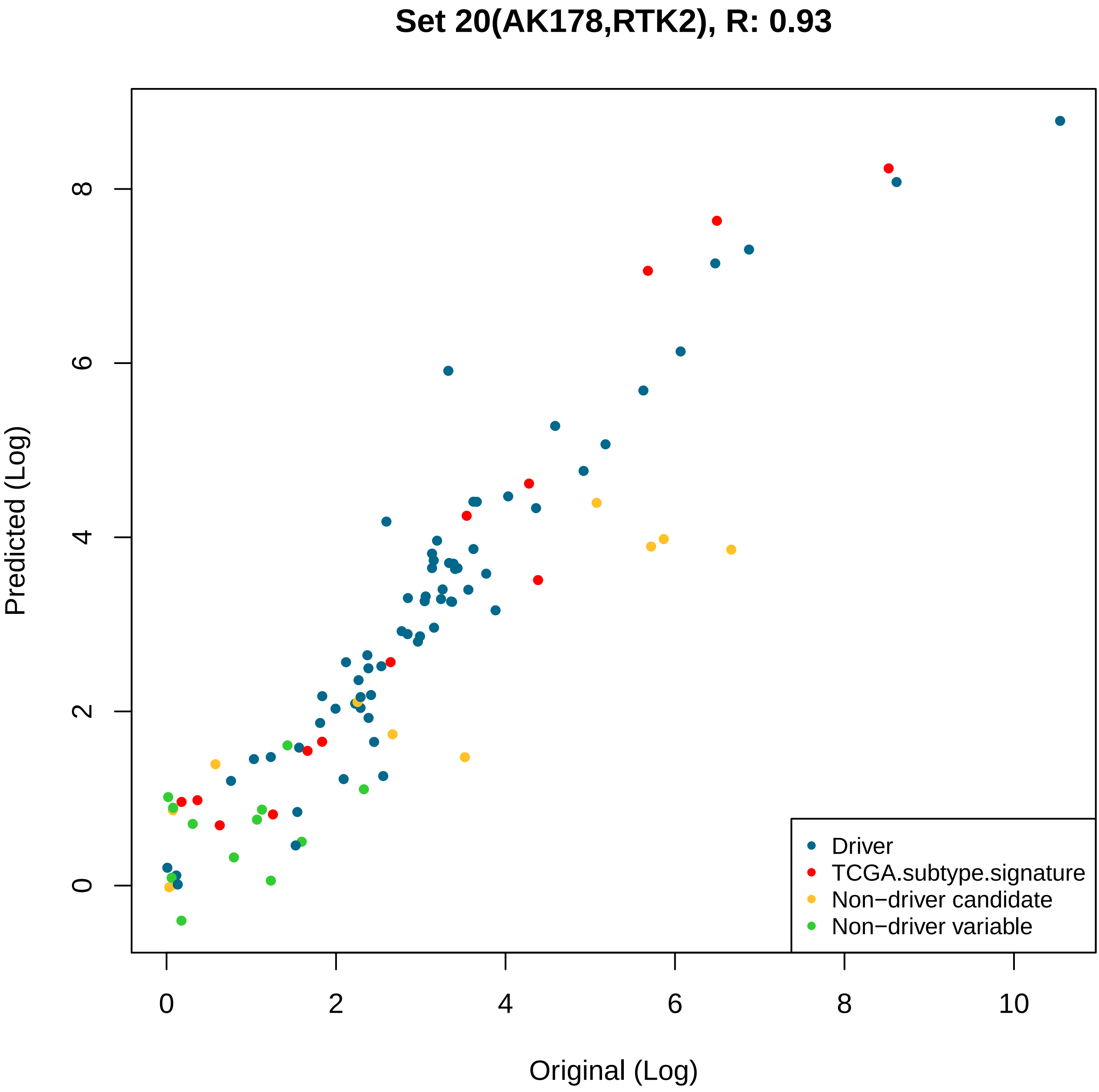

Set 20(AK178,RTK2), R: 0.93
Predicted (Log)
Original (Log)
0
2
4
6
8
10
Driver
TCGA.subtype.signature
Non−driver candidate
Non−driver variable

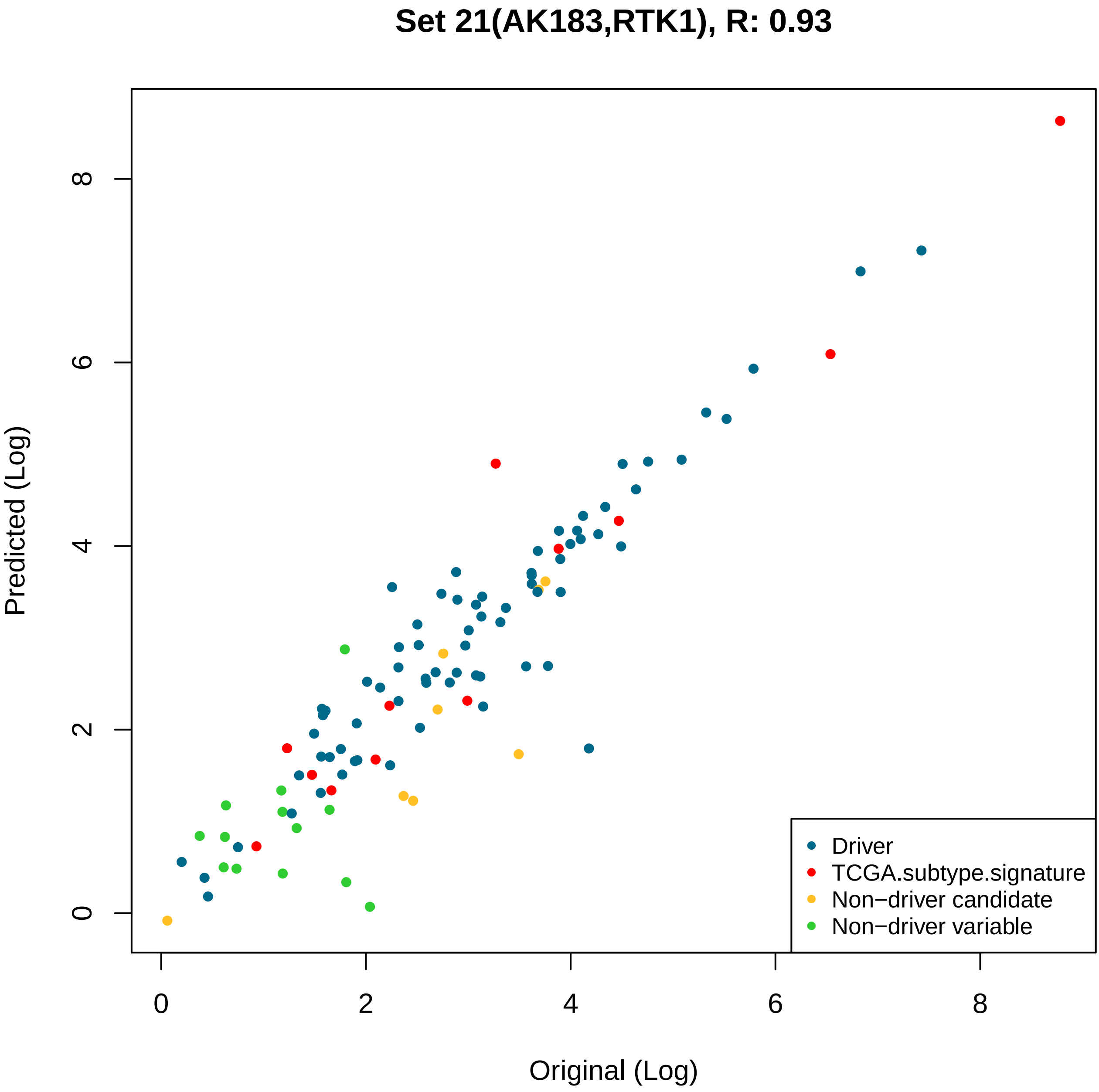
Set 21(AK183,RTK1), R: 0.93
Predicted (Log)
Original (Log)
0
2
4
6
8
Driver
TCGA.subtype.signature
Non−driver candidate
Non−driver variable

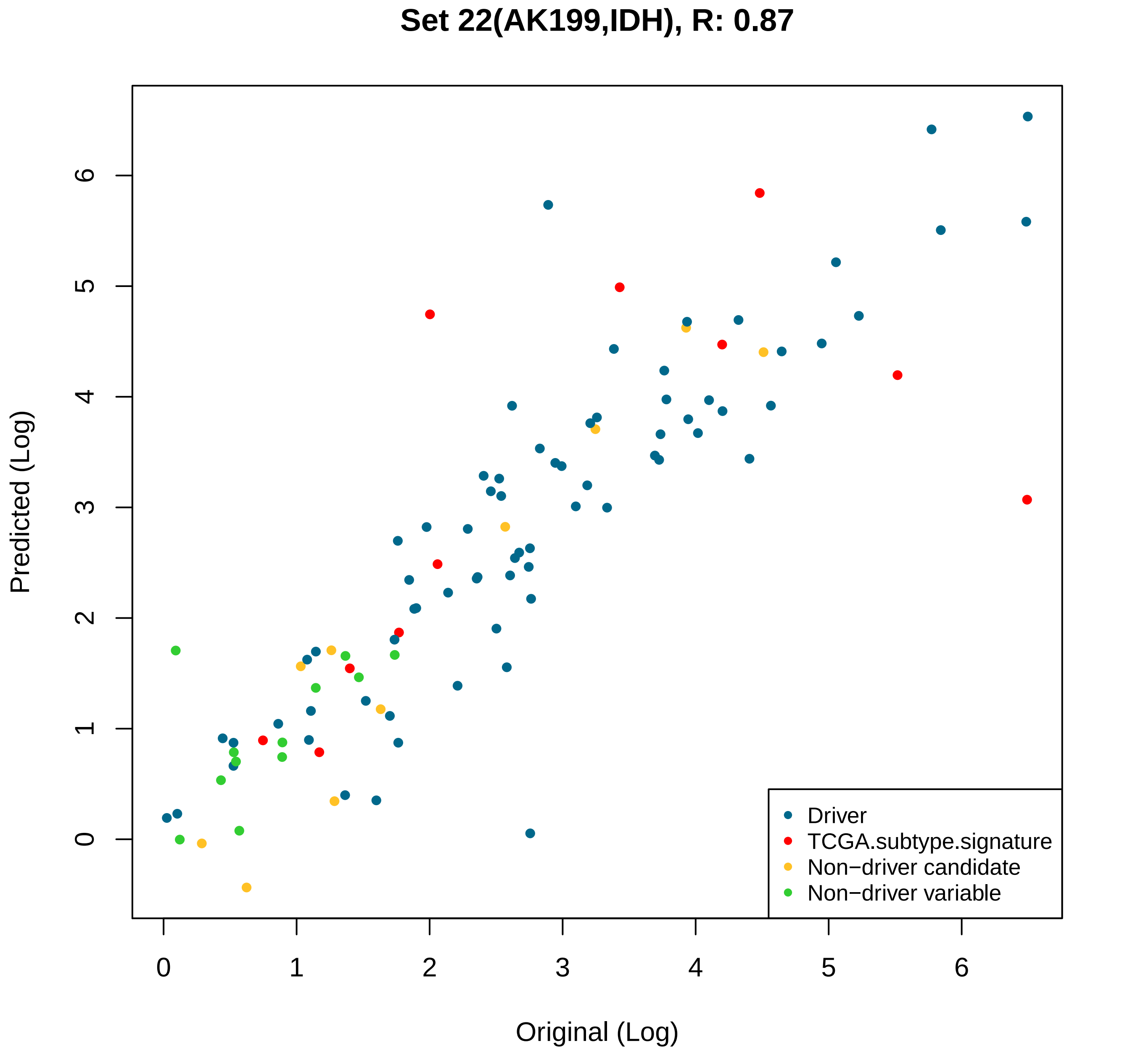
Set 22(AK199,IDH), R: 0.87
Predicted (Log)
Original (Log)
0
1
2
3
4
5
6
Driver
TCGA.subtype.signature
Non−driver candidate
Non−driver variable

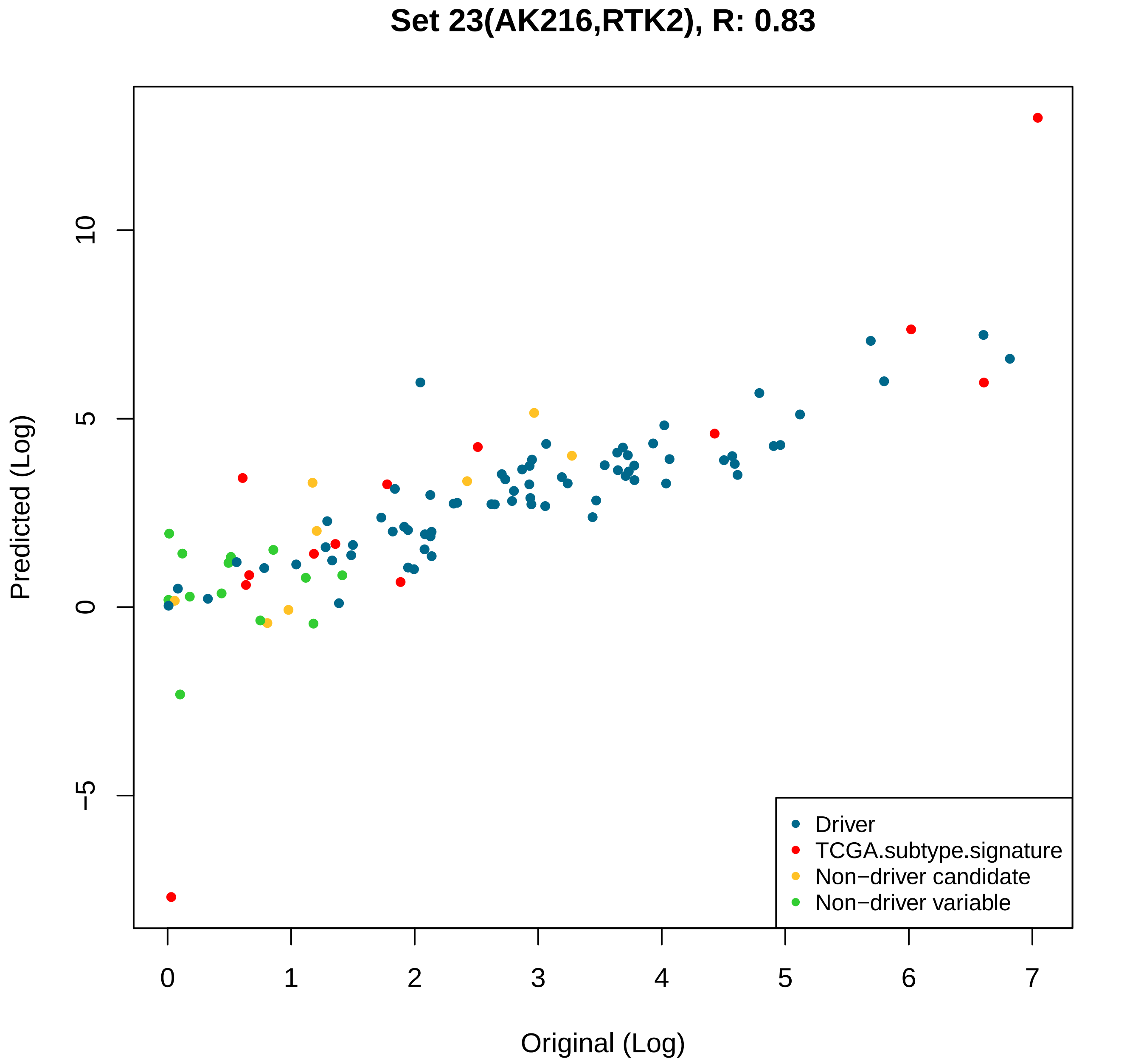

Set 23(AK216,RTK2), R: 0.83
Predicted (Log)
Original (Log)
−5
0
5
10
0
1
2
3
4
5
6
7
Driver
TCGA.subtype.signature
Non−driver candidate
Non−driver variable

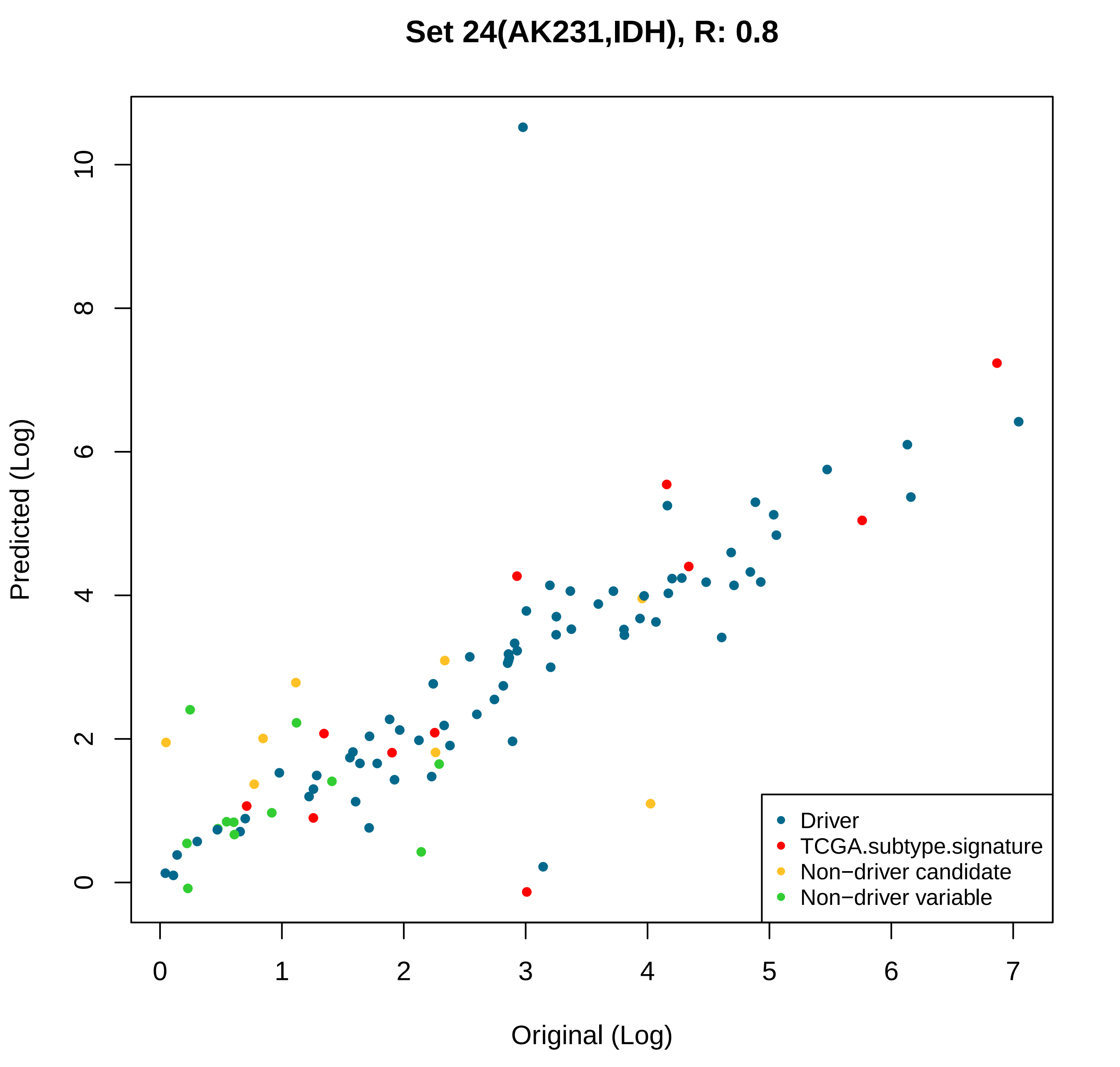

Set 24(AK231,IDH), R: 0.8
Predicted (Log)
Original (Log)
0
2
4
6
8
10
0
1
2
3
4
5
6
7
Driver
TCGA.subtype.signature
Non−driver candidate
Non−driver variable
