# Supplementary material for: Methylation-directed regulatory networks determine enhancing and silencing of mutation disease driver genes and explain inter-patient expression variation
Source: Genome Biol. 2023 Nov 28;24:264. doi: 10.1186/s13059-023-03094-6 (PMC10683314; doi:10.1186/s13059-023-03094-6)
Supplement: Supplementary file 6 — Additional file 6. Review history. [file 13059_2023_3094_MOESM6_ESM.docx]

**Review history**

**First round of review**

**Reviewer 1**

In the manuscript entitled "Methylation-directed regulatory networks determine enhancing and silencing of disease driver-genes and explain inter-patient expression variation," Edrei and colleagues use innovative techniques and analyses to survey how DNA methylation of enhancers and silencers affects gene expression in GBM patients. There is a lot of information to digest upon reading this report, but the authors provided a substantial set of graphics and summaries in display items to describe their experimental procedures. Overall, this is an interesting manuscript, however, the questions below should be addressed:

1. The selection of 125 GBM driver genes is unclear. It seems from the Methods section that these are selected based on mutation profiles in primary GBM and other tumor types. Were any epigenetic target genes included in this analysis? From a disease perspective and there are many epigenetic disease drivers of GBM that are not discussed here. If not, then it makes sense to adjust the article title to classify the driver genes as "mutation driver genes".

2. Is it correct that three GBM cell lines/samples were used for the initial analysis? There are several molecular subsets of GBM - G-CIMP and G-CIMP-low are recent examples of novel DNA methylation based molecular subgroups. How were tumor samples selected? Were samples with IDH mutations and/or MGMT DNA hypermethylation selected/not selected?

3. How do the authors describe the mechanism of how the regulation of enhancers and silencers occurs within these drivers - is there a summary graphic that can be included that provides an overview summary as to how regulation of these driver genes correlates with tumorigenesis and progression? Do the authors argue that regulation of these driver genes by enhancer/silencer DNA methylation is the cause of inter-patient heterogeneity with respect to driver gene penetrance in human cancers? How does regulation of driver genes affect their role as drivers in GBM and potentially other cancers?

4. Since promoters and gene bodies were removed from analyses that identified cis-regulatory circuits between methylation sites and control genes, how were enhancers/silencers linked to a specific gene? Were these simply within the 2Mb window around the promoter of each gene? How were other genes within these regions evaluated for their association with the enhancers identified in the 2Mb windows? Was the expression of each enhancer correlated to each driver gene window to look for associations with DNA methylation? Perhaps the authors can clarify how these interactions were characterized.

5. From the analyses of SMO and TNFAIP3 (Fig 4-5),it appears that negative associations are much broader and with reduced correlation than positive associations - what are the possible reasons for this? Does this implicate a larger impact of variation of these specific changes on disease driver effects?

**Reviewer 2**

In the manuscript "Methylation-directed regulatory networks determine enhancing and silencing of disease driver genes and explain inter-patient expression variation" Edrei et al. identify gene regulatory networks around glioblastoma-driver genes composed of enhancers and silencers and investigate the role of DNA methylation in the regulation of the activity of these elements. They show additivity of the different enhancing and repressing elements leading to variability of the expression of the associated gene and correlating with phenotypic variability among the analyzed patients.

The study addresses a timely topic and is very well-performed. The results are to the best of my knowledge completely novel and can have a major impact on the explanation of phenotypic variation in different complex diseases opening many new research avenues. The study is therefore of interest to the broad readership of Genome Biology. The study is very dense with a high number experiments performed to support the conclusions of the authors and a lot of data included.
There are some limitations to the study such as the arbitrary size limits for the region for which are tested for enhancers and silencers which would take into account large DNA loops bringing regulatory elements in proximity to gene promoters. These limitations are clearly discussed and while perhaps not being complete, the study already yields a large number of regulatory regions correlating collectively with the gene activity.


Some recent publications / reviews on the topic are not referenced and the authors should update the introduction and discussion. E.g. it might be useful to add the reference of a recent review on the topic of silencers (Pang et al., Nat Rev Mol Cell Biol, 2023) and the authors might particularly consider adding the reference of Huang & Ovcharenko, Genome Res 2022, who studied the properties of enhancer /silencer elements as well as the transition between the two classes of regulatory elements investigating chromatin accessibility, but not DNA methylation, in human T cells and compare the results obtained in their study with those by Huang and Ovcharenko.

While DNA methylation clearly plays a role in the finetuning of the activity, the sequence themselves seem to be sufficient to induce gene activation or silencing as measured by the TAS score in the experimental system. This raises the question if the authors see differential TF binding motifs between gene expression enhancing and repressing elements and/or overlap of different motifs between repressing and activating TFs in those switching. Might these motifs be used to identify potentially activating or repressing regulatory circuits or is there no enrichment of specific TFBS for the glioblastoma regulatory circuits ? Are these - if any - TFs known to be deregulated in glioblastoma and could explain the observations and is their binding supposed to be methylation-dependent and /methylation sensitive ? Alternatively, could expression of TFs explain the observed patient variability in expression ?


Minor:

Should line 128 not read , averaging 4.5 units per gene (instead of 2.5), as there are 2.1 positive and 2.4 negative associations on average ?

There are some errors in the ToC of the supplementary material with S9 Fig not defined and the names of other figures missing, please check.

**Authors’ response to reviewers**

Reviewer #1
Point 1. The selection of 125 GBM driver genes is unclear. It seems from the Methods section that these are selected based on mutation profiles in primary GBM and other tumor types. Were any epigenetic target genes included in this analysis? From a disease perspective and there are many epigenetic disease drivers of GBM that are not discussed here. If not, then it makes sense to adjust the article title to classify the driver genes as "mutation driver genes".

Response: We agree with the reviewer that the term "mutation driver genes" is better describing the studied genes. Indeed, in this paper we have focused on genes that are frequently altered by sequence mutations. We accept the reviewer’s suggestion and adjusted the article's title as suggested.

Point 2. Is it correct that three GBM cell lines/samples were used for the initial analysis? There are several molecular subsets of GBM - G-CIMP and G-CIMP-low are recent examples of novel DNA methylation based molecular subgroups. How were tumor samples selected? Were samples with IDH mutations and/or MGMT DNA hypermethylation selected/not selected?

Response: Yes, the analysis was done in GBM samples that represent four main GBM subgroups, based on both molecular and DNA hypermethylation data, as described (reference #62 in the revised manuscript). An equal representation was given to each of the subgroups, including the IDH subgroup (Table S5). We have added a description of the included GBM subgroups in the Methods>Input materials section (Lines 10-11).

Point 3. How do the authors describe the mechanism of how the regulation of enhancers and silencers occurs within these drivers - is there a summary graphic that can be included that provides an overview summary as to how regulation of these driver genes correlates with tumorigenesis and progression? Do the authors argue that regulation of these driver genes by enhancer/silencer DNA methylation is the cause of inter-patient heterogeneity with respect to driver gene penetrance in human cancers? How does regulation of driver genes affect their role as drivers in GBM and potentially other cancers?

Response: We have added a summary graphic and related text to better convey our view of the above issues.

Explanation: Our results suggest that DNA methylation either directly affects, or is a very close bystander, of the binding profiles of transcriptional activators and repressors to regulatory DNA sites. In turn, the binding of these factors determined the transcriptional activity of genes, which involve in cancer initiation and progression pathways. We further showed that the methylation profiles at key regulatory sites efficiently predict the variation in the activity level of these genes among GBM patients. While the experimental assay supports a direct, causative role for methylation in the setting of these expression profiles, we cannot rule out a more complex mechanism in actual chromatin.

Modifications to the manuscript
• Panel D in Figure 1 and its legend were modified to provide an overview summary of how enhancers/silencers DNA methylation describe inter-patient drive-gene heterogeneity. The revised manuscript also included a graphic summary of the functions of the studied genes during cancer initiation and progression (Supplemental Figure S14).
• A text section describing our understanding of the mentioned issues was added (lines 245-252).

Point 4. Since promoters and gene bodies were removed from analyses that identified cis-regulatory circuits between methylation sites and control genes, how were enhancers/silencers linked to a specific gene? Were these simply within the 2Mb window around the promoter of each gene? How were other genes within these regions evaluated for their association with the enhancers identified in the 2Mb windows? Was the expression of each enhancer correlated to each driver gene window to look for associations with DNA methylation? Perhaps the authors can clarify how these interactions were characterized.

Response: We have linked enhancers and silencers to specific genes based on the previously-established association between enhancer methylation levels and the expression levels of associated genes [Aran, Sabato & Hellman, Genome Biol 2013; Aran & Hellman, Cell 2013]. These interactions were evaluated for each of the targeted methylation sites excluding promoter and gene-body sites, versus any one of the genes within the 2MB intervals. We have clarified this issue in the revised manuscript (lines 101-102).

Point 5. From the analyses of SMO and TNFAIP3 (Fig 4-5), it appears that negative associations are much broader and with reduced correlation than positive associations - what are the possible reasons for this? Does this implicate a larger impact of variation of these specific changes on disease driver effects?

Response: It is true that negative correlations seemed to be more abundant than positive correlations, possibly due to an excess of bound gene activators over gene repressors. This tendency may be seen in the example genes (Figures 4-5), as well as across the studied genes (Additional File 1, Supplementary Table S9, Table S10), and it is in line with the excess of functional enhancers that we observed using the functional assay (Figure 2b). Interestingly, many gene expression models relay on both positive and negative associations (Figure 6c), suggesting that gene regulation often required the both. We have added this interesting observation to the discussion section (line 338-343).

Reviewer #2
Point 1. Some recent publications / reviews on the topic are not referenced and the authors should update the introduction and discussion. E.g. it might be useful to add the reference of a recent review on the topic of silencers (Pang et al., Nat Rev Mol Cell Biol, 2023) and the authors might particularly consider adding the reference of Huang & Ovcharenko, Genome Res 2022, who studied the properties of enhancer /silencer elements as well as the transition between the two classes of regulatory elements investigating chromatin accessibility, but not DNA methylation, in human T cells and compare the results obtained in their study with those by Huang and Ovcharenko.

Response: We have referred to the suggested publications and discussed our results compared with these reports
Modifications to the manuscript
• In the revised manuscript we have further describe and discussed these issues (lines 268-314).
• We added the two suggested references (Ref #17 Pang et al., Nat Rev Mol Cell Biol, 2023, and ref #23 Huang & Ovcharenko, Genome Res 2022)

Point 2. While DNA methylation clearly plays a role in the fine tuning of the activity, the sequence themselves seem to be sufficient to induce gene activation or silencing as measured by the TAS score in the experimental system. This raises the question if the authors see differential TF binding motifs between gene expression enhancing and repressing elements and/or overlap of different motifs between repressing and activating TFs in those switching. Might these motifs be used to identify potentially activating or repressing regulatory circuits or is there no enrichment of specific TFBS for the glioblastoma regulatory circuits? Are these - if any - TFs known to be deregulated in glioblastoma and could explain the observations and is their binding supposed to be methylation-dependent and /methylation sensitive? Alternatively, could expression of TFs explain the observed patient variability in expression?

Response: We have explored these interesting questions during our study. Indeed, it was previously shown that silencers are relatively enriched by sequence binding motifs of transcriptional repressors. However, it was also shown that of regulatory sites bind both activators and repressors over alternate cellular conditions, and may swap between silencing and enhancing effects in alternate cellular contexts. Our analysis showed a weak tendency of the positive sites towered repressor binding motifs, including EZH2 and REST motifs, however both positive and negative sites encompass binding motifs of repressors and activators, and no significant difference revealed. Moreover, positive and negative sites bind both activators and repressors, over a range of tissues and cell types (Fig. 2e). We have further demonstrated the ability of regulatory sites to swap between enhancing and silencing functioning upon altering their DNA methylation (Fig. 2g), suggesting that DNA methylation is both required and sufficient to induce functionality switching, at lease under experimental conditions. Due to this effect, methylation of cis-regulatory sites may generate both enhancing or silencing of gene expression, depending of the specific regulatory context. Whether this is a unique feature of methylation-depended regulatory sites, or is it also applied to other sites, remined an open question.

Modifications to the manuscript
In the revised manuscript we have further describe and discussed these issues (lines 268-314).

Minor:
- Should line 128 not read, averaging 4.5 units per gene (instead of 2.5), as there are 2.1 positive and 2.4 negative associations on average?
Response: Fixed (lines 129-130).

- There are some errors in the ToC of the supplementary material with S9 Fig not defined and the names of other figures missing, please check.
Response: Fixed.

Editorials:
• The main figures were provided in editable formats and resized as follow: Figures 1, 3-5: Half page. Figures 2, 6: Full page.
• A new research grant for A.H. was added to the Funding section.
• Acknowledgments and Author information sections were added.
• The text related to the GEO secure token was deleted (The secure token itself is still active, we will allow open access to the deposited data once accepted for publication).
